# First-in-Class Dual Hybrid Carbonic Anhydrase Inhibitors and Transient Receptor Potential Vanilloid 1 Agonists Revert Oxaliplatin-Induced Neuropathy

**DOI:** 10.1021/acs.jmedchem.2c01911

**Published:** 2023-01-10

**Authors:** Andrea Angeli, Laura Micheli, Fabrizio Carta, Marta Ferraroni, Tracey Pirali, Asia Fernandez Carvajal, Antonio Ferrer Montiel, Lorenzo Di Cesare Mannelli, Carla Ghelardini, Claudiu T. Supuran

**Affiliations:** † NEUROFARBA Department, Sezione di Scienze Farmaceutiche, University of Florence, via Ugo Schiff 6, 50019 Sesto Fiorentino, Florence, Italy; ‡ Pharmacology and Toxicology Section, Department of Neuroscience, Psychology, Drug Research and Child Health (NEUROFARBA), University of Florence, viale Gaetano Pieraccini 6, 50139 Firenze, Florence, Italy; § Department of Chemistry ″Ugo Schiff″, University of Florence, via della Lastruccia 3-13, I-50019 Sesto Fiorentino, Italy; ∥ Dipartimento Di Scienze del Farmaco, Università Degli Studi del Piemonte Orientale, 28100 Novara, Italy; ⊥ Instituto de Investigación, Desarrollo e Innovación en Biotecnología Sanitaria de Elche (IDiBE), Universitas Miguel Hernández, 03202 Elche, Spain

## Abstract

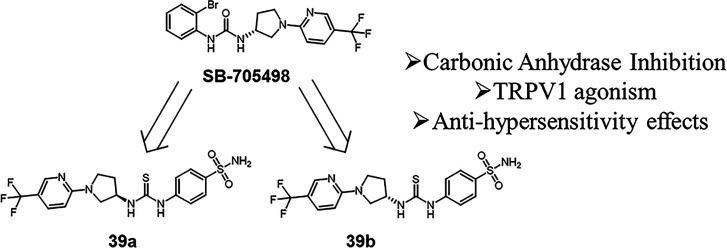

Here, we report for the first time a series of compounds potentially useful for the management of oxaliplatin-induced neuropathy (OINP) able to modulate the human Carbonic Anhydrases (hCAs) as well as the Transient Receptor Potential Vanilloid 1 (TRPV1). All compounds showed effective *in vitro* inhibition activity toward the main hCAs involved in such a pathology, whereas selected items reported moderate agonism of TRPV1. X-ray crystallographic experiments assessed the binding modes of the two enantiomers (*R*)-**37a** and (*S*)-**37b** within the hCA II cleft. Although the tails assumed diverse orientations, no appreciable effects were observed for their hCA II affinity. Similarly, the activity of (*R*)-**39a** and (*S*)-**39b** on TRPV1 was not influenced by the stereocenters. *In vivo* evaluation of the most promising derivatives (*R*)-**12a**, (*R*)-**37a**, and the two enantiomers (*R*)-**39a**, (*S*)-**39b** revealed antihypersensitivity effects in a mouse model of OINP with potent and persistent effect up to 75 min after administration.

## Introduction

Cancer is a major health threat worldwide and is estimated that more than half a million deaths in the United States alone by 2021 are directly correlated to such a disease.^1^ Nevertheless, cancer survival has improved over the last 50 years thanks to new therapeutic breakthroughs although frequent adverse effects remain.^2^ The platinum-based chemotherapy (*i.e.*, cisplatin, carboplatin, and oxaliplatin) has acquired and still retains significant importance since it is widely used within the oncological field for the management of advanced metastatic cancers (*i.e.*, colorectal, ovarian, breast, and lung as the major examples).^3^ However, several side effects are associated with platinum drugs, and among others, dose-limiting toxicity, nephrotoxicity, ototoxicity, myelosuppression, and neurotoxicity are those of major concern as often result in discontinuation of the therapy.^4,5^ For instance, peripheral neurotoxicity affects almost all patients with acute symptoms (*i.e.*, paresthesia/dysesthesias) which over time turn into chronic sensory neurotoxicity. In addition, chronic painful pathologies are highly debilitating and heavily affect life quality.^6^ To date, there are no effective options for the management of oxaliplatin-induced neuropathic pain (OINP), being only the nonsteroidal anti-inflammatory drugs (NSAIDs) and opioids able to act slightly as pain relievers although associated with important side effects.^7,8^ Although the pathophysiology of OINP is not fully understood, several reports agree that homeostasis dysfunctions of dorsal root ganglion (DRG) neurons take place.^9−12^ Such a hypothesis is consistent with the location of DRGs outside the central nervous system (CNS) and thus not protected by the blood–brain barrier.^13^ An important piece of evidence is that patients treated with oxaliplatin showed an interference with some members of the Transient Receptor Potential (TRP) channel family (such as TRPM8, TRPV1, and TRPA1) through the chelation of calcium ions by oxaliplatin metabolites (*i.e.*, oxalate). In this context, we turned our attention to TRPV1 as it recently assumed importance as a potential analgesic target since it is involved in the transmission of nociceptive stimuli by triggering an important cellular influx of Ca^2+^ ions.^14,15^

**Figure 1:**
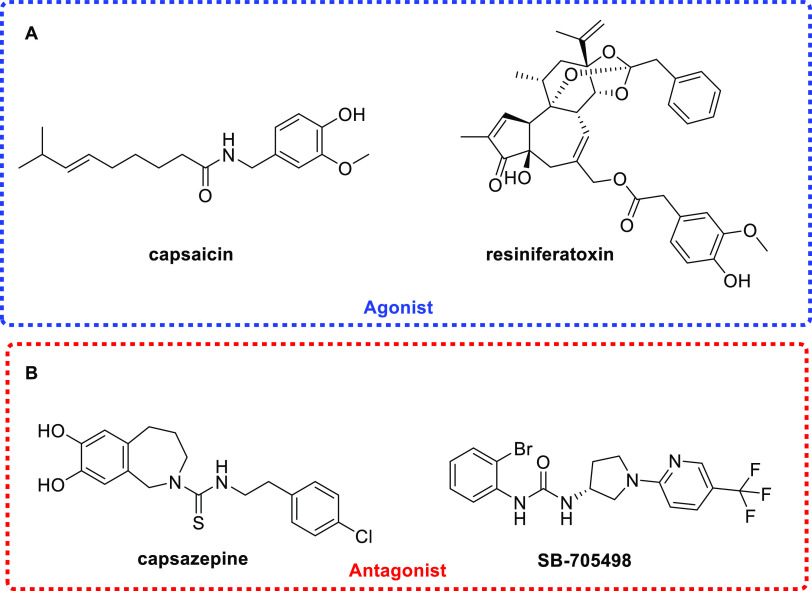
Structures of agonists (A) and antagonists (B) of TRPV1 receptor.

Desensitization of the TRPV1 receptor through its activation^16,17^ represents a promising strategy for pain management. Early attempts to manipulate TRPV1 receptor did make use of agonists such as capsaicin (Figure 1A)^18^ or resiniferatoxin (Figure 1). The latter is better considered as “molecular scalpel” since it was reported to cause prolonged TRPV1 channel opening with cytotoxicity effects evident only to sensory neurons expressing it.^19^ TRPV1 partial agonists were also effective in inducing pain relief.^20^

An alternative approach to modulate pain relief includes compounds endowed with TRPV1 antagonist features such as capsazepine or **SB-705498** in Figure 1B. In this case, the pain reversal effects were strongly associated with risks of hyperthermia and accidental burns and that did make such a route unfeasible for further development.^21^ OINPs usually are associated with uncontrolled intracellular acidification of DRG neurons as a result of the formation of metal (*i.e.*, platinum) adducts with hemoglobin.^12^ The same study reported that uncontrolled pH fluctuations by subtraction of the main pH buffering system may be reverted by inhibition of the Carbonic Anhydrase (CA, EC 4.2.1.1) isoforms therein present (*i.e.*, hCA II).^12^ Based on the seminal study from Potenzieri et al., we sought to intervene in OINP pH imbalances by making use of compounds able to inhibit the metalloenzymes CAs and activate TRPV1 receptors.^12,22−25^ Besides the evident pH implications, inhibition of the highly abundant CNS-expressed CAs (*i.e.*, II, VII, and XII) may be expected to induce a reduction of the bicarbonate-dependent depolarization of GABAA receptors when KCC2 is compromised in peripheral nerve injuries.^26,27^ Our interests in this field were also fostered by seminal contributions from some of us which demonstrated that CAs inhibitors (CAIs) synergistically enhanced the antitumor activity of platinum-based drugs.^28,29^

## Results and Discussion

### Design and Synthesis

**Figure 2:**
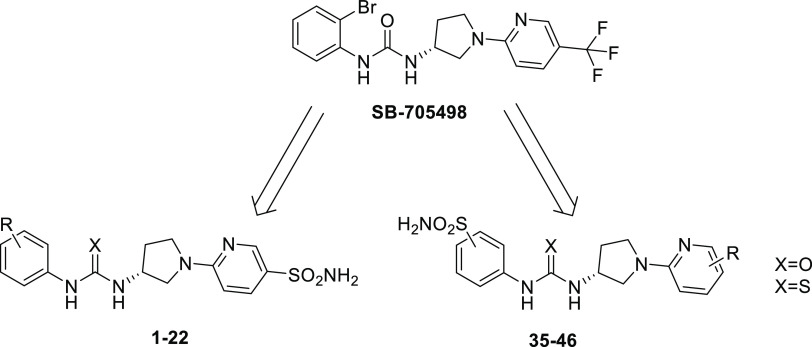
Design of TRPV1-CA derivatives reported in this study.

We sought to make use of the potent CNS-penetrant and selective TRPV1 antagonism of **SB-705498** with the aim to introduce within its chemical scaffold minimal functional groups necessary to endow the final products with activity against the hCAs of interest (Figure 2).^30^

Our synthetic strategy accounted for: (i) replacement of CF_3_ moiety on **SB-705498** with the prototypic pharmacophore for CA inhibition such as the primary sulfonamide; moreover, different substituents on phenyl ring were employed to discover the best interactions in both targets; (ii) replacement of the bromine atom within **SB-705498** with the same group either in meta or para position and, in addition, other groups instead CF_3_ moiety was investigated (*i.e.*, NO_2_ and H) (Figure 2). Finally, we investigated whether the stereocenter could affect the binding affinity against the different CA isoforms and TRPV1 receptor.

**Scheme 1:**
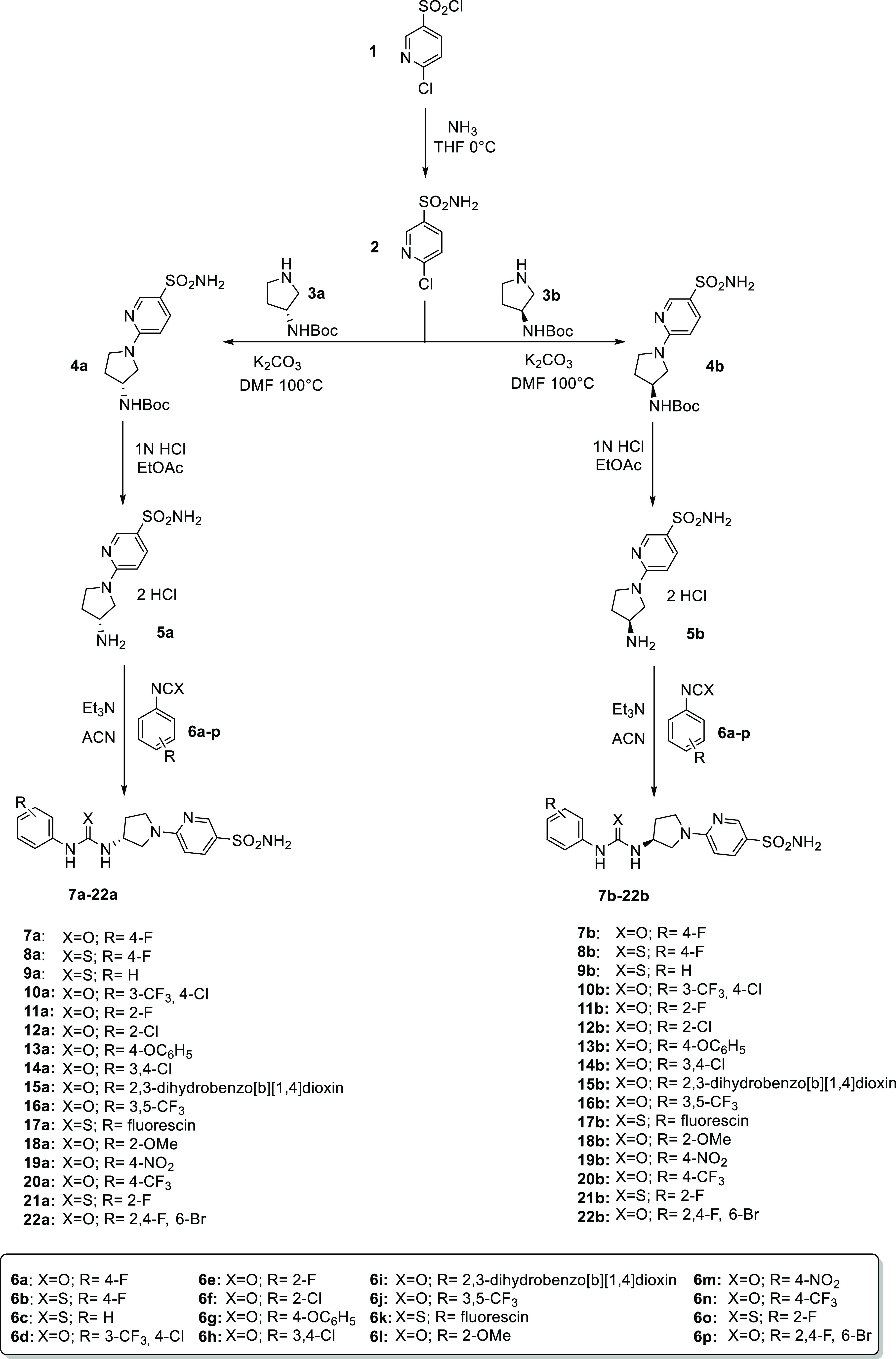
General Synthesis of Derivatives **7–22**

The first synthetic route was accomplished by preparing the intermediate **2** in a single-step procedure which involved the nucleophilic reaction between the commercially available sulfonyl chloride **1** and ammonia in tetrahydrofuran (THF) at 0 °C. The chloro pyrimidine derivative **2** was reacted with enantiopure-protected pyrrolidines (*R*)-**3a** and (*S*)-**3b** in *N*,*N*-dimethylformamide (DMF) at 100 °C with K_2_CO_3_ to afford **4a** and **4b**. Boc-deprotection by hydrolysis afforded **5a-b**, which were subjected to coupling reactions with available isocyanates and isothiocyanates **6a-p** to provide the sulfonamide containing urea and thiourea derivatives **7–22** (Scheme 1).

**Scheme 2:**
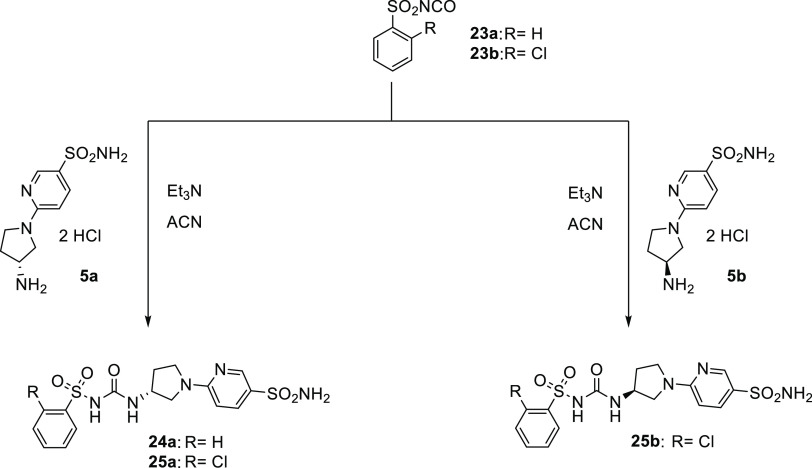
General Synthesis of Derivatives **24–25**

Moreover, we employed two aromatic sulfonyl isocyanates (**23a-b**) to obtain compounds **24** and **25** bearing the sulfonylureido moiety (Scheme 2).

**Scheme 3:**
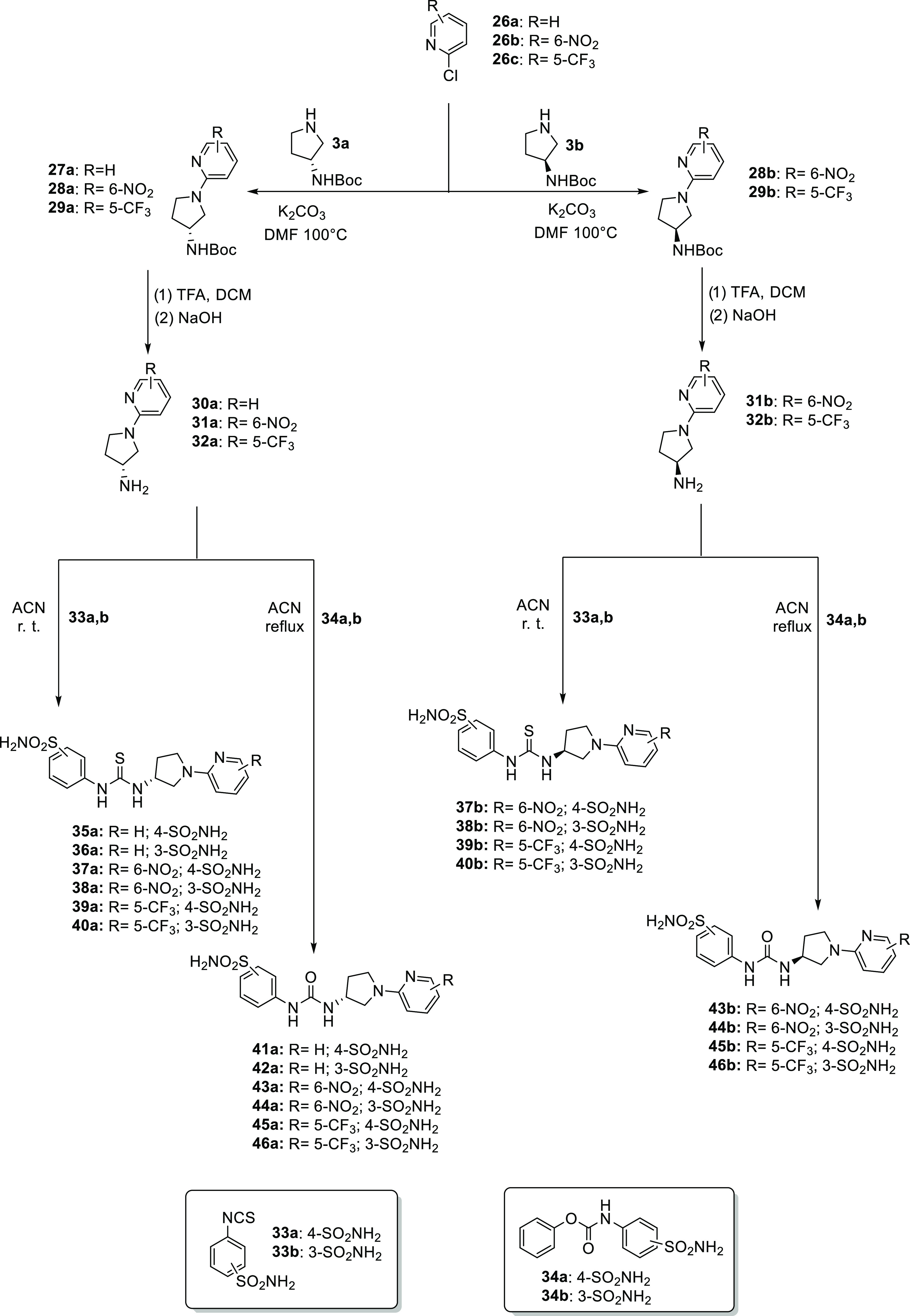
General Synthesis of Derivatives **41–46**

As for the second synthetic route, the chloropyridine **26a-c** are reacted with the enantiopure pyrrolidines **3a-b** using the same conditions previously reported for **4a-b**. Subsequently, standard Boc-deprotection was carried out with trifluoroacetic acid (TFA) followed by treatment with a 1 N aqueous solution of NaOH to obtain free amines **30–32**. Two different synthetic pathways were pursued for the synthesis of **35–46** (Scheme 3).

As reported above, first we used two different sulfonamide isothiocyanates (**33a-b**) to give the thioureido derivatives **35–40**. On the other hand, the ureido ones (*i.e.*, **41–46)** were obtained by means of carbamates **34a-b** (Scheme 3).

### Carbonic Anhydrase Inhibition

The inhibition profiles of all product synthesized (**7a,b–22a,b**, **24a, 25a-b**, **35a–46a, 37b–40b, 43b–46b**) against the physiologically relevant hCAs I, II, IV, VII, IX, and XII isoforms were investigated by the stopped-flow CO_2_ hydrase assay and compared to the reference CAI acetazolamide (**AAZ**)^31^ (Table 1).

**Table 1:** Inhibition Data of hCA Isoforms I, II, IV, VII, IX, and XII with Compounds **7a,b–22a,b**, **24a, 25a,b**, **35–46a, 37b–40b, 43b–46b**, and **AAZ** by a Stopped-Flow CO_2_ Hydrase Assay

*K*_I_ (nM)^a^						
cmp	hCA I	hCAII	hCAIV	hCAVII	hCAIX	hCAXII
**7a**	27.4	15.1	2656	6.6	32.2	59.3
**7b**	260.4	187.3	8242	12.1	20.2	50.3
**8a**	68.8	12.1	3115	8.6	30.8	39.5
**8b**	94.5	55.6	2695	2.6	27.3	35.4
**9a**	164.0	45.9	3732	8.5	25.5	59.3
**9b**	321.1	178.9	9478	11.6	54.1	23.4
**10a**	788.1	453.4	4019	78.6	737.0	67.6
**10b**	707.3	478.3	8516	15.6	77.5	7.6
**11a**	37.0	23.3	2516	187.3	280.9	73.0
**11b**	475.8	137.5	4458	11.6	20.1	43.9
**12a**	82.5	70.3	3002	73.5	316.2	83.6
**12b**	393.0	95.2	2031	0.9	22.9	41.7
**13a**	937.6	818.0	3079	76.9	245.0	406.6
**13b**	871.4	725.5	5354	52.0	89.8	89.9
**14a**	547.0	82.2	4903	26.5	1798	7.2
**14b**	959.0	555.6	2528	12.1	74.4	44.6
**15a**	72.2	50.2	3560	9.4	192.4	6.7
**15b**	544.8	400.3	5725	29.5	82.0	62.9
**16a**	903.3	714.9	5161	40.8	1438	37.9
**16b**	625.3	432.3	2495	8.3	54.9	9.4
**17a**	80.6	42.2	848.6	7.8	32.0	3.9
**17b**	91.5	42.9	5217	14.9	8.6	27.8
**18a**	73.5	287.4	5077	68.7	36.1	72.2
**18b**	476.0	309.6	7857	58.8	61.8	38.5
**19a**	252.3	436.8	1525	56.1	145.0	57.4
**19b**	800.4	525.0	5716	8.2	67.5	8.5
**20a**	154.2	409.2	4817	27.9	379.3	44.2
**20b**	72.4	35.3	2686	8.6	5.5	48.7
**21a**	31.3	14.9	3087	50.7	378.2	8.3
**21b**	68.7	31.3	843.4	2.6	7.2	41.0
**22a**	93.8	446.1	4883	41.1	35.8	8.1
**22b**	584.3	481.9	8195	14.0	48.3	8.2
**24a**	19.5	513.0	36.6	78.8	27.5	49.3
**25a**	72.3	15.4	704.5	30.1	9.2	279.7
**25b**	89.2	38.5	911.4	46.4	9.7	308.6
**35a**	48.0	6.9	774.5	9.7	9.6	9.0
**36a**	85.1	9.6	320.4	55.4	310.8	78.2
**37a**	74.8	6.7	471.3	174.7	16.8	408.6
**37b**	54.5	4.9	3203	7.7	1.3	8.8
**38a**	78.5	8.6	238.0	83.6	162.0	237.7
**38b**	8.7	3.9	1295	15.6	1.4	9.3
**39a**	90.8	18.9	4969	73.3	355.0	62.5
**39b**	93.2	11.5	9722	15.7	6.0	9.4
**40a**	490.3	20.4	805.9	55.1	36.4	29.4
**40b**	412.7	95.4	668.7	14.2	7.9	9.6
**41a**	58.3	8.0	3511	9.5	29.1	31.6
**42a**	9.6	1.7	2476	8.1	125.5	8.2
**43a**	68.3	7.5	2293	163.6	93.6	83.7
**43b**	76.2	14.9	8226	8.7	26.9	9.1
**44a**	76.0	6.1	2101	74.8	971.3	37.9
**44b**	87.2	6.6	9551	17.7	192.7	48.4
**45a**	72.4	27.4	3900	8.2	36.7	6.3
**45b**	68.6	46.3	9711	12.8	54.0	8.6
**46a**	198.6	8.4	4023	6.9	247.0	6.6
**46b**	305.5	8.9	8770	13.4	44.0	8.9
**AAZ**	250.0	12.1	74.0	2.5	25.7	5.7

a Mean from three different assays, by a stopped-flow technique (errors were in the range of ±5–10% of the reported values).

Taking into account the reported data, the structure–activity relationship (SAR) based on specific isoforms is drawn below:

- The cytosolic hCA II is inhibited by derivatives **7–22a,b** with *K*_i_’s spanning from low nanomolar range (12.1 nM **8a**) up to high nanomolar values (*i.e.*, *K*_i_ of 818 nM **13a**). The affinities for hCA I are similar, thus falling within comparable inhibition ranges. Also in this case, **13a** was the least effective (*K*_i_ of 937.6 nM for the hCA I). From the general point of view, the same kinetic trend for isoforms I and II were observed. Of note, all of the (*R*) enantiomers (*i.e.*, **7–22a**, **24a, 25a**, and **35–46a**) were more effective inhibitors compared to the (*S*) series comprising **7–22b**, **25b**, **37–40b**, and **43–46b**. Among them, compound **7a** was almost 10-fold more potent than its (*S*) enantiomer **7b** on both hCA isoforms. The derivative **11a** (*K*_i_ 37.0 nM) became 13-fold more active than **11b** (*K*_i_ 475.8 nM) against hCA I.
  As in the case of compounds **10a-b**, **16a-b**, and **20a**-**b**, an opposite inhibition trend for the two enantiomers was observed, being the (*S*)-**10b**, (*S*)-**16b**, and (*S*)-**20b** far more effective hCA I, II inhibitors compared to the (*R*) counterparts (*i.e.*, (*R*)-**10b**, (*R*)-**16b**, and (*R*)-**20b**). It is reasonable to speculate that such a reversal activity between (*R*) and (*S*) may be attributed to the CF_3_ group.^32^ On the other hand, the introduction of a bulky scaffold such as in compounds **13a-b** and **17a-b** flattened any discrimination between the enantiomeric series. As for the hCA II, the compound series bearing the sulfonamide moiety on the phenyl ring (**35–46**), it is interesting to note that the position of the sulfonamide in para or meta for thioureido derivatives **37–40a-b** induced an increase in the potency of the (*R*) enantiomers, as in the case of **40a**. On the other hand, ureido derivatives showed the same trend when the sulfonamide moiety was placed in para position (*i.e.*, **43a** and **45a**).
- The membrane isoform hCA IV was inhibited by almost all derivatives with *K*_I_ values in the micromolar range (Table 1). Of note, the replacement of the ureido moiety with sulfonylureido resulted in a drastic increase of the potency up to medium nanomolar values as for compound **24a** (*K*_i_ 36.6 nM). The addition of one chlorine atom in ortho of sulfonylureido moiety (derivative **25a-b**) decreased the potency about 20-fold.
- The brain-associated isoform hCA VII was strongly inhibited by almost all of the series reported with *K*_I_ inhibition values in the sub-nanomolar range (*i.e.*, **12b***K*_i_ 0.9 nM). For this isoform, we observed a different inhibition trend compared to hCA I and II, as most (*S*) enantiomers became far more effective compared to the (*R*) ones. An interesting feature was represented by the simple replacement of the ureido moiety with the thioureido instead (*i.e.*, **7a-b** and **8a-b**). In this case, an inversion of activity for the corresponding enantiomers was observed. Indeed, the ureido derivative **7a** (*K*_i_ 6.6 nM) showed 2-fold higher potency compared to the (*S*) enantiomer (**7b**, *K*_i_ 12.1 nM). In contrast, the thioureido derivative **8b** (*K*_i_ 2.6 nM) showed 3 times higher selectivity than the (*R*) enantiomer (**8a**, *K*_i_ 8.6 nM). A halogen atom in ortho position would appear to be essential for selectivity toward the (*S*) enantiomer, as shown by derivatives **11a-b** and **21a-b** with over 10-fold and 80-fold more selective than compounds **12a-b**. From the general point of view, the position of the sulfonamide group in **37–46** seems also to play an important role in the enantiomeric-dependent selectivity. For instance, within the meta regioisomeric series, the selectivity for (*R*) enantiomer over (*S*) increased.
- The tumor-associate isoforms hCA IX and hCA XII were effectively inhibited by all compounds herein reported and showed *K*_I_ values comprised between 1.3 and 971.3 nM (Table 1). In addition, a pivotal role for the enantiomeric-dependent selectivity for such isoforms was represented by the substituents placed on the phenyl rather than the pyridine ring. We observed for derivatives **9**, **14**, **15**, **17**, **18**, and **21** an inverted selectivity between the hCA IX and XII (Table 1). An interesting case for the hCA IX was observed among compounds **11a-b** and **21a-b** as replacement of the ureido group in the former with the thioureido in the latter resulting in increased (*S*) selectivity (**11b** was 13.9-fold more active than **11a**; **21b** was 52.5-fold more active than **21a**). On the other hand, for hCA XII, the inhibition selectivity shifted toward the enantiomer (*R*) such as for derivative **21a-b**. Compounds **37–46** observed the (*S*) enantiomers as the best inhibitors against both isoforms, especially for derivatives **38a-b** showing a selectivity of over 100-fold for the enantiomer (*S*) **38b**.

### TRPV1 Assay

The ability of the selected (*R*) enantiomers **7a**, **9a–16a**, **18a–22a**, **24a**, **35a–46a**, and the (*S*) counterparts **39b** and **45b** to modulate TRPV1 receptor activity was assessed, and the data are reported in Table 2.

**Table 2:** EC_50_ Values for **7a**, **9a–16a**, **18a–22a**, **24a**, **35a–46a**, and **39b** on TRPV1 Receptor Activity

cmp	EC_50_ (μM)	SD
**7a**	>100	
**9a**	>100	
**10a**	74.5	2.3
**11a**	>100	
**12a**	11.9	1.6
**13a**	>100	
**14a**	>100	
**15a**	>100	
**16a**	>100	
**18a**	>100	
**19a**	>100	
**20a**	>100	
**21a**	>100	
**22a**	>100	
**24a**	>100	
**35a**	>100	
**36a**	>100	
**37a**	8.0	1.7
**38a**	44.2	1.1
**39a**	12.4	1.4
**39b**	12.5	1.8
**40a**	21.9	1.0
**41a**	>100	
**42a**	>100	
**43a**	>100	
**44a**	>100	
**45a**	29.5	0.3
**45b**	3.1	1.6
**46a**	37.3	2.5

Although the compounds reported in this study were all derived from the TRPV1 antagonist **SB-705498**, the data obtained accounted for a clear agonism effect (Table 2). This is not surprising as it is well known that even small chemical modifications might lead to an agonism–antagonism switch in the modulation of TRPV1 activity.

For instance, **10a**, **37a**, **38a**, **39a-b**, **40a**, **45a-b**, and **46a** showed moderate agonism effects with EC_50_ values spanning between 3.1 and 74.5 μM (Table 2). In more detail, the sulfonamide group placed into the pyridine ring became deleterious as most of such derivatives were ineffective. A slight activity (*i.e.*, EC_50_ of 74.5 μM) was detected for **10a**, which was remarkably restored when the chlorine atom at position 2 was introduced (EC_50_ of 11.9 μM) as in compound **12a**. Conversely, most of the products bearing the sulfonamide moiety onto the phenyl ring showed activity with associated EC_50_ values in the low micromolar range such as **37a** and **45b** with 8.0 and 3.1 μM, respectively. Quite interestingly, the configuration of the stereocenter in some cases did not influence either the activity or the potency as clearly shown by the enantiomers **39a** and **39b**, which reported equal EC_50_ value of 12.5 μM. Isomeric-dependent discrimination in terms of potency was reported for (*R*)-**45a** and (*S*)**-45b** being the latter 9-fold more active than its counterpart **45a**.

### X-ray Crystal Structures

**Figure 3:**
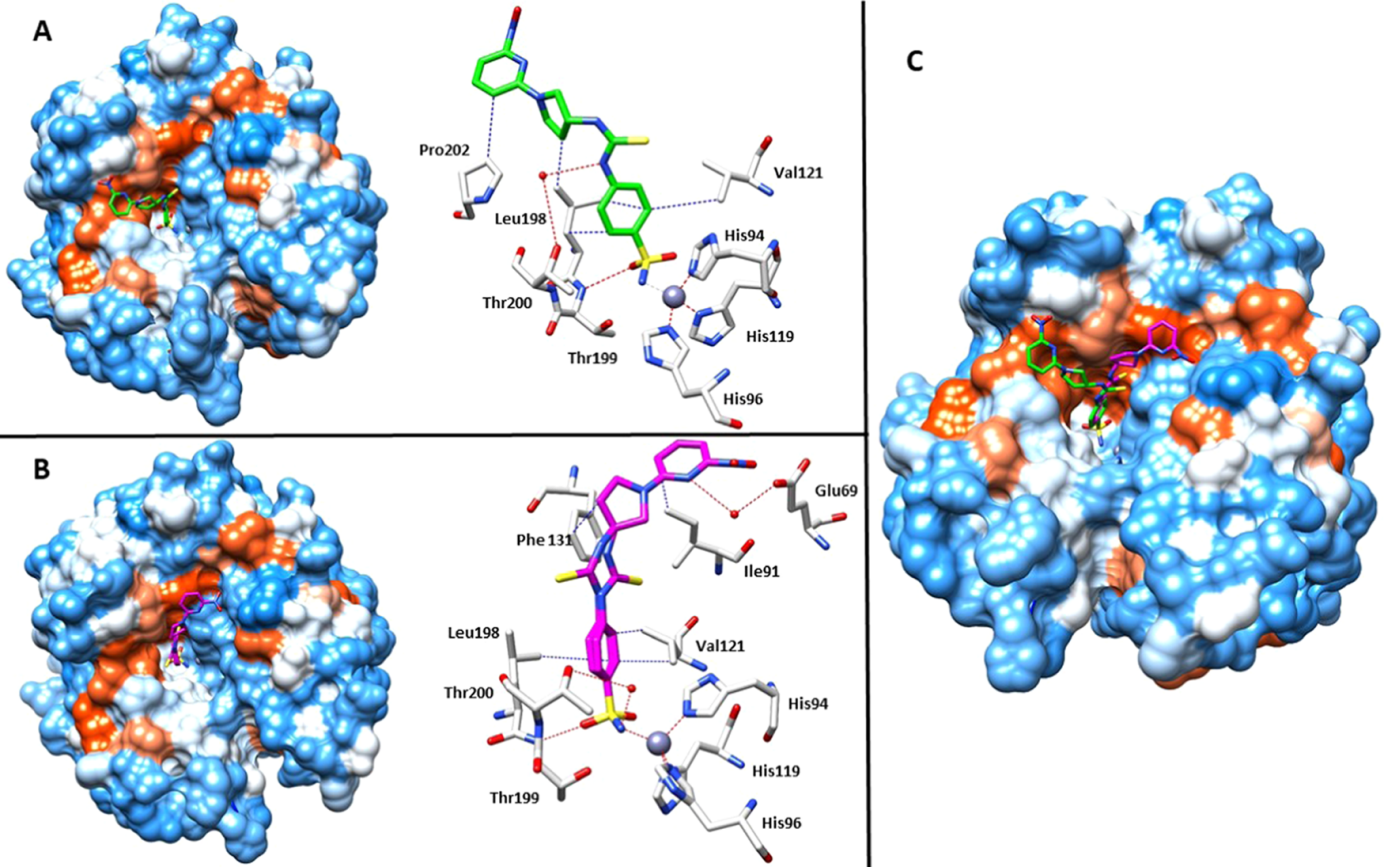
(A) X-ray crystal structures of hCA II bound with compound (*R*)-**37a** (green, PDB: 8BJX). (B) X-ray crystal structures of hCA II bound with compound (*S*)**-37b** (magenta, PDB: 8BOE). (C) Overlay of compounds (*R*)-**37a** and (*S*)**-37b** with hCA II. Residues involved in the binding of inhibitors are also shown; the gray sphere represents the zinc ion in the active site of the proteins.

To clarify the molecular basis of CA inhibition by our derivatives, we determined the X-ray structures of hCA II in complex with the enantiomers (*R*)-**37a** and (*S*)**-37b** at 1.3 and 1.6 Å resolution, respectively (Figure 3).

Analysis of the electron density maps (Figure S1 in the Supporting Information (SI)) showed for the inhibitor (*R*)-**37a** a density, into the catalytic cleft, fully compatible with our ligand. As expected, the sulfonamide moiety interacted directly with the zinc ion and a hydrogen bond with the residue of Thr199, thus showing the typical binding mode of this class of inhibitors.^33^ Furthermore, typical hydrophobic interactions between the benzenesulfonamide moiety and side chains of Val121 and Leu198 were established and contributed to strengthen the complex within the active site. The proximal nitrogen atom of (*R*)-**37a** thioureido moiety was engaged in a water bridge with Thr200. Valuable additional hydrophobic interactions were observed between Leu198 and Pro202 and the hydrophobic sections of the main scaffold which were responsible for sticking the entire ligand within the hydrophobic region of the active site (Figure 3A).

Interesting structural features were also revealed for the second inhibitor (*S*)**-37b** bound within the hCA II (Figure 3B). First, the thioureido moiety was observed in double conformation. Moreover, interesting features were also observed for the tail of derivative (*S*)**-37b**. Indeed, the (*S*) stereocenter of pyrrolidine ring moved this moiety on the other side of Phe131 engaging a hydrophobic interaction with this residue. This different location of the tail of (*S*)**-37b** is also stabilized by a water bridge between the nitrogen of pyridine ring with Glu69 and the hydrophobic interaction with Ile91. The structural comparison (Figure 3C) among the two enantiomers (*R*)-**37a** and (*S*)**-37b** revealed also similar features, such as the typical benzenesulfonamide interactions with the catalytic zinc atom and Thr199; on the other hand, the stereocenter is able to influence the tail conformations of the two molecules which occupy two different hydrophobic pockets divided by Phe131 residue. Nevertheless, this structural diversity does not significantly affect the grade of inhibition of the two inhibitors for this isoform.

### *In Vivo* Pain-Relieving Effect

**Figure 4:**
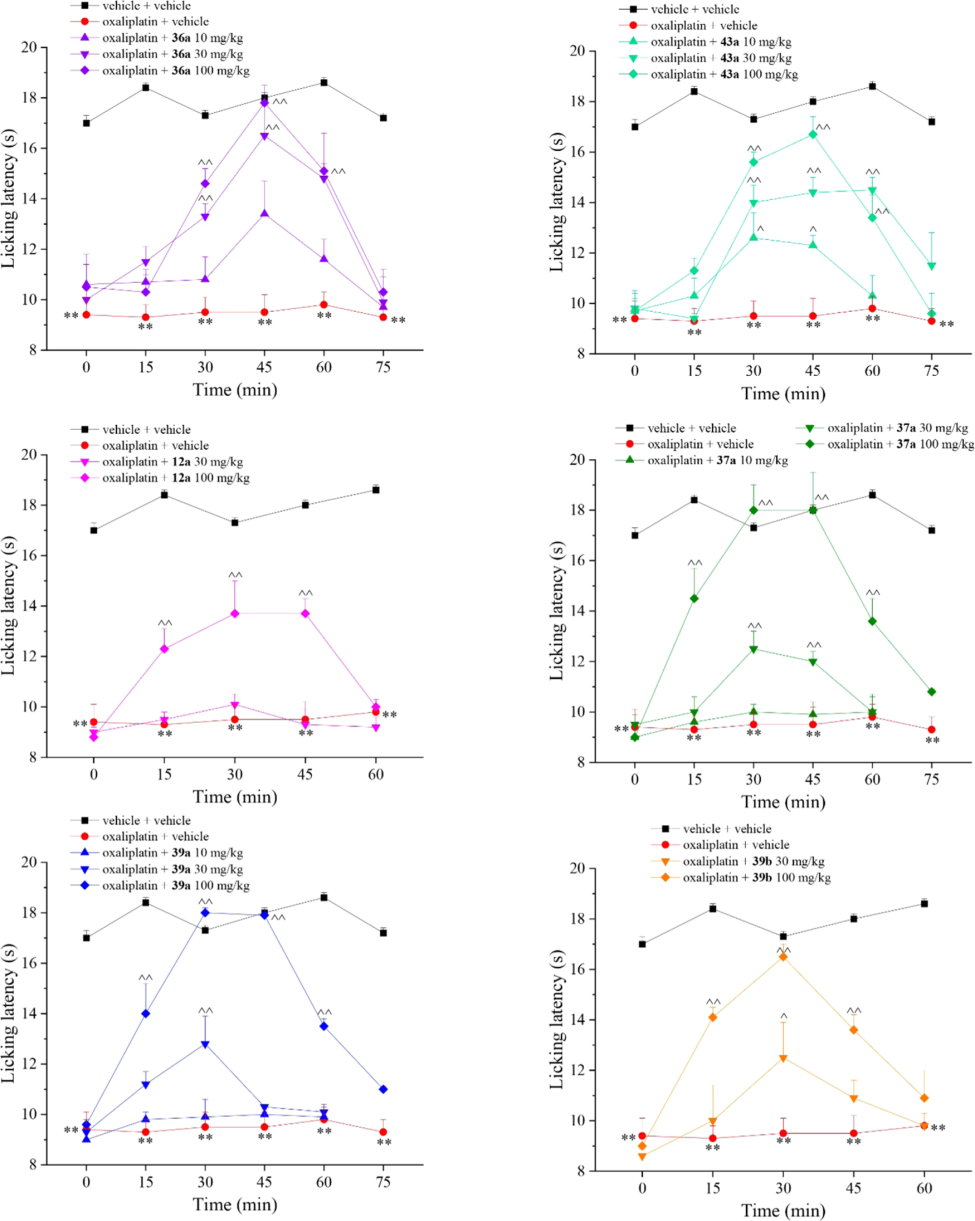
Pain-relieving effect of acute administration of derivatives **12a**, **36a**, **37a**, **39a**, **39b**, and **43a** in a mouse model of oxaliplatin-induced neuropathic pain. Sensitivity to a non-noxious thermal stimulus was assessed by the Cold plate test. Oxaliplatin (2.4 mg/kg, i.p.) was injected on days 1–2, 5–9, and 12–14 (10 injections). On day 15, compounds were acutely per os administered in a range dose of 10–100 mg/kg. Assessment of cold allodynia was performed before and 15, 30, 45, 60, and 75 min after treatments. Results are expressed as the mean ± standard error of the mean (S.E.M.) of 10 mice analyzed in two different experimental sets. **P < 0.01 vs vehicle + vehicle; ^∧^*P* < 0.05 and ^∧∧^*P* < 0.01 vs oxaliplatin + vehicle-treated animals. Each value represents the mean ± S.E.M. of 10 mice performed in two different experimental sets.

Based on *in vitro* obtained CA and TRPV1 profiles, we selected the most appropriate compounds to subject to an *in vivo* mouse model of neuropathic pain induced by oxaliplatin repeated treatment.^34,35^ For instance, we considered derivatives: (i) (*R*)-**36a** and (*R*)-**43a** as potent CAs inhibitors devoid of TRPV1 activity; (ii) (*R*)-**12a** and (*R*)-**37a**, which are effective on both targets; and (iii) the two enantiomers (*R*)-**39a** and (*S*)-**39b**, which showed close effectiveness on CA II and TRPV1. The results are highlighted in Figure 4.

In our experimental conditions, we evaluated the animal licking latency after oral administration of the selected compounds at increasing concentrations up to 100 mg/kg. Overall, we observed dose-dependent correlations with various outcomes as below reported:

(1) Compounds (*R*)-**36a** and (*R*)-**43a** devoid of any activity on TRPV1 showed a dose-dependent effectiveness peaking at 45 min post-administration, followed by a rapid decrease of the effect which was suppressed at 75 min (Figure 4).
(2) (*R*)-**12a** and (*R*)-**37a** peaked at 30 min post-administration, and were effective up to 45 min. (*R*)-**37a** was more potent and effective than (*R*)-**12a** (Figure 4). Such an effect may be reasonably attributed to the major efficacy of (*R*)-**37a** in inhibiting the CA II over (*R*)-**12a** (*i.e.*, 10.4-fold) also in consideration DRG neurons are particularly rich in such an isoform.^12^
(3) Quite interestingly (*R*)-**39a** and (*S*)-**39b** were significantly effective at 30 and 100 mg/kg, completely reverting oxaliplatin hypersensitive at the higher dose. Since the *in vitro* activity on CA II and TRPV1 were close matching (Tables 1 and 2), the slightly better profile of (*S*)-**39a** may be ascribed to differentiated metabolic processes which take place on each enantiomer after oral administration (Figure 4).

## Conclusions

To the best of our knowledge, this is the first report on dual-targeting molecules able to relieve OINPs by simultaneous activation of TRPV1 and inhibition of CA enzymes. Preliminary SARs were performed by *in vitro* evaluation of the effects on both targets when substitutions of aromatic rings, bio-isosteric switch between ureido and thioureido linkers, as well as the introduction of stereocenters were operated. Overall, (*R*)- or (*S*)-stereocenters present within the set of compounds synthesized did not seem to have relevant effects on the activity of both targets. Particularly striking was the case of (*R*)-**37a** and (*S*)**-37b** (*i.e.*, CA II *K*_I_’s of 6.7 and 4.9 nM, respectively) as the X-ray structures of their adducts with CA II showed the molecular tails lying onto the enzymatic hydrophobic section of the active site and occupying distinct subpockets split apart by the Phe131 residue.

Our approach to introduce the CA warhead sulfonamide moiety into the TRPV1 antagonist modulator **SB-705498** resulted in the reversal of activity up to moderate agonism. The observed *in vitro* effects of molecular stereocenters on TRPV1 were various. For instance, (*R*)-**39a** and (*S*)-**39b** (*i.e.*, EC_50_ of 12.5 μM for both compounds) did not induce any potency change, whereas for the derivatives **45**, the (*S*)-enantiomer was 9-fold more effective than its corresponding (*R*)-counterpart (*i.e.*, ECs_50_ of 29.5 and 3.1 μM for (*R*)-**45** and (*S*)-**45**, respectively).

A selection among the most valuable *in vitro* performing compounds (*i.e.*, (*R*)-**12a**, (*R*)-**36a**, (*R*)-**37a**, (*R*)-**39a**, (*S*)-**39b**, and (*R*)-**43a**) allowed us to explore their effects on an *in vivo* mouse model of OINP. All derivatives endowed with activity either on CA II or TRPV1 induced long-lasting pain-relieving effects with maximum efficacy at 30 min after administration. Conversely, compounds (*R*)-**36a** and (*R*)-**43a** endowed only with activity against the CAs reported moderate and shorter relieving outcomes, thus demonstrating the important contribution to the biological model ascribed to the TRPV1 agonist section of the molecules reported. Quite interestingly, the enantiomers (*R*)-**39a** and (*S*)-**39b** became significantly dissimilar in inducing a biochemical response in our *in vivo* model, with the former being far more effective and lasting compared to its (*S*)-counterpart.

Although this study is not exhaustive in defining the kinetic as well as biochemical features of the entire set of molecules reported to manage OINPs, it gives solid pieces of evidence that small-size molecules acting simultaneously as mild TRPV1 agonists and potent inhibitors of the CAs represent a valid and worth developing strategy useful to minimize OINP-induced symptoms such as pain.

## Experimental Section

### General

Anhydrous solvents and all reagents were purchased from Sigma-Aldrich, VWR, and TCI. All reactions involving air- or moisture-sensitive compounds were performed under a nitrogen atmosphere. Nuclear magnetic resonance (^1^H NMR, ^13^C NMR, ^19^F NMR) spectra were recorded using a Bruker Advance III 400 MHz spectrometer in DMSO-*d*_6_. Chemical shifts are reported in parts per million (ppm), and the coupling constants (*J*) are expressed in Hertz (Hz). Splitting patterns are designated as follows: s, singlet; d, doublet; t, triplet; m, multiplet; brs, broad singlet; dd, double of doubles. The assignment of exchangeable protons (NH) was confirmed by the addition of D_2_O. Analytical thin-layer chromatography (TLC) was carried out on Merck silica gel F-254 plates. Flash chromatography purifications were performed on Merck silica gel 60 (230–400 mesh ASTM) as the stationary phase, and ethyl acetate, *n*-hexane, acetonitrile, and methanol were used as eluents. The solvents used in MS measurements were acetone, acetonitrile (Chromasolv grade), purchased from Sigma-Aldrich (Milan, Italy), and Milli-Q water 18 MΩ, obtained from Millipore’s Simplicity system (Milan, Italy). The mass spectra were obtained using a Varian 1200L triple quadrupole system (Palo Alto, CA) equipped with electrospray source (ESI) operating in both positive and negative ions. Stock solutions of analytes were prepared in acetone at 1.0 mg mL^–1^ and stored at 4 °C. Working solutions of each analyte were freshly prepared by diluting stock solutions in a mixture of Milli-Q H_2_O/ACN 1/1 (v/v) up to a concentration of 1.0 μg mL^–1^. The mass spectra of each analyte were acquired by introducing, via a syringe pump at 10/L min^–1^, the working solution. Raw data were collected and processed by Varian Workstation, version 6.8, software. All compounds reported here are >95% of purity by NMR.

#### Synthesis of 6-Chloropyridine-3-sulfonamide (**2**)

6-Chloropyridine-3-sulfonyl chloride **1** (1 g) was dissolved at 0 °C in THF and was added to ammonia solution (28%, 4 mL). The solution was stirred at 0 °C for 4 h, quenched with H_2_O, extracted with EtOAc, and dried over Na_2_SO_4_ to afford compound **2** as a white solid, yield 83%. ^**1**^**H NMR** (400 MHz, DMSO-*d*_6_) δ(ppm): 8.84 (1H, d, *J* = 2.21 Hz), 8.26 (1H, dd, *J* = 8.36, 2.25 Hz), 7.81 (1H, d, *J* = 8.39 Hz), 7.75 (2H, bs); ^**13**^**C NMR** (100 MHz, DMSO-*d*_6_) δ(ppm): 153.9, 147.9, 140.6, 138.2, 125.8; MS (ESI positive) *m*/*z*: 192.9 [M + H]^+^.

### General Synthesis of Compounds **4a-b**

To a solution of 6-chloropyridine-3-sulfonamide (**2**, 1 equiv) and K_2_CO_3_ (1.3 equiv) in dry DMF under inert atmosphere (N_2_) was added the appropriate pyrrolidine (**3a,b**, 1 equiv). The mixture was stirred for 4 h at 100 °C. The reaction mixture was quenched with ice-cooled, saturated NH_4_Cl solution and stirred for 15 min to give a precipitate, which was collected by vacuum filtration and washed with water. The obtained solid was triturated with Et_2_O to yield the derivatives **4a,b**.

#### *tert*-Butyl (*R*)-(1-(5-Sulfamoylpyridin-2-yl)pyrrolidin-3-yl)carbamate (**4a**)

Following the general procedure, the product was a white solid **4a**, yield 80%. ^**1**^**H NMR** (400 MHz, DMSO-*d*_6_) δ(ppm): 8.46 (1H, d, *J* = 2.17 Hz), 7.83 (1H, dd, *J* = 8.93, 2.25 Hz), 7.26 (1H, d, *J* = 5.21 Hz), 7.17 (2H, bs), 6.57 (1H, d, *J* = 9.00 Hz), 4.15 (1H, m), 3.68 (1H, m), 3.66 (1H, m), 3.58 (1H, m), 3.32 (1H, m), 2.17 (1H, m), 1.94 (1H, m), 1.43 (9H, s); ^**13**^**C NMR** (100 MHz, DMSO-*d*_6_) δ(ppm): 158.5, 156.2, 147.2, 135.6, 128.1, 106.6, 78.8, 53.1, 50.7, 45.8, 31.4, 29.1; MS (ESI positive) *m*/*z*: 343.1 [M + H]^+^.

#### *tert*-Butyl (*S*)-(1-(5-Sulfamoylpyridin-2-yl)pyrrolidin-3-yl)carbamate (**4b**)

Following the general procedure, the product was a white solid **4b**, yield 82%. ^**1**^**H NMR** (400 MHz, DMSO-*d*_6_) δ(ppm): 8.45 (1H, d, *J* = 2.21 Hz), 7.82 (1H, d, *J* = 8.97, 2.46 Hz), 7.28 (1H, d, *J* = 6.04 Hz), 7.18 (2H, bs), 6.57 (1H, d, *J* = 9.01 Hz), 4.16 (1H, m), 3.70–3.67 (1H, m), 3.58 (1H, m), 3.48 (1H, m), 3.31 (1H, m), 2.18–2.15 (1H, m), 1.95–1.92 (1H, m), 1.43 (9H, s); ^**13**^**C NMR** (100 MHz, DMSO-*d*_6_) δ(ppm): 158.6, 156.2, 147.2, 135.5, 128.1, 106.6, 78.8, 53.1, 50.7, 45.8, 31.4, 29.1; MS (ESI positive) *m*/*z*: 343.1 [M + H]^+^.

### General Synthesis of Compounds **5a-b**

To a solution of 1 N HCl in EtOAc was added *tert*-butyl (1-(5-sulfamoylpyridin-2-yl)pyrrolidin-3-yl)carbamate **4a** or **4b**, and the mixture was stirred overnight at room temperature. Subsequently, the solvent was removed under vacuum to obtain the HCl salt of derivatives **5a,b**.

#### (*R*)-6-(3-Aminopyrrolidin-1-yl)pyridine-3-sulfonamide (**5a**)

Following the general procedure, the product was a light yellow solid **5a**, yield 99%. ^**1**^**H NMR** (400 MHz, DMSO-*d*_6_) δ(ppm): 8.49 (1H, d, *J* = 1.82 Hz), 8.28 (3H, bs), 7.88 (1H, dd, *J* = 8.93, 1.37 Hz), 7.23 (2H, bs), 6.65 (1H, d, *J* = 8.84 Hz), 4.00 (1H, m), 3.79–3.74 (1H, m), 3.66–3.57 (3H, m), 2.42–2.35 (2H, m); ^**13**^**C NMR** (100 MHz, DMSO-*d*_6_) δ(ppm): 158.6, 147.3, 135.5, 127.9, 106.5, 55.3, 51.3, 46.0, 34.1; MS (ESI positive) *m*/*z*: 243.1 [M + H]^+^.

#### (*S*)-6-(3-Aminopyrrolidin-1-yl)pyridine-3-sulfonamide (**5b**)

Following the general procedure, the product was a light yellow solid **5b**, yield 97%. ^**1**^**H NMR** (400 MHz, DMSO-*d*_6_) δ(ppm): 8.63 (3H, m), 8.42 (1H, d, *J* = 1.81 Hz), 8.03 (1H, dd, *J* = 9.08, 1.69 Hz), 7.49 (2H, bs), 6.92 (1H, d, *J* = 9.12 Hz), 4.02 (1H, m), 3.88–3.76 (3H, m), 3.67 (1H, m), 2.41–2.37 (1H, m), 2.29–2.25 (1H, m); ^**13**^**C NMR** (100 MHz, DMSO-*d*_6_) δ(ppm): 154.2, 141.0, 138.2, 129.3, 111.8, 52.7, 50.3, 47.1, 29.8; MS (ESI positive) *m*/*z*: 243.1 [M + H]^+^.

### General Synthesis of Compounds **7–22**

Compound **5a/5b** (1 equiv) in acetonitrile was added to isocyanate or isothiocyanate **6a-p** (1 equiv) and Et_3_N (3 equiv). The solution was stirred overnight at room temperature. The reaction was quenched with saturated solution of NH_4_Cl, extracted with EtOAc, and dried over Na_2_SO_4_. The crude material was purified by flash column chromatography (MeOH/DCM: 5:95), to yield compounds **7–22**.

#### (*R*)-6-(3-(3-(4-Fluorophenyl)ureido)pyrrolidin-1-yl)pyridine-3-sulfonamide (**7a**)

Following the general procedure, the product was a white solid **7a**, yield 78%. ^**1**^**H NMR** (400 MHz, DMSO-*d*_6_) δ(ppm): 8.48 (1H, bs), 8.39 (1H, bs), 7.85 (1H, d, *J* = 8.95 Hz), 7.42 (2H, m), 7.18 (2H, bs), 7.11–7.07 (2H, m), 6.62 (1H, d, *J* = 8.66 Hz), 6.55 (1H, d, *J* = 6.50 Hz), 4.36 (1H, m), 3.74–3.70 (1H, m), 3.58 (2H, m), 3.41 (1H, m), 2.28–2.23 (1H, m), 2.00–1.96 (1H, m); ^**13**^**C NMR** (100 MHz, DMSO-*d*_6_) δ(ppm): 158.6, 157.9 (d, *J* = 237.52 Hz), 155.9, 147.2, 137.5, 135.6, 128.4, 120.2 (d, *J* = 7.15 Hz), 116.0 (d, *J* = 22.09 Hz), 106.7, 53.5, 50.0, 45.8, 31.9; ^**19**^**F NMR** (376 MHz, DMSO-*d*_6_) δ(ppm): −122.4; MS (ESI positive) *m*/*z*: 380.1 [M + H]^+^.

#### (*S*)-6-(3-(3-(4-Fluorophenyl)ureido)pyrrolidin-1-yl)pyridine-3-sulfonamide (**7b**)

Following the general procedure, the product was a white solid **7b**, yield 47%. ^**1**^**H NMR** (400 MHz, DMSO-*d*_6_) δ(ppm): 8.48 (1H, bs), 8.39 (1H, bs), 7.85 (1H, d, *J* = 8.56 Hz), 7.42 (2H, m), 7.18 (2H, bs), 7.09 (2H, t, *J* = 8.61 Hz), 6.62 (1H, d, *J* = 8.93 Hz), 6.55 (1H, d, *J* = 6.35 Hz), 4.36 (1H, m), 3.74–3.70 (1H, m), 3.58 (2H, m), 3.41 (1H, m), 2.28–2.23 (1H, m), 2.03–1.98 (1H, m); ^**13**^**C NMR** (100 MHz, DMSO-*d*_6_) δ(ppm): 158.6, 157.9 (d, *J* = 237.00 Hz), 155.9, 147.2, 137.5, 135.6, 128.4, 120.2 (d, *J* = 7.48 Hz), 116.0 (d, *J* = 22.11 Hz), 106.7, 53.5, 50.0, 45.8, 31.9; MS (ESI positive) *m*/*z*: 380.1 [M + H]^+^.

#### (*R*)-6-(3-(3-(4-Fluorophenyl)thioureido)pyrrolidin-1-yl)pyridine-3-sulfonamide (**8a**)

Following the general procedure, the product was a white solid **8a**, yield 56%. ^**1**^**H NMR** (400 MHz, DMSO-*d*_6_) δ(ppm): 9.45 (1H, bs), 8.49 (1H, d, *J* = 1.96 Hz), 8.14 (1H, bs); 7.85 (1H, dd, *J* = 8.97, 2.07 Hz), 7.48 (2H, dd, *J* = 8.69, 4.95 Hz), 7.17 (3H, m), 6.63 (1H, d, *J* = 8.93 Hz), 4.90 (1H, m), 3.84–3.80 (1H, m), 3.60–3.49 (3H, m), 2.36–2.31 (1H, m), 2.16–2.11 (1H, m); ^**13**^**C NMR** (100 MHz, DMSO-*d*_6_) δ(ppm): 181.8, 158.6, 152.0 (d, *J* = 192.6 Hz), 147.2, 136.7, 135.7, 128.3, 126.3 (d, *J* = 13.3 Hz), 115.9 (d, *J* = 22.57 Hz), 106.7, 54.2, 52.9, 45.9, 31.3; ^**19**^**F NMR** (376 MHz, DMSO-*d*_6_) δ(ppm): −118.5; MS (ESI positive) *m*/*z*: 396.1 [M + H]^+^.

#### (*S*)-6-(3-(3-(4-Fluorophenyl)thioureido)pyrrolidin-1-yl)pyridine-3-sulfonamide (**8b**)

Following the general procedure, the product was a white solid **8b**, yield 46%. ^**1**^**H NMR** (400 MHz, DMSO-*d*_6_) δ(ppm): 9.49 (1H, bs), 8.48 (1H, s), 7.87 (1H, bs); 7.86 (1H, dd, *J* = 8.90, 2.13 Hz), 7.48–7.47 (2H, m), 7.18–7.15 (4H, m), 6.63 (1H, d, *J* = 8.99 Hz), 4.91 (1H, m), 3.83–3.80 (1H, m), 3.61–3.59 (2H, m), 3.53–3.50 (1H, m), 2.36–2.32 (1H, m), 2.16–2.10 (1H, m); ^**13**^**C NMR** (100 MHz, DMSO-*d*_6_) δ(ppm): 181.8, 158.5, 151.3 (d, *J* = 265.18 Hz), 147.1, 136.7, 135.7, 128.3, 126.4 (d, *J* = 7.21 Hz), 115.9 (d, *J* = 22.43 Hz), 106.8, 54.2, 52.9, 45.9, 31.2; MS (ESI positive) *m*/*z*: 396.1 [M + H]^+^.

#### (*R*)-6-(3-(3-Phenylthioureido)pyrrolidin-1-yl)pyridine-3-sulfonamide (**9a**)

Following the general procedure, the product was a white solid **9a**, yield 77%. ^**1**^**H NMR** (400 MHz, DMSO-*d*_6_) δ(ppm): 9.47 (1H, bs), 8.48 (1H, s), 8.13 (1H, s), 7.86 (1H, d, *J* = 8.56 Hz), 7.50 (2H, d, *J* = 7.57 Hz), 7.34 (2H, t, *J* = 7.37 Hz), 7.36 (2H, bs), 7.34–7.19 (1H, m), 6.63 (1H, d, *J* = 8.96 Hz), 4.92 (1H, m), 3.82 (1H, m), 3.60–3.53 (3H, m), 2.33 (1H, m), 2.13 (1H, m); ^**13**^**C NMR** (100 MHz, DMSO-*d*_6_) δ(ppm): 181.4, 158.6, 147.3, 140.4, 135.7, 129.4, 128.3, 125.0, 123.8, 106.8, 54.2, 52.9, 45.9, 31.3; MS (ESI positive) *m*/*z*: 378.1 [M + H]^+^.

#### (*S*)-6-(3-(3-Phenylthioureido)pyrrolidin-1-yl)pyridine-3-sulfonamide (**9b**)

Following the general procedure, the product was a white solid **9b**, yield 46%. ^**1**^**H NMR** (400 MHz, DMSO-*d*_6_) δ(ppm): 9.47 (1H, bs), 8.48 (1H, s), 8.12 (1H, d, *J* = 5.41 Hz); 7.85 (1H, m), 7.50 (2H, d, *J* = 7.78 Hz), 7.34 (2H, t, *J* = 7.48 Hz), 7.18 (2H, bs), 7.12 (1H, t, *J* = 7.20 Hz), 6.63 (1H, d, *J* = 8.96 Hz), 4.92 (1H, m), 3.85–3.81 (1H, m), 3.61–3.51 (3H, m), 2.37–2.32 (1H, m), 2.15 (1H, m); ^**13**^**C NMR** (100 MHz, DMSO-*d*_6_) δ(ppm): 181.4, 158.6, 147.2, 140.4, 135.7, 129.3, 128.3, 124.9, 123.7, 106.7, 54.2, 52.9, 45.9, 31.2; MS (ESI positive) *m*/*z*: 378.1 [M + H]^+^.

#### (*R*)-6-(3-(3-(4-Chloro-3-(trifluoromethyl)phenyl)ureido)pyrrolidin-1-yl)pyridine-3-sulfonamide (**10a**)

Following the general procedure, the product was a white solid **10a**, yield 62%. ^**1**^**H NMR** (400 MHz, DMSO-*d*_6_) δ(ppm): 8.89 (1H, bs), 8.48 (1H, s), 8.11 (1H, s), 7.85 (1H, d, *J* = 8.81 Hz), 7.58 (2H, s), 7.19 (2H, bs), 6.80 (1H, d, *J* = 5.96 Hz), 6.62 (1H, d, *J* = 8.91 Hz), 4.37 (1H, m), 3.75–3.71 (1H, m), 3.57 (2H, m), 3.39 (1H, m), 2.27–2.24 (1H, m), 2.02–2.01 (1H, m); ^**13**^**C NMR** (100 MHz, DMSO-*d*_6_) δ(ppm): 158.6, 155.5, 147.2, 140.8, 135.6, 132.7, 128.3, 127.4, 125.1, 123.4, 122.5, 117.1, 106.7, 53.4, 50.1, 45.8, 31.7; ^**19**^**F NMR** (376 MHz, DMSO-*d*_6_) δ(ppm): −61.4; MS (ESI positive) *m*/*z*: 464.1 [M + H]^+^; [α]_D_^22°^ = −10 (*c* = 2.7; Acetone).

#### (*R*)-6-(3-(3-(4-Chloro-3-(trifluoromethyl)phenyl)ureido)pyrrolidin-1-yl)pyridine-3-sulfonamide (**10b**)

Following the general procedure, the product was a white solid **10b**, yield 55%. ^**1**^**H NMR** (400 MHz, DMSO-*d*_6_) δ(ppm): 8.87 (1H, bs), 8.48 (1H, d, *J* = 1.82 Hz), 8.11 (1H, s), 7.85 (1H, dd, *J* = 8.91, 2.06 Hz), 7.58 (2H, s), 7.18 (2H, bs), 6.79 (1H, d, *J* = 6.26 Hz), 6.62 (1H, d, *J* = 8.99 Hz), 4.37 (1H, m), 3.76–3.72 (1H, m), 3.59–3.57 (2H, m), 3.45–3.41 (1H, m), 2.29–2.24 (1H, m), 2.04–1.99 (1H, m); ^**13**^**C NMR** (100 MHz, DMSO-*d*_6_) δ(ppm): 158.6, 155.5, 147.2, 140.8, 135.6, 132.8, 128.3, 125.1, 123.4, 122.5, 117.1 (q, *J* = 5.39 Hz), 106.7, 53.4, 50.1, 45.8, 31.7;MS (ESI positive) *m*/*z*: 464.1 [M + H]^+^; [α]_D_^22°^ = +11 (*c* = 3.1; Acetone).

#### (*R*)-6-(3-(3-(2-Fluorophenyl)ureido)pyrrolidin-1-yl)pyridine-3-sulfonamide (**11a**)

Following the general procedure, the product was a white solid **11a**, yield 52%. ^**1**^**H NMR** (400 MHz, DMSO-*d*_6_) δ(ppm): 8.49 (1H, bs), 8.20–8.15 (2H, m), 7.85 (1H, dd, *J* = 8.76, 1.50 Hz), 7.20–7.18 (3H, m), 7.12 (1H, t, *J* = 7.70 Hz), 7.04 (1H, d, *J* = 6.57 Hz), 6.96 (1H, dd, *J* = 12.46, 6.57 Hz), 6.64 (1H, d, *J* = 8.97 Hz), 4.37 (1H, m), 3.74–3.70 (1H, m), 3.59 (2H, m), 3.43 (1H, m), 2.29–2.15 (1H, m), 2.00–1.97 (1H, m); ^**13**^**C NMR** (100 MHz, DMSO-*d*_6_) δ(ppm): 158.6, 155.3, 152.4 (d, *J* = 240.88 Hz), 147.2, 135.6, 129.0 (d, *J* = 10.17 Hz), 125.2 (d, *J* = 3.30 Hz), 122.5 (d, *J* = 7.55 Hz), 115.6 (d, *J* = 18.97 Hz), 106.7, 53.6, 50.0, 45.7, 31.9; ^**19**^**F NMR** (376 MHz, DMSO-*d*_6_) δ(ppm): −130.9; MS (ESI positive) *m*/*z*: 380.1 [M + H]^+^; [α]_D_^22°^ = −9 (*c* = 3.1; Acetone).

#### (*S*)-6-(3-(3-(2-Fluorophenyl)ureido)pyrrolidin-1-yl)pyridine-3-sulfonamide (**11b**)

Following the general procedure, the product was a white solid **11b**, yield 57%. ^**1**^**H NMR** (400 MHz, DMSO-*d*_6_) δ(ppm): 8.48 (1H, d, *J* = 2.24 Hz), 8.22 (1H, d, *J* = 1.84 Hz), 8.17 (1H, t, *J* = 7.76 Hz), 7.86 (1H, dd, *J* = 8.94, 2.39 Hz), 7.21–7.19 (3H, m), 7.12 (1H, t, *J* = 7.54 Hz), 7.06 (1H, d, *J* = 6.66 Hz), 7.00 (1H, m), 6.65 (1H, d, *J* = 9.00 Hz), 4.37 (1H, m), 3.75–3.71 (1H, m), 3.59 (2H, m), 3.43–3.41 (1H, m), 2.29–2.23 (1H, m), 2.00–1.95 (1H, m); ^**13**^**C NMR** (100 MHz, DMSO-*d*_6_) δ(ppm): 158.5, 155.3, 152.5 (d, *J* = 240.66 Hz), 147.0, 135.7, 129.0 (d, *J* = 10.21 Hz), 125.2, 122.5 (d, *J* = 7.36 Hz), 115.7 (d, *J* = 18.85 Hz), 106.9, 53.7, 50.0, 45.8, 31.9; MS (ESI positive) *m*/*z*: 380.1 [M + H]^+^; [α]_D_^22°^ = +10 (*c* = 2.7; Acetone).

#### (*R*)-6-(3-(3-(2-Chlorophenyl)ureido)pyrrolidin-1-yl)pyridine-3-sulfonamide (**12a**)

Following the general procedure, the product was a white solid **12a**, yield 60%. ^**1**^**H NMR** (400 MHz, DMSO-*d*_6_) δ(ppm): 8.62 (1H, bs), 8.40 (1H, m), 8.22 (1H, s), 7.47–7.42 (3H, m), 7.29 (2H, bs), 7.11 (1H, aps), 6.98 (1H, aps), 6.66 (1H, bs), 4.37 (1H, m), 3.74 (1H, m), 3.61 (3H, m), 2.25 (1H, m), 2.00 (1H, m); ^**13**^**C NMR** (100 MHz, DMSO-*d*_6_) δ(ppm): 159.1, 155.2, 149.9, 147.3, 137.4, 130.0, 128.4, 125.5, 123.4, 122.6, 121.4, 116.3, 53.7, 50.1, 45.9, 31.9; MS (ESI positive) *m*/*z*: 396.1 [M + H]^+^; [α]_D_^22°^ = −20 (*c* = 1.3; Acetone); Elemental analysis: calculated: C, 48.55; H, 4.58; Cl, 8.96; N, 17.69; O, 12.12; S, 8.10; found: C, 47.42; H, 4.51; N, 17.18.

#### (*S*)-6-(3-(3-(2-Chlorophenyl)ureido)pyrrolidin-1-yl)pyridine-3-sulfonamide (**12b**)

Following the general procedure, the product was a white solid **12b**, yield 66%. ^**1**^**H NMR** (400 MHz, DMSO-*d*_6_) δ(ppm): 8.49 (1H, d, *J* = 2.27 Hz), 8.22 (1H, dd, *J* = 8.23, 0.93 Hz), 7.99 (1H, s), 7.86 (1H, dd, *J* = 8.96, 2.40 Hz), 7.44–7.42 (2H, m), 7.28 (1H, m), 7.18 (2H, bs), 6.99 (1H, m), 6.65 (1H, d, *J* = 9.00 Hz), 4.39 (1H, m), 3.75–3.71 (1H, m), 3.59 (2H, m), 3.45–3.42 (1H, m), 2.29–2.25 (1H, m), 2.02–1.97 (1H, m); ^**13**^**C NMR** (100 MHz, DMSO-*d*_6_) δ(ppm): 158.6, 155.2, 147.2, 137.4, 135.7, 130.0, 128.4, 128.3, 123.4, 121.8, 121.3, 106.7, 53.6, 50.0, 45.8, 31.9; MS (ESI positive) *m*/*z*: 396.1 [M + H]^+^; [α]_D_^22°^ = +18 (*c* = 1.0; Acetone).

#### (*R*)-6-(3-(3-(4-Phenoxyphenyl)ureido)pyrrolidin-1-yl)pyridine-3-sulfonamide (**13a**)

Following the general procedure, the product was a white solid **13a**, yield 69%. ^**1**^**H NMR** (400 MHz, DMSO-*d*_6_) δ(ppm): 8.48 (1H, bs), 8.39 (1H, m), 7.85 (1H, s), 7.44–7.38 (4H, m), 7.19 (2H, bs), 7.11 (1H, d, *J* = 6.01 Hz), 6.97 (4H, m), 6.63 (1H, s), 6.56 (1H, s), 4.37 (1H, m), 3.73 (1H, m), 3.58 (2H, m), 3.37 (1H, m), 2.26 (1H, m), 1.99 (1H, m); ^**13**^**C NMR** (100 MHz, DMSO-*d*_6_) δ(ppm): 158.7, 158.6, 155.9, 151.0, 147.2, 137.3, 135.6, 130.8, 128.2, 123.5, 120.7, 120.3, 118.3, 106.7, 53.6, 50.0, 45.8, 31.9; MS (ESI positive) *m*/*z*: 454.1 [M + H]^+^; [α]_D_^22°^ = −13 (*c* = 1.8; Acetone).

#### (*S*)-6-(3-(3-(4-Phenoxyphenyl)ureido)pyrrolidin-1-yl)pyridine-3-sulfonamide (**13b**)

Following the general procedure, the product was a white solid **13b**, yield 55%. ^**1**^**H NMR** (400 MHz, DMSO-*d*_6_) δ(ppm): 8.49 (1H, bs), 8.37 (1H, s), 7.86 (1H, dd, *J* = 8.90. 1.91 Hz), 7.44 (2H, d, *J* = 8.76 Hz), 7.38 (2H, t, *J* = 7.84 Hz), 7.18 (2H, bs), 7.11 (1H, t, *J* = 7.32 Hz), 6.97 (4H, m), 6.63 (1H, d, *J* = 9.00 Hz), 6.55 (1H, d, *J* = 6.70 Hz), 4.38 (1H, m), 3.75–3.71 (1H, m), 3.58 (2H, m), 3.43–3.41 (1H, m), 2.29–2.24 (1H, m), 2.02–1.97 (1H, m); ^**13**^**C NMR** (100 MHz, DMSO-*d*_6_) δ(ppm): 158.7, 158.6, 155.9, 151.0, 147.2, 137.3, 135.6, 130.8, 128.2, 123.5, 120.7, 120.3, 118.3, 106.7, 53.6, 50.0, 45.8, 31.9; MS (ESI positive) *m*/*z*: 454.1 [M + H]^+^; [α]_D_^22°^ = +11 (*c* = 2.9; Acetone).

#### (*R*)-6-(3-(3-(3,4-Dichlorophenyl)ureido)pyrrolidin-1-yl)pyridine-3-sulfonamide (**14a**)

Following the general procedure, the product was a white solid **14a**, yield 54%. ^**1**^**H NMR** (400 MHz, DMSO-*d*_6_) δ(ppm): 8.71 (1H, bs), 8.47 (1H, s), 7.87–7.84 (2H, m), 7.49 (1H, d, *J* = 8.78 Hz), 7.28 (1H, d, *J* = 8.56 Hz), 7.19 (2H, bs), 6.76 (1H, d, *J* = 6.41 Hz), 6.62 (1H, d, *J* = 8.95 Hz), 4.36 (1H, m), 3.73 (1H, m), 3.58 (2H, m), 3.43 (1H, m), 2.28–2.24 (1H, m), 2.02–1.99 (1H, m); ^**13**^**C NMR** (100 MHz, DMSO-*d*_6_) δ(ppm): 158.6, 155.5, 147.2, 141.4, 135.6, 131.8, 131.3, 128.3, 123.3, 119.7, 118.7, 106.7, 53.4, 50.1, 45.8, 31.8; MS (ESI positive) *m*/*z*: 430.0 [M + H]^+^; [α]_D_^22°^ = −10 (*c* = 1.9; Acetone).

#### (*S*)-6-(3-(3-(3,4-Dichlorophenyl)ureido)pyrrolidin-1-yl)pyridine-3-sulfonamide (**14b**)

Following the general procedure, the product was a white solid **14b**, yield 43%. ^**1**^**H NMR** (400 MHz, DMSO-*d*_6_) δ(ppm): 9.11 (1H, bs), 8.47 (1H, s), 7.88–7.85 (2H, m), 7.48 (1H, d, *J* = 8.48 Hz), 7.29 (1H, d, *J* = 7.92 Hz), 7.20 (2H, bs), 7.06 (1H, aps), 6.64 (1H, d, *J* = 8.63 Hz), 4.36 (1H, m), 3.75 (1H, m), 3.58 (2H, m), 3.41 (1H, m), 2.27 (1H, m), 2.00–1.99 (1H, m); ^**13**^**C NMR** (100 MHz, DMSO-*d*_6_) δ(ppm): 158.3, 155.6, 146.8, 141.5, 135.8, 131.8, 131.3, 128.3, 123.1, 119.5, 118.6, 107.0, 53.5, 50.0, 45.9, 31.7; MS (ESI positive) *m*/*z*: 430.0 [M + H]^+^; [α]_D_^22°^ = +12 (*c* = 2.2; Acetone).

#### (*R*)-6-(3-(3-(2,3-Dihydrobenzo[*b*][1,4]dioxin-6-yl)ureido)pyrrolidin-1-yl)pyridine-3-sulfonamide (**15a**)

Following the general procedure, the product was a white solid **15a**, yield 54%. ^**1**^**H NMR** (400 MHz, DMSO-*d*_6_) δ(ppm): 8.47 (1H, d, *J* = 1.67 Hz), 8.16 (1H, bs), 7.84 (1H, dd, *J* = 8.92. 1.90 Hz), 7.19 (2H, bs), 7.07 (1H, s), 6.73 (2H, s), 6.62 (1H, d, *J* = 9.01 Hz), 6.46 (1H, d, *J* = 6.65 Hz), 4.34 (1H, m), 4.33–4.20 (4H, m), 3.71–3.69 (1H, m), 3.57 (2H, m), 3.38 (1H, m), 2.26–2.22 (1H, m), 1.99–1.96 (1H, m); ^**13**^**C NMR** (100 MHz, DMSO-*d*_6_) δ(ppm): 158.6, 155.8, 147.2, 143.9, 138.8, 135.6, 134.9, 128.2, 117.6, 112.0, 107.9, 106.7, 65.1, 53.6, 49.9, 45.8, 31.9; MS (ESI positive) *m*/*z*: 420.1 [M + H]^+^.

#### (*S*)-6-(3-(3-(2,3-Dihydrobenzo[*b*][1,4]dioxin-6-yl)ureido)pyrrolidin-1-yl)pyridine-3-sulfonamide (**15b**)

Following the general procedure, the product was a white solid **15b**, yield 42%. ^**1**^**H NMR** (400 MHz, DMSO-*d*_6_) δ(ppm): 8.76, (1H, bs), 8.47 (1H, s), 7.85 (1H, dd, *J* = 8.92. 1.88 Hz),7.70–7.65 (1H, m), 7.18 (2H, bs), 7.08 (1H, s), 6.73 (2H, s), 6.62 (1H, d, *J* = 9.01 Hz), 4.34 (1H, m), 4.33–4.19 (4H, m), 3.74–3.69 (1H, m), 3.58 (2H, m), 3.39 (1H, m), 2.26–2.22 (1H, m), 1.99–1.96 (1H, m); ^**13**^**C NMR** (100 MHz, DMSO-*d*_6_) δ(ppm): 158.5, 155.9, 147.0, 143.9, 138.8, 135.7, 128.2, 126.1, 117.6, 111.9, 107.8, 106.8, 65.1, 64.7, 53.6, 49.9, 45.9, 31.9; MS (ESI positive) *m*/*z*: 420.1 [M + H]^+^.

#### (*R*)-6-(3-(3-(3,5-Bis(trifluoromethyl)phenyl)ureido)pyrrolidin-1-yl)pyridine-3-sulfonamide (**16a**)

Following the general procedure, the product was a white solid **16a**, yield 75%. ^**1**^**H NMR** (400 MHz, DMSO-*d*_6_) δ(ppm): 9.14 (1H, bs), 8.48 (1H, s), 8.12 (2H, s), 7.85 (1H, d, *J* = 8.72 Hz), 7.60 (1H, s), 7.19 (2H, bs), 6.98 (1H, d, *J* = 6.13 Hz), 6.63 (1H, d, *J* = 8.92 Hz), 4.39 (1H, m), 3.77–3.73 (1H, m), 3.60 (2H, m), 3.46 (1H, m), 2.28–2.25 (1H, m), 2.03 (1H, m); ^**13**^**C NMR** (100 MHz, DMSO-*d*_6_) δ(ppm): 158.6, 155.5, 147.2, 143.2, 135.6, 131.5 (q, *J* = 32.57 Hz), 128.3, 122.9 (q, *J* = 272.42 Hz), 118.3, 114.6, 106.7, 53.3, 50.2, 45.8, 31.7; ^**19**^**F NMR** (376 MHz, DMSO-*d*_6_) δ(ppm): −61.7; MS (ESI positive) *m*/*z*: 498.1 [M + H]^+^; [α]_D_^22°^ = −12 (*c* = 1.0; Acetone).

#### (*S*)-6-(3-(3-(3,5-Bis(trifluoromethyl)phenyl)ureido)pyrrolidin-1-yl)pyridine-3-sulfonamide (**16b**)

Following the general procedure, the product was a white solid **16a**, yield 67%. ^**1**^**H NMR** (400 MHz, DMSO-*d*_6_) δ(ppm): 9.39 (1H, bs), 8.48 (1H, s), 8.12 (2H, s), 7.86 (1H, d, *J* = 8.29 Hz), 7.58 (1H, s), 7.19 (3H, bs), 6.64 (1H, d, *J* = 8.79 Hz), 4.39 (1H, m), 3.75–3.74 (3H, m), 3.46 (1H, m), 2.29–2.28 (1H, m), 2.04–2.03 (1H, m); ^**13**^**C NMR** (100 MHz, DMSO-*d*_6_) δ(ppm): 158.5, 155.6, 147.1, 143.4, 135.7, 131.5 (q, *J* = 32.45 Hz), 128.3, 124.3 (q, *J* = 272.61 Hz), 114.5, 106.8, 53.4, 50.2, 45.9, 31.6; MS (ESI positive) *m*/*z*: 498.1 [M + H]^+^; [α]_D_^22°^ = +10 (*c* = 1.4; Acetone).

#### (*R*)-2-(3,6-Dihydroxy-9*H*-xanthen-9-yl)-5-(3-(1-(5-sulfamoylpyridin-2-yl)pyrrolidin-3-yl)thioureido)benzoic Acid (**17a**)

Following the general procedure, the product was an orange solid **17a**, yield 41%. ^**1**^**H NMR** (400 MHz, DMSO-*d*_6_) δ(ppm): 10.13 (1H, bs), 8.65 (1H, bs), 8.49 (1H, d, *J* = 2.01 Hz), 8.39 (2H, s), 7.87 (1H, dd, *J* = 8.95, 2.28 Hz), 7.82 (1H, *J* = 8.12 Hz), 7.22 (3H, m), 6.71 (2H, s), 6.66–6.59 (6H, m), 4.93 (1H, m), 3.88–3.83 (1H, m), 3.64 (3H, m), 2.44–2.36 (1H, m), 2.18–2.15 (1H, m); ^**13**^**C NMR** (100 MHz, DMSO-*d*_6_) δ(ppm): 181.4, 169.5, 160.4, 158.6, 152.8, 147.2, 142.4, 135.7, 130.0, 129.2, 128.4, 127.4, 126.2, 124.9, 114.6, 113.6, 110.7, 106.8, 103.2, 56.0, 54.2, 53.1, 46.0, 31.6; MS (ESI positive) *m*/*z*: 634.1 [M + H]^+^; [α]_D_^22°^ = −9 (*c* = 1.0; Acetone).

#### (*S*)-2-(3,6-Dihydroxy-9*H*-xanthen-9-yl)-5-(3-(1-(5-sulfamoylpyridin-2-yl)pyrrolidin-3-yl)thioureido)benzoic Acid (**17b**)

Following the general procedure, the product was an orange solid **17b**, yield 34%. ^**1**^**H NMR** (400 MHz, DMSO-*d*_6_) δ(ppm): 10.16 (1H, bs), 9.94 (1H, bs), 8.54 (1H, bs), 8.50 (1H, d, *J* = 2.16 Hz), 8.37 (1H, s), 7.87 (1H, dd, *J* = 8.96, 2.33 Hz), 7.81 (1H, *J* = 8.01 Hz), 7.20 (2H, m), 6.71 (2H, s), 6.66–6.59 (6H, m), 4.95 (1H, m), 3.89–3.84 (1H, m), 3.64–3.56 (4H, m), 2.41–2.37 (1H, m), 2.19–2.12 (1H, m); ^**13**^**C NMR** (100 MHz, DMSO-*d*_6_) δ(ppm): 181.4, 169.4, 160.5, 160.4, 158.6, 152.7, 148.2, 147.2, 142.4, 135.7, 129.9, 128.4, 127.3, 124.9, 113.5, 110.6, 106.8, 103.2, 54.2, 52.9, 45.9, 31.2; MS (ESI positive) *m*/*z*: 634.1 [M + H]^+^; [α]_D_^22°^ = +6 (*c* = 4.3; Acetone).

#### (*R*)-6-(3-(3-(2-Methoxyphenyl)ureido)pyrrolidin-1-yl)pyridine-3-sulfonamide (**18a**)

Following the general procedure, the product was a white solid **18a**, yield 41%. ^**1**^**H NMR** (400 MHz, DMSO-*d*_6_) δ(ppm): 8.48 (1H, s), 8.13 (1H, d, *J* = 7.61 Hz), 7.91 (1H, s), 7.85 (1H, dd, *J* = 8.93, 1.97 Hz), 7.27 (1H, *J* = 6.65 Hz), 7.19 (2H, bs), 6.99 (1H, d, *J* = 7.50 Hz), 6.92–6.85 (2H, m), 6.64 (1H, d, *J* = 9.0 Hz), 4.36 (1H, m), 3.85 (3H, s), 3.73–3.68 (1H, m), 3.58 (2H, m), 3.43 (1H, m), 2.27–2.22 (1H, m), 1.98–1.94 (1H, m); ^**13**^**C NMR** (100 MHz, DMSO-*d*_6_) δ(ppm): 158.7, 155.7, 148.1, 147.3, 135.7, 130.2, 128.7, 122.0, 121.4, 118.7, 111.5, 106.8, 56.6, 53.8, 49.9, 45.8, 32.0; MS (ESI positive) *m*/*z*: 392.1 [M + H]^+^; [α]_D_^22°^ = −14 (*c* = 1.8; Acetone).

#### (*S*)-6-(3-(3-(2-Methoxyphenyl)ureido)pyrrolidin-1-yl)pyridine-3-sulfonamide (**18b**)

Following the general procedure, the product was a white solid **18b**, yield 41%. ^**1**^**H NMR** (400 MHz, DMSO-*d*_6_) δ(ppm): 8.48 (1H, s), 8.13 (1H, d, *J* = 7.14 Hz), 7.89 (1H, s), 7.85 (1H, d, *J* = 7.33 Hz), 7.29 (1H, m), 7.18 (2H, bs), 6.98 (1H, d, *J* = 7.23 Hz), 6.92–6.86 (2H, m), 6.63 (1H, d, *J* = 8.93 Hz), 4.36 (1H, m), 3.84 (3H, s), 3.73–3.69 (2H, m), 3.49–3.42 (2H, m), 2.27–2.23 (1H, m), 1.98–1.95 (1H, m); ^**13**^**C NMR** (100 MHz, DMSO-*d*_6_) δ(ppm): 158.7, 155.7, 148.1, 147.2, 135.7, 130.2, 128.3, 122.0, 121.4, 118.7, 111.5, 106.7, 56.6, 53.8, 49.9, 45.8, 31.6; MS (ESI positive) *m*/*z*: 392.1 [M + H]^+^; [α]_D_^22°^ = +12 (*c* = 1.0; Acetone).

#### (*R*)-6-(3-(3-(4-Nitrophenyl)ureido)pyrrolidin-1-yl)pyridine-3-sulfonamide (**19a**)

Following the general procedure, the product was a yellow solid **19a**, yield 61%. ^**1**^**H NMR** (400 MHz, DMSO-*d*_6_) δ(ppm): 9.19 (1H, bs), 8.48 (1H, d, *J* = 1.70 Hz), 8.18 (2H, d, *J* = 9.06 Hz), 7.85 (1H, dd, *J* = 8.91, 1.87 Hz), 7.65 (2H, d, *J* = 9.06 Hz), 7.19 (2H, bs), 6.92 (1H, d, *J* = 6.63 Hz), 6.63 (2H, d, *J* = 8.98 Hz), 4.40 (1H, m), 3.76–3.72 (1H, m), 3.59 (2H, m), 3.43 (1H, m), 2.30–2.27 (1H, m), 2.04–2.00 (1H, m); ^**13**^**C NMR** (100 MHz, DMSO-*d*_6_) δ(ppm): 158.6, 155.1, 147.8, 147.2, 141.5, 135.7, 128.3, 126.1, 117.8, 106.8, 53.4, 50.1, 45.8, 37.7; MS (ESI positive) *m*/*z*: 407.1 [M + H]^+^; [α]_D_^22°^ = −26 (*c* = 3.1; Acetone).

#### (*S*)-6-(3-(3-(4-Nitrophenyl)ureido)pyrrolidin-1-yl)pyridine-3-sulfonamide (**19b**)

Following the general procedure, the product was a yellow solid **19b**, yield 58%. ^**1**^**H NMR** (400 MHz, DMSO-*d*_6_) δ(ppm): 9.15 (1H, bs), 8.49 (1H, d, *J* = 2.11 Hz), 8.18 (2H, d, *J* = 9.12 Hz), 7.85 (1H, dd, *J* = 8.95, 2.26 Hz), 7.65 (2H, d, *J* = 9.14 Hz), 7.18 (2H, bs), 6.89 (1H, d, *J* = 6.66 Hz), 6.63 (1H, d, *J* = 9.00 Hz), 4.41 (1H, m), 3.77–3.72 (1H, m), 3.59 (2H, m), 3.46 (1H, m), 2.31–2.26 (1H, m), 2.05–2.00 (1H, m); ^**13**^**C NMR** (100 MHz, DMSO-*d*_6_) δ(ppm): 158.6, 155.1, 147.8, 147.2, 141.5, 135.7, 128.3, 126.1, 117.8, 106.7, 53.4, 50.1, 45.8, 31.7; MS (ESI positive) *m*/*z*: 407.1 [M + H]^+^; [α]_D_^22°^ = +29 (*c* = 3.2; Acetone).

#### (*R*)-6-(3-(3-(4-(Trifluoromethyl)phenyl)ureido)pyrrolidin-1-yl)pyridine-3-sulfonamide (**20a**)

Following the general procedure, the product was a white solid **20a**, yield 72%. ^**1**^**H NMR** (400 MHz, DMSO-*d*_6_) δ(ppm): 8.81 (1H, bs), 8.48 (1H, d, *J* = 1.67 Hz), 7.85 (1H, dd, *J* = 8.91, 2.01 Hz), 7.62 (4H, aps), 7.19 (2H, bs), 6.75 (1H, d, *J* = 6.57 Hz), 6.63 (2H, d, *J* = 8.98 Hz), 4.39 (1H, m), 3.76–3.72 (1H, m), 3.59 (2H, m), 3.44 (1H, m), 2.29–2.25 (1H, m), 2.03–1.9 (1H, m); ^**13**^**C NMR** (100 MHz, DMSO-*d*_6_) δ(ppm): 158.7, 155.5, 147.2, 144.9, 135.7, 129.3, 126.9, 126.8, 124.2, 122.2 (q, *J* = 30.81 Hz), 118.2, 106.7, 53.5, 50.0, 45.8, 31.8; ^**19**^**F NMR** (376 MHz, DMSO-*d*_6_) δ(ppm): −59.9; MS (ESI positive) *m*/*z*: 430.1 [M + H]^+^; [α]_D_^22°^ = −22 (*c* = 3.9; Acetone).

#### (*S*)-6-(3-(3-(4-(Trifluoromethyl)phenyl)ureido)pyrrolidin-1-yl)pyridine-3-sulfonamide (**20b**)

Following the general procedure, the product was a white solid **20b**, yield 56%. ^**1**^**H NMR** (400 MHz, DMSO-*d*_6_) δ(ppm): 9.20 (1H, bs), 8.47 (1H, d, *J* = 2.04 Hz), 7.85 (1H, dd, *J* = 8.93, 2.16 Hz), 7.62 (4H, apq, *J* = 8.82 Hz), 7.19 (2H, bs), 7.18 (1H, bs), 6.63 (1H, d, *J* = 8.99 Hz), 4.39 (1H, m), 3.76–3.72 (1H, m), 3.60–3.58 (2H, m), 3.43–3.41 (1H, m), 2.29–2.25 (1H, m), 2.02–2.00 (1H, m); ^**13**^**C NMR** (100 MHz, DMSO-*d*_6_) δ(ppm): 158.5, 155.6, 147.1, 145.1, 135.7, 128.3, 126.9, 123.2 (q, *J* = 212.9 Hz), 121.9 (q, *J* = 30.81 Hz), 118.1, 106.8, 53.5, 50.0, 45.9, 31.8; MS (ESI positive) *m*/*z*: 430.1 [M + H]^+^;[α]_D_^22°^ = +20 (*c* = 6.0; Acetone).

#### (*R*)-6-(3-(3-(2-Fluorophenyl)thioureido)pyrrolidin-1-yl)pyridine-3-sulfonamide (**21a**)

Following the general procedure, the product was a pale yellow solid **21a**, yield 73%. ^**1**^**H NMR** (400 MHz, DMSO-*d*_6_) δ(ppm): 9.18 (1H, bs), 8.49 (1H, s), 8.37 (1H, s), 7.86 (2H, d, *J* = 8.71 Hz), 7.29–7.19 (4H, m), 6.64 (1H, d, *J* = 8.91 Hz), 4.91 (1H, m), 3.84–3.80 (1H, m), 3.60–3.54 (3H, m), 2.36–2.32 (1H, m), 2.15 (1H, m); ^**13**^**C NMR** (100 MHz, DMSO-*d*_6_) δ(ppm):182.2, 158.6, 147.2, 135.7, 128.3, 128.1, 124.8 (d, *J* = 3.07 Hz), 116.4 (d, *J* = 19.9 Hz), 106.8, 54.4, 52.9, 45.9, 31.3; ^**19**^**F NMR** (376 MHz, DMSO-*d*_6_) δ(ppm): −124.0; MS (ESI positive) *m*/*z*: 396.1 [M + H]^+^; [α]_D_^22°^ = −27 (*c* = 3.5; Acetone).

#### (*S*)-6-(3-(3-(2-Fluorophenyl)thioureido)pyrrolidin-1-yl)pyridine-3-sulfonamide (**21b**)

Following the general procedure, the product was a pale yellow solid **21b**, yield 51%. ^**1**^**H NMR** (400 MHz, DMSO-*d*_6_) δ(ppm): 9.19 (1H, bs), 8.49 (1H, d, *J* = 1.99 Hz), 8.38 (1H, s), 7.86 (2H, dd, *J* = 8.77, 1.83 Hz), 7.24–7.19 (4H, m), 6.64 (1H, d, *J* = 9.03 Hz), 4.91 (1H, m), 3.85–3.80 (1H, m), 3.61–3.51 (3H, m), 2.37–2.32 (1H, m), 2.12 (1H, m); ^**13**^**C NMR** (100 MHz, DMSO-*d*_6_) δ(ppm):182.3, 158.7, 156.7 (d, *J* = 381.42 Hz), 147.2, 135.9, 128.5, 128.4, 125.0 (d, *J* = 2.86 Hz), 116.6 (d, *J* = 19.92 Hz), 107.0, 54.6, 53.1, 46.1, 31.4; MS (ESI positive) *m*/*z*: 396.1 [M + H]^+^; [α]_D_^22°^ = +30 (*c* = 1.1; Acetone).

#### (*R*)-6-(3-(3-(2-Bromo-4,6-difluorophenyl)ureido)pyrrolidin-1-yl)pyridine-3-sulfonamide (**22a**)

Following the general procedure, the product was a white solid **22a**, yield 60%. ^**1**^**H NMR** (400 MHz, DMSO-*d*_6_) δ(ppm): 8.48 (1H, d, *J* = 2.15 Hz), 7.85 (1H, dd, *J* = 8.95, 2.30 Hz), 7.72 (1H, s), 7.55 (1H, m), 7.42 (1H, td, *J* = 9.59, 2.63 Hz), 7.18 (2H, bs), 6.88 (1H, d, *J* = 6.83 Hz), 6.62 (1H, d, *J* = 9.00 Hz), 4.34 (1H, m), 3.75–3.70 (1H, m), 3.57 (2H, m), 3.36 (1H, m), 2.27–2.23 (1H, m), 2.02–1.97 (1H, m); ^**13**^**C NMR** (100 MHz, DMSO-*d*_6_) δ(ppm): 161.6, 160.6, 158.6, 158.1, 155.7, 147.2, 135.6, 128.2, 124.7, (d, *J* = 14.75 Hz), 124.1 (d, *J* = 15.73 Hz), 116.2 (d, *J* = 22.42 Hz), 106.7, 104.9 (t, *J* = 26.12 Hz), 53.5, 50.3, 45.8, 31.8; ^**19**^**F NMR** (376 MHz, DMSO-*d*_6_) δ(ppm): −110.9, -112.1; MS (ESI positive) *m*/*z*: 476.0 [M + H]^+^; [α]_D_^22°^ = −12 (*c* = 3.7; Acetone).

#### (*S*)-6-(3-(3-(2-Bromo-4,6-difluorophenyl)ureido)pyrrolidin-1-yl)pyridine-3-sulfonamide (**22b**)

Following the general procedure, the product was a white solid **22b**, yield 44%. ^**1**^**H NMR** (400 MHz, DMSO-*d*_6_) δ(ppm): 8.48 (1H, d, *J* = 2.30 Hz), 7.85 (1H, dd, *J* = 8.98, 2.45 Hz), 7.76 (1H, s), 7.57–7.55 (1H, m), 7.43 (1H, td, *J* = 9.64, 2.77 Hz), 7.19 (2H, bs), 6.91 (1H, d, *J* = 6.50 Hz), 6.63 (1H, d, *J* = 9.01 Hz), 4.34 (1H, m), 3.75–3.71 (1H, m), 3.61–3.58 (2H, m), 3.40 (1H, m), 2.27–2.22 (1H, m), 2.02–1.97 (1H, m); ^**13**^**C NMR** (100 MHz, DMSO-*d*_6_) δ(ppm): 158.6, 155.8, 147.1, 135.7, 128.2, 116.4, 116.1, 106.8, 105.4, 105.2, 53.6, 50.3, 45.9, 31.9; MS (ESI positive) *m*/*z*: 476.0 [M + H]^+^; [α]_D_^22°^ = +14 (*c* = 1.7; Acetone).

### General Synthesis of Compounds **24** and **25**

Compound **5a/5b** (1 equiv) in acetonitrile was added to benzenesulfonyl isocyanate **23a-b** (1 equiv) and Et_3_N (3 equiv). The solution was stirred overnight at room temperature. The reaction was quenched with saturated solution of NH_4_Cl, extracted with EtOAc, and dried over Na_2_SO_4_. The crude material was purified by flash column chromatography (MeOH/DCM: 5:95), to yield compounds **24** or **25**.

#### (*R*)-6-(3-(3-(Phenylsulfonyl)ureido)pyrrolidin-1-yl)pyridine-3-sulfonamide (**24a**)

Following the general procedure, the product was a white solid **24a**, yield 56%. ^**1**^**H NMR** (400 MHz, DMSO-*d*_6_) δ(ppm): 8.45 (1H, d, *J* = 2.21 Hz), 7.92 (2H, d, *J* = 7.44 Hz), 7.83 (1H, dd, *J* = 8.93, 2.43 Hz) 7.68 (1H, m), 7.61 (2H, t, *J* = 7.46 Hz), 7.50 (1H, m), 7.18 (2H, bs), 6.89 (1H, bs), 6.57 (1H, d, *J* = 8.99 Hz), 4.19 (1H, m), 3.63 (1H, m), 3.50 (3H, m), 2.16 (1H, m), 1.91 (1H, m); ^**13**^**C NMR** (100 MHz, DMSO-*d*_6_) δ(ppm): 158.6, 153.3, 147.2, 135.6, 129.7, 128.8, 128.3, 127.9, 127.7, 106.7, 53.0, 50.2, 45.7, 31.4; MS (ESI positive) *m*/*z*: 426.1 [M + H]^+^.

#### (*R*)-6-(3-(3-((2-Chlorophenyl)sulfonyl)ureido)pyrrolidin-1-yl)pyridine-3-sulfonamide (**25a**)

Following the general procedure, the product was a white solid **25a**, yield 36%. ^**1**^**H NMR** (400 MHz, DMSO-*d*_6_) δ(ppm): 8.46 (1H, d, *J* = 1.62 Hz), 8.07 (1H, d, *J* = 7.74 Hz), 7.85–7.83 (2H, m), 7.67 (2H, aps), 7.50 (1H, s), 7.19 (2H, bs), 6.86 (1H, aps), 6.58 (1H, d, *J* = 8.98 Hz), 4.18 (1H, m), 3.65–3.61 (1H, m), 3.50–3.48 (3H, m), 2.17 (1H, m), 1.95–1.88 (1H, m); ^**13**^**C NMR** (100 MHz, DMSO-*d*_6_) δ(ppm): 158.6, 147.2, 135.7, 135.3, 132.6, 132.4, 131.8, 131.5, 131.4, 128.3, 127.5, 106.8, 53.1, 45.8, 31.6, 30.6; MS (ESI positive) *m*/*z*: 460.0 [M + H]^+^; [α]_D_^22°^ = −14 (*c* = 1.4; Acetone).

#### (*S*)-6-(3-(3-((2-Chlorophenyl)sulfonyl)ureido)pyrrolidin-1-yl)pyridine-3-sulfonamide (**25b**)

Following the general procedure, the product was a white solid **25b**, yield 35%. ^**1**^**H NMR** (400 MHz, DMSO-*d*_6_) δ(ppm): 8.50 (1H, s), 8.23 (1H, d, *J* = 8.17 Hz), 7.99 (1H, s), 7.86 (1H, dd, *J* = 8.91, 1.96 Hz), 7.44–7.42 (2H, m), 7.28 (2H, t,, *J* = 7.48 Hz), 7.19 (2H, bs), 6.99 (1H, dd,, *J* = 11.09, 4.11 Hz), 6.64 (1H, d, *J* = 9.00 Hz), 4.39 (1H, m), 3.75–3.71 (1H, m), 3.45 (2H, m), 3.43 (1H, m), 2.29–2.25 (1H, m), 2.00–1.98 (1H, m); ^**13**^**C NMR** (100 MHz, DMSO-*d*_6_) δ(ppm): 158.7, 155.3, 147.3, 137.5, 135.7, 130.1, 128.5, 128.4, 123.4, 121.9, 121.4. 106.8, 53.7, 50.1. 45.9, 32.1; MS (ESI positive) *m*/*z*: 460.0 [M + H]^+^; [α]_D_^22°^ = +15 (*c* = 1.2; Acetone).

### General Synthesis of Compounds **27–29**

To a solution of 2-chloropyridine (**26a-c**, 1 equiv) and K_2_CO_3_ (1.3 equiv) in dry DMF and inert atmosphere (N_2_) was added pyrrolidine (**3a,b**, 1 equiv). The mixture was stirred for 4 h at 100 °C. The reaction mixture was quenched with ice-cooled, saturated NH_4_Cl solution and stirred for 15 min to give a precipitate, which was collected by vacuum filtration and washed with water. The obtained solid was triturated with Et_2_O to yield the derivatives **27–29**.

#### *tert*-Butyl (*R*)-(1-(Pyridin-2-yl)pyrrolidin-3-yl)carbamate (**27a**)

Following the general procedure, the product was a white solid **27a**, yield 76%. ^**1**^**H NMR** (400 MHz, DMSO-*d*_6_) δ(ppm): 8.08 (1H, d, *J* = 3.29 Hz), 7.50 (1H, t, *J* = 7.13 Hz), 7.22 (1H, d, *J* = 4.67 Hz), 6.56 (1H, m), 6.43 (1H, d, *J* = 8.40 Hz), 4.14 (1H, m), 3.60 (1H, m), 3.52 (1H, m), 3.49 (1H, m), 3.23 (1H, m), 2.16 (1H, m), 1.92 (1H, m), 1.43 (9H, s); ^**13**^**C NMR** (100 MHz, DMSO-*d*_6_) δ(ppm): 157.8, 156.2, 148.7, 137.8, 112.1, 107.1, 78.7, 52.9, 50.8, 40.4, 31.5, 29.1; MS (ESI positive) *m*/*z*: 263.2 [M + H]^+^.

#### *tert*-Butyl (*R*)-(1-(6-Nitropyridin-2-yl)pyrrolidin-3-yl)carbamate(**28a**)

Following the general procedure, the product was a yellow solid **28a**, yield 79%. ^**1**^**H NMR** (400 MHz, DMSO-*d*_6_) δ(ppm): 8.43 (1H, m), 8.22 (1H, m), 7.26 (1H, d, *J* = 4.58 Hz), 6.84 (1H, dd, *J* = 7.96, 4.51 Hz), 4.10 (1H, m), 3.52–3.44 (1H, m), 3.43–3.41 (2H, m), 3.12–3.08 (1H, m), 2.12–2.09 (1H, m), 1.94–1.90 (1H, m), 1.41 (9H, s); ^**13**^**C NMR** (100 MHz, DMSO-*d*_6_) δ(ppm): 156.1, 153.0, 150.6, 135.8, 132.3, 112.6, 78.8, 55.0, 50.6, 48.0, 30.9, 29.1; MS (ESI positive) *m*/*z*: 309.2 [M + H]^+^.

#### *tert*-Butyl (*S*)-(1-(6-Nitropyridin-2-yl)pyrrolidin-3-yl)carbamate(**28b**)

Following the general procedure, the product was a yellow solid **28b**, yield 92%. ^**1**^**H NMR** (400 MHz, DMSO-*d*_6_) δ(ppm): 8.43 (1H, dd, *J* = 4.47, 1.53 Hz), 8.22 (1H, dd, *J* = 8.03, 1.52 Hz), 7.25 (1H, d, *J* = 4.52 Hz), 6.85 (1H, dd, *J* = 8.03, 4.51 Hz), 4.09 (1H, m), 3.52–3.42 (1H, m), 3.12–3.08 (2H, m), 3.10 (1H, dd, *J* = 11.32, 4.57 Hz), 2.14–2.09 (1H, m), 1.94–1.91 (1H, m), 1.41 (9H, s); ^**13**^**C NMR** (100 MHz, DMSO-*d*_6_) δ(ppm): 156.1, 153.1, 150.6, 135.8, 132.3, 112.6, 78.8, 55.0, 50.7, 48.0, 30.9, 29.1; MS (ESI positive) *m*/*z*: 309.2 [M + H]^+^.

#### *tert*-Butyl (*R*)-(1-(5-(Trifluoromethyl)pyridin-2-yl)pyrrolidin-3-yl)carbamate (**29a**)

Following the general procedure, the product was a white solid **29a**, yield 70%. ^**1**^**H NMR** (400 MHz, DMSO-*d*_6_) δ(ppm): 8.41 (1H, s), 7.77 (1H, dd, *J* = 8.94, 2.21 Hz), 7.26 (1H, d, *J* = 5.53 Hz), 6.58 (1H, d, *J* = 8.96 Hz), 4.17 (1H, m), 3.70–3.65 (1H, m), 3.60–3.58 (1H, m), 3.49–3.47 (1H, m), 3.34 (1H, m), 2.20–2.16 (1H, m), 1.96–1.91 (1H, m), 1.43 (9H, s); ^**13**^**C NMR** (100 MHz, DMSO-*d*_6_) δ(ppm): 159.2, 156.2, 146.5, 146.4, 134.6, 126.1 (q, *J* = 270.01 Hz), 113.0 (q, *J* = 32.13 Hz), 106.9, 78.8, 53.1, 50.7, 45.8, 31.4, 29.1; ^**19**^**F NMR** (376 MHz, DMSO-*d*_6_) δ(ppm): −59.0; MS (ESI positive) *m*/*z*: 332.2 [M + H]^+^.

#### *tert*-Butyl (*S*)-(1-(5-(Trifluoromethyl)pyridin-2-yl)pyrrolidin-3-yl)carbamate (**29b**)

Following the general procedure, the product was a white solid **29b**, yield 89%. ^**1**^**H NMR** (400 MHz, DMSO-*d*_6_) δ(ppm): 8.41 (1H, s), 7.77 (1H, dd, *J* = 8.90, 1.98 Hz), 7.28 (1H, d, *J* = 5.89 Hz), 6.58 (1H, d, *J* = 8.95 Hz), 4.17 (1H, m), 3.69–3.67 (1H, m), 3.58 (1H, m), 3.49 (1H, m), 3.33 (1H, m), 2.20–2.15 (1H, m), 1.96–1.93 (1H, m), 1.43 (9H, s); ^**13**^**C NMR** (100 MHz, DMSO-*d*_6_) δ(ppm): 159.2, 156.2, 146.5, 134.7, 126.1 (q, *J* = 269.83 Hz), 113.0 (q, *J* = 31.97 Hz), 107.0, 78.8, 53.1, 50.7, 45.8, 31.4, 29.1; MS (ESI positive) *m*/*z*: 332.2 [M + H]^+^.

### General Synthesis of Compounds **30–32**

To the corresponding compounds **27–29** (1 equiv) in dichloromethane (DCM) was added TFA (6 equiv), and the solution was stirred overnight at room temperature. The solvents were evaporated, and the residue was dissolved in EtOAc and washed with 1 N NaOH solution to give pure compounds **30–32**.

#### (*R*)-1-(Pyridin-2-yl)pyrrolidin-3-amine (**30a**)

Following the general procedure, the product was a yellow oil **30a**, yield 68%. ^**1**^**H NMR** (400 MHz, DMSO-*d*_6_) δ(ppm): 8.07 (1H, dd, *J* = 4.79, 1.01 Hz), 7.48 (1H, m), 6.53 (1H, dd, *J* = 6.52, 5.40 Hz), 6.40 (1H, d, *J* = 8.48 Hz), 3.59–3.51 (3H, m), 3.37–3.35 (1H, m), 3.07–3.03 (1H, m), 2.10–2.03 (1H, m), 1.76–1.68 (3H, m); ^**13**^**C NMR** (100 MHz, DMSO-*d*_6_) δ(ppm): 148.7, 137.7, 11.7, 106.9, 55.9, 51.7, 45.8, 35.1; MS (ESI positive) *m*/*z*: 164.1 [M + H]^+^.

#### (*R*)-1-(6-Nitropyridin-2-yl)pyrrolidin-3-amine (**31a**)

Following the general procedure, the product was a yellow oil **31a**, yield 79%. ^**1**^**H NMR** (400 MHz, DMSO-*d*_6_) δ(ppm): 8.42 (1H, dd, *J* = 4.47, 1.58 Hz), 8.20 (1H, dd, *J* = 8.04. 1.57 Hz), 6.81 (1H, d, *J* = 8.04, 4.50 Hz), 3.61–3.55 (2H, m), 3.44–3.39 (1H, m), 3.33 (1H, dd, *J* = 11.19, 5.72 Hz), 2.91 (1H, dd, *J* = 11.17. 4.80 Hz), 2.08–2.00 (3H, m), 1.77–1.69 (1H, m); ^**13**^**C NMR** (100 MHz, DMSO-*d*_6_) δ(ppm): 153.1, 150.7, 135.8, 132.1, 112.3, 58.2, 51.5, 48.4, 34.0; MS (ESI positive) *m*/*z*: 209.1 [M + H]^+^.

#### (*S*)-1-(6-Nitropyridin-2-yl)pyrrolidin-3-amine (**31b**)

Following the general procedure, the product was a yellow oil **31b**, yield 93%. ^**1**^**H NMR** (400 MHz, DMSO-*d*_6_) δ(ppm): 8.47 (1H, dd, *J* = 4.47, 1.45 Hz), 8.26 (1H, dd, *J* = 8.03. 1.43 Hz), 8.19 (3H, bs), 6.91 (1H, d, *J* = 8.03, 4.54 Hz), 3.95 (1H, m), 3.62 (2H, m), 3.60 (1H, m), 3.59 (1H, m), 2.37–2.29 (1H, m), 2.12–2.03 (1H, m); ^**13**^**C NMR** (100 MHz, DMSO-*d*_6_) δ(ppm): 153.1, 150.2, 135.9, 132.8, 113.3, 53.0, 50.0, 47.7, 29.6; MS (ESI positive) *m*/*z*: 209.1 [M + H]^+^.

#### (*R*)-1-(5-(Trifluoromethyl)pyridin-2-yl)pyrrolidin-3-amine (**32a**)

Following the general procedure, the product was a white solid **32a**, yield 99%. ^**1**^**H NMR** (400 MHz, DMSO-*d*_6_) δ(ppm): 8.39 (1H, s), 7.75 (1H, dd, *J* = 8.91, 2.06 Hz), 6.55 (1H, d, *J* = 8.95 Hz), 3.60–3.58 (3H, m), 3.47 (1H, m), 3.16 (1H, m), 2.12–2.03 (1H, m), 1.78–1.74 (1H, m); ^**13**^**C NMR** (100 MHz, DMSO-*d*_6_) δ(ppm): 159.3, 146.5, 146.4, 134.6, 126.1 (q, *J* = 269.55 Hz), 112.6 (q, *J* = 32.17 Hz), 106.8, 55.9, 51.5, 46.0, 34.8; ^**19**^**F NMR** (376 MHz, DMSO-*d*_6_) δ(ppm): −58.9; MS (ESI positive) *m*/*z*: 232.1 [M + H]^+^.

#### (*S*)-1-(5-(Trifluoromethyl)pyridin-2-yl)pyrrolidin-3-amine (**32b**)

Following the general procedure, the product was a white solid **32b**, yield 78%. ^**1**^**H NMR** (400 MHz, DMSO-*d*_6_) δ(ppm): 8.39 (1H, s), 7.75 (1H, dd, *J* = 8.77, 1.70 Hz), 6.55 (1H, d, *J* = 8.95 Hz), 3.60–3.58 (2H, m), 3.47 (1H, m), 3.34 (1H, m), 3.16 (1H, m), 2.10–2.06 (1H, m), 1.76–1.75 (3H, m); ^**13**^**C NMR** (100 MHz, DMSO-*d*_6_) δ(ppm): 159.3, 146.5, 146.4, 134.6, 128.9 (q, *J* = 269.89 Hz), 112.6 (q, *J* = 31.98 Hz), 106.8, 56.0, 51.6, 46.1, 34.8; MS (ESI positive) *m*/*z*: 232.1 [M + H]^+^.

### General Synthesis of Compounds **35–40**

The appropriate isothiocyanate (**33a-b**, 1 equiv) was dissolved in acetonitrile and treated with the corresponding amine **30-32** (1 equiv). The mixture was stirred overnight at r.t., quenched with H_2_O, and the readily formed precipitate was collected by filtration and dried on air to afford the titled thiourea **35–40**.

#### (*R*)-4-(3-(1-(Pyridin-2-yl)pyrrolidin-3-yl)thioureido)benzenesulfonamide (**35a**)

Following the general procedure, the product was a white solid **35a**, yield 53%. ^**1**^**H NMR** (400 MHz, DMSO-*d*_6_) δ(ppm): 9.74 (1H, bs), 8.36 (1H, s), 8.11 (1H, d, *J* = 4.23 Hz), 7.77–7.72 (4H, m), 7.55–7.52 (1H, m), 7.29 (2H, bs), 6.61 (1H, t, *J* = 5.75 Hz), 6.52 (1H, d, *J* = 8.40 Hz), 4.90 (1H, m), 3.77–3.73 (1H, m), 3.56–3.47 (4H, m), 2.36–2.30 (1H, m), 2.12–2.11 (1H, m); ^**13**^**C NMR** (100 MHz, DMSO-*d*_6_) δ(ppm):168.1, 155.8, 145.6, 145.3, 143.5, 131.4, 128.3, 126.7, 123.9, 117.7, 67.9, 46.5, 43.3, 26.1; MS (ESI positive) *m*/*z*: 378.1 [M + H]^+^; [α]_D_^22°^ = −20 (*c* = 2.5; Acetone).

#### (*R*)-3-(3-(1-(Pyridin-2-yl)pyrrolidin-3-yl)thioureido)benzenesulfonamide (**36a**)

Following the general procedure, the product was a white solid **36a**, yield 52%. ^**1**^**H NMR** (400 MHz, DMSO-*d*_6_) δ(ppm): 9.66 (1H, bs), 8.26 (1H, s), 8.11 (1H, d, *J* = 3.68 Hz), 8.04 (1H, aps) 7.74 (1H, d, *J* = 7.66 Hz), 7.57–7.49 (3H, m), 7.39 (2H, bs), 6.61 (1H, m), 6.52 (1H, d. *J* = 8.42 Hz), 4.90 (1H, m), 3.75 (1H, m), 3.56–3.45 (3H, m), 2.33 (1H, m), 2.12 (1H, m); ^**13**^**C NMR** (100 MHz, DMSO-*d*_6_) δ(ppm): 181.4, 157.8, 148.8, 145.1, 141.1, 138.0, 134.7, 129.8, 124.3, 121.7, 112.4, 107.3, 54.3, 52.7, 45.6, 31.5; MS (ESI positive) *m*/*z*: 378.1 [M + H]^+^; Elemental analysis: calculated: C, 50.91; H, 5.07; N, 18.55; O, 8.48; S, 16.99 found: C, 49.73; H, 5.06; N, 18.36.

#### (*R*)-4-(3-(1-(6-Nitropyridin-2-yl)pyrrolidin-3-yl)thioureido)benzenesulfonamide (**37a**)

Following the general procedure, the product was a yellow solid **37a**, yield 56%. ^**1**^**H NMR** (400 MHz, DMSO-*d*_6_) δ(ppm): 9.79 (1H, bs), 8.47 (1H, dd, *J* = 4.46, 1.43 Hz), 8.40 (1H, bs), 8.26 (1H, dd, *J* = 8.04, 1.42 Hz), 7.76 (2H, d, *J* = 8.73 Hz), 7.69 (2H, d, *J* = 8.75 Hz), 7.31 (2H, bs), 6.88 (1H, dd, *J* = 8.04, 4.53 Hz), 4.85 (1H, m), 3.69–3.61 (2H, m), 3.48–3.46 (1H, m), 3.32 (1H, dd, *J* = 11.64, 3.94 Hz), 2.35–2.27 (1H, m), 2.17–2.11 (1H, m); ^**13**^**C NMR** (100 MHz, DMSO-*d*_6_) δ(ppm): 181.3, 153.2, 150.6, 143.6, 139.5, 136.0, 132.5, 127.2, 122.4, 112.9, 54.9, 54.1, 48.1, 30.7; MS (ESI positive) *m*/*z*: 423.1 [M + H]^+^ Elemental analysis: calculated: C, 45.49; H, 4.29; N, 19.89; O, 15.15; S, 15.18 found: C, 45.21; H, 4.28; N, 19.82.

#### (*S*)-4-(3-(1-(6-Nitropyridin-2-yl)pyrrolidin-3-yl)thioureido)benzenesulfonamide (**37b**)

Following the general procedure, the product was a yellow solid **37b**, yield 53%. ^**1**^**H NMR** (400 MHz, DMSO-*d*_6_) δ(ppm): 9.77 (1H, bs), 8.47 (1H, d, *J* = 3.35 Hz), 8.38 (1H, d, *J* = 3.78 Hz), 8.25 (1H, d, *J* = 7.04 Hz), 7.76 (2H, d, *J* = 8.68 Hz), 7.69 (2H, d, *J* = 8.66 Hz), 7.29 (2H, bs), 6.88 (1H, dd, *J* = 8.02, 4.52 Hz), 4.85 (1H, m), 3.69–3.60 (2H, m), 3.50–3.46 (1H, m), 3.30 (1H, m), 2.34–2.29 (1H, m), 2.16–2.14 (1H, m); ^**13**^**C NMR** (100 MHz, DMSO-*d*_6_) δ(ppm): 181.4, 153.2, 150.6, 143.6, 136.4, 132.6, 127.2, 122.6, 122.5, 113.0, 55.0, 54.2, 48.2, 30.8; MS (ESI positive) *m*/*z*: 423.1 [M + H]^+^.

#### (*R*)-3-(3-(1-(6-Nitropyridin-2-yl)pyrrolidin-3-yl)thioureido)benzenesulfonamide (**38a**)

Following the general procedure, the product was a yellow solid **38a**, yield 66%. ^**1**^**H NMR** (400 MHz, DMSO-*d*_6_) δ(ppm): 9.70 (1H, bs), 8.46 (1H, s), 8.25 (2H, m), 8.03 (1H, s), 7.72 (2H, m), 7.56 (2H, m), 7.39 (2H, bs), 6.88 (1H, d, *J* = 4.17 Hz), 4.86 (1H, m), 3.65 (2H, m), 3.49–3.46 (2H, m), 2.31 (1H, m), 2.12 (1H, m); ^**13**^**C NMR** (100 MHz, DMSO-*d*_6_) δ(ppm): 181.7, 153.2, 150.6, 145.2, 141.0, 136.0, 132.5, 130.0, 129.9, 122.5, 121.5, 112.9, 54.9, 54.0, 48.1, 30.8; MS (ESI positive) *m*/*z*: 423.1 [M + H]^+^; [α]_D_^22°^ = −13 (*c* = 4.1; Acetone).

#### (*S*)-3-(3-(1-(6-Nitropyridin-2-yl)pyrrolidin-3-yl)thioureido)benzenesulfonamide (**38b**)

Following the general procedure, the product was a yellow solid **38b**, yield 65%. ^**1**^**H NMR** (400 MHz, DMSO-*d*_6_) δ(ppm): 9.70 (1H, bs), 8.46 (1H, s), 8.24 (2H, m), 8.03 (1H, s), 7.72 (1H, d, *J* = 7.92 Hz), 7.56–7.51 (2H, m), 7.39 (2H, bs), 6.88 (1H, dd, *J* = 7.98, 4.51 Hz), 4.86 (1H, m), 3.69–3.61 (2H, m), 3.51–3.47 (1H, m), 3.33–3.29 (1H, m), 2.34–2.29 (1H, m), 2.14 (1H, m); ^**13**^**C NMR** (100 MHz, DMSO-*d*_6_) δ(ppm): 181.7, 153.2, 150.6, 145.2, 141.0, 136.0, 132.5, 129.8, 126.7, 121.8, 120.5, 112.9, 54.9, 54.0, 48.1, 30.8; MS (ESI positive) *m*/*z*: 423.1 [M + H]^+^; [α]_D_^22°^ = +15 (*c* = 2.3; Acetone).

#### (*R*)-4-(3-(1-(5-(Trifluoromethyl)pyridin-2-yl)pyrrolidin-3-yl)thioureido)benzenesulfonamide (**39a**)

Following the general procedure, the product was a white solid **39a**, yield 71%. ^**1**^**H NMR** (400 MHz, DMSO-*d*_6_) δ(ppm): 9.78 (1H, bs), 8.45 (2H, m), 7.82 (1H, dd, *J* = 8.95, 2.16 Hz), 7.76 (2H, d, *J* = 8.81 Hz), 7.72 (2H, d, *J* = 8.79 Hz), 7.31 (2H, bs), 6.65 (1H, d, *J* = 8.94 Hz), 4.92 (1H, m), 3.85–3.81 (1H, m), 3.61 (3H, m), 2.39–2.34 (1H, m), 2.17–2.10 (1H, m); ^**13**^**C NMR** (100 MHz, DMSO-*d*_6_) δ(ppm): 181.2, 159.2, 146.5, 143.6, 139.5, 134.8, 127.1, 126.1 (q, *J* = 251.52 Hz), 122.4, 113.3 (q, *J* = 32.06 Hz), 107.1, 54.2, 52.8, 45.8, 31.2; ^**19**^**F NMR** (376 MHz, DMSO-*d*_6_) δ(ppm): −59.0; MS (ESI positive) *m*/*z*: 446.1 [M + H]^+^; [α]_D_^22°^ = −45 (*c* = 5.1; Acetone); Elemental analysis: calculated: C, 45.84; H, 4.07; F, 12.79; N, 15.72; O, 7.18; S, 14.39 found: C, 45.73; H, 4.06; F, 12.71; N, 15.68.

#### (*S*)-4-(3-(1-(5-(Trifluoromethyl)pyridin-2-yl)pyrrolidin-3-yl)thioureido)benzenesulfonamide (**39b**)

Following the general procedure, the product was a white solid **39b**, yield 60%. ^**1**^**H NMR** (400 MHz, DMSO-*d*_6_) δ(ppm): 9.76 (1H, bs), 8.45 (1H, s), 8.40 (1H, d, *J* = 3.67 Hz), 7.81 (1H, d, *J* = 7.85 Hz), 7.76 (2H, d, *J* = 8.57 Hz), 7.72 (2H, d, *J* = 8.55 Hz), 7.30 (2H, bs), 6.65 (1H, d, *J* = 8.90 Hz), 4.92 (1H, m), 3.86–3.82 (1H, m), 3.61 (3H, m), 2.39–2.34 (1H, m), 2.17–2.14 (1H, m); ^**13**^**C NMR** (100 MHz, DMSO-*d*_6_) δ(ppm): 181.2, 159.2, 146.5, 143.6, 139.4, 134.8, 127.1, 126.1 (q, *J* = 269.91 Hz), 122.4, 113.3 (q, *J* = 32.25 Hz), 107.1, 54.2, 52.8, 45.8, 31.2; MS (ESI positive) *m*/*z*: 446.1 [M + H]^+^; [α]_D_^22°^ = +40 (*c* = 1.9; Acetone) Elemental analysis: calculated: C, 45.84; H, 4.07; F, 12.79; N, 15.72; O, 7.18; S, 14.39 found: C, 45.74; H, 4.06; F, 12.73; N, 15.71.

#### (*R*)-3-(3-(1-(5-(Trifluoromethyl)pyridin-2-yl)pyrrolidin-3-yl)thioureido)benzenesulfonamide (**40a**)

Following the general procedure, the product was a white solid **40a**, yield 61%. ^**1**^**H NMR** (400 MHz, DMSO-*d*_6_) δ(ppm): 9.69 (1H, bs), 8.45 (1H, s), 8.31 (1H, s), 8.04 (1H, s), 7.82 (1H, dd, *J* = 8.94, 2.11 Hz), 7.73 (1H, d, *J* = 7.63 Hz), 7.57–7.50 (2H. m), 7.40 (2H, bs), 6.66 (1H, d, *J* = 8.95 Hz), 4.92 (1H, m), 3.86–3.82 (1H, m), 3.62–3.57 (2H, m), 3.45–3.38 (1H, m), 2.40–2.32 (1H, m), 2.17–2.12 (1H, m); ^**13**^**C NMR** (100 MHz, DMSO-*d*_6_) δ(ppm): 181.5, 159.2, 146.5, 145.1, 141.0, 134.8, 129.8, 126.5, 126.1 (q, *J* = 270.0 Hz), 121.7, 120.4, 113.3 (q, *J* = 32.16 Hz), 107.1, 54.2, 52.8, 45.8, 31.2; ^**19**^**F NMR** (376 MHz, DMSO-*d*_6_) δ(ppm): −59.0; MS (ESI positive) *m*/*z*: 446.1 [M + H]^+^; [α]_D_^22°^ = −55 (*c* = 5.9; Acetone).

#### (*S*)-3-(3-(1-(5-(Trifluoromethyl)pyridin-2-yl)pyrrolidin-3-yl)thioureido)benzenesulfonamide (**40b**)

Following the general procedure, the product was a white solid **40b**, yield 60%. ^**1**^**H NMR** (400 MHz, DMSO-*d*_6_) δ(ppm): 9.68 (1H, bs), 8.45 (1H, s), 8.30 (1H, s), 8.04 (1H, s), 7.81 (1H, dd, *J* = 8.94, 2.16 Hz), 7.73 (1H, d, *J* = 7.70 Hz), 7.57–7.50 (2H. m), 7.40 (2H, bs), 6.66 (1H, d, *J* = 8.95 Hz), 4.92 (1H, m), 3.86–3.82 (1H, m), 3.62–3.57 (3H, m), 2.41–2.32 (1H, m), 2.17–2.12 (1H, m); ^**13**^**C NMR** (100 MHz, DMSO-*d*_6_) δ(ppm): 181.5, 159.2, 146.5, 145.2, 141.0, 134.9, 129.9, 127.4, 126.1 (q, *J* = 269.94 Hz), 121.8, 120.5, 113.3 (q, *J* = 33.93 Hz), 107.1, 54.2, 52.8, 45.8, 31.3; MS (ESI positive) *m*/*z*: 446.1 [M + H]^+^; [α]_D_^22°^ = +50 (*c* = 6.1; Acetone).

### General Synthesis of Compounds **41–46**

A mixture of corresponding carbamate (**34a-b**, 1 equiv) and corresponding amine **30–32** (1 equiv) in acetonitrile was stirred at reflux overnight. Then, water was added and the precipitate was filtered off.

#### (*R*)-4-(3-(1-(Pyridin-2-yl)pyrrolidin-3-yl)ureido)benzenesulfonamide (**41a**)

Following the general procedure, the product was a white solid **41a**, yield 51%. ^**1**^**H NMR** (400 MHz, DMSO-*d*_6_) δ(ppm): 8.77 (1H, s), 8.11 (1H, d, *J* = 3.84 Hz), 7.71 (2H, d, *J* = 8.70 Hz), 7.56 (2H, d, *J* = 8.79 Hz), 7.53–7.20 (1H, m), 7.20 (2H, bs), 6.72 (1H, d, *J* = 6.80 Hz), 6.59 (1H, m), 6.50 (1H, d, *J* = 8.48 Hz), 4.37 (1H, m), 3.67–3.63 (1H, m), 3.51–3.49 (3H, m), 2.27–2.22 (1H, m), 1.99–1.95 (1H, m); ^**13**^**C NMR** (100 MHz, DMSO-*d*_6_) δ(ppm):157.9, 155.4, 148.8, 144.3, 137.9, 137.1, 127.7, 117.7, 112.3, 107.2, 53.3, 50.1, 45.4, 32.1; MS (ESI positive) *m*/*z*: 362.1 [M + H]^+^.

#### (*R*)-3-(3-(1-(Pyridin-2-yl)pyrrolidin-3-yl)ureido)benzenesulfonamide (**42a**)

Following the general procedure, the product was a white solid **42a**, yield 51%. ^**1**^**H NMR** (400 MHz, DMSO-*d*_6_) δ(ppm): 8.71 (1H, s), 8.11 (1H, d, *J* = 4.47 Hz), 8.04 (1H, s), 7.52–7.50 (2H, m), 7.44–7.40 (2H, m), 7.35 (2H, bs), 6.63 (1H, d, *J* = 6.84 Hz), 6.59 (1H, m), 6.50 (1H, d, *J* = 8.46 Hz), 4.37 (1H, m), 3.66–3.64 (1H, m), 3.51–3.49 (2H, m), 3.33 (1H, m), 2.29–2.21 (1H, m), 2.01–1.94 (1H, m); ^**13**^**C NMR** (100 MHz, DMSO-*d*_6_) δ(ppm): 155.6, 148.8, 145.5, 141.7, 137.9, 136.8, 130.2, 121.4, 119.1, 115.4, 112.3, 107.3, 53.3, 50.1, 45.5, 32.1; MS (ESI positive) *m*/*z*: 362.1 [M + H]^+^; [α]_D_^22°^ = −49 (*c* = 2.3; Acetone).

#### (*R*)-4-(3-(1-(6-Nitropyridin-2-yl)pyrrolidin-3-yl)ureido)benzenesulfonamide (**43a**)

Following the general procedure, the product was a yellow solid **43a**, yield 63%. ^**1**^**H NMR** (400 MHz, DMSO-*d*_6_) δ(ppm): 8.79 (1H, s), 8.46 (1H, s), 8.25 (1H, m), 7.70 (2H, d, *J* = 8.70 Hz), 7.56 (2H, d, *J* = 8.79 Hz), 7.20 (2H, bs), 6.88 (1H, d, *J* = 4.16 Hz), 6.74 (1H, s), 4.34 (1H, m), 3.58 (2H, m), 3.46 (1H, m), 3.17 (1H, m), 2.22 (1H, m), 1.99 (1H, m); ^**13**^**C NMR** (100 MHz, DMSO-*d*_6_) δ(ppm): 155.5, 153.2, 150.6, 144.2, 137.2, 135.9, 132.4, 127.8, 112.8, 55.6, 49.9, 48.0, 31.3; MS (ESI positive) *m*/*z*: 407.1 [M + H]^+^; [α]_D_^22°^ = −18 (*c* = 1.7; Acetone) Elemental analysis: calculated: C, 47.29; H, 4.46; N, 20.68; O, 19.68; S, 7.89; found: C, 47.21; H, 4.45; N, 20.62.

#### (*S*)-4-(3-(1-(6-Nitropyridin-2-yl)pyrrolidin-3-yl)ureido)benzenesulfonamide (**43b**)

Following the general procedure, the product was a yellow solid **43b**, yield 98%. ^**1**^**H NMR** (400 MHz, DMSO-*d*_6_) δ(ppm): 8.77 (1H, s), 8.46 (1H, dd, *J* = 4.40, 1.38 Hz), 8.25 (1H, dd, *J* = 8.02, 1.36 Hz), 7.70 (2H, d, *J* = 8.72 Hz), 7.56 (2H, d, *J* = 8.76 Hz), 7.16 (2H, bs), 6.87 (1H, dd, *J* = 8.02, 4.52 Hz), 6.72 (1H, d, *J* = 6.37 Hz), 4.34 (1H, m), 3.60 (2H, m), 3.55 (1H, m), 3.18 (1H, dd, *J* = 11.46, 4.06 Hz), 2.26–2.18 (1H, m), 2.03–1.95 (1H, m); ^**13**^**C NMR** (100 MHz, DMSO-*d*_6_) δ(ppm): 155.5, 153.1, 150.6, 144.2, 137.2, 135.9, 127.8, 122.1, 117.8, 112.8, 55.6, 49.9, 47.9, 31.3; MS (ESI positive) *m*/*z*: 407.1 [M + H]^+^; [α]_D_^22°^ = +16 (*c* = 1.9; Acetone).

#### (*R*)-3-(3-(1-(6-Nitropyridin-2-yl)pyrrolidin-3-yl)ureido)benzenesulfonamide (**44a**)

Following the general procedure, the product was a yellow solid **44a**, yield 52%. ^**1**^**H NMR** (400 MHz, DMSO-*d*_6_) δ(ppm): 8.74 (1H, s), 8.46 (1H, s), 8.25 (1H, m), 8.03 (1H, s), 7.50 (1H,m), 7.43–7.39 (2H, m), 7.33 (2H, bs), 6.87 (1H, s), 6.66 (1H, s), 4.33 (1H, m), 3.58 (2H, m), 3.46 (1H, m), 3.19–3.16 (1H, m), 2.22 (1H, m), 2.02 (1H, m); ^**13**^**C NMR** (100 MHz, DMSO-*d*_6_) δ(ppm): 155.7, 153.2, 150.6, 145.5, 141.6, 136.0, 132.4, 130.2, 121.5, 119.2, 115.6, 112.9, 55.6, 50.0, 48.1, 31.3; MS (ESI positive) *m*/*z*: 407.1 [M + H]^+^; [α]_D_^22°^ = −43 (*c* = 5.1; Acetone).

#### (*S*)-3-(3-(1-(6-Nitropyridin-2-yl)pyrrolidin-3-yl)ureido)benzenesulfonamide (**44b**)

Following the general procedure, the product was a yellow solid **44b**, yield 50%. ^**1**^**H NMR** (400 MHz, DMSO-*d*_6_) δ(ppm): 8.72 (1H, s), 8.46 (1H, d, *J* = 1.58 Hz), 8.25 (1H, d, *J* = 7.98 Hz), 8.03 (1H, s), 7.51 (1H,m), 7.44–7.40 (2H, m), 7.32 (2H, bs), 6.87 (1H, m), 6.64 (1H, d, *J* = 5.93 Hz), 4.34 (1H, m), 3.62–3.54 (2H, m), 3.46 (1H, m), 3.19–3.17 (1H, m), 2.25–2.20 (1H, m), 2.02–1.96 (1H, m); ^**13**^**C NMR** (100 MHz, DMSO-*d*_6_) δ(ppm): 155.6, 153.1, 150.6, 145.5, 141.5, 135.9, 132.4, 130.1, 121.5, 119.2, 115.5, 112.8, 55.6, 50.0, 48.0, 31.3; MS (ESI positive) *m*/*z*: 407.1 [M + H]^+^; [α]_D_^22°^ = +40 (*c* = 5.5; Acetone).

#### (*R*)-4-(3-(1-(5-(Trifluoromethyl)pyridin-2-yl)pyrrolidin-3-yl)ureido)benzenesulfonamide (**45a**)

Following the general procedure, the product was a white solid **45a**, yield 78%. ^**1**^**H NMR** (400 MHz, DMSO-*d*_6_) δ(ppm): 8.78 (1H, bs), 8.44 (1H, s), 7.80 (1H, dd, *J* = 8.96, 2.16 Hz), 7.71 (2H, d, *J* = 8.65 Hz), 7.57 (2H. d, *J* = 8.70 Hz), 7.22 (2H, bs), 6.75 (1H, d, *J* = 6.72 Hz), 6.64 (1H, d, *J* = 8.95 Hz), 4.39 (1H, m), 3.75–3.71 (1H, m), 3.59 (2H, m), 3.44 (1H, m), 2.30–2.25 (1H, m), 2.03–1.99 (1H, m); ^**13**^**C NMR** (100 MHz, DMSO-*d*_6_) δ(ppm): 159.3, 155.5, 146.5, 144.3, 137.2, 134.9, 137.2, 134.9, 127.7, 126.1 (q, *J* = 268.0 Hz), 117.8, 113.3 (q, *J* = 32.18 Hz), 107.1, 53.4, 50.1, 45.8, 31.9; MS (ESI positive) *m*/*z*: 430.1 [M + H]^+^; [α]_D_^22°^ = −26 (*c* = 2.0; Acetone).

#### (*S*)-4-(3-(1-(5-(Trifluoromethyl)pyridin-2-yl)pyrrolidin-3-yl)ureido)benzenesulfonamide (**45b**)

Following the general procedure, the product was a white solid **45b**, yield 50%. ^**1**^**H NMR** (400 MHz, DMSO-*d*_6_) δ(ppm): 8.77 (1H, bs), 8.44 (1H, s), 7.80 (1H, d, *J* = 8.16 Hz), 7.72 (2H, d, *J* = 8.43 Hz), 7.57 (2H. d, *J* = 8.45 Hz), 7.20 (2H, bs), 6.74 (1H, d, *J* = 6.41 Hz), 6.64 (1H, d, *J* = 8.87 Hz), 4.39 (1H, m), 3.76–3.72 (1H, m), 3.59 (2H, m), 3.44 (1H, m), 2.30–2.25 (1H, m), 2.03–2.00 (1H, m); ^**13**^**C NMR** (100 MHz, DMSO-*d*_6_) δ(ppm): 159.3, 155.4, 146.5, 144.2, 137.2, 134.8, 130.1, 127.7, 126.1 (q, *J* = 270.81 Hz), 117.8, 113.2 (q, *J* = 32.35 Hz), 107.0, 53.4, 50.0, 45.7, 31.8; MS (ESI positive) *m*/*z*: 430.1 [M + H]^+^; [α]_D_^22°^ = +30 (*c* = 3.0; Acetone).

#### (*R*)-3-(3-(1-(5-(Trifluoromethyl)pyridin-2-yl)pyrrolidin-3-yl)ureido)benzenesulfonamide (**46a**)

Following the general procedure, the product was a white solid **46a**, yield 61%. ^**1**^**H NMR** (400 MHz, DMSO-*d*_6_) δ(ppm): 8.73 (1H, bs), 8.44 (1H, s), 8.05 (1H, s), 7.80 (1H, d, *J* = 8.17 Hz), 7.53–7.36 (4H, m), 6.66 (1H, t, *J* = 8.62 Hz), 4.38 (1H, m), 3.76 (1H, m), 3.58 (2H, m), 3.41 (1H, m), 2.28–2.25 (1H, m), 2.02–2.01 (1H, m); ^**13**^**C NMR** (100 MHz, DMSO-*d*_6_) δ(ppm): 159.3, 155.7, 146.5, 145.5, 141.6, 134.9, 130.2, 126.2 (q, *J* = 270.01 Hz), 121.5, 119.2, 115.5, 113.3 (q, *J* = 32.31 Hz), 107.1, 53.4, 50.1, 45.8, 31.9; MS (ESI positive) *m*/*z*: 430.1 [M + H]^+^; [α]_D_^22°^ = −32 (*c* = 4.3; Acetone).

#### (*S*)-3-(3-(1-(5-(Trifluoromethyl)pyridin-2-yl)pyrrolidin-3-yl)ureido)benzenesulfonamide (**46b**)

Following the general procedure, the product was a white solid **46b**, yield 62%. ^**1**^**H NMR** (400 MHz, DMSO-*d*_6_) δ(ppm): 8.71 (1H, bs), 8.44 (1H, s), 8.04 (1H, s), 7.80 (1H, dd, *J* = 8.92, 2.08 Hz), 7.52 (1H, d, *J* = 7.93 Hz), 7.42–7.34 (2H, m), 7.34 (2H, bs), 6.66–6.64 (2H, m), 4.39 (1H, m), 3.76–3.73 (1H, m), 3.59 (2H, m), 3.41 (1H, m), 2.32–2.25 (1H, m), 2.04–1.99 (1H, m); ^**13**^**C NMR** (100 MHz, DMSO-*d*_6_) δ(ppm): 159.4, 155.7, 146.6, 145.6, 141.7, 134.9, 130.3, 126.2 (q, *J* = 269.76 Hz), 121.5, 119.2, 115.5, 113.3 (q, *J* = 32.00 Hz), 107.2, 53.5, 50.2, 45.9, 31.9; MS (ESI positive) *m*/*z*: 430.1 [M + H]^+^; [α]_D_^22°^ = +37 (*c* = 2.3; Acetone).

### Carbonic Anhydrase Inhibition

An Applied Photophysics stopped-flow instrument was used to assay the CA-catalyzed CO_2_ hydration activity.^31^ Phenol red (at a concentration of 0.2 mM) was used as an indicator, working at the absorbance maximum of 557 nm, with 20 mM Hepes (pH 7.4) as a buffer, and 20 mM Na_2_SO_4_ (to maintain constant ionic strength), following the initial rates of the CA-catalyzed CO_2_ hydration reaction for a period of 10–100 s. The CO_2_ concentrations ranged from 1.7 to 17 mM for the determination of the kinetic parameters and inhibition constants.^36^ Enzyme concentrations ranged between 5 and 12 nM. For each inhibitor, at least six traces of the initial 5–10% of the reaction were used to determine the initial velocity. The uncatalyzed rates were determined in the same manner and subtracted from the total observed rates. Stock solutions of the inhibitor (0.1 mM) were prepared in distilled–deionized water, and dilutions up to 0.01 nM were done thereafter with the assay buffer. Inhibitor and enzyme solutions were preincubated together for 15 min at room temperature prior to the assay, to allow for the formation of the E–I complex. The inhibition constants were obtained by nonlinear least-squares methods using PRISM 3 and the Cheng-Prusoff equation as reported earlier and represent the mean from at least three different determinations. All CA isoforms were recombinant proteins obtained in-house, as reported earlier.^37−39^

### TRPV1 Assay

SH-SY5Y-TRPV1 cells (kindly provided by Johanna Lilja and Anna Forsby (University of Stockholm, Stockholm, Sweden))^40^ were cultured in 96 wells. Upon 90% confluence had been reached, the cells were loaded with 5 μM Fluo-4NW for 1 h at 37 °C. The Fluo-4NW fluorescence was measured through cycles (1.7 min each) of excitation at 485 nm and emission at 535 nm (POLARstar Omega BMG LABtech). Briefly, after measuring the basal fluorescence of the plate (four cycles), testing compounds were added to the plate, and the fluorescence was measured for additional 10 cycles; thereafter, 10 μM capsaicin was added with a microinjector and the fluorescence was monitored for an additional 10 cycles. Under these conditions, testing agonists and antagonists were exposed to the cells for 25 min.

### Crystallization and X-ray Data Collection

Crystals of hCAII were obtained using the hanging drop vapor diffusion method using a 24-well Linbro plate. A 10 mg/mL solution of hCA II (2 μL) in Tris-HCl 20 mM pH 8.0 was mixed with 2 μL of a solution of 1.5 M sodium citrate and 0.1 M Tris pH 8.0 and was equilibrated against the same solution at 296 K. The complexes were prepared by soaking the hCA II native crystals in the mother liquor solution containing the inhibitors at a concentration of 10 mM for 2 days. All crystals were flash-frozen at 100 K using a solution obtained by adding 15% (v/v) glycerol to the mother liquor solution as a cryoprotectant. Data on crystals of the complexes were collected using synchrotron radiation at the XRD2 beamline at Elettra Synchrotron (Trieste, Italy) with a wavelength of 1.000 Å and a DECTRIS Pilatus 6 M detector. Data were integrated and scaled using the program energy-dispersive X-ray spectrum (XDS).^41^ Data processing statistics are shown in the Supporting Information.

### Structure Determination

The crystal structure of hCA II (PDB accession code: 4FIK) without solvent molecules and other heteroatoms was used to obtain initial phases using Refmac5;^42^ 5% of the unique reflections were selected randomly and excluded from the refinement data set for the purpose of *R*_free_ calculations. The initial |*F*_o_ – *F*_c_| difference electron density maps unambiguously showed the inhibitor molecules. The inhibitor was introduced in the model with 1.0 occupancy. Refinements proceeded using normal protocols of positional, isotropic atomic displacement parameters alternating with manual building of the models using COOT.^43^ The quality of the final models was assessed with COOT and RAMPAGE.^44^ Crystal parameters and refinement data are summarized in the Supporting Information (SI). Atomic coordinates were deposited in the Protein Data Bank (PDB) accession code: 8BJX; 8BOE. Graphical representations were generated with Chimera.^45^

### *In Vivo* Experiment

#### Animals

Male CD-1 albino mice (Envigo, Varese, Italy) weighing approximately 22–25 g at the beginning of the experimental procedure were used. The animals were housed in Ce.S.A.L (Centro Stabulazione Animali da Laboratorio, University of Florence) and used at least 1 week after their arrival. Ten mice were housed per cage (size 26 × 41 cm). The animals were fed a standard laboratory diet and tap water ad libitum and kept at 23 ± 1 °C with a 12 h light/dark cycle, light at 7 a.m. All animal manipulations were carried out according to the Directive 2010/63/EU of the European Parliament and of the European Union council (22 September 2010) on the protection of animals used for scientific purposes. The ethical policy of the University of Florence complies with the Guide for the Care and Use of Laboratory Animals of the US National Institutes of Health (NIH Publication No. 85-23, revised 1996; University of Florence assurance number: A5278-01). Formal approval to conduct the experiments described was obtained from the Animal Subjects Review Board of the University of Florence. Experiments involving animals have been reported according to ARRIVE guideline.^46^ All efforts were made to minimize animal suffering and to reduce the number of animals used. Protocol number of ethical assessment 229/2020-PR.

#### Oxaliplatin-Induced Neuropathic Pain Model and Pharmacological Treatments

Mice treated with oxaliplatin (2.4 mg/kg) were administered intraperitoneally (i.p.) on days 1–2, 5–9, and 12–14 (10 i.p. injections) according to Cavaletti and colleagues^47^ with minor modifications concerning the days of oxaliplatin administration.^48^ Oxaliplatin was dissolved in 5% glucose solution. Control animals received an equivalent volume of vehicle. Behavioral tests were performed starting from day 15 when neuropathy was well established. Compounds **12a**, **37a**, **39a**, and **39b** were suspended in 1% carboxymethylcellulose sodium salt (CMC; Sigma-Aldrich, Milan, Italy) and per os (p.o.) acutely administered in a range dose of 10–100 mg/kg. Behavioral tests were carried out before and after (15, 30, 45, and 60 min) compound’s injection.

#### Cold Plate Test

Thermal allodynia was assessed using the cold plate test. With minimal animal-handler interaction, mice were taken from home cages and placed onto the surface of the cold plate (Ugo Basile, Varese, Italy) maintained at a constant temperature of 4 ± 1 °C. Ambulation was restricted by a cylindrical Plexiglas chamber (diameter: 10 cm, height: 15 cm), with open top. A timer controlled by foot peddle began timing response latency from the moment the mouse was placed onto the cold plate. Pain-related behavior (licking of the hind paw) was observed, and the time (seconds) of the first sign was recorded. The cutoff time of the latency of paw lifting or licking was set at 30 s.

#### Statistical Analysis

Behavioral measurements were performed on 12 mice for each treatment carried out in two different experimental sets. Results were expressed as mean ± S.E.M. The analysis of variance of behavioral data was performed by one-way ANOVA, and a Bonferroni’s significant difference procedure was used as post hoc comparison. *P* values of less than 0.05 or 0.01 were considered significant. Investigators were blind to all experimental procedures. Data were analyzed using the “Origin 9” software (OriginLab, Northampton).

## Supporting Information Available

^1^H, ^13^C, and ^19^F NMR spectra of compounds; summary of data collection and atomic model; and refinement statistics for hCAII (Figure S1) (PDF)
Molecular formula strings (CSV)

## Abbreviations Used

AAZ: acetazolamide
CA: Carbonic Anhydrase
CAI: Carbonic Anhydrase Inhibitors
CNS: central nervous system
DRG: dorsal root ganglion
NSAIDs: nonsteroidal anti-inflammatory drugs
OINP: oxaliplatin-induced neuropathy
TLC: thin-layer chromatography
TRPV1: Transient Receptor Potential Vanilloid 1
