# Supplementary material for: First-in-Class Dual Hybrid Carbonic Anhydrase Inhibitors and Transient Receptor Potential Vanilloid 1 Agonists Revert Oxaliplatin-Induced Neuropathy
Source: J Med Chem. 2023 Jan 10;66(2):1616–33. doi: 10.1021/acs.jmedchem.2c01911 (PMC9940855; doi:10.1021/acs.jmedchem.2c01911)
Supplement: Supplementary file 1 — jm2c01911_si_001.pdf [file jm2c01911_si_001.pdf]

Supplementary Material for

First in Class Dual Hybrid Carbonic Anhydrase Inhibitors and Transient Receptor Potential Vanilloid 1 Agonists Revert Oxaliplatin-Induced Neuropathy

Andrea Angeli[1*], Laura Micheli[2], Fabrizio Carta[1], Marta Ferraroni[3], Tracey Pirali[4], Asia Fernandez Carvajal[5], Antonio Ferrer Montiel[5], Lorenzo Di Cesare Mannelli[2], Carla Ghelardini[2], Claudiu T. Supuran[1]

[1] NEUROFARBA Department, Sezione di Scienze Farmaceutiche, University of Florence, Via Ugo Schiff 6, 50019 Sesto Fiorentino, Florence, Italy

[2] Pharmacology and Toxicology Section, Department of Neuroscience, Psychology, Drug Research and Child Health (NEUROFARBA), University of Florence, viale Gaetano Pieraccini 6, Firenze, 50139 Florence, Italy.

[3] Department of Chemistry "Ugo Schiff", University of Florence, Via della Lastruccia 3-13, I-50019, Sesto Fiorentino, Italy

[4] Dipartimento Di Scienze del Farmaco, Università Degli Studi del Piemonte Orientale, 28100, Novara, Italy.

[5] Instituto de Investigación, Desarrollo e Innovación en Biotecnología Sanitaria de Elche (IDiBE), Universitas Miguel Hernández, 03202, Elche, Spain.

*Corresponding author: Andrea Angeli (andrea.angeli@unifi.it)

Index

^{1}H, ^{13}C, ^{19}F Spectra of compounds *S2-78*

Summary of Data Collection and Atomic Model Refinement Statistics for hCAII *S79*

Figure S1 *S80*

Copies of NMR Spectrum of synthesized compounds

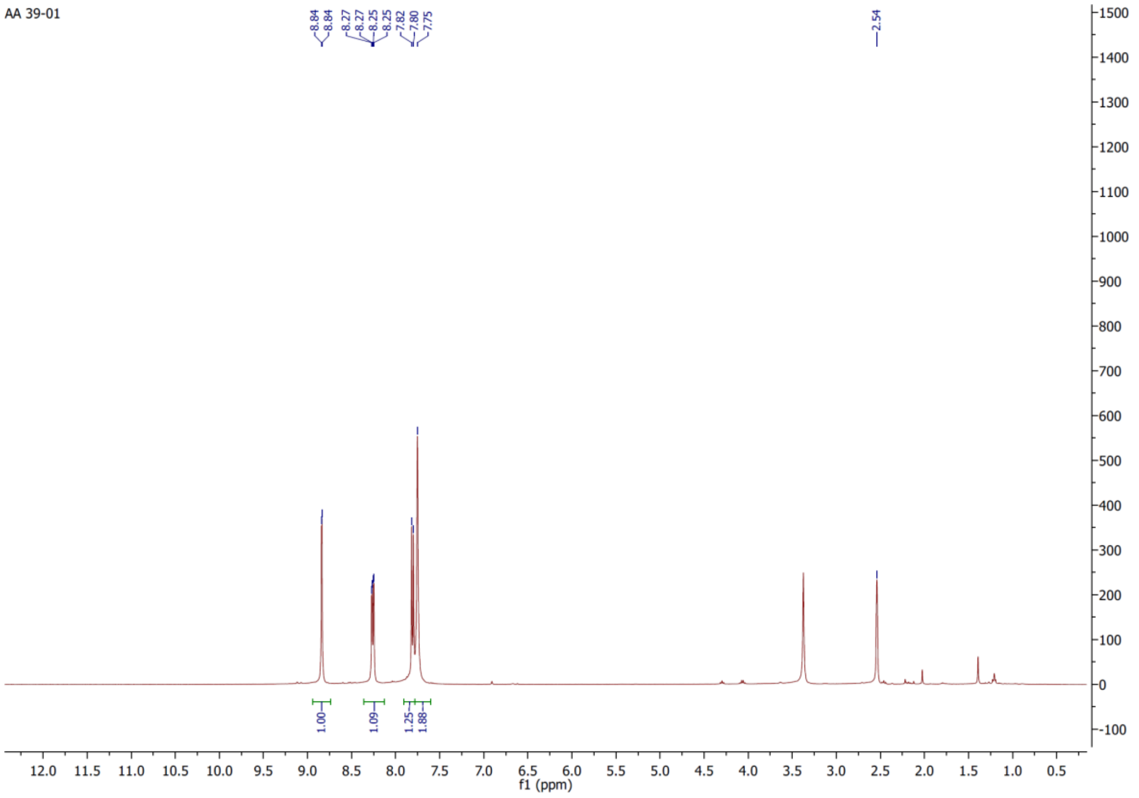

^{1}H NMR spectrum of compound **2** (400 MHz, DMSO-d_6)

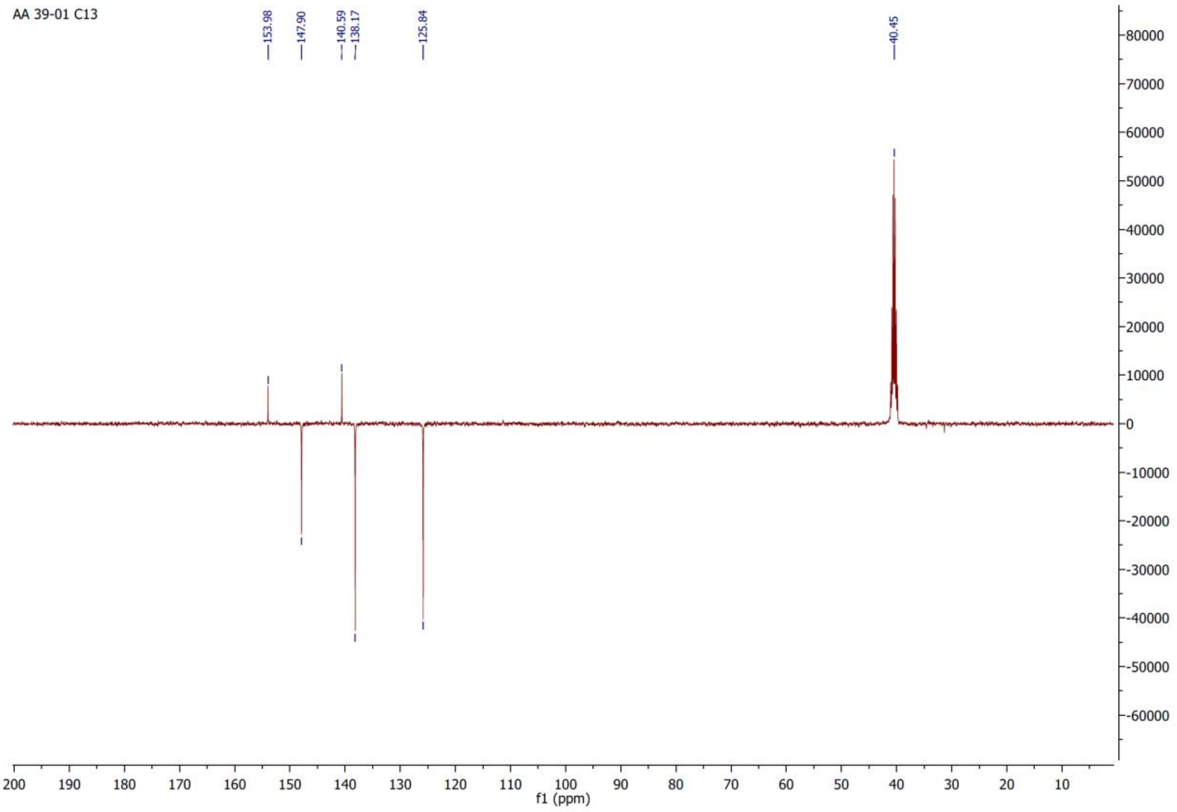

^{13}C NMR spectrum of compound **2** (100 MHz, DMSO-d_6)

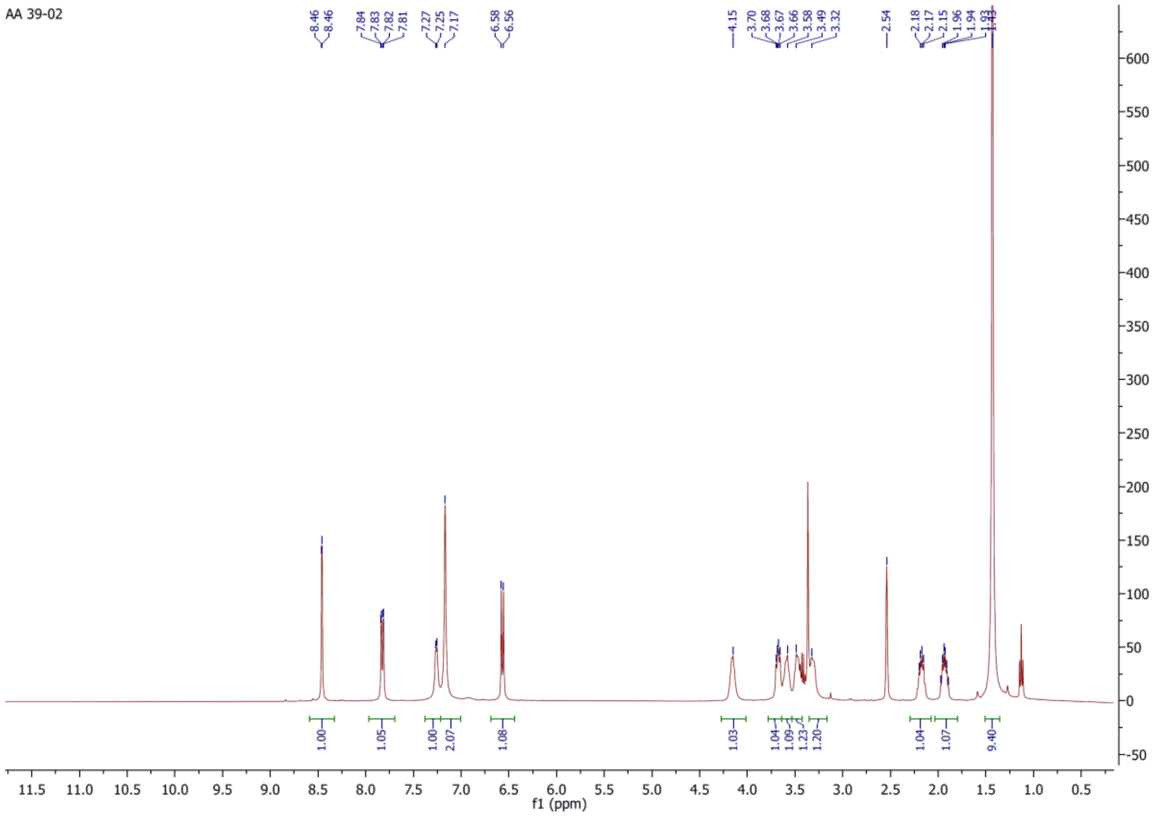

^{1}H NMR spectrum of compound **4a** (400 MHz, DMSO-d_6)

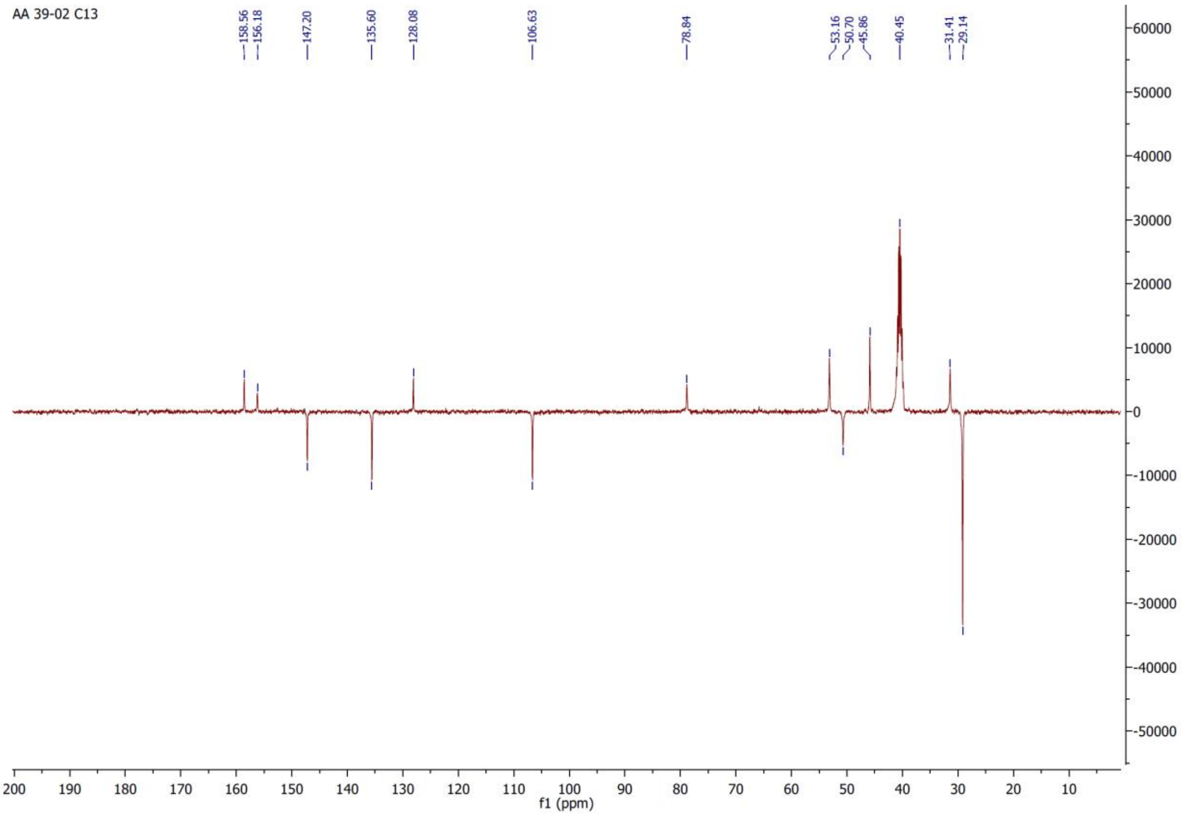

^{13}C NMR spectrum of compound **4a** (100 MHz, DMSO-d_6)

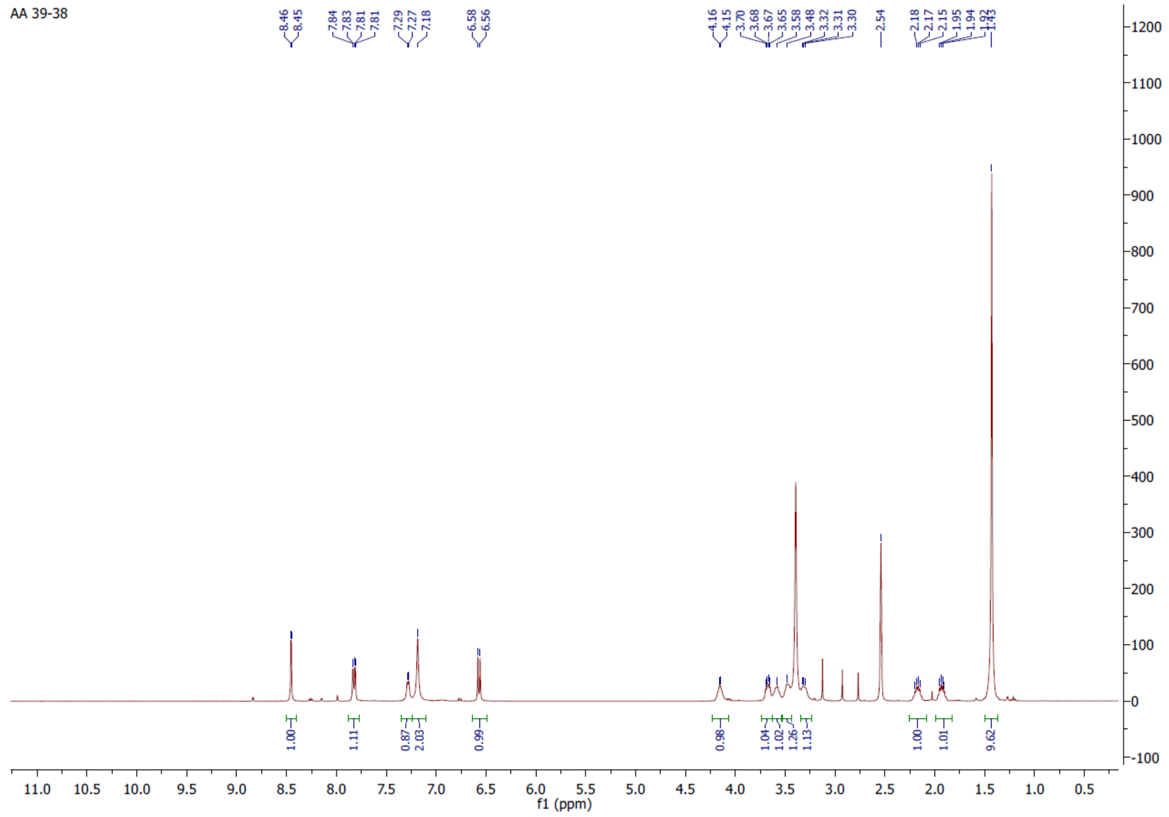

^{1}H NMR spectrum of compound **4b** (400 MHz, DMSO-d_6)

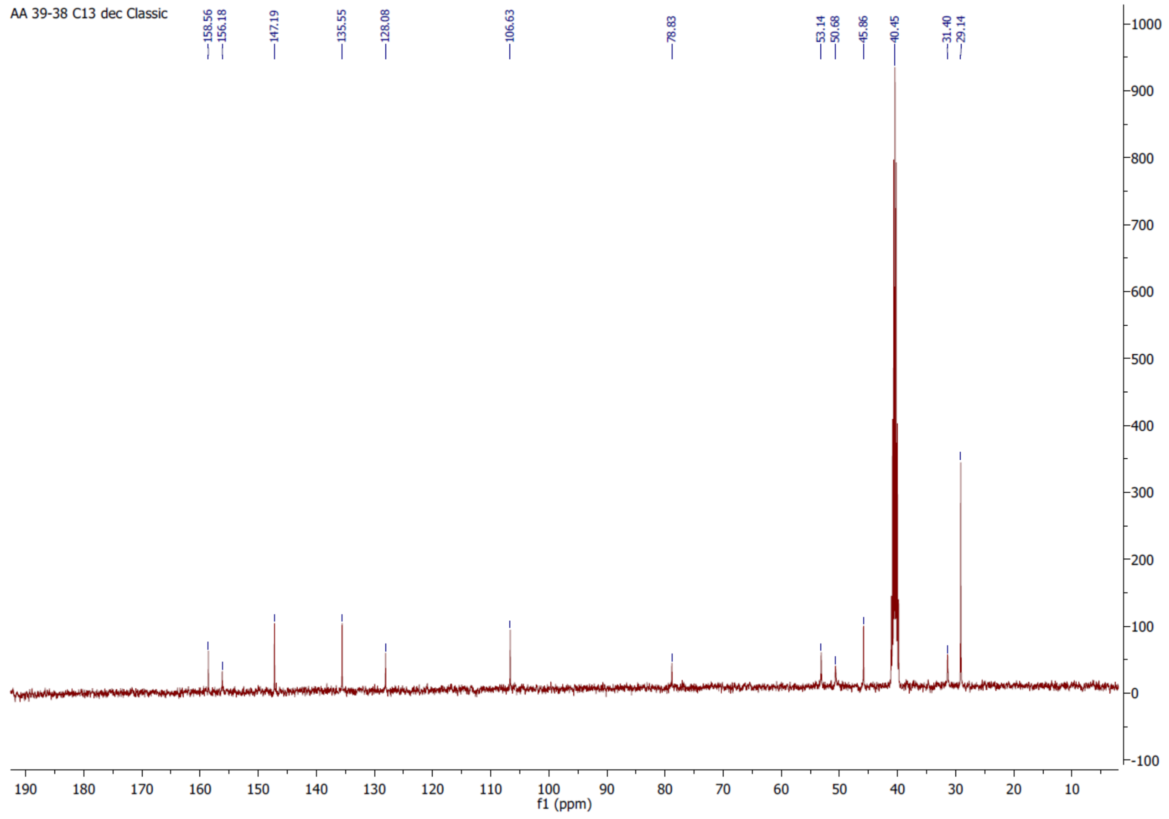

^{13}C NMR spectrum of compound **4b** (100 MHz, DMSO-d_6)

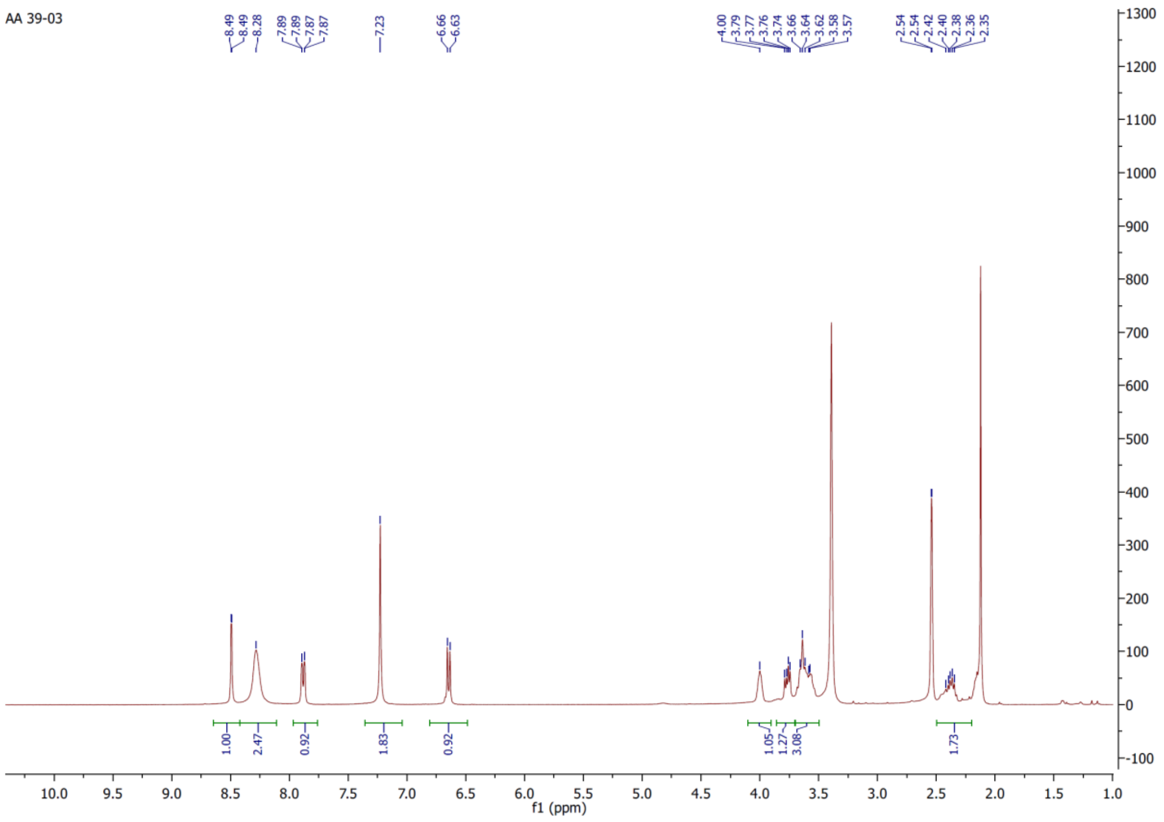

^{1}H NMR spectrum of compound **5a** (400 MHz, DMSO-d_6)

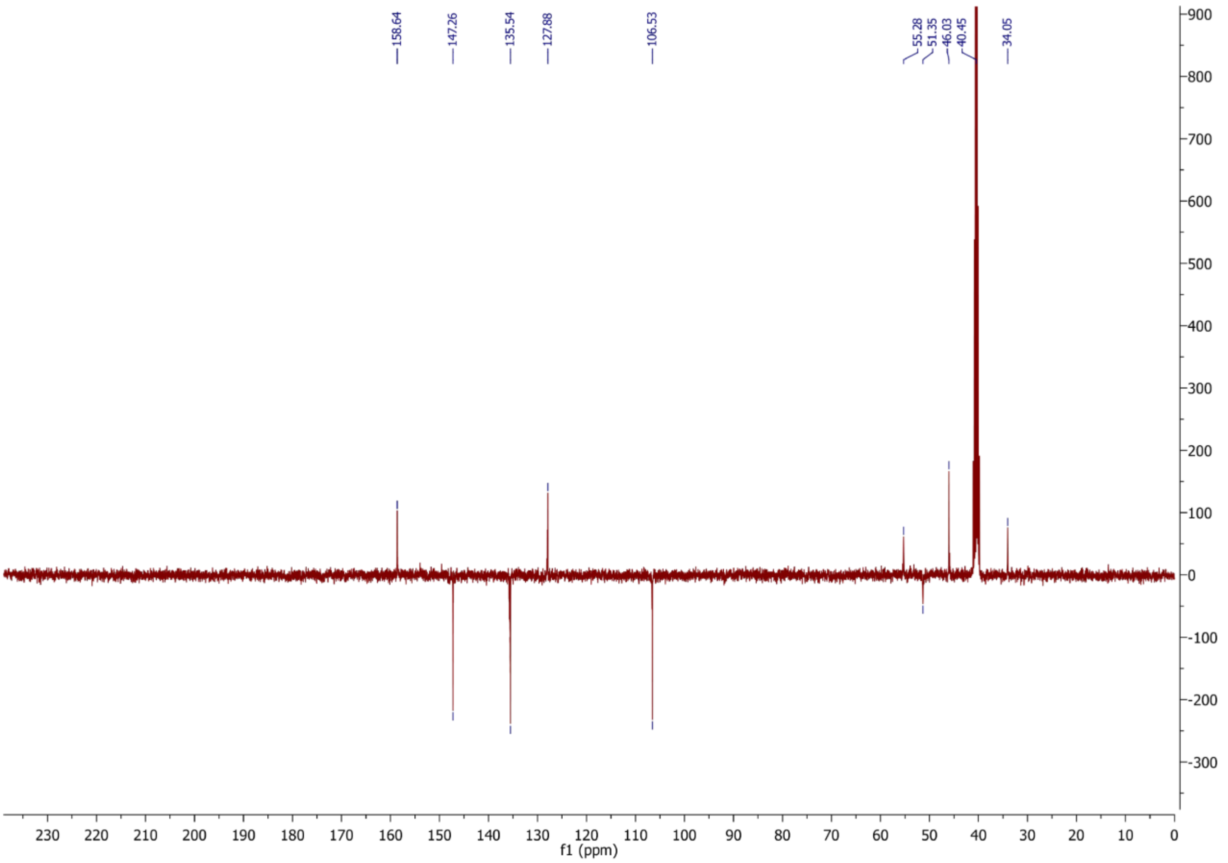

^{13}C NMR spectrum of compound **5a** (100 MHz, DMSO-d_6)

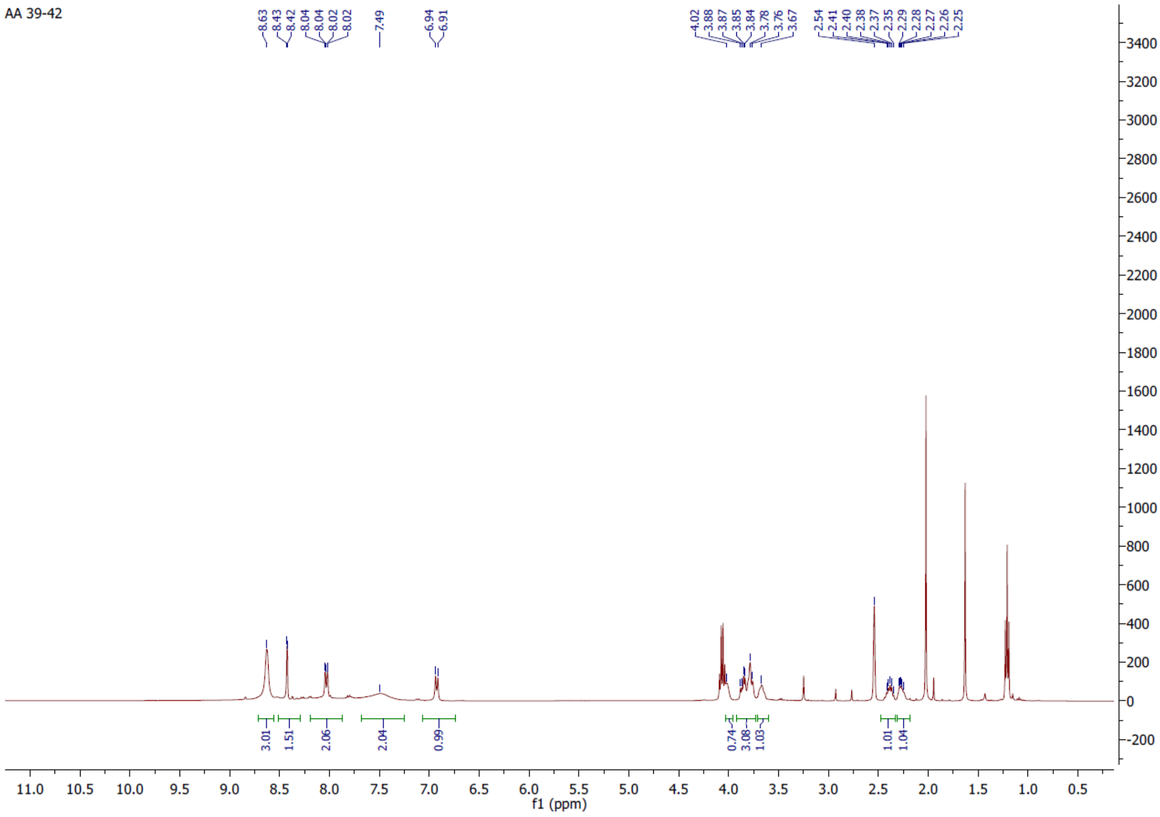

^{1}H NMR spectrum of compound **5b** (400 MHz, DMSO-d_6)

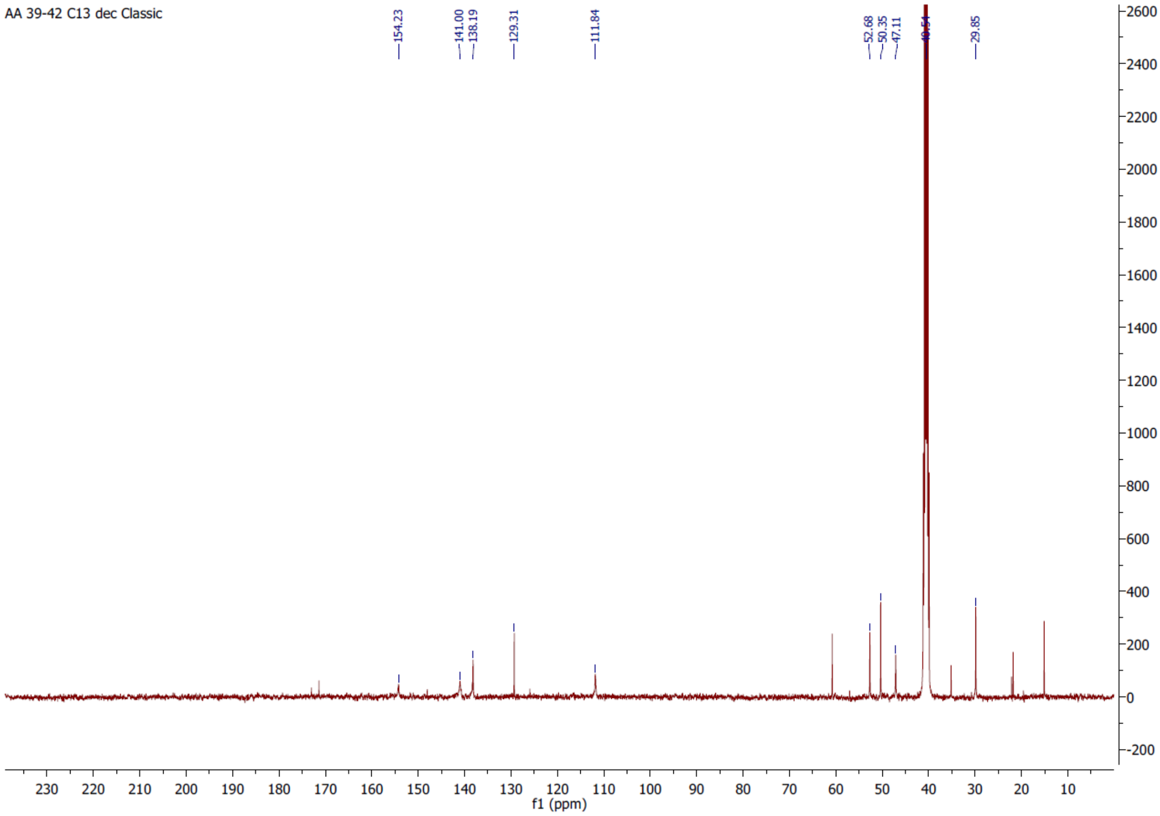

^{13}C NMR spectrum of compound **5b** (100 MHz, DMSO-d_6)

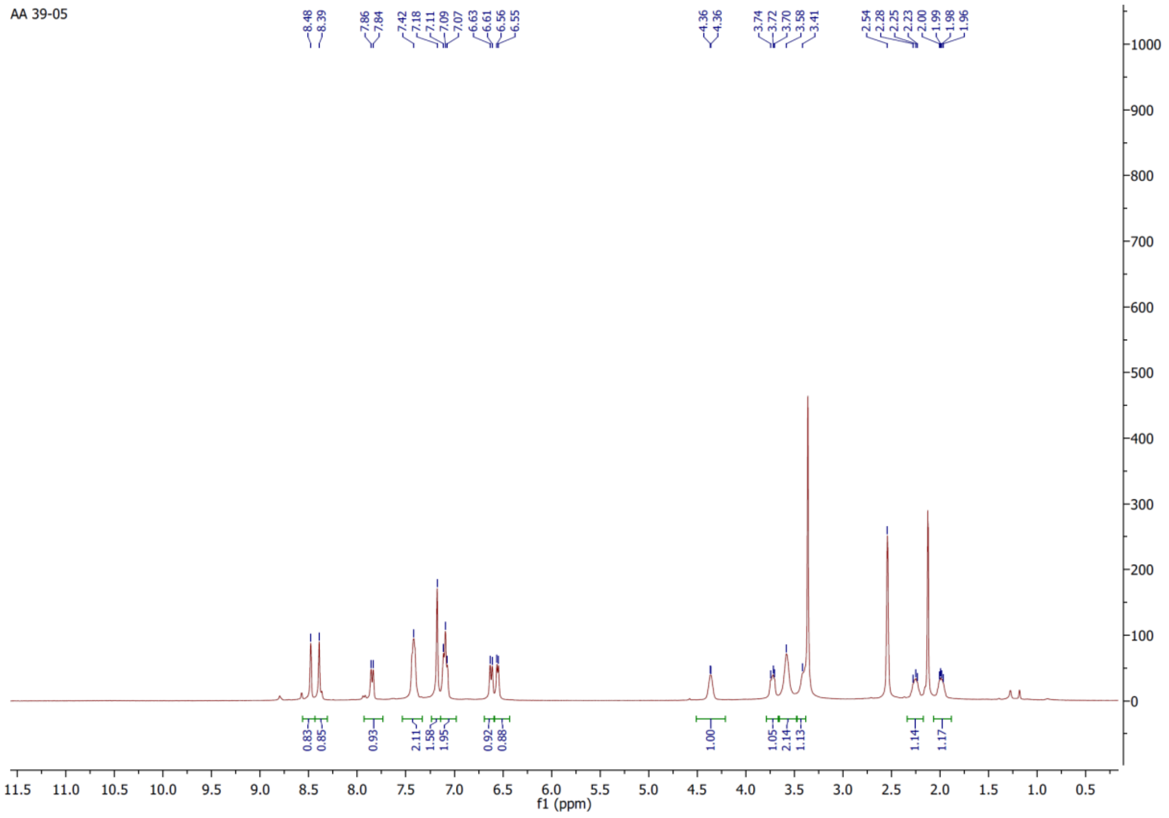

^{1}H NMR spectrum of compound **7a** (400 MHz, DMSO-d_6)

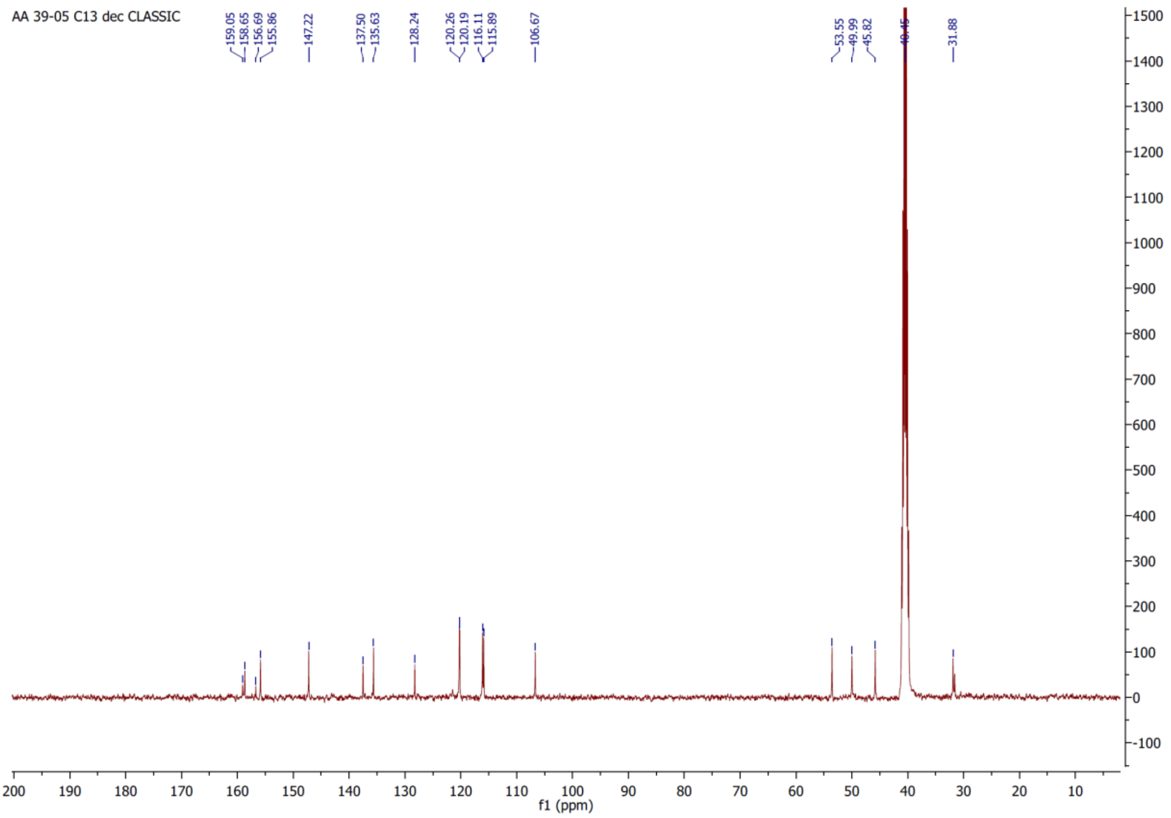

^{13}C NMR spectrum of compound **7a** (100 MHz, DMSO-d_6)

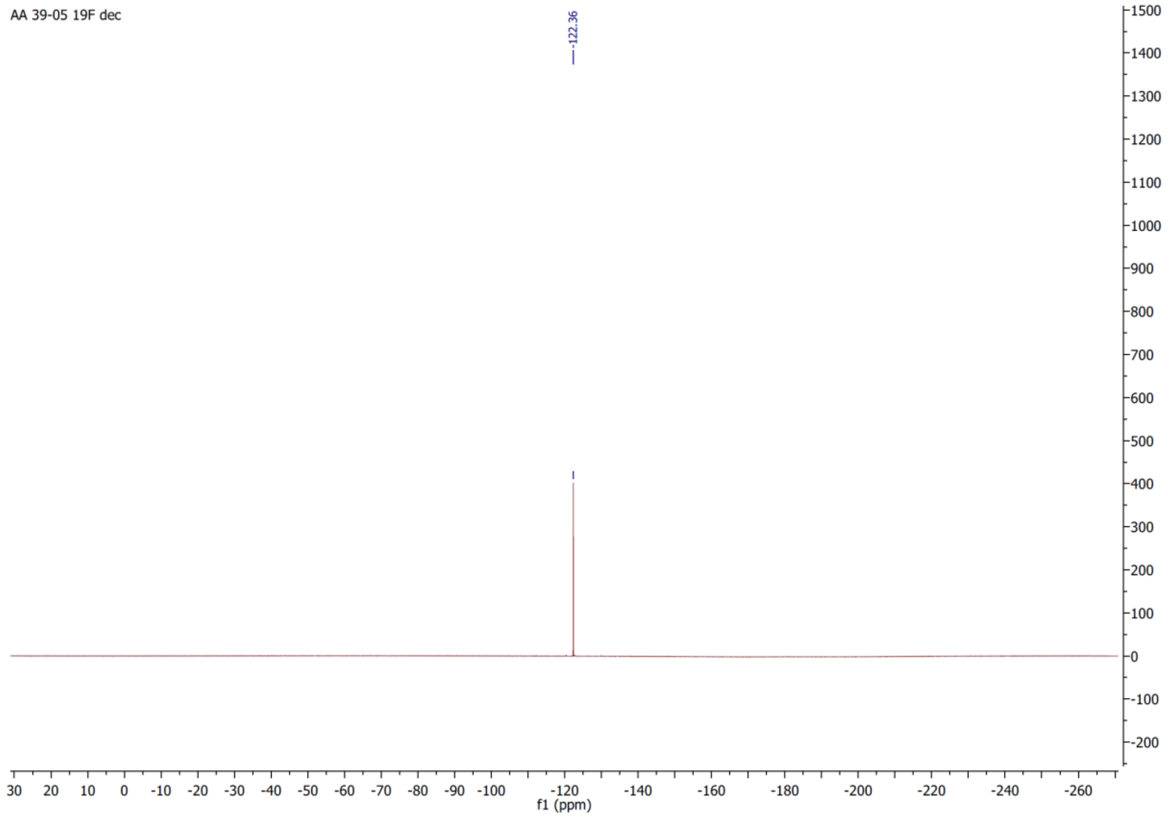

^{19}F NMR spectrum of compound **7a** (376 MHz, DMSO-d_6)

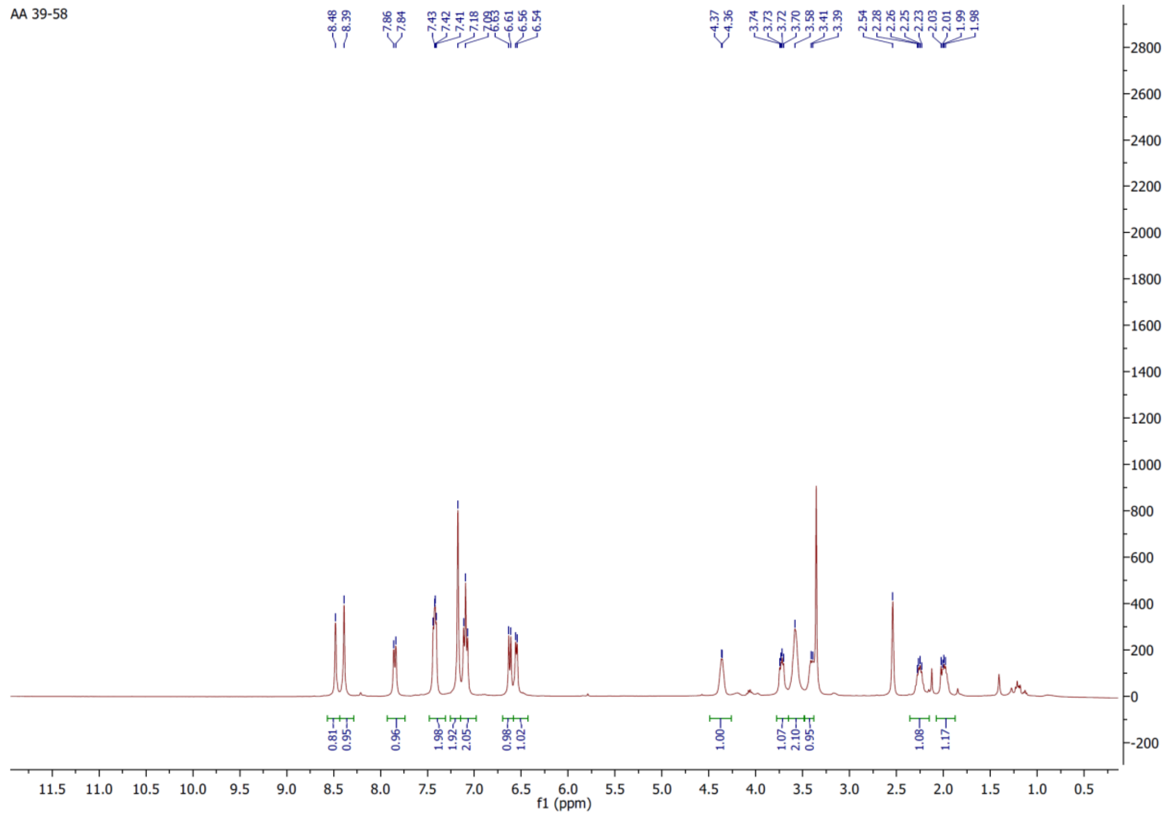

^{1}H NMR spectrum of compound **7b** (400 MHz, DMSO-d_6)

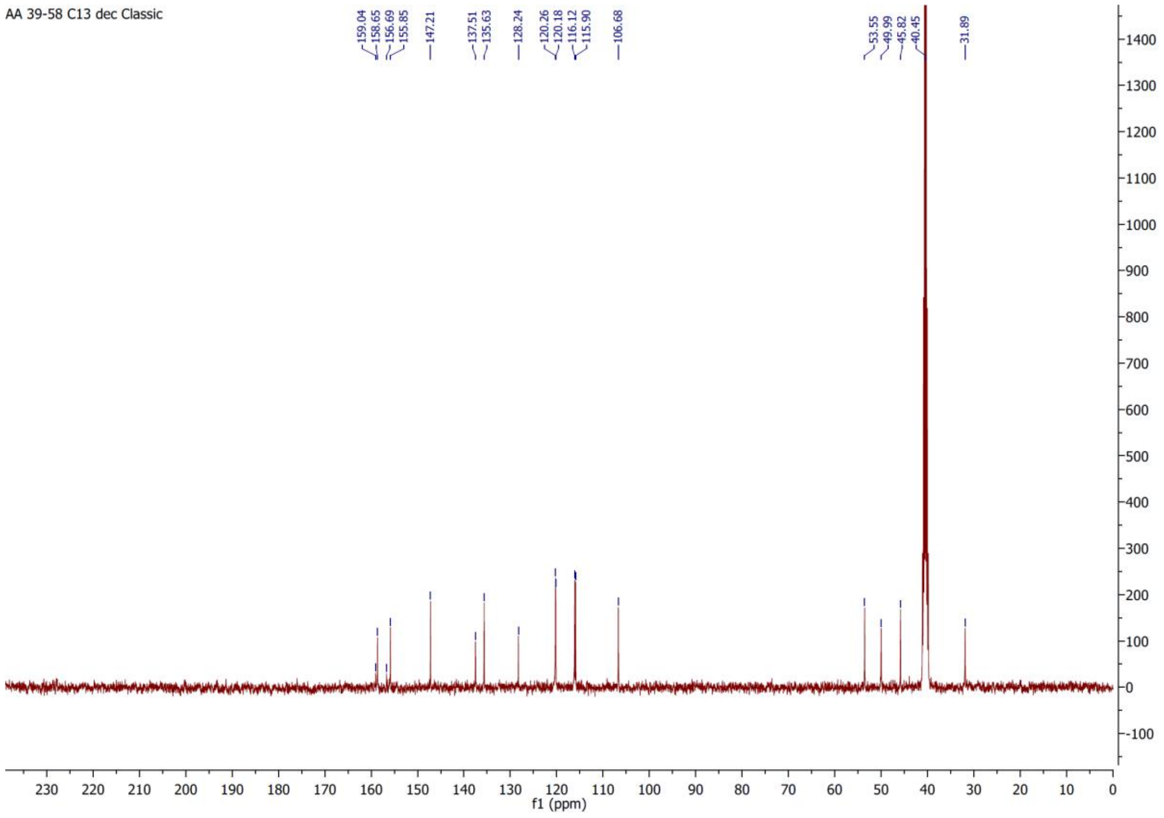

^{13}C NMR spectrum of compound **7b** (100 MHz, DMSO-d_6)

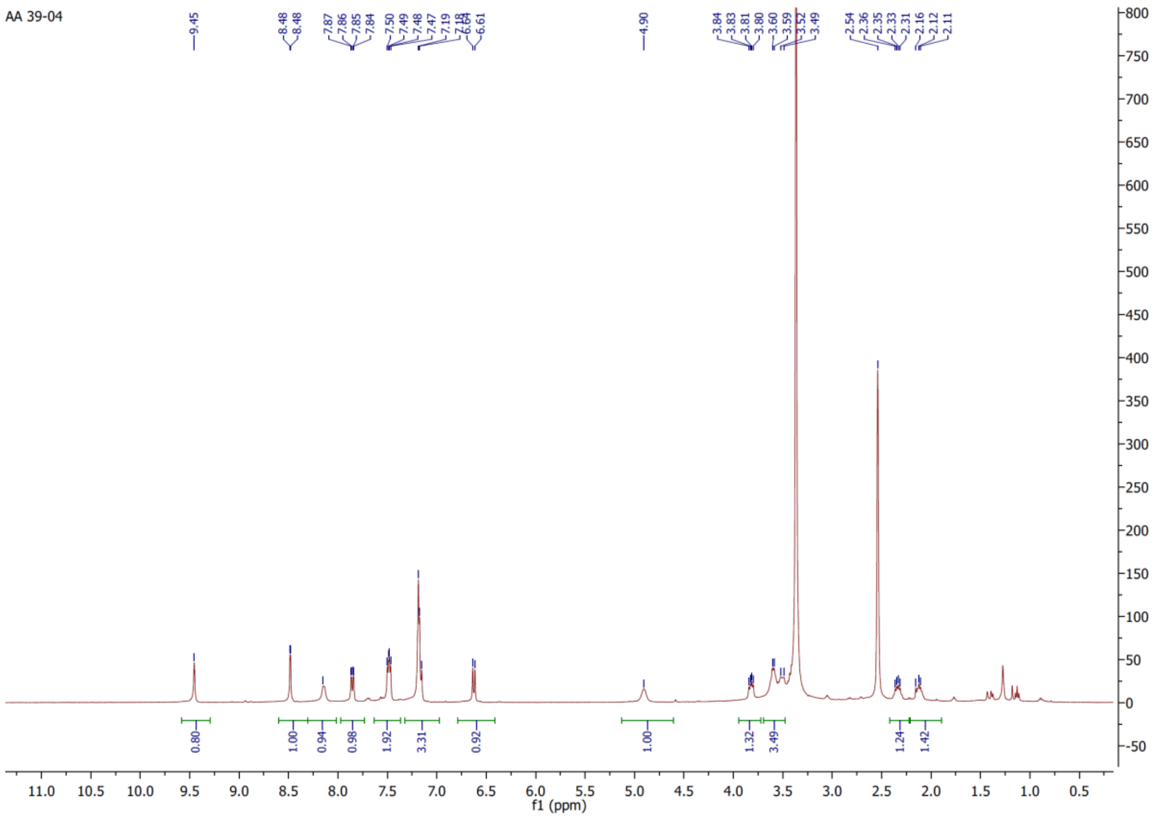

^{1}H NMR spectrum of compound **8a** (400 MHz, DMSO-d_6)

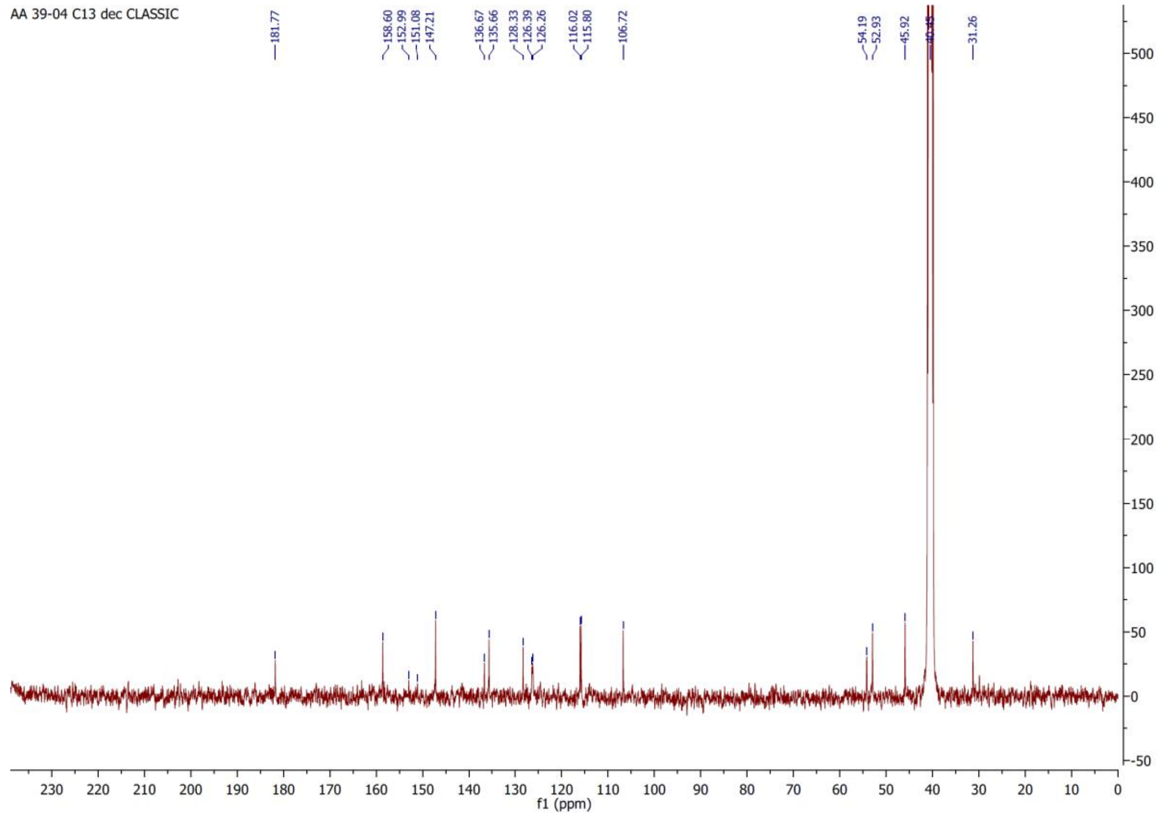

^{13}C NMR spectrum of compound **8a** (100 MHz, DMSO-d_6)

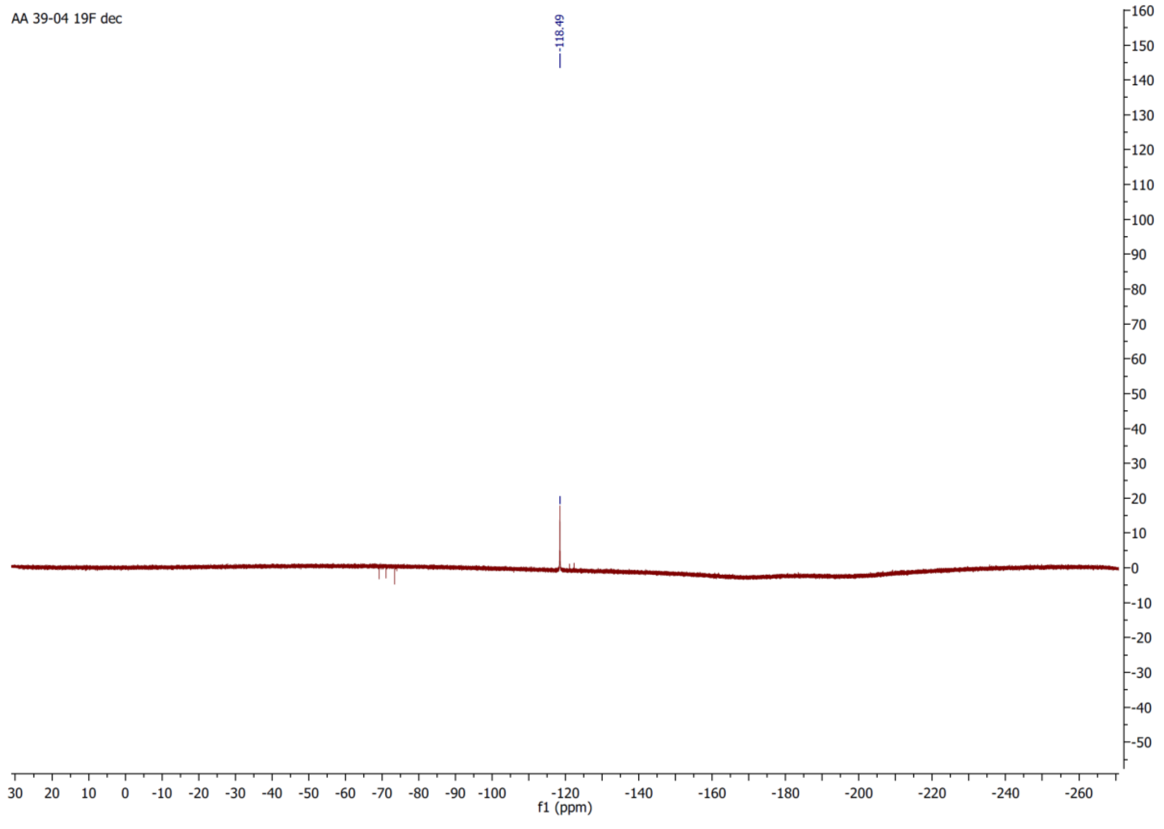

^{19}F NMR spectrum of compound **8a** (376 MHz, DMSO-d_6)

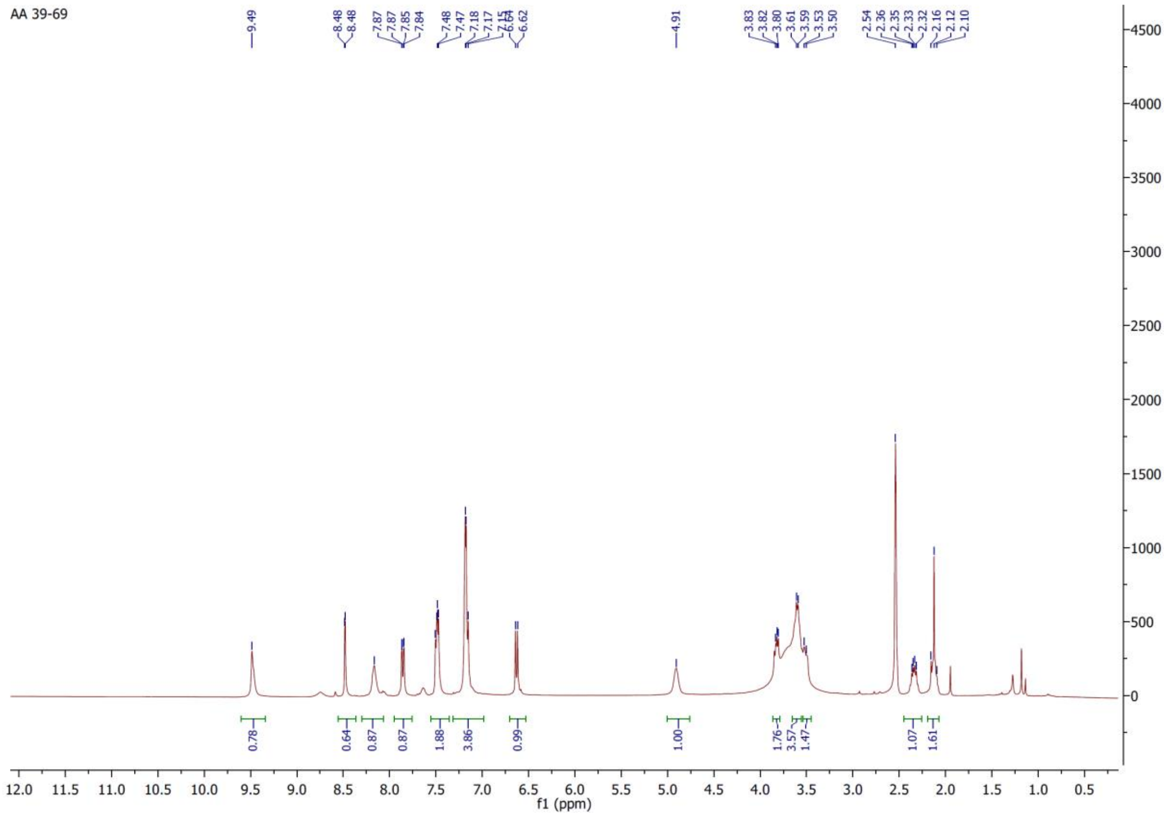

^{1}H NMR spectrum of compound **8b** (400 MHz, DMSO-d_6)

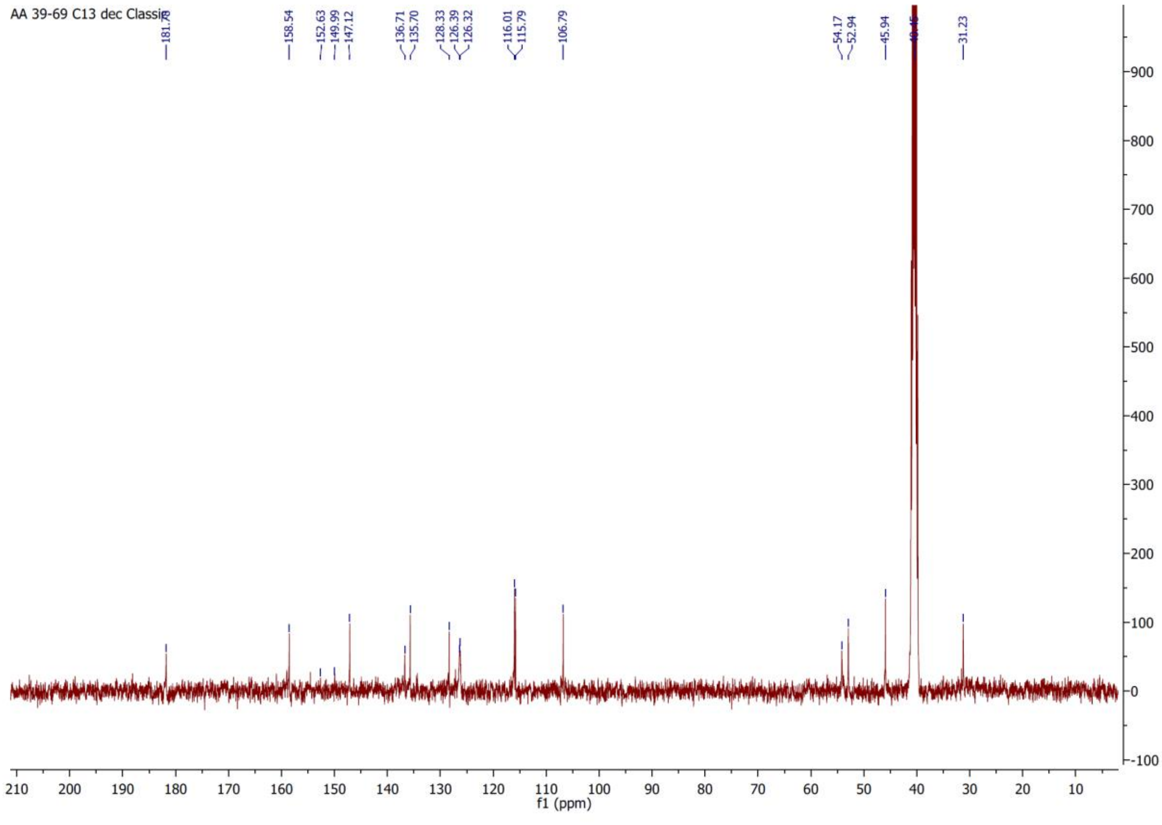

^{13}C NMR spectrum of compound **8b** (100 MHz, DMSO-d_6)

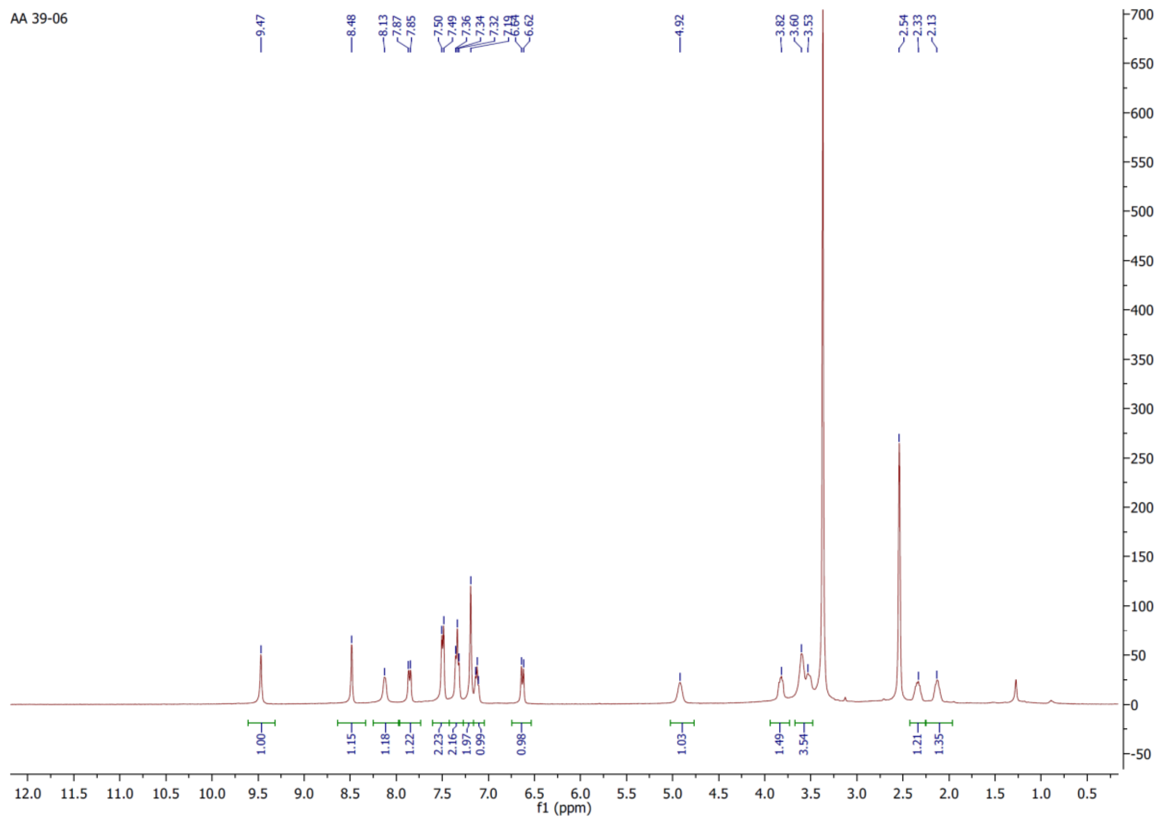

^{1}H NMR spectrum of compound **9a** (400 MHz, DMSO-d_6)

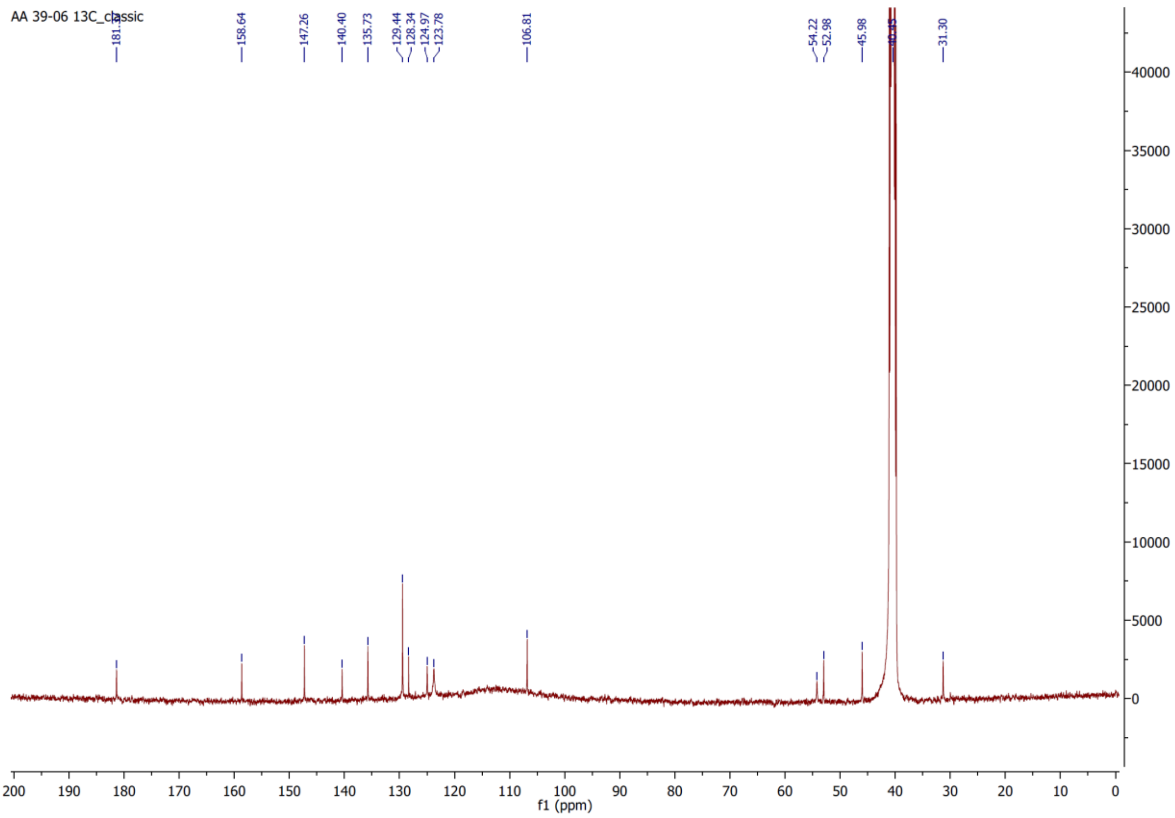

^{13}C NMR spectrum of compound **9a** (100 MHz, DMSO-d_6)

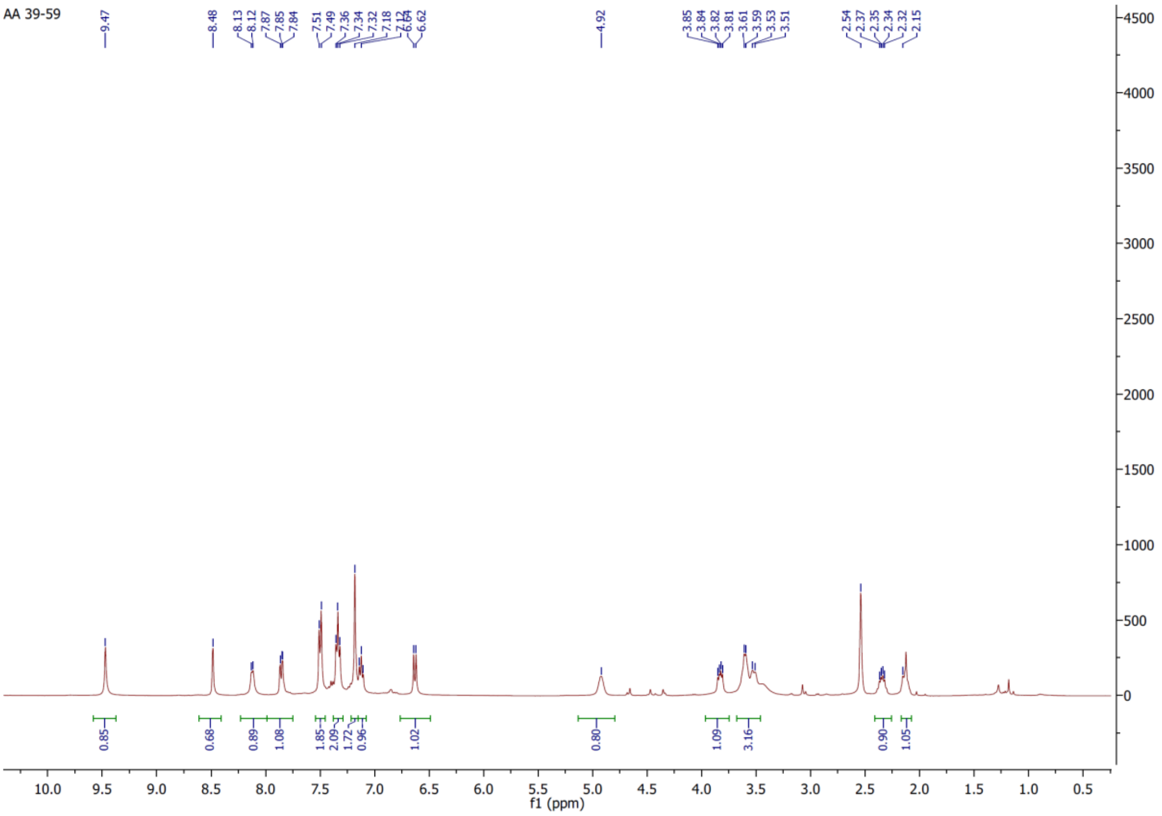

^{1}H NMR spectrum of compound **9b** (400 MHz, DMSO-d_6)

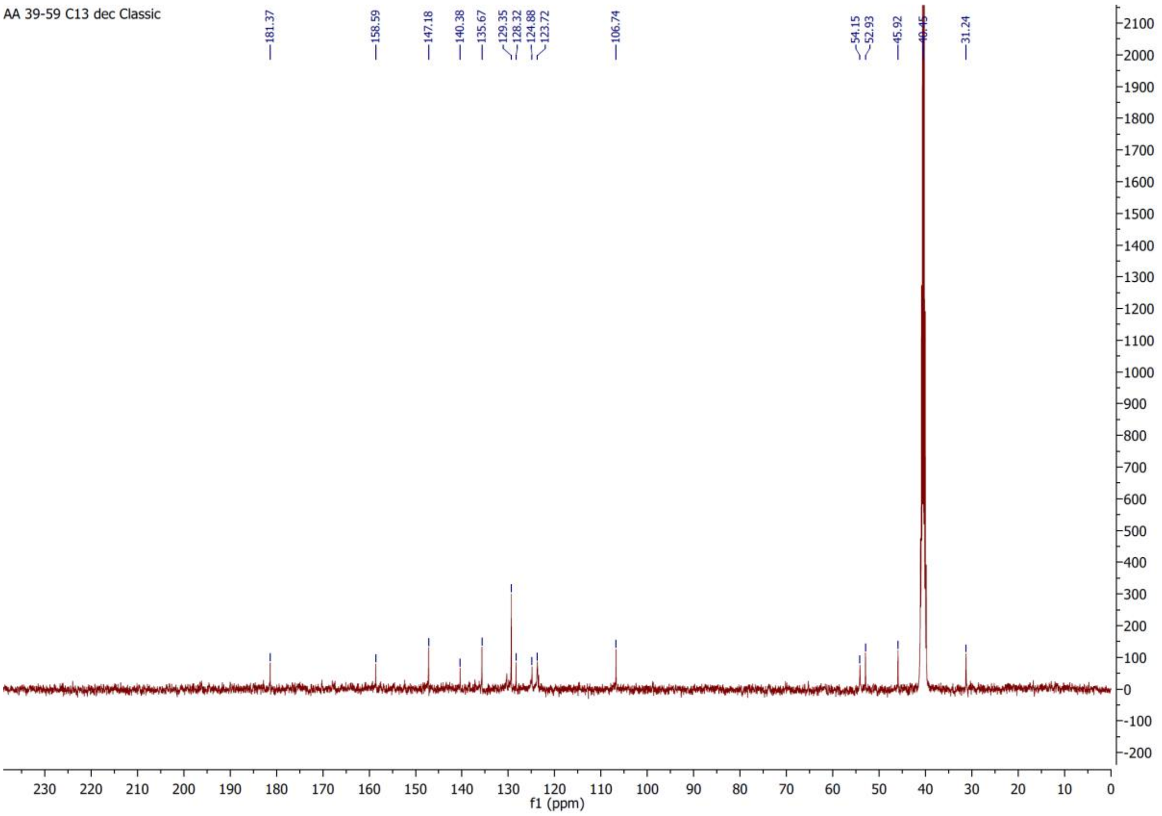

^{13}C NMR spectrum of compound **9b** (100 MHz, DMSO-d_6)

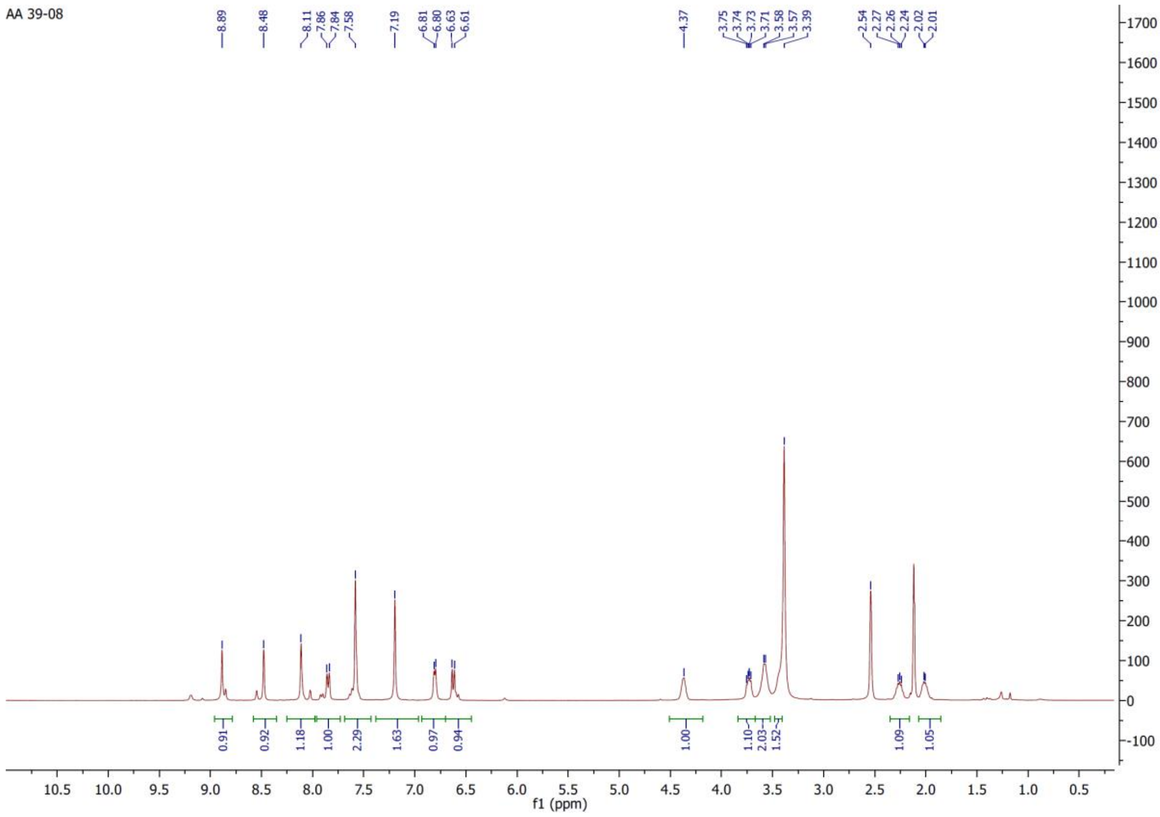

^{1}H NMR spectrum of compound **10a** (400 MHz, DMSO-d_6)

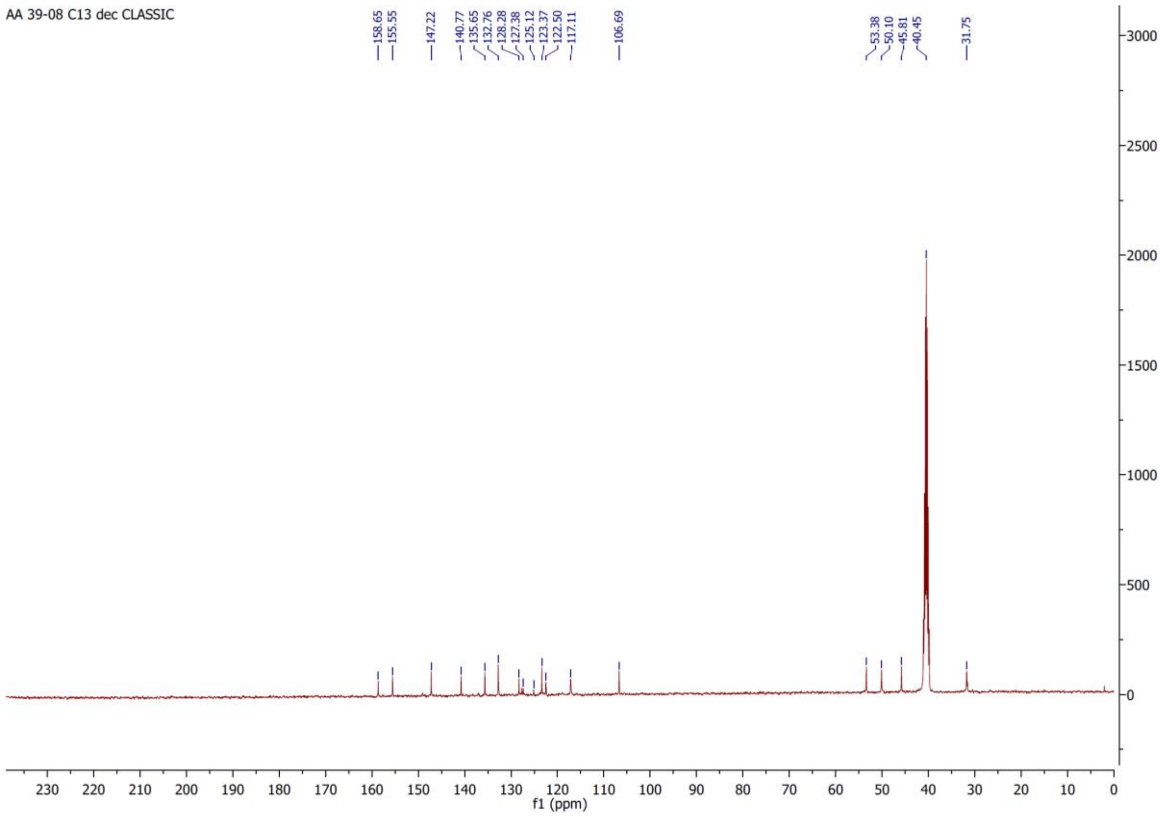

^{13}C NMR spectrum of compound **10a** (100 MHz, DMSO-d_6)

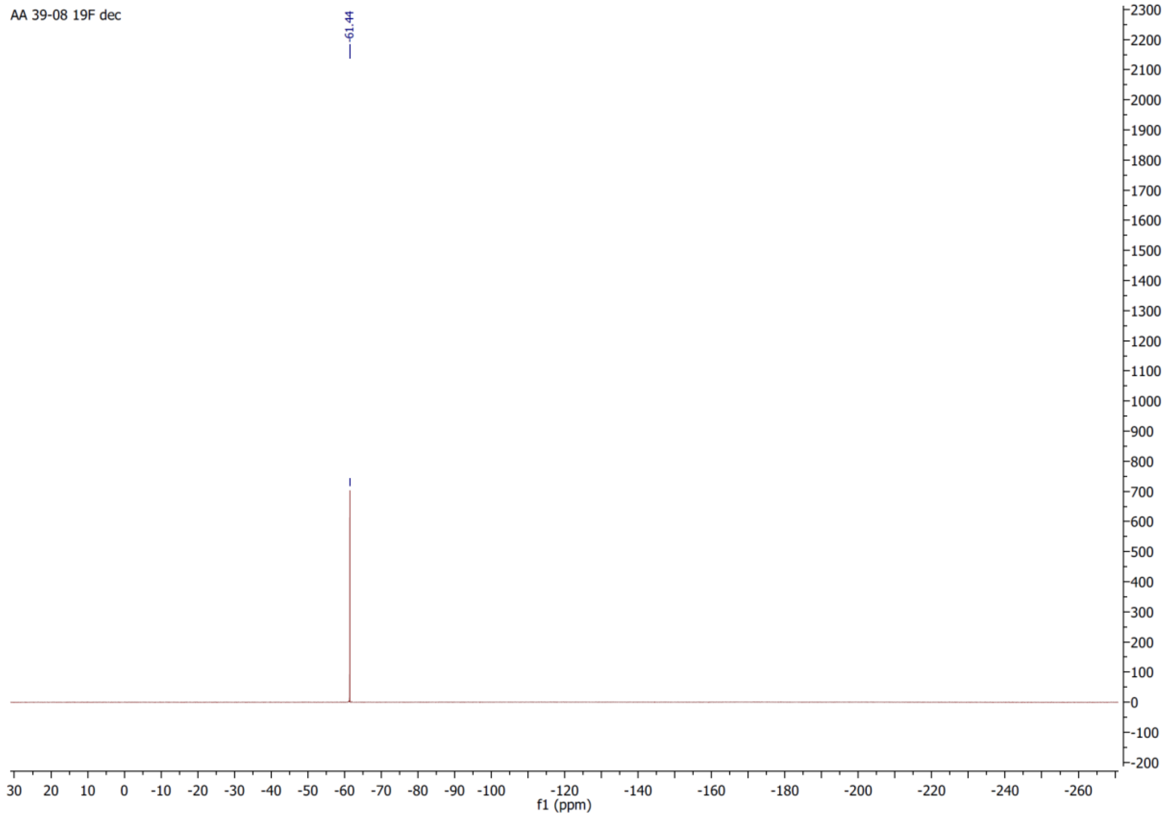

^{19}F NMR spectrum of compound **10a** (376 MHz, DMSO-d_6)

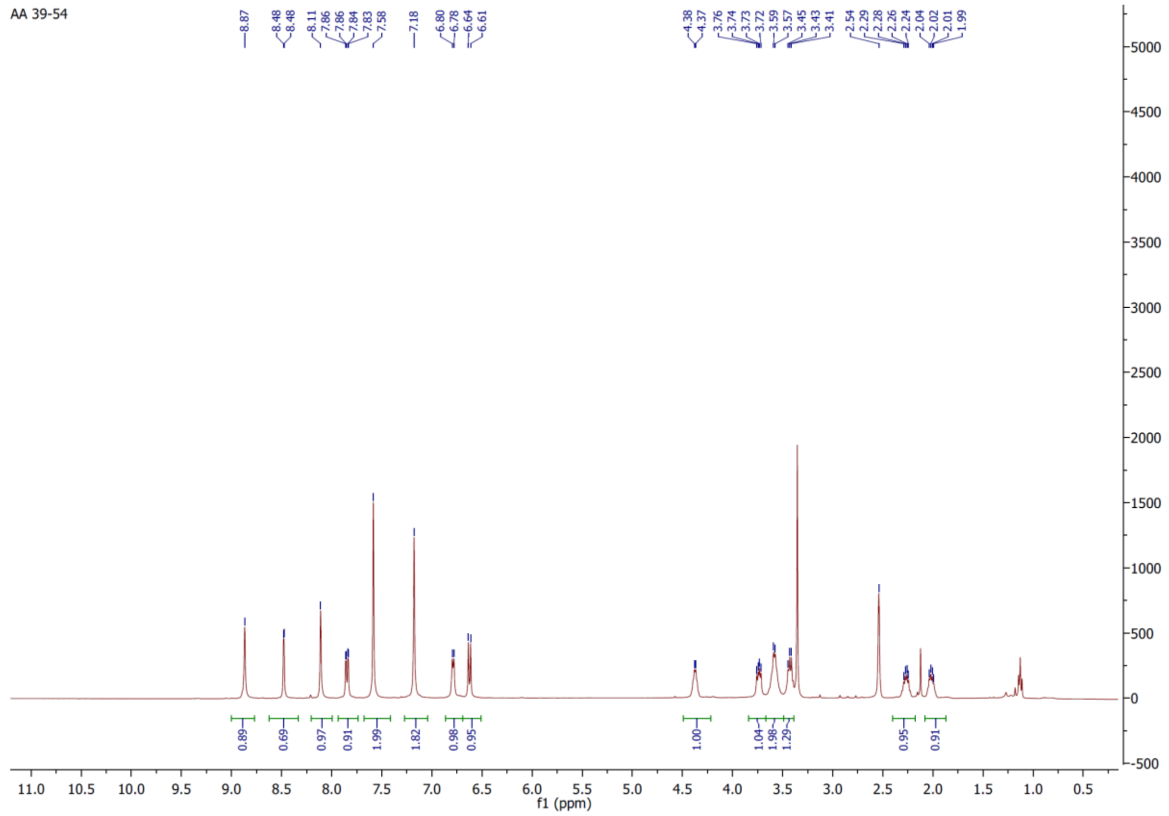

^{1}H NMR spectrum of compound **10b** (400 MHz, DMSO-d_6)

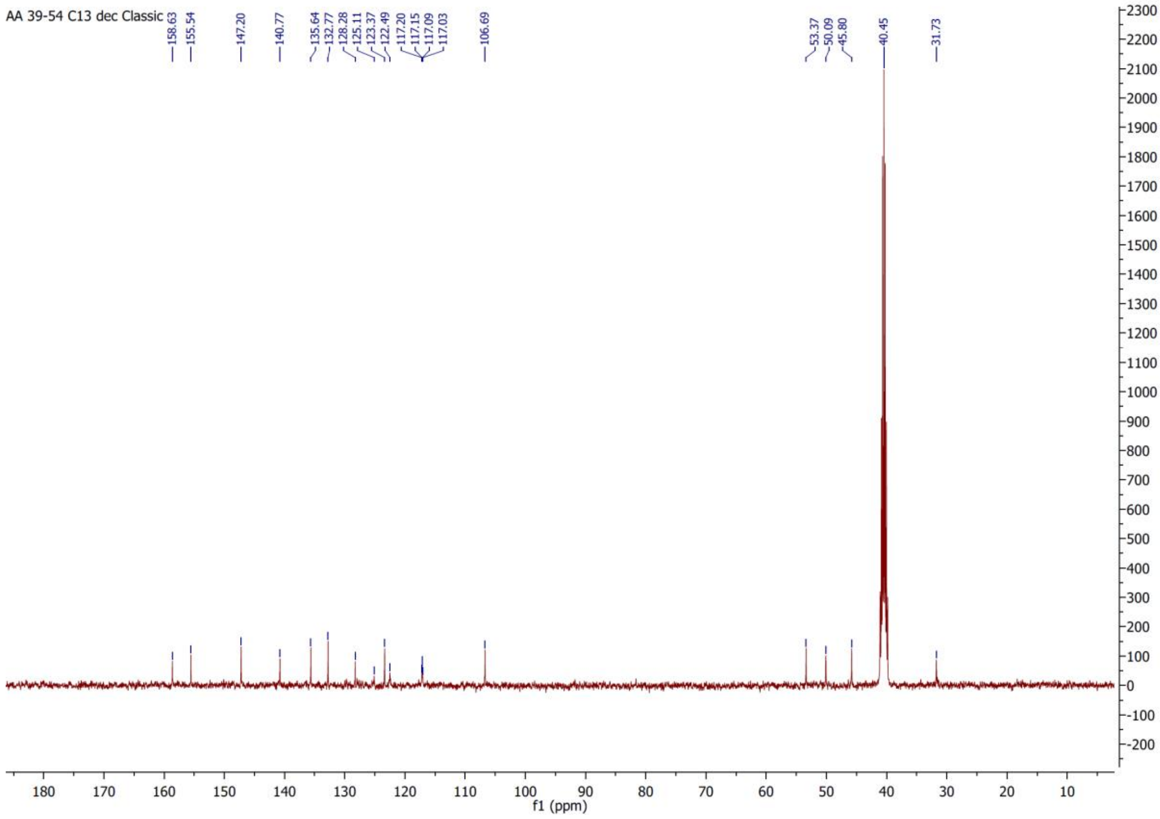

^{13}C NMR spectrum of compound **10b** (100 MHz, DMSO-d_6)

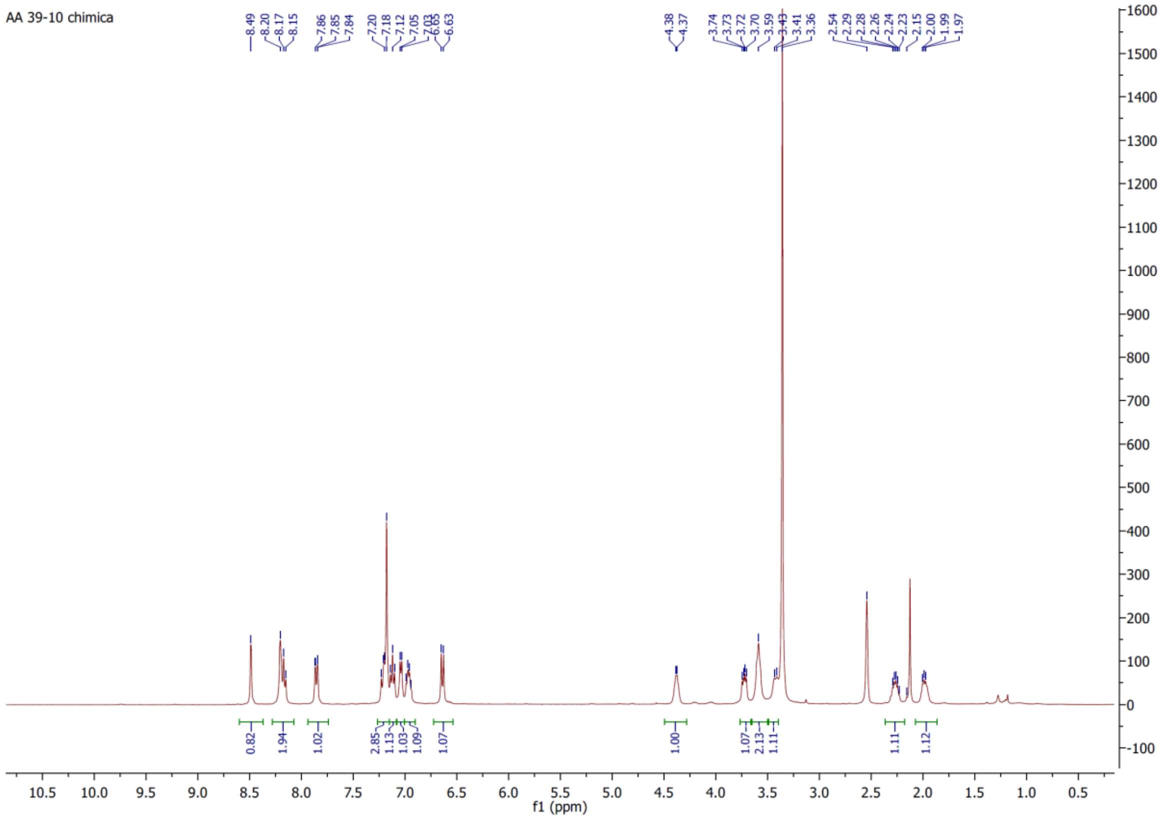

^{1}H NMR spectrum of compound **11a** (400 MHz, DMSO-d_6)

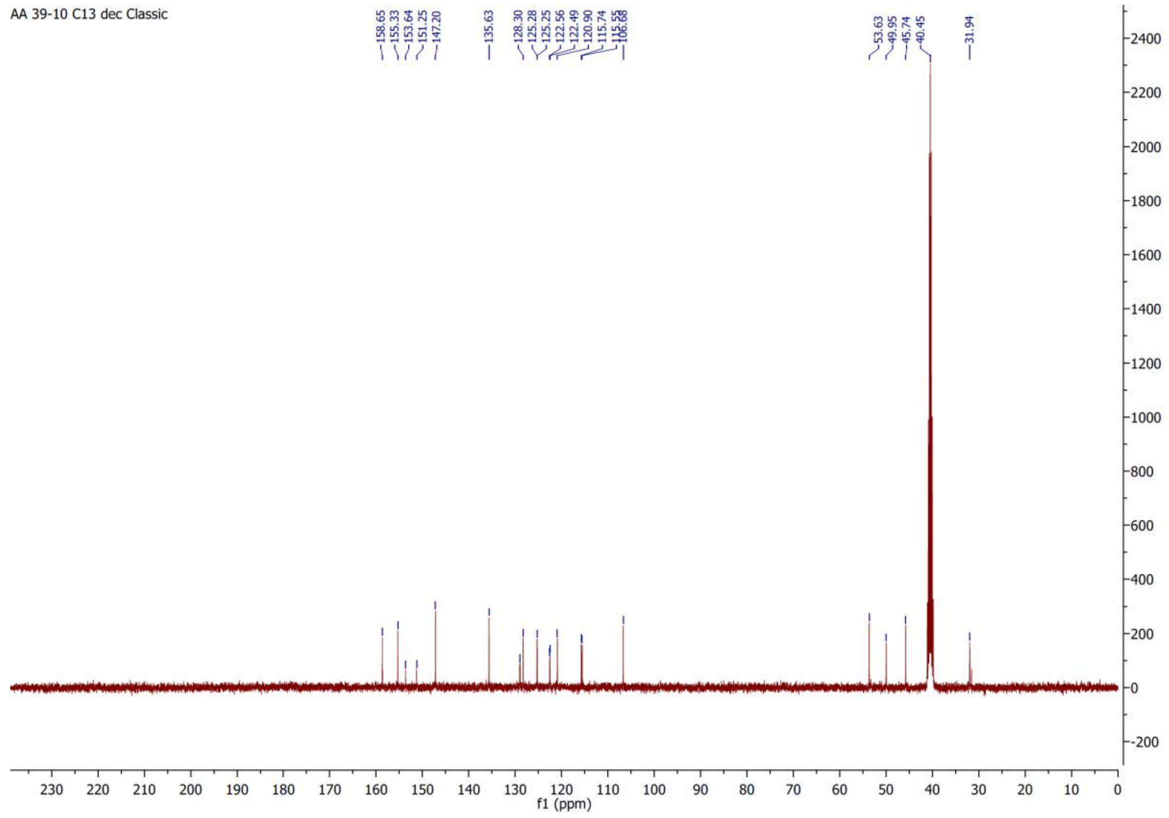

^{13}C NMR spectrum of compound **11a** (100 MHz, DMSO-d_6)

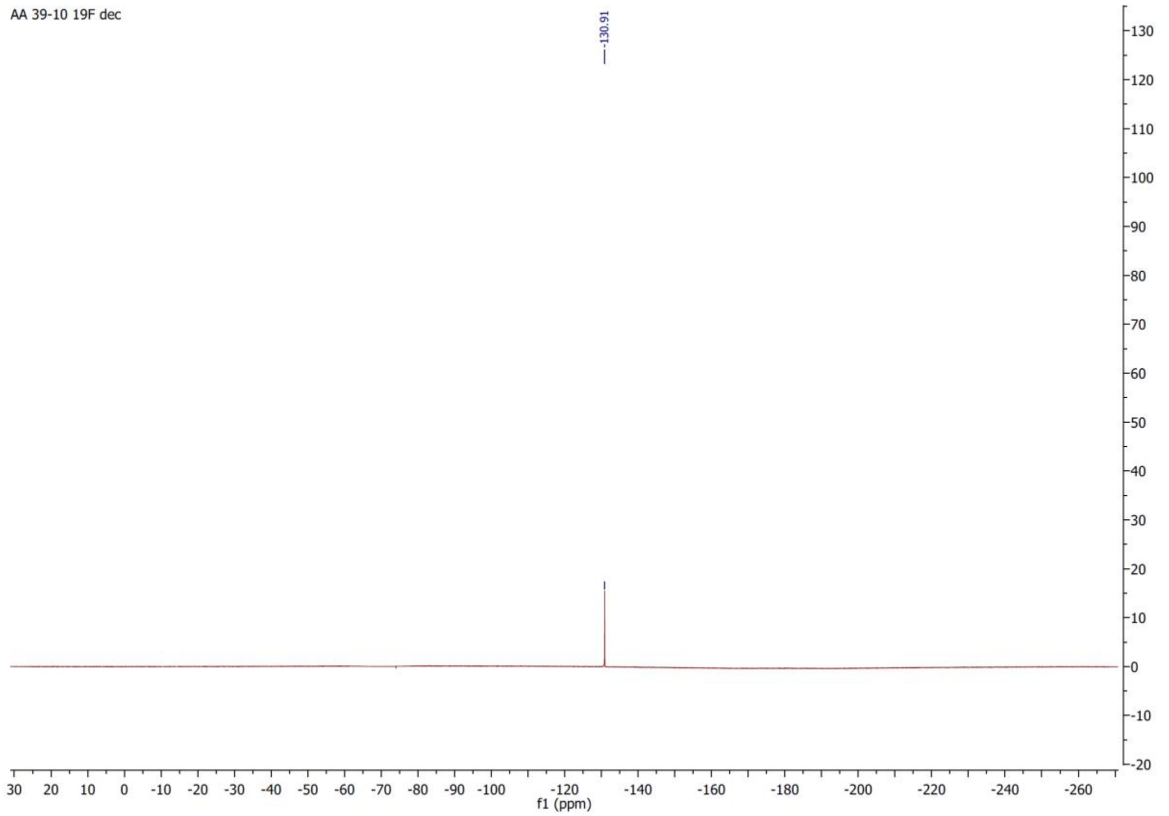

^{19}F NMR spectrum of compound **11a** (376 MHz, DMSO-d_6)

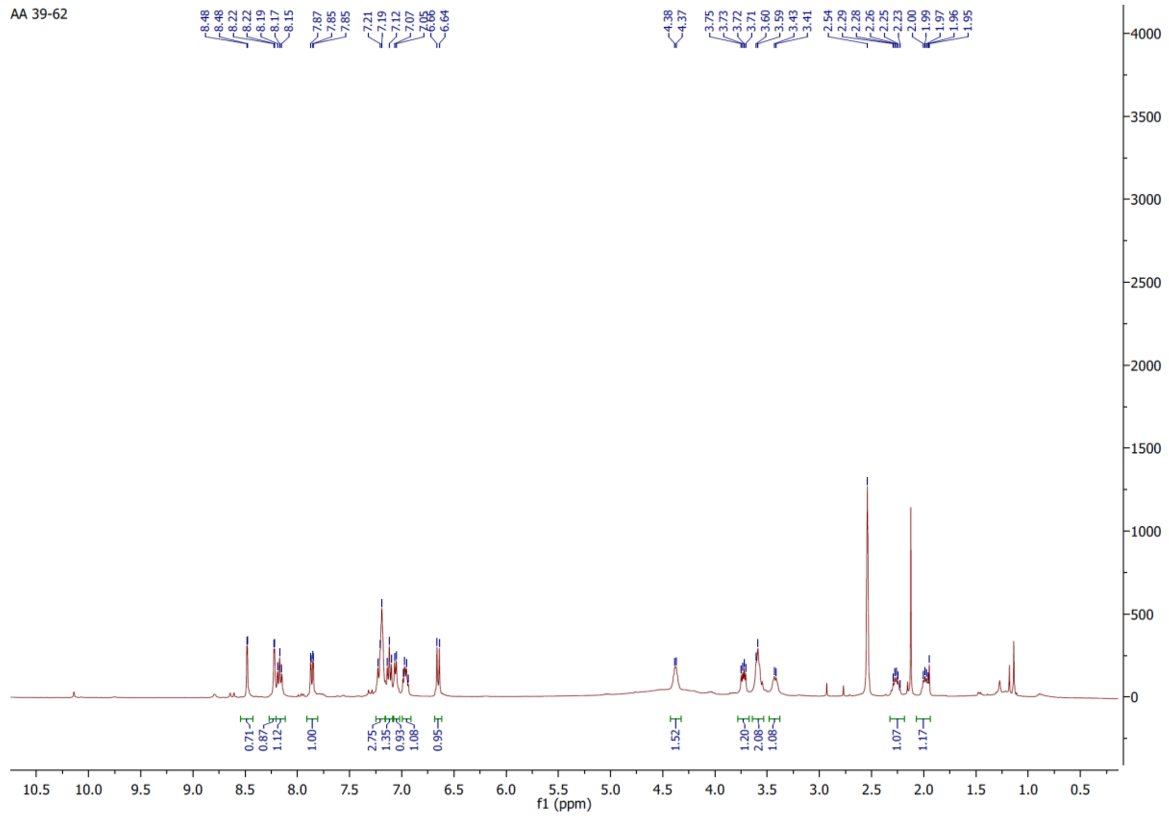

^{1}H NMR spectrum of compound **11b** (400 MHz, DMSO-d_6)

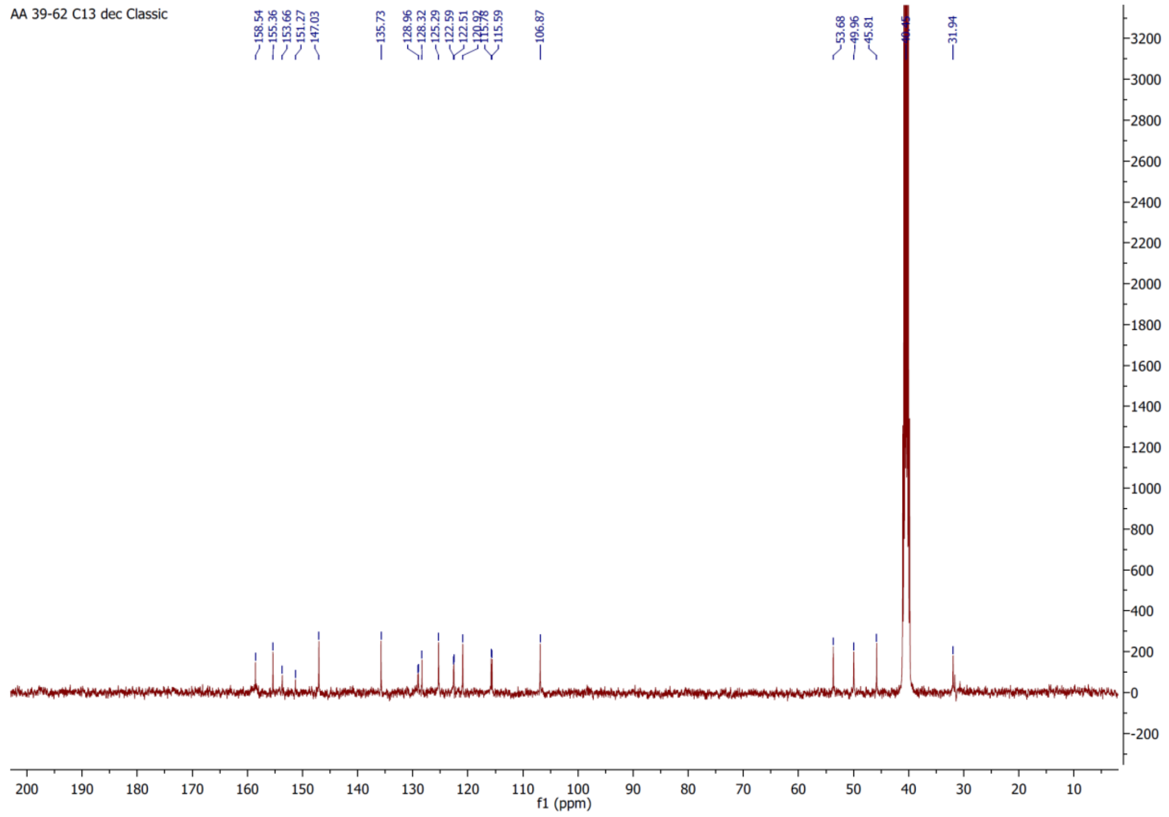

^{13}C NMR spectrum of compound **11b** (100 MHz, DMSO-d_6)

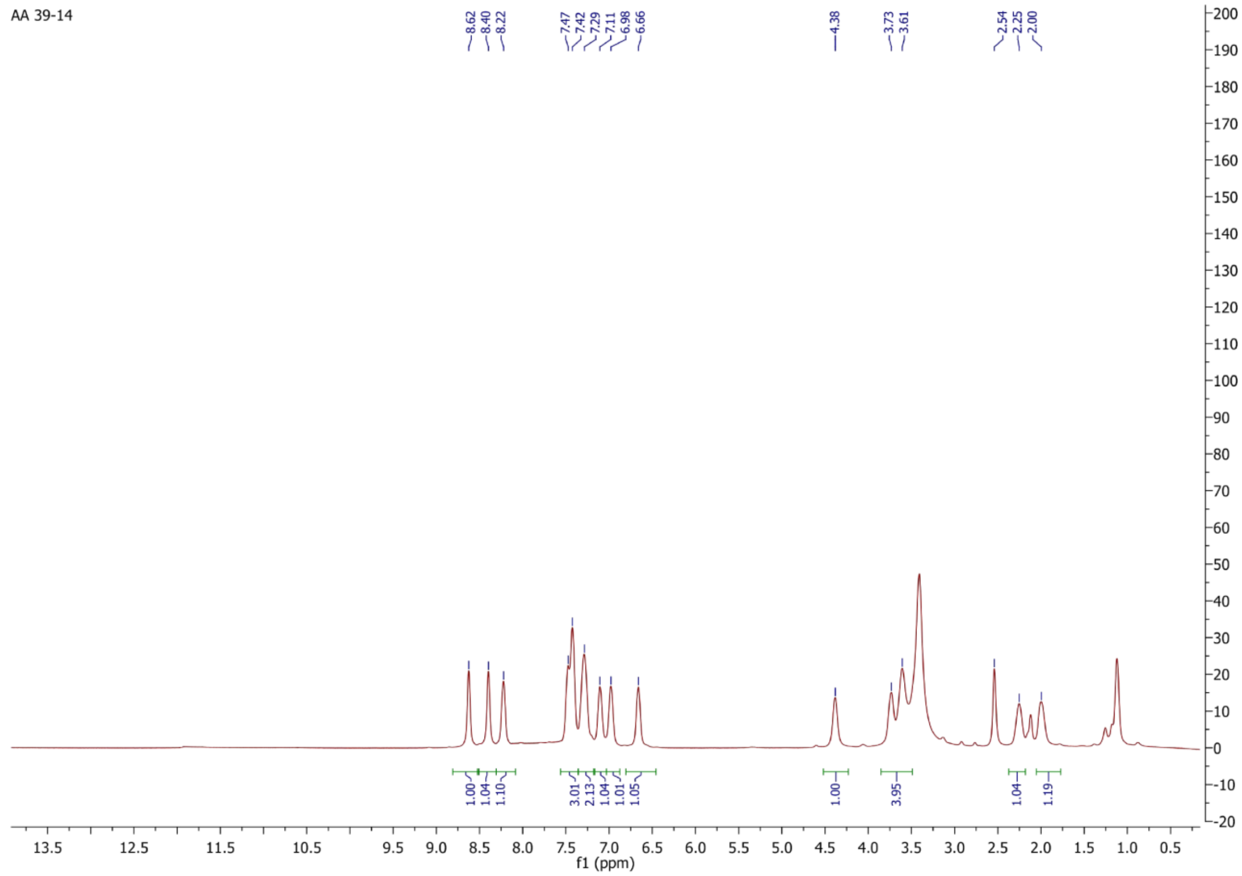

^{1}H NMR spectrum of compound **12a** (400 MHz, DMSO-d_6)

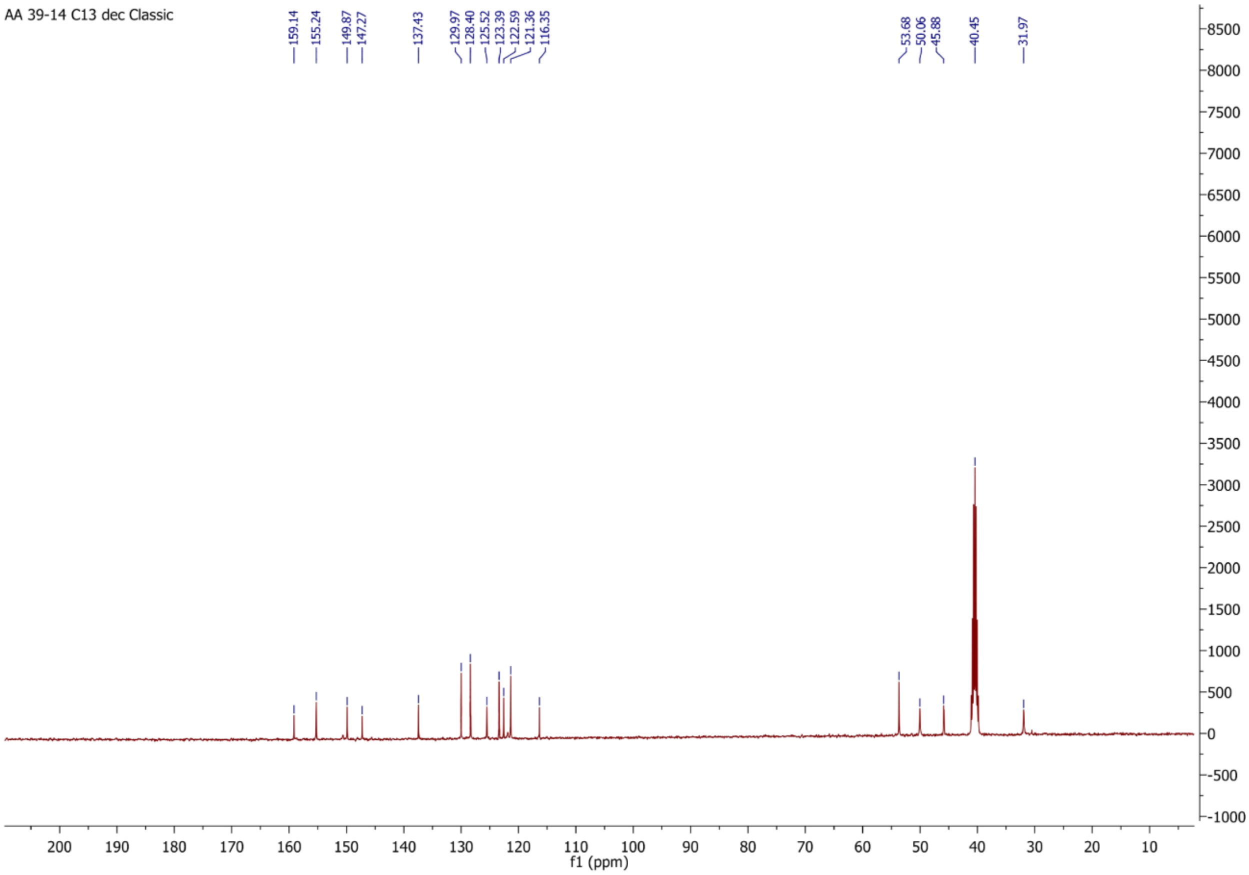

^{13}C NMR spectrum of compound **12a** (100 MHz, DMSO-d_6)

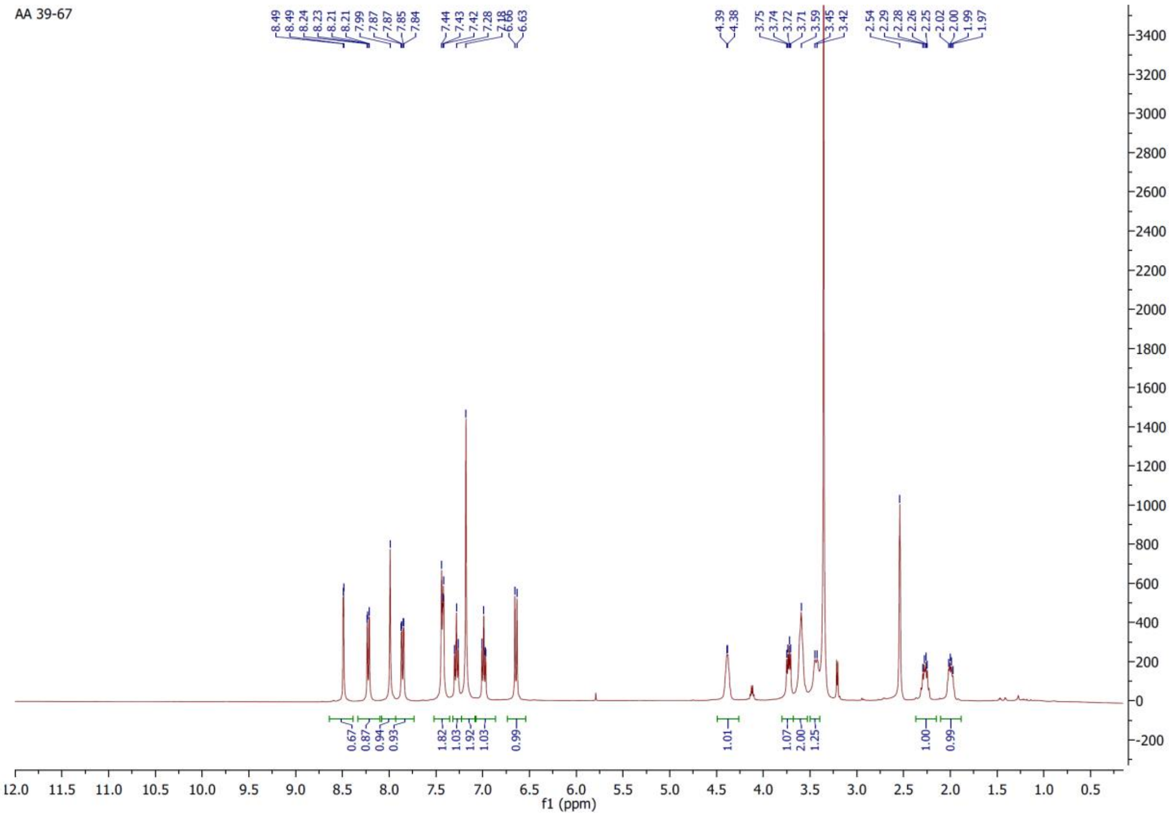

^{1}H NMR spectrum of compound **12b** (400 MHz, DMSO-d_6)

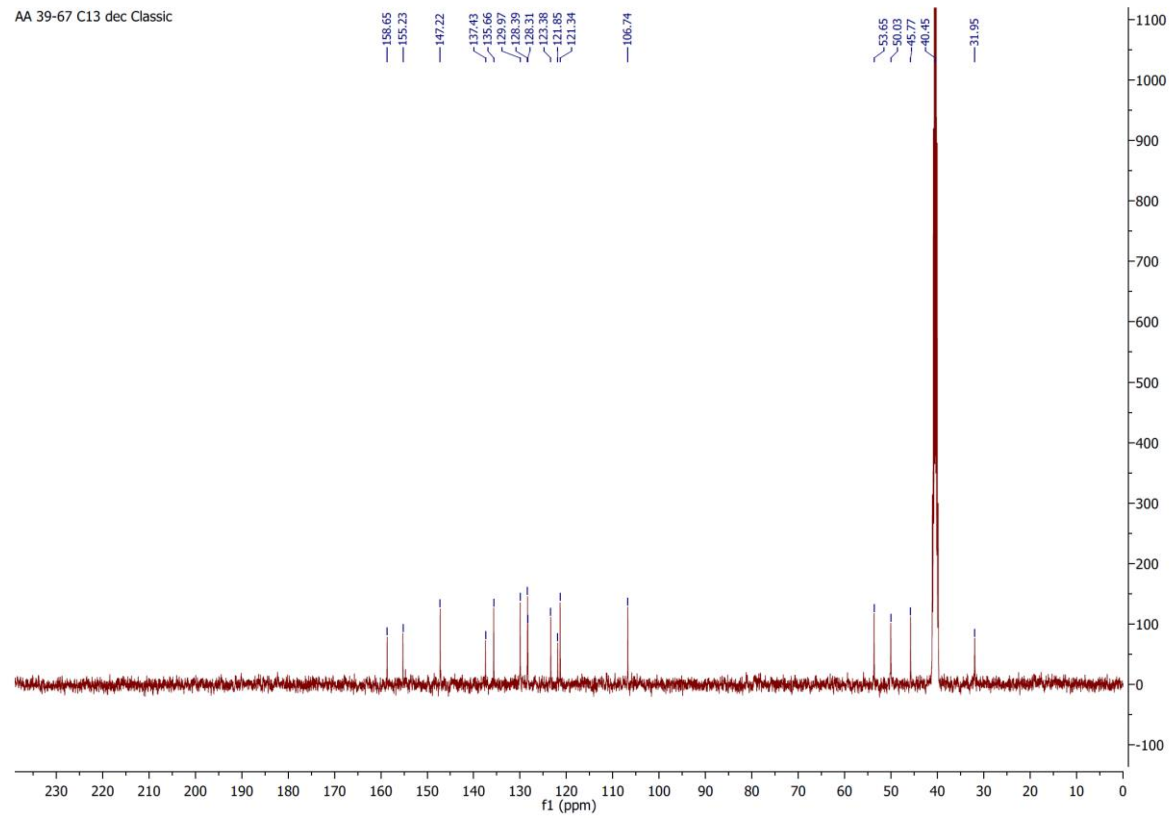

^{13}C NMR spectrum of compound **12b** (100 MHz, DMSO-d_6)

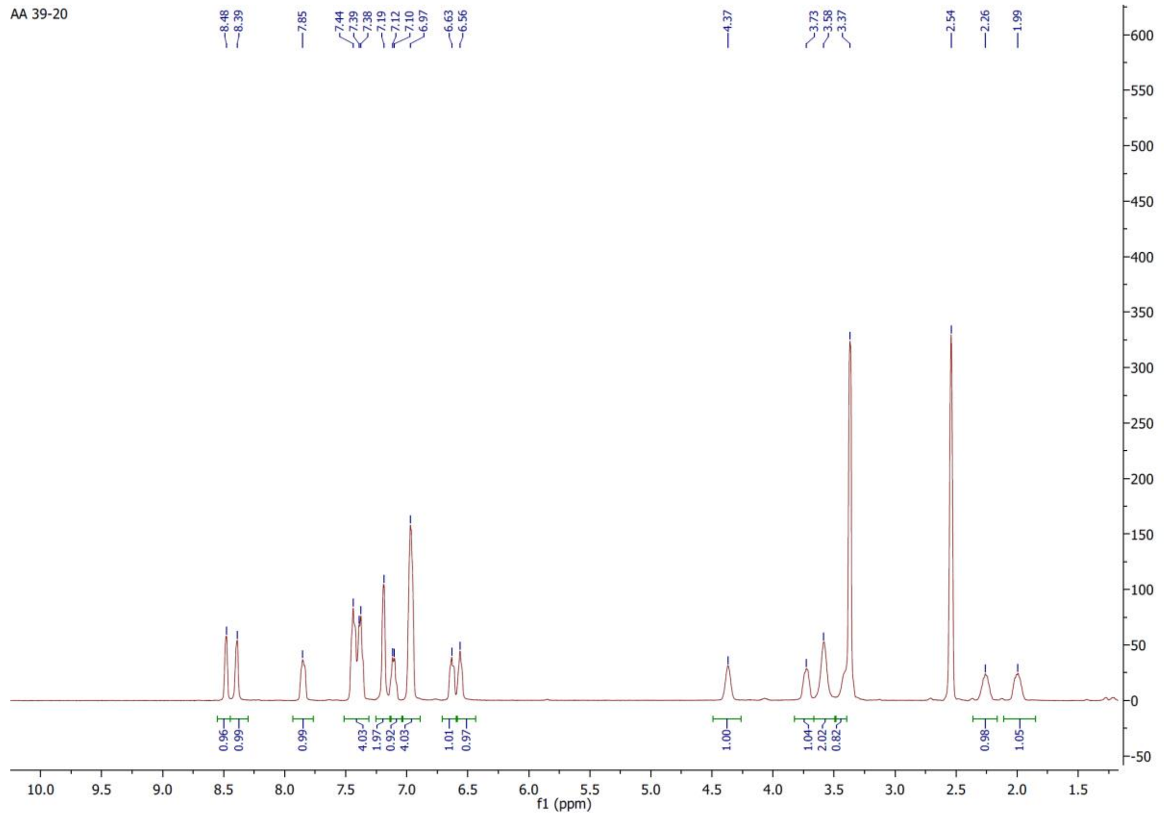

^{1}H NMR spectrum of compound **13a** (400 MHz, DMSO-d_6)

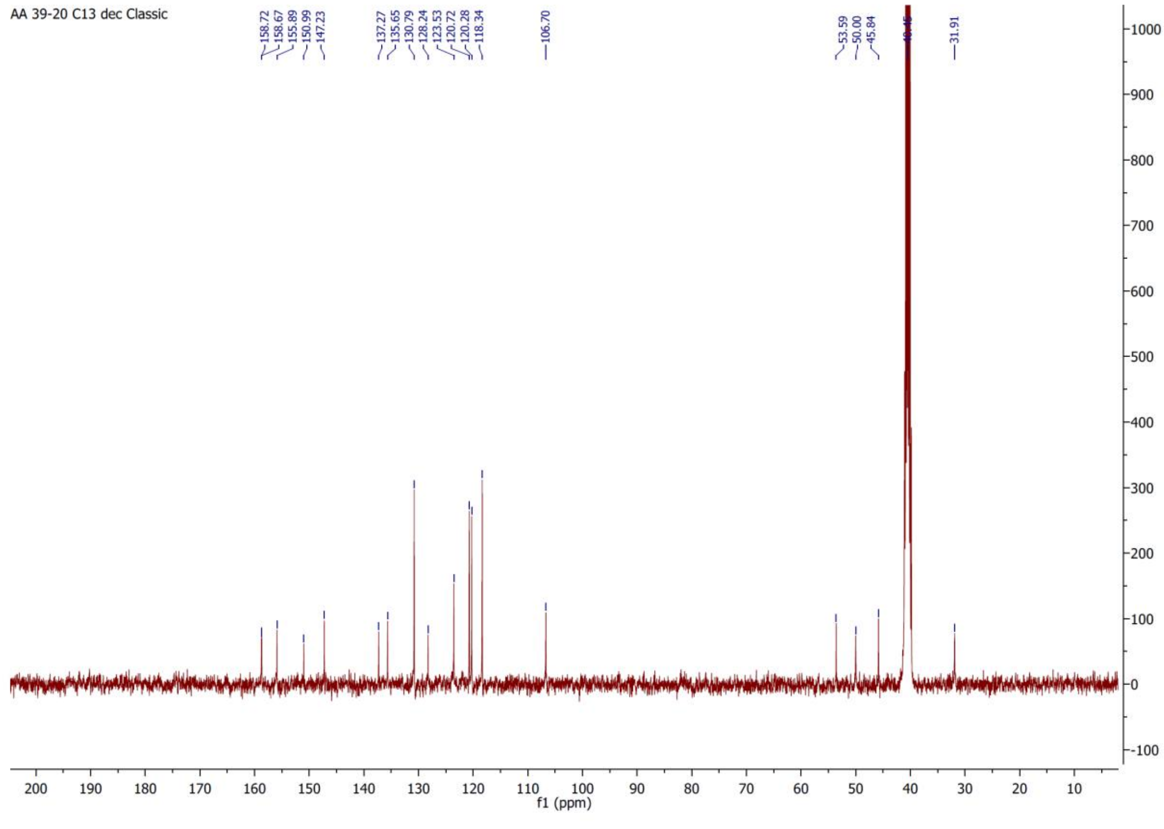

^{13}C NMR spectrum of compound **13a** (100 MHz, DMSO-d_6)

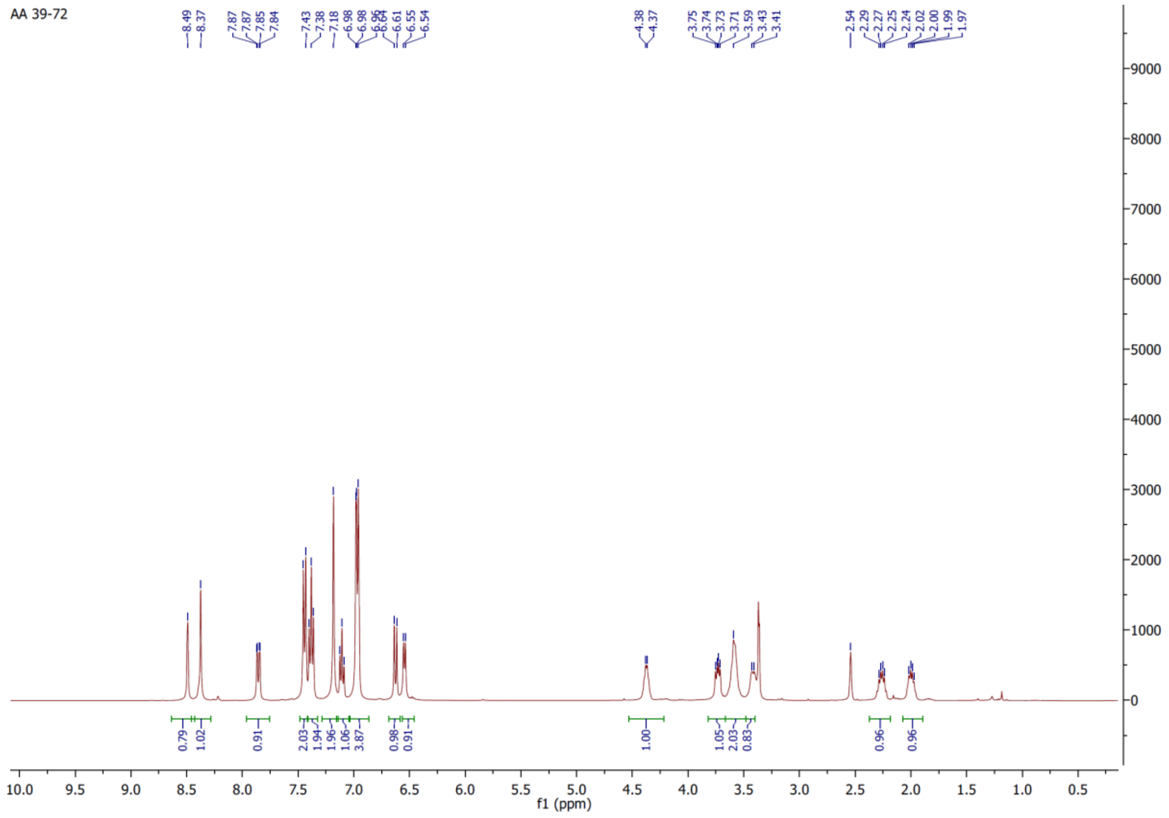

^{1}H NMR spectrum of compound **13b** (400 MHz, DMSO-d_6)

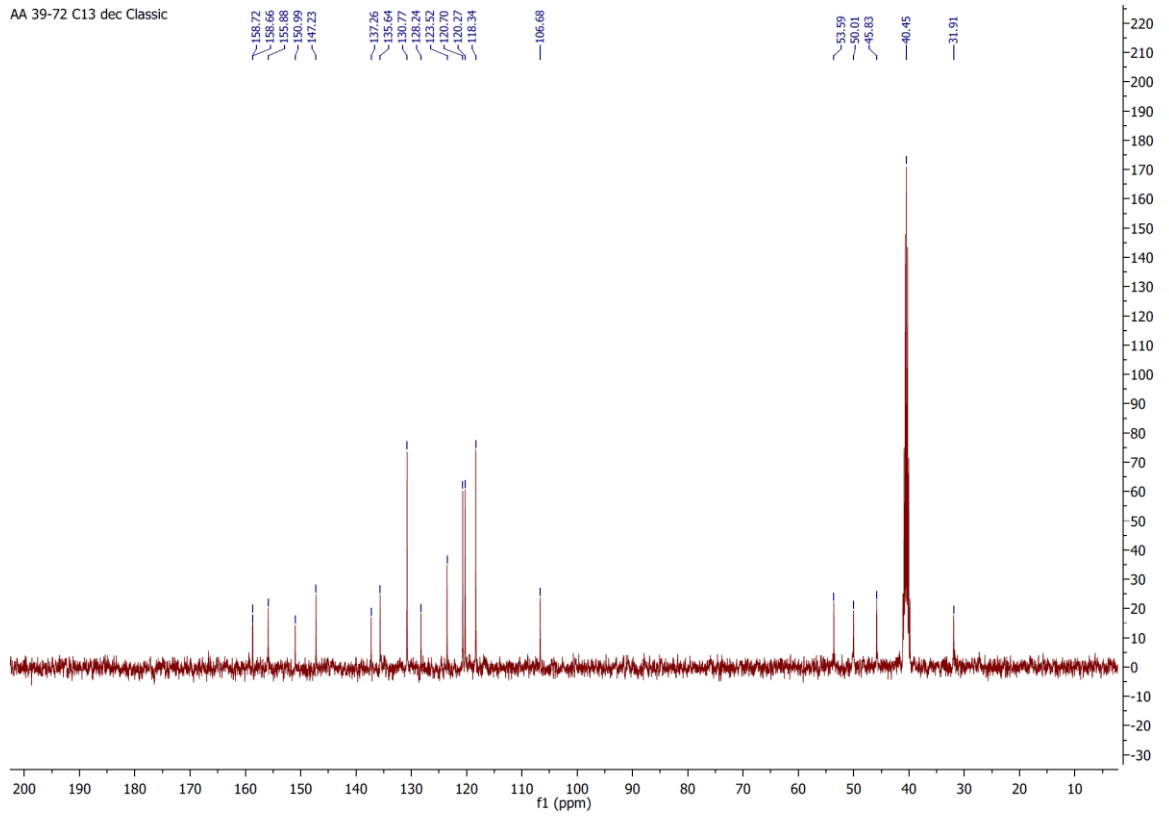

^{13}C NMR spectrum of compound **13b** (100 MHz, DMSO-d_6)

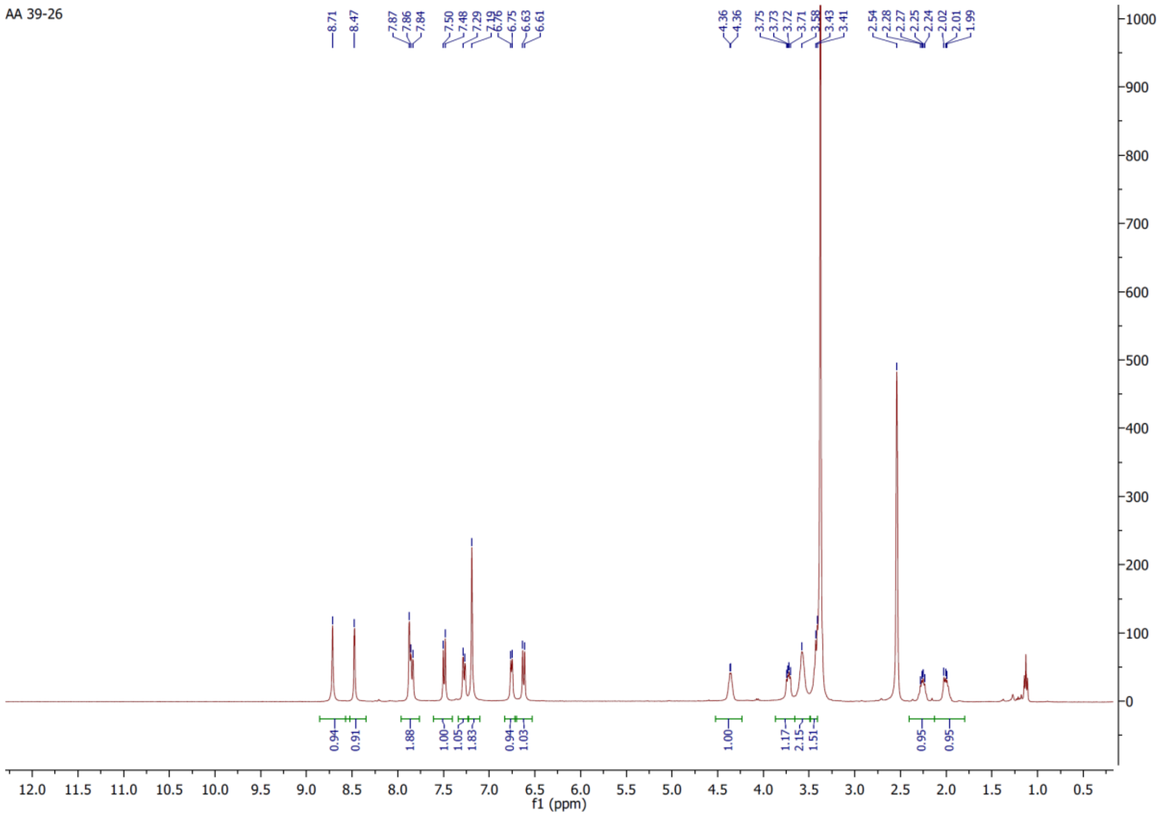

^{1}H NMR spectrum of compound **14a** (400 MHz, DMSO-d_6)

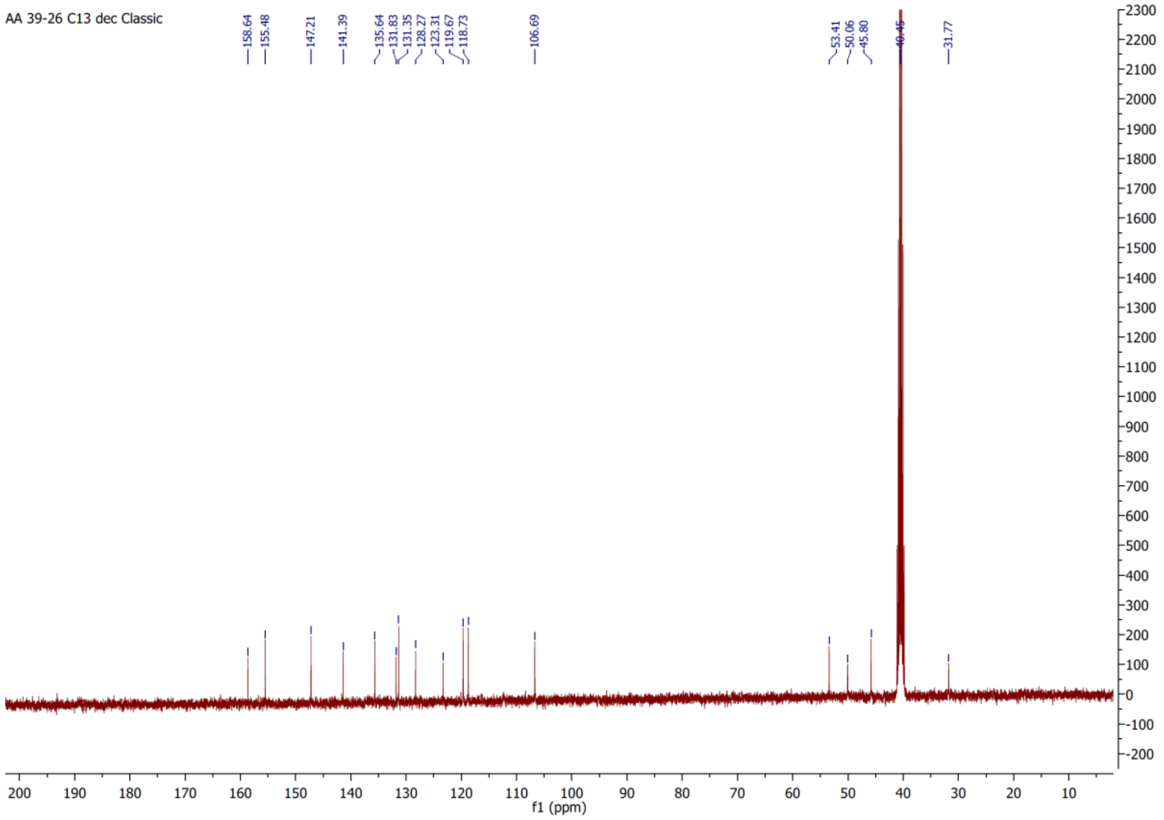

^{13}C NMR spectrum of compound **14a** (100 MHz, DMSO-d_6)

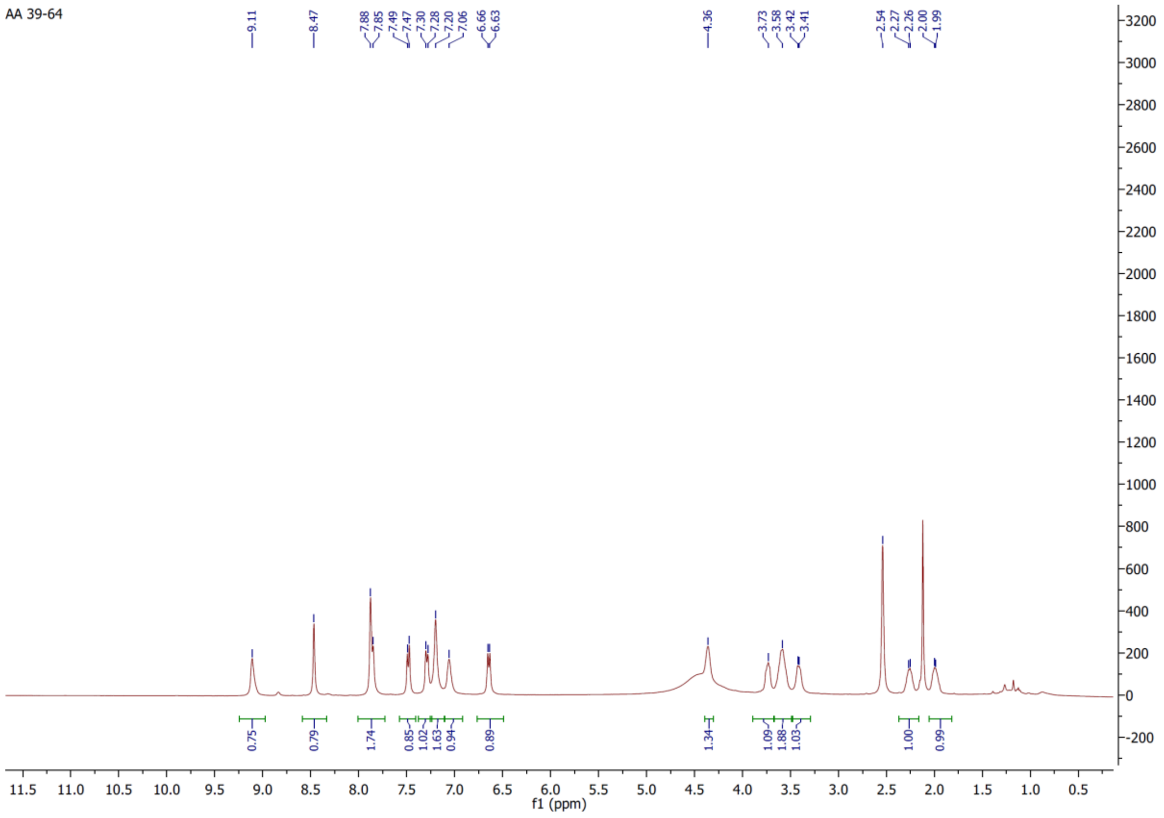

^{1}H NMR spectrum of compound **14b** (400 MHz, DMSO-d_6)

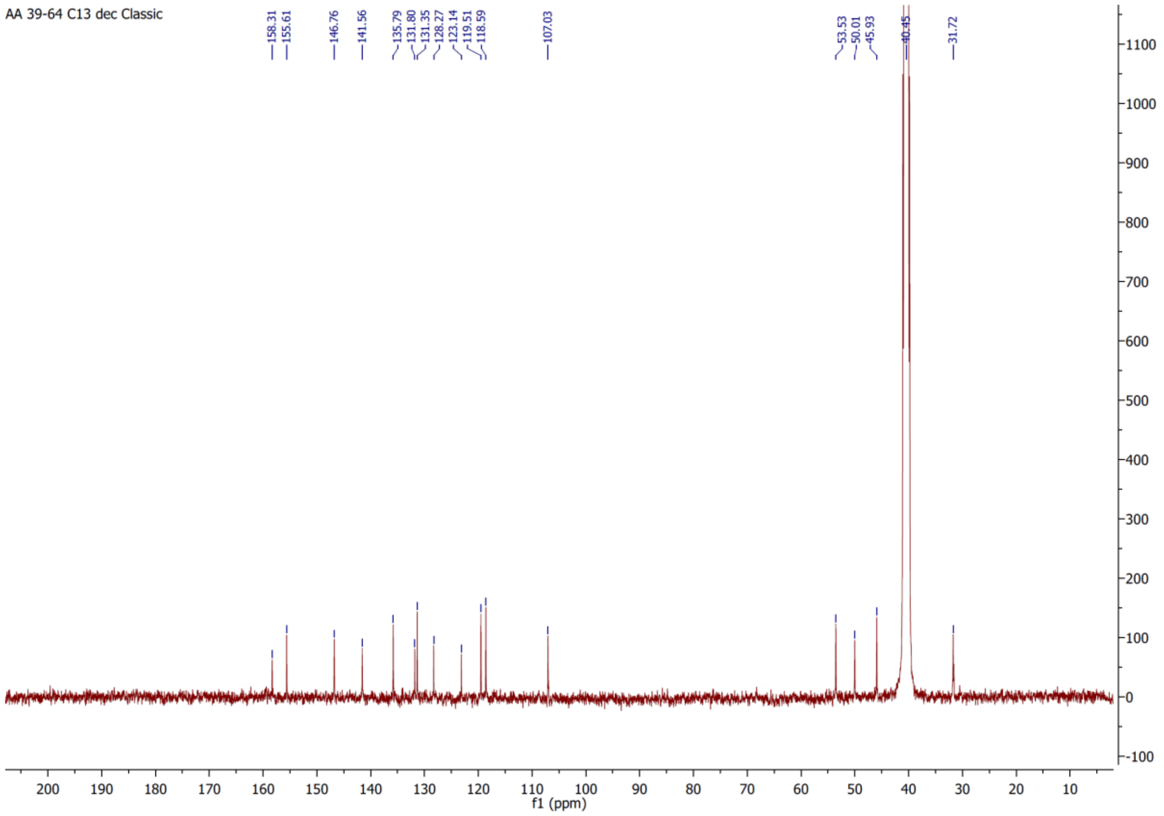

^{13}C NMR spectrum of compound **14b** (100 MHz, DMSO-d_6)

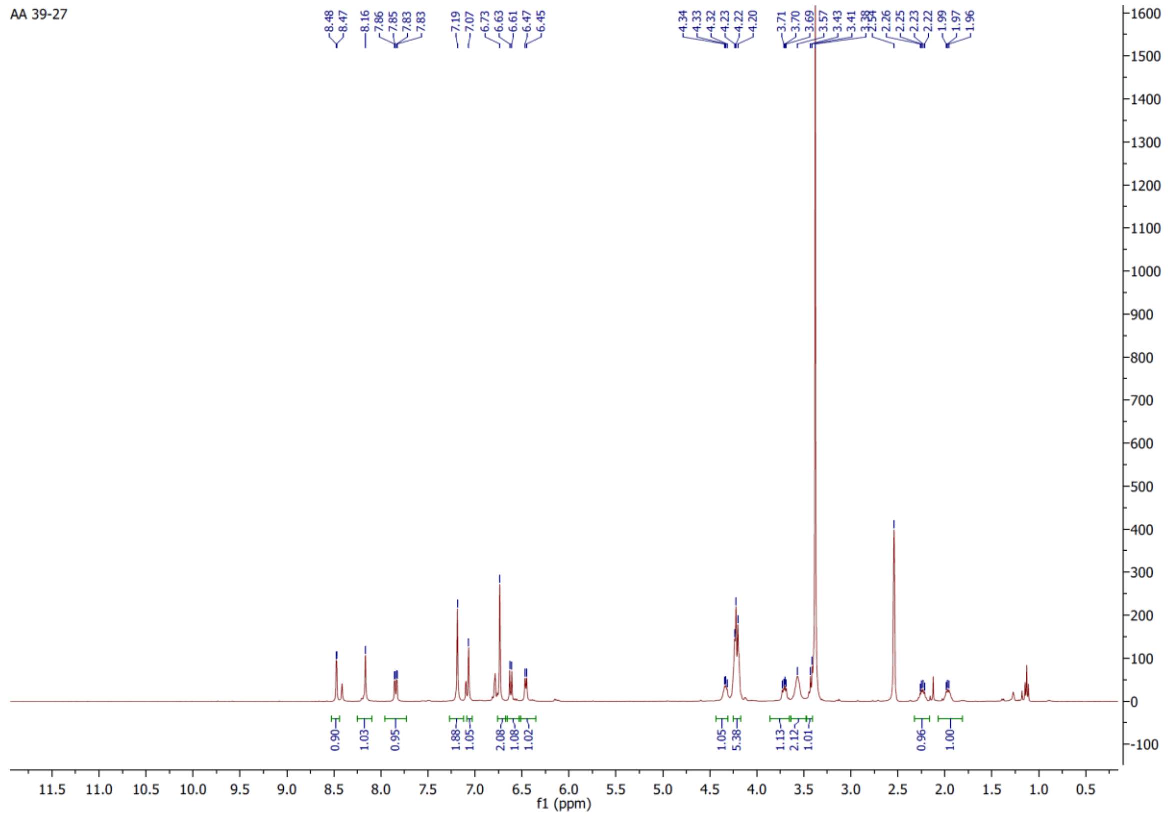

^{1}H NMR spectrum of compound **15a** (400 MHz, DMSO-d_6)

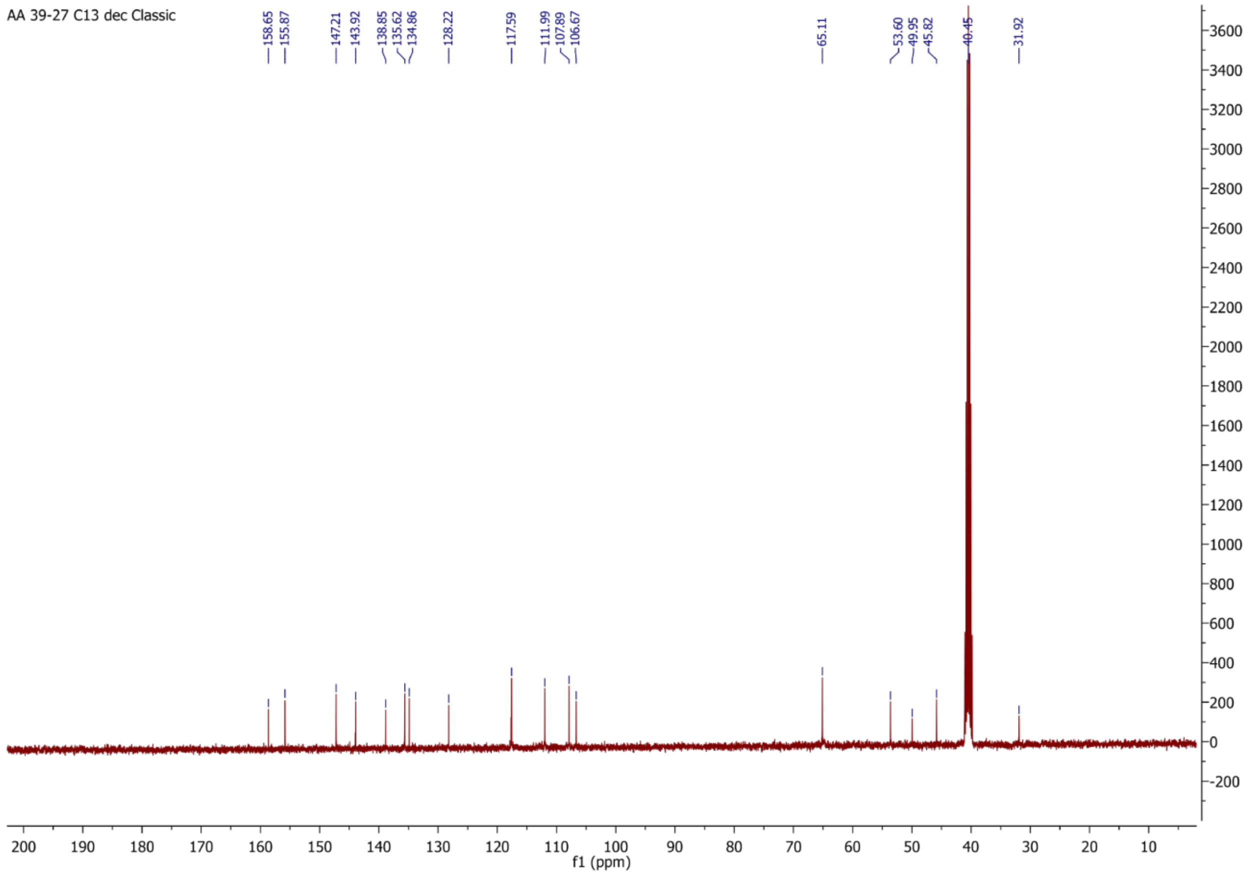

^{13}C NMR spectrum of compound **15a** (100 MHz, DMSO-d_6)

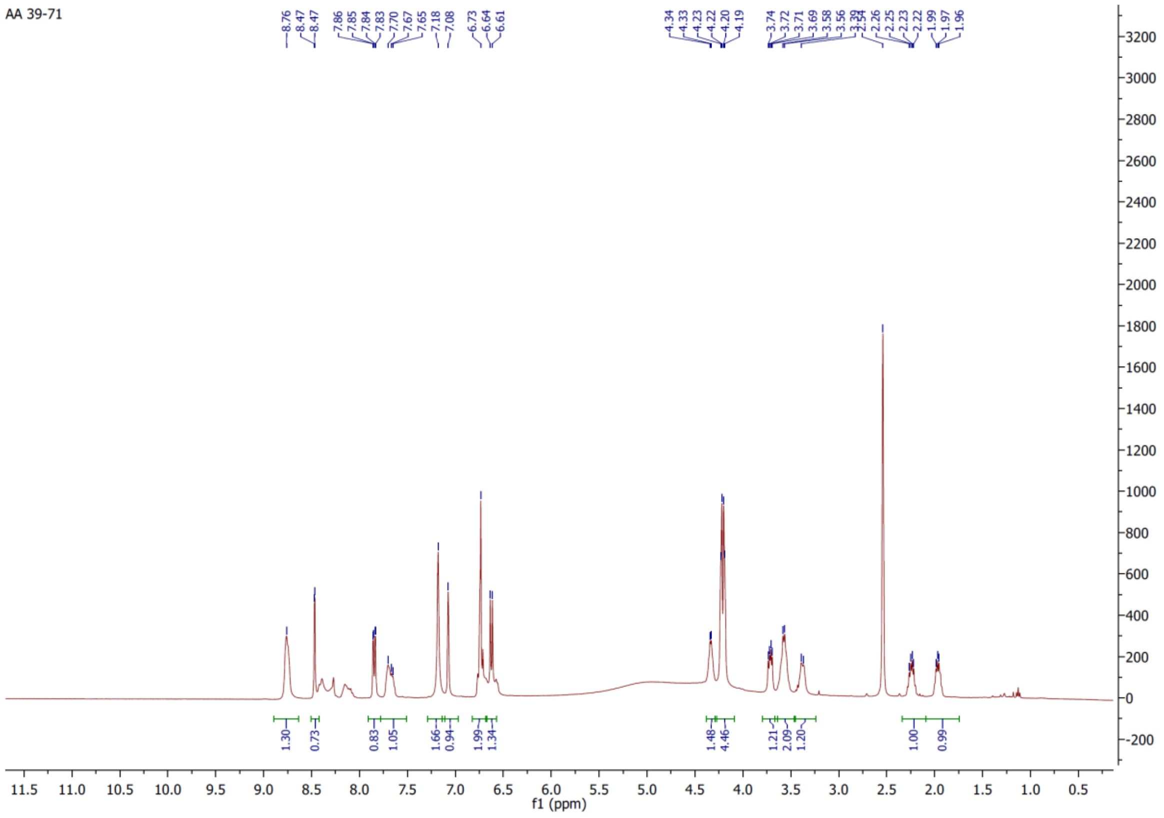

^{1}H NMR spectrum of compound **15b** (400 MHz, DMSO-d_6)

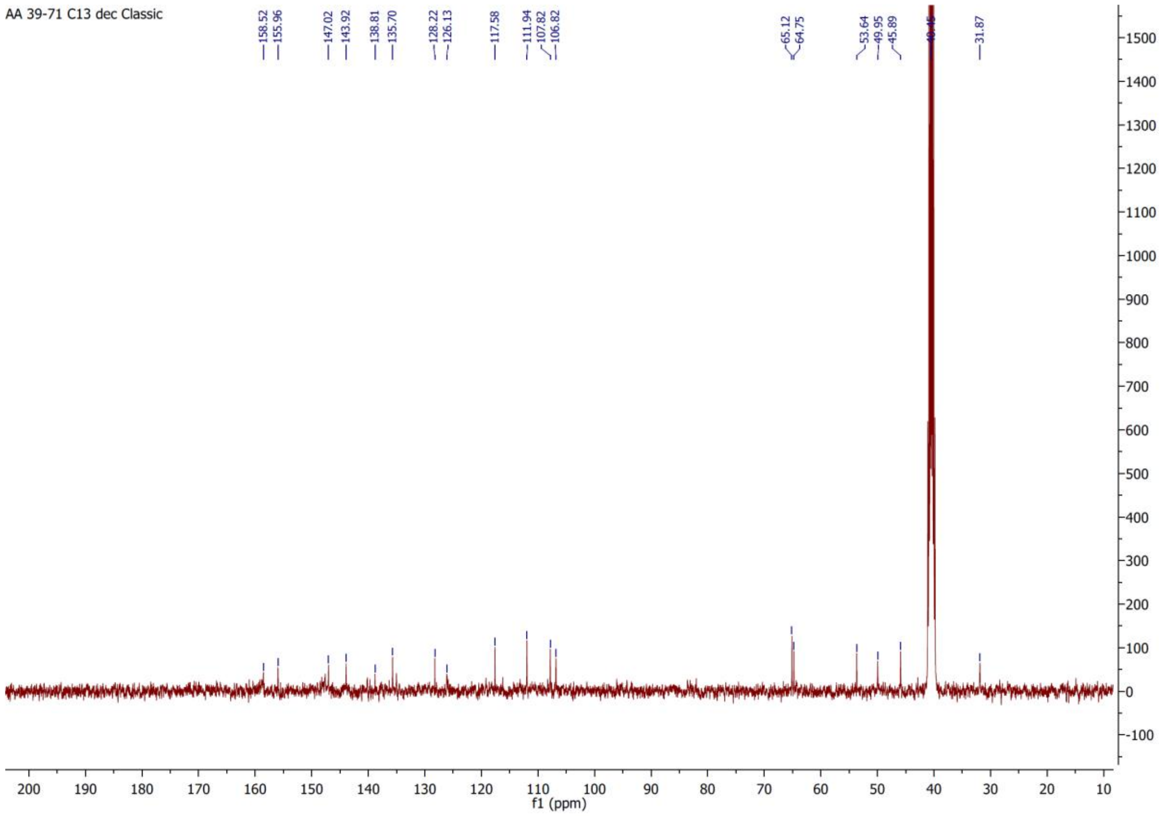

^{13}C NMR spectrum of compound **15b** (100 MHz, DMSO-d_6)

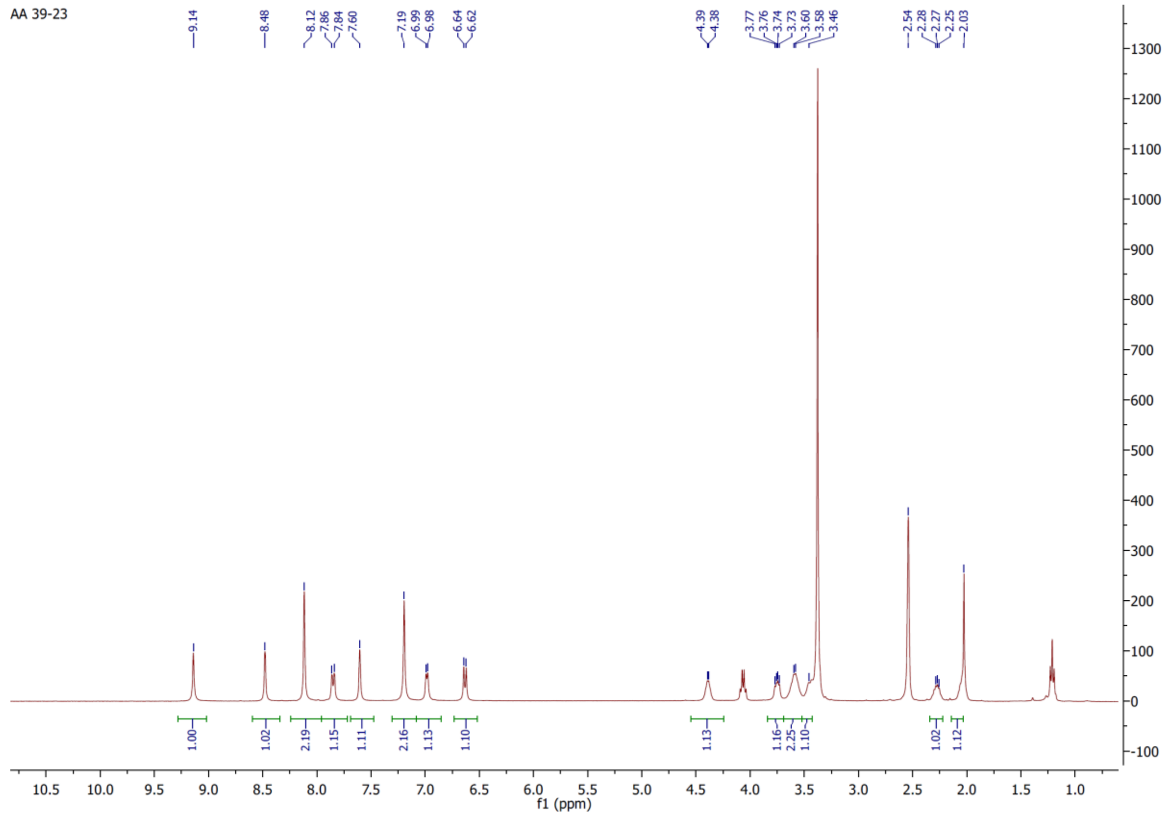

^{1}H NMR spectrum of compound **16a** (400 MHz, DMSO-d_6)

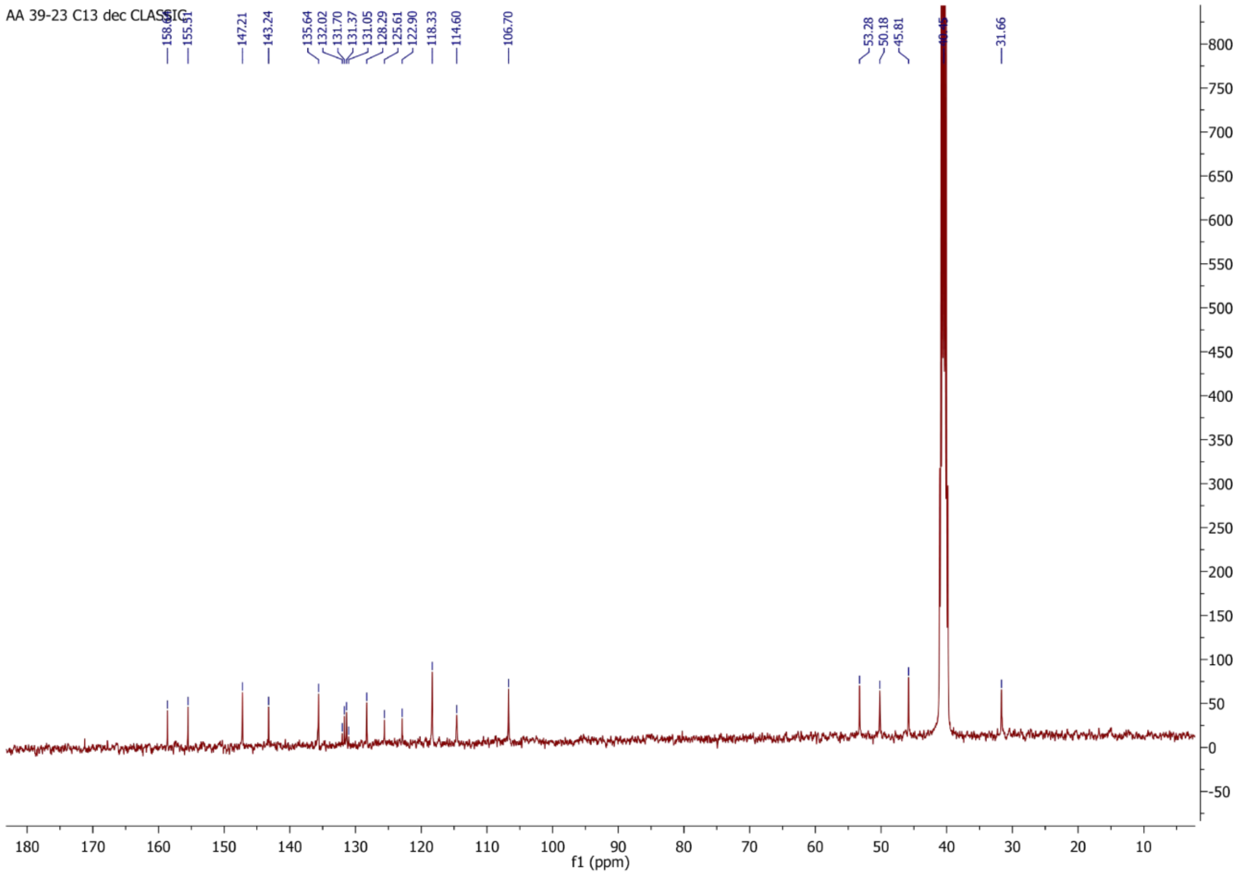

^{13}C NMR spectrum of compound **16a** (100 MHz, DMSO-d_6)

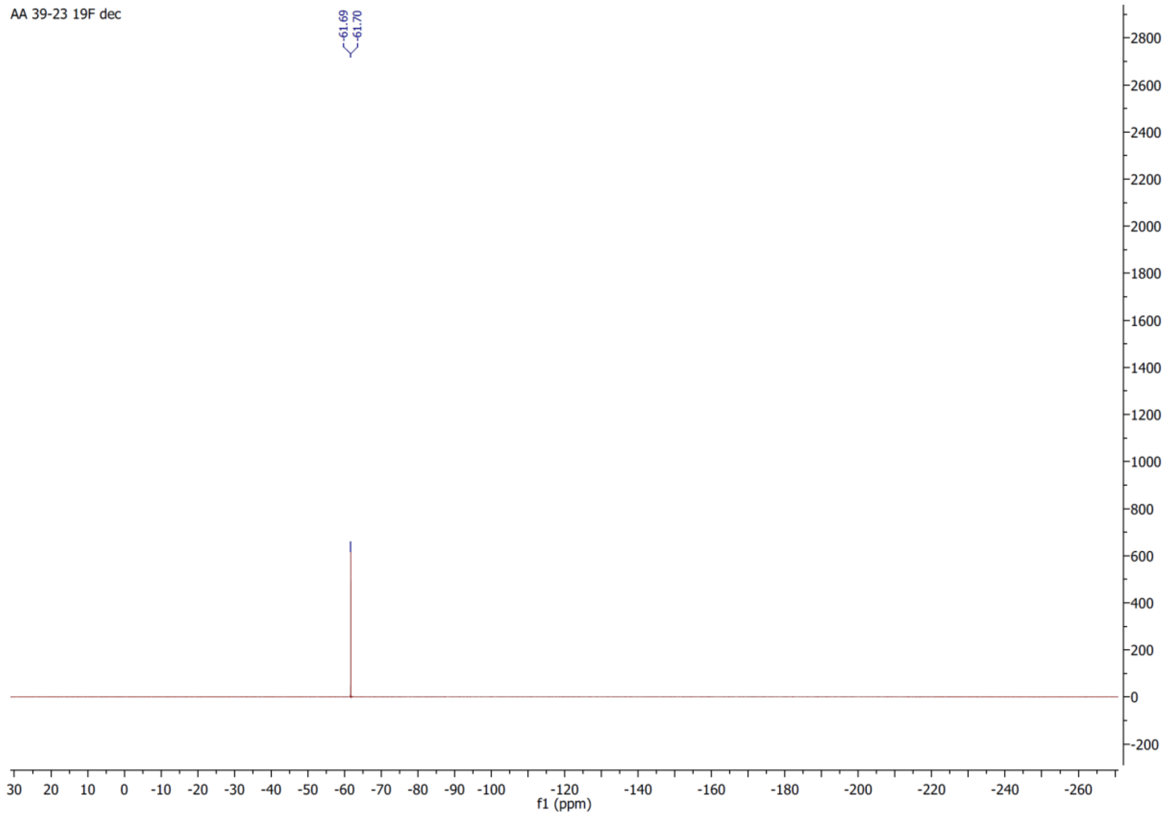

^{19}F NMR spectrum of compound **16a** (376 MHz, DMSO-d_6)

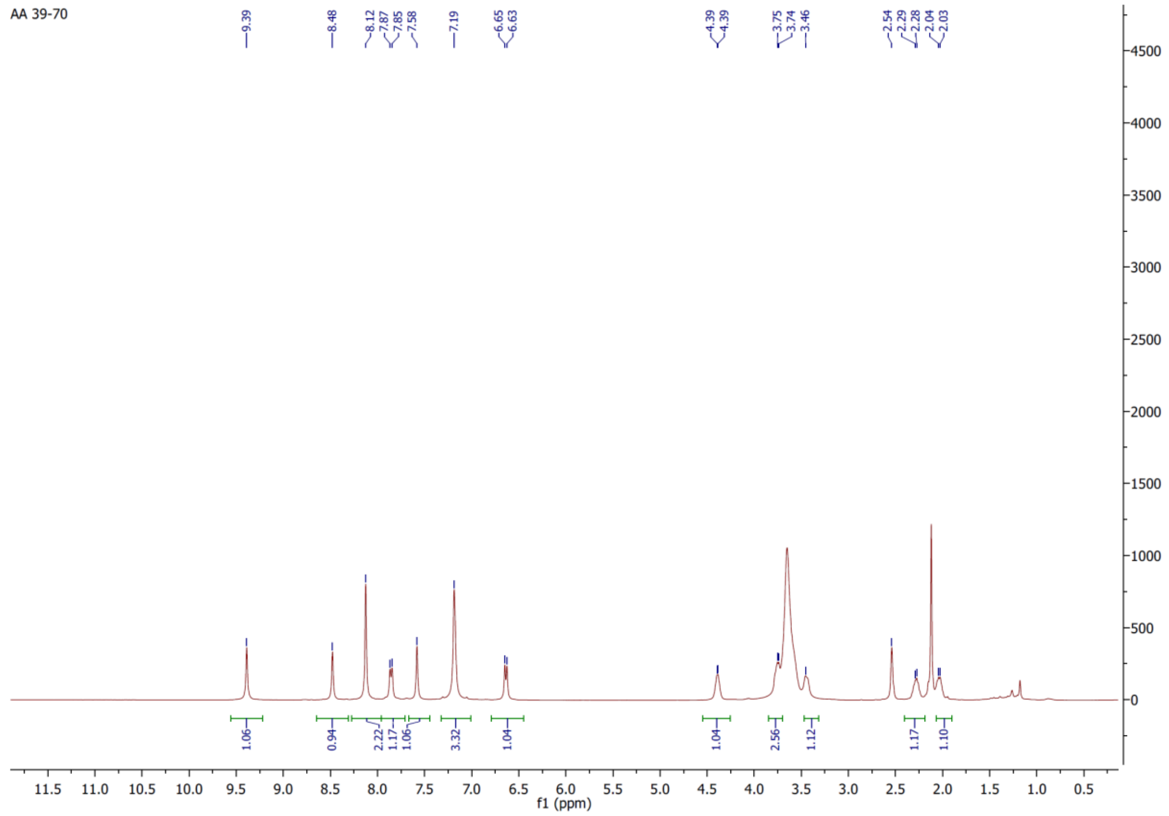

^{1}H NMR spectrum of compound **16b** (400 MHz, DMSO-d_6)

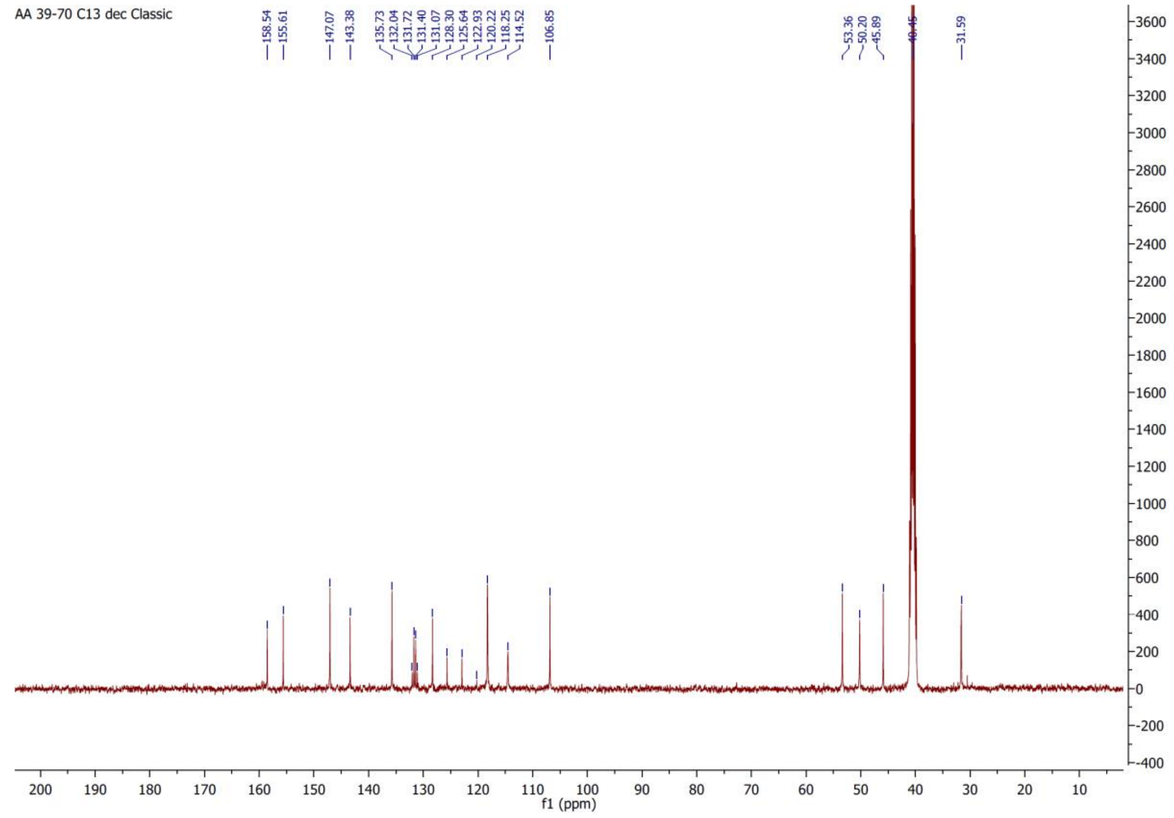

^{13}C NMR spectrum of compound **16b** (100 MHz, DMSO-d_6)

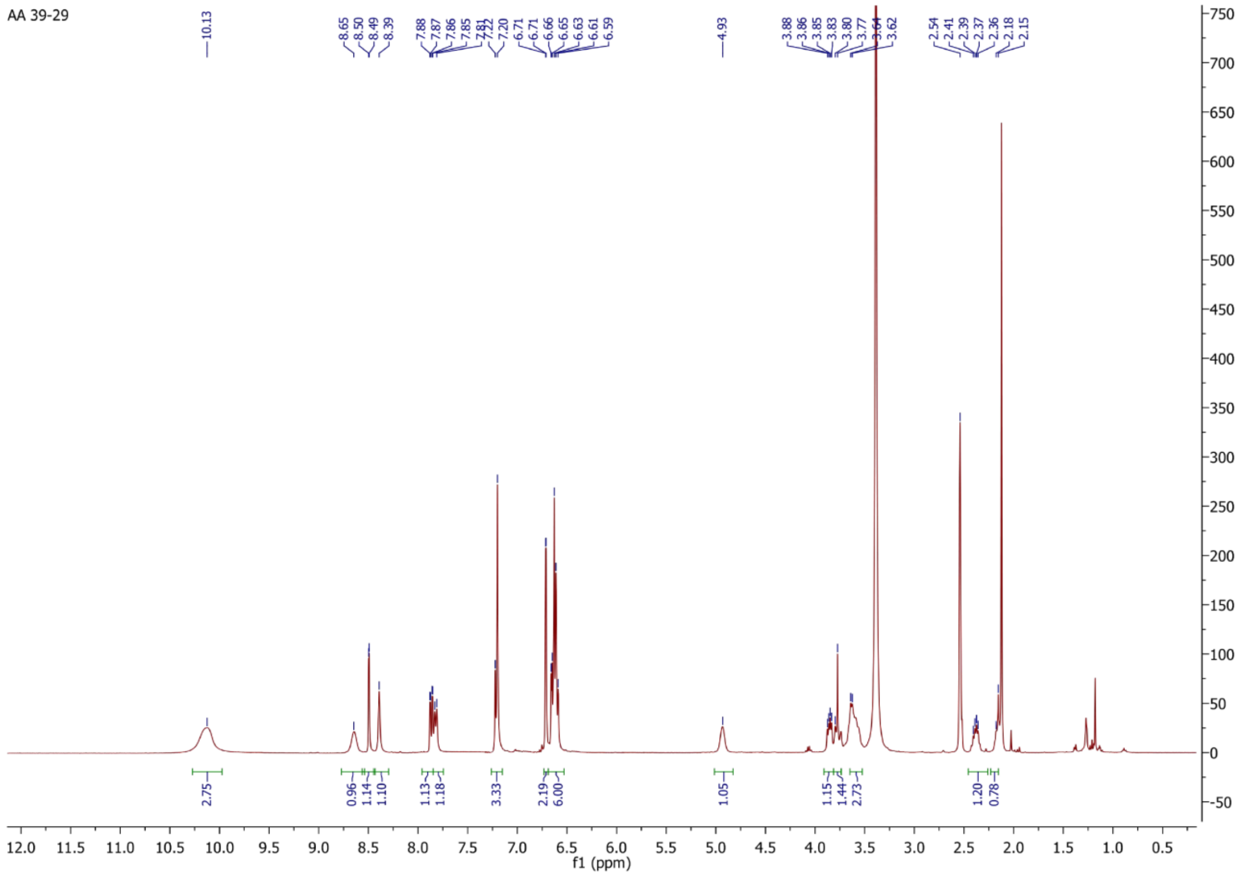

^{1}H NMR spectrum of compound **17a** (400 MHz, DMSO-d_6)

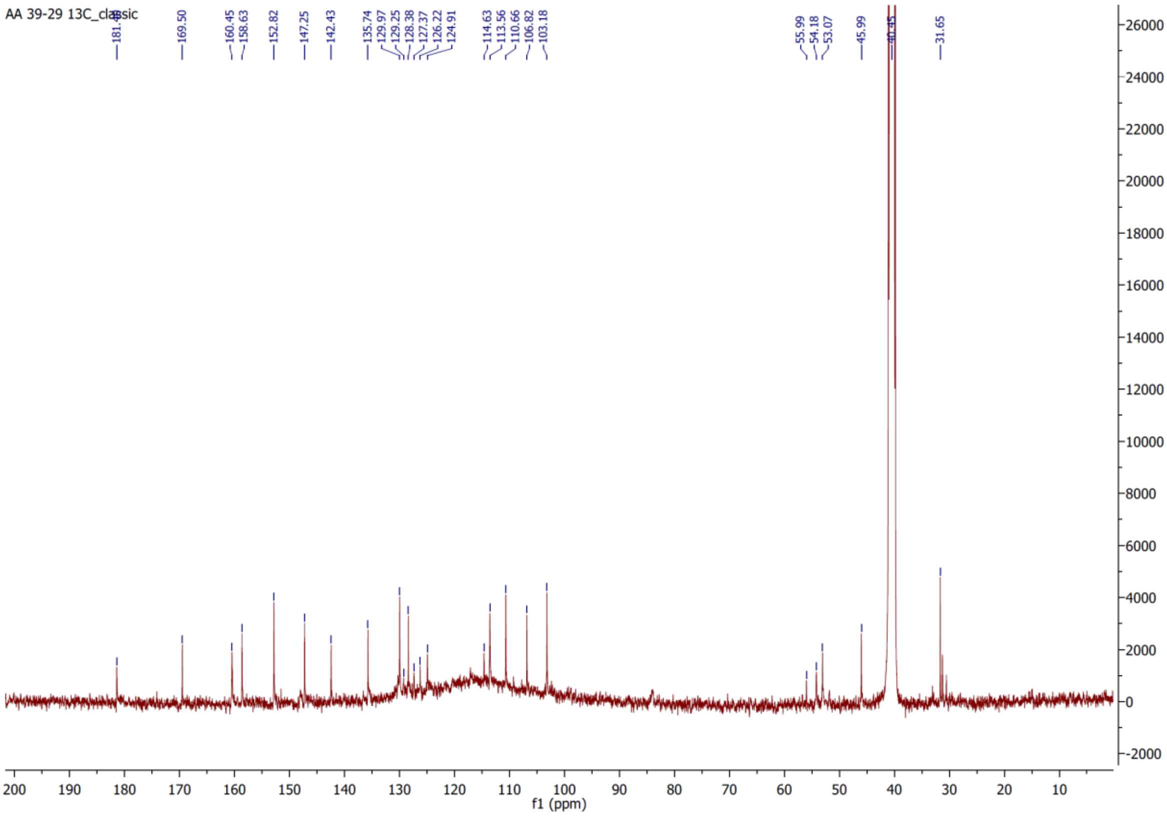

^{13}C NMR spectrum of compound **17a** (100 MHz, DMSO-d_6)

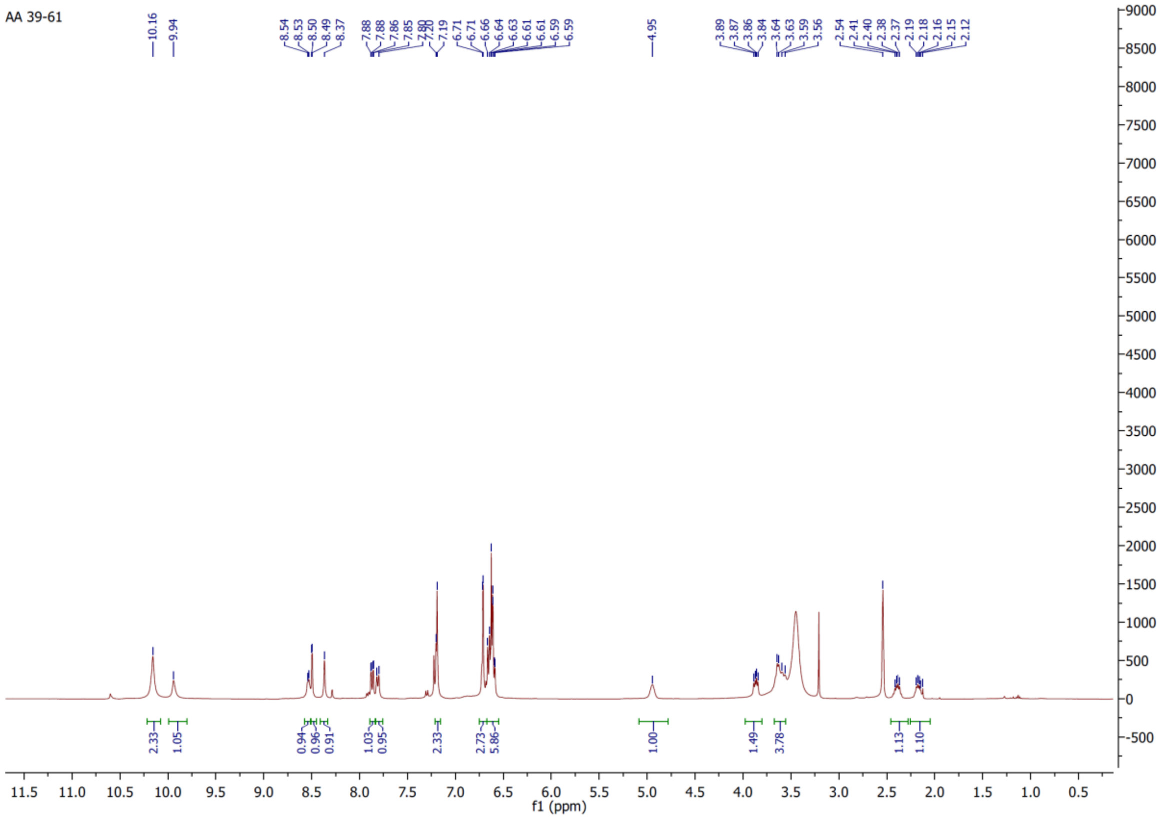

^{1}H NMR spectrum of compound **17b** (400 MHz, DMSO-d_6)

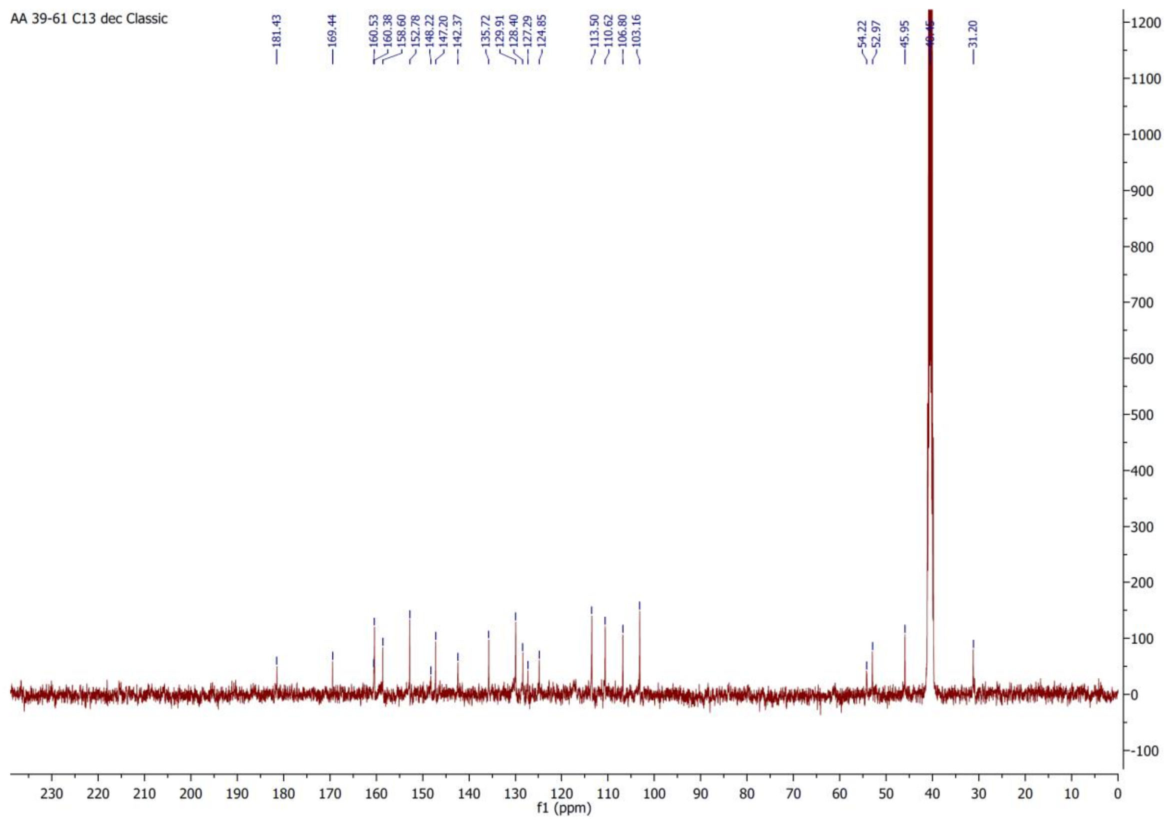

^{13}C NMR spectrum of compound **17b** (100 MHz, DMSO-d_6)

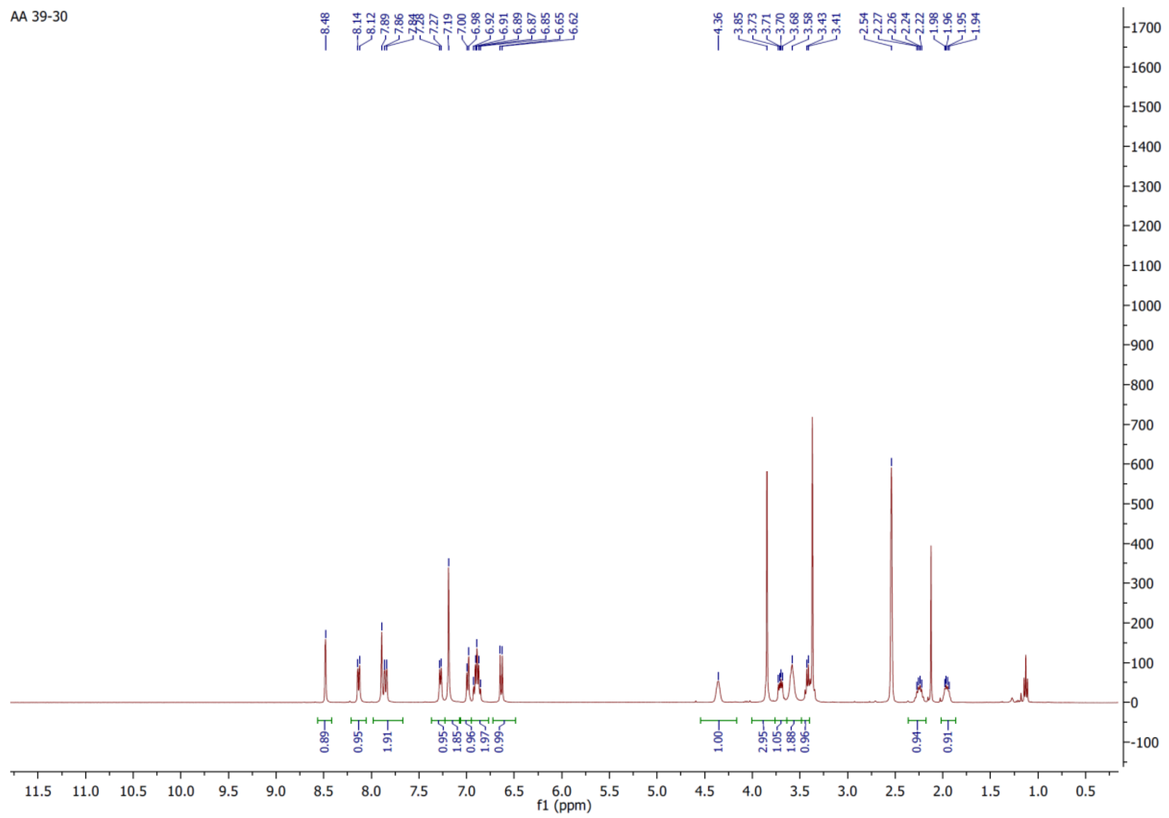

^{1}H NMR spectrum of compound **18a** (400 MHz, DMSO-d_6)

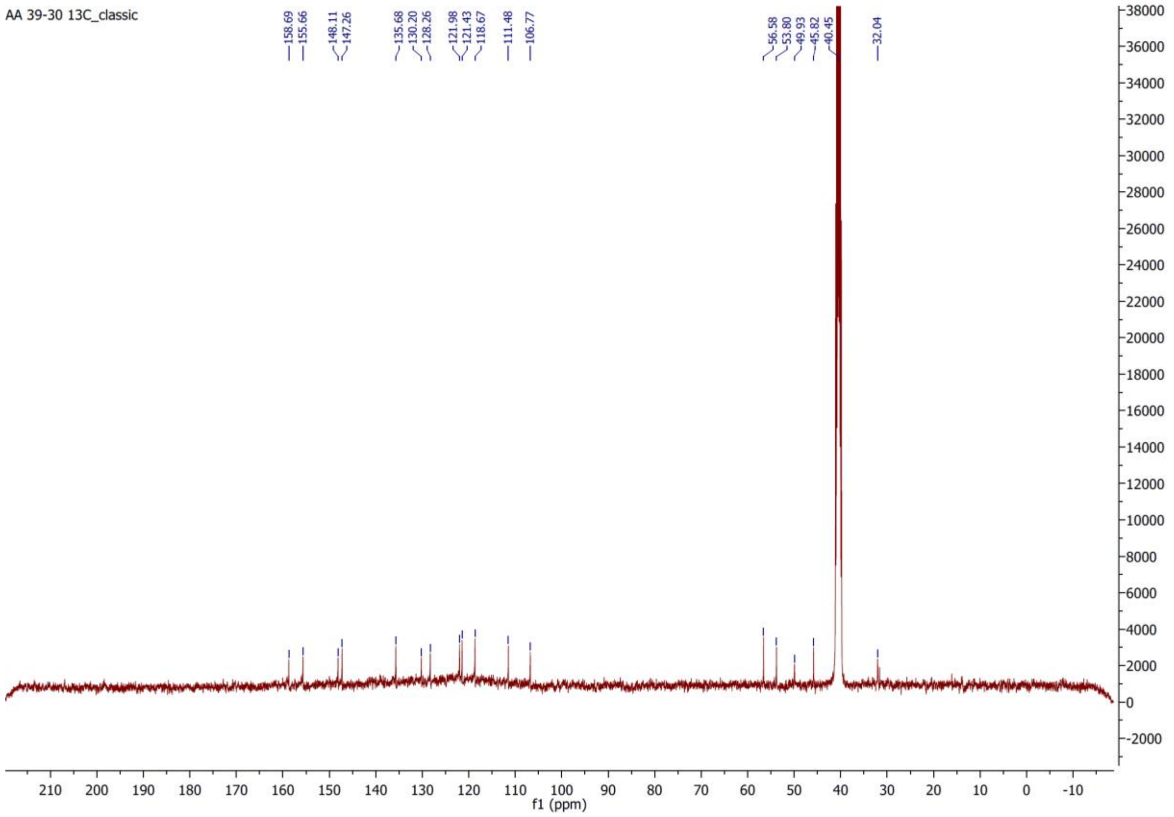

^{13}C NMR spectrum of compound **18a** (100 MHz, DMSO-d_6)

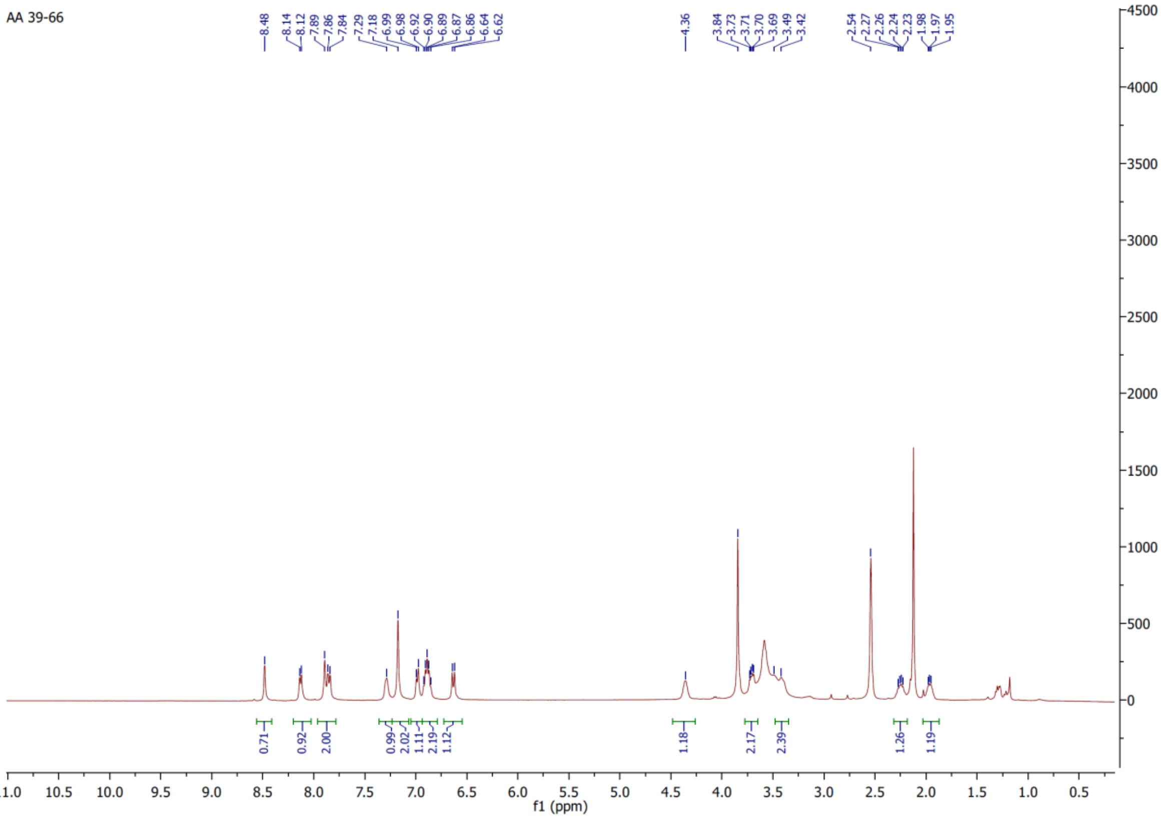

^{1}H NMR spectrum of compound **18b** (400 MHz, DMSO-d_6)

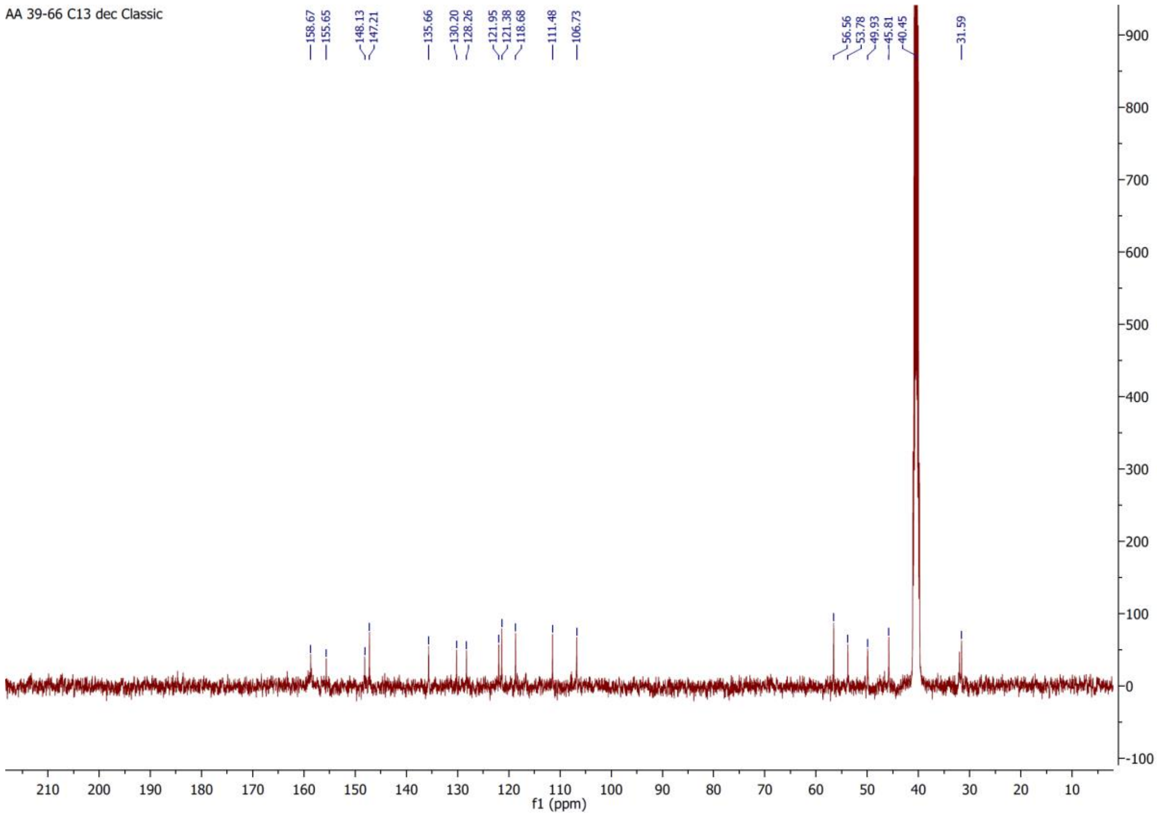

^{13}C NMR spectrum of compound **18b** (100 MHz, DMSO-d_6)

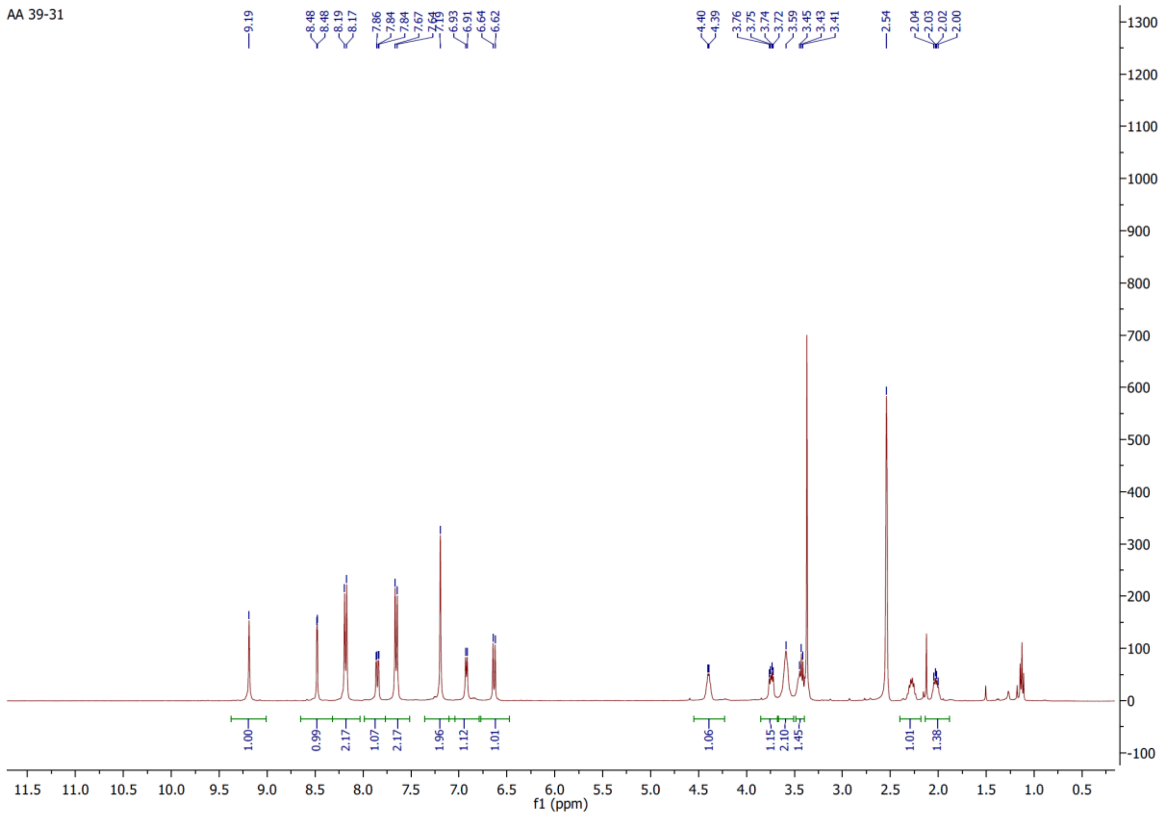

^{1}H NMR spectrum of compound **19a** (400 MHz, DMSO-d_6)

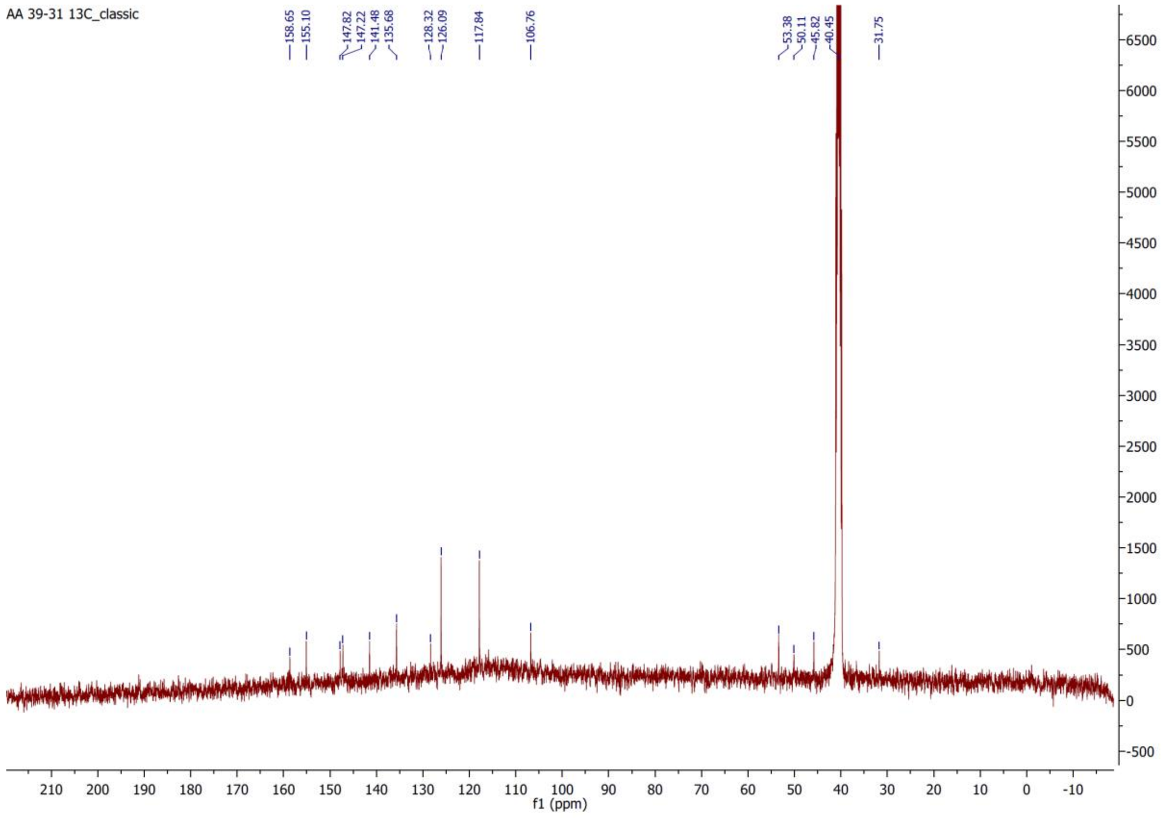

^{13}C NMR spectrum of compound **19a** (100 MHz, DMSO-d_6)

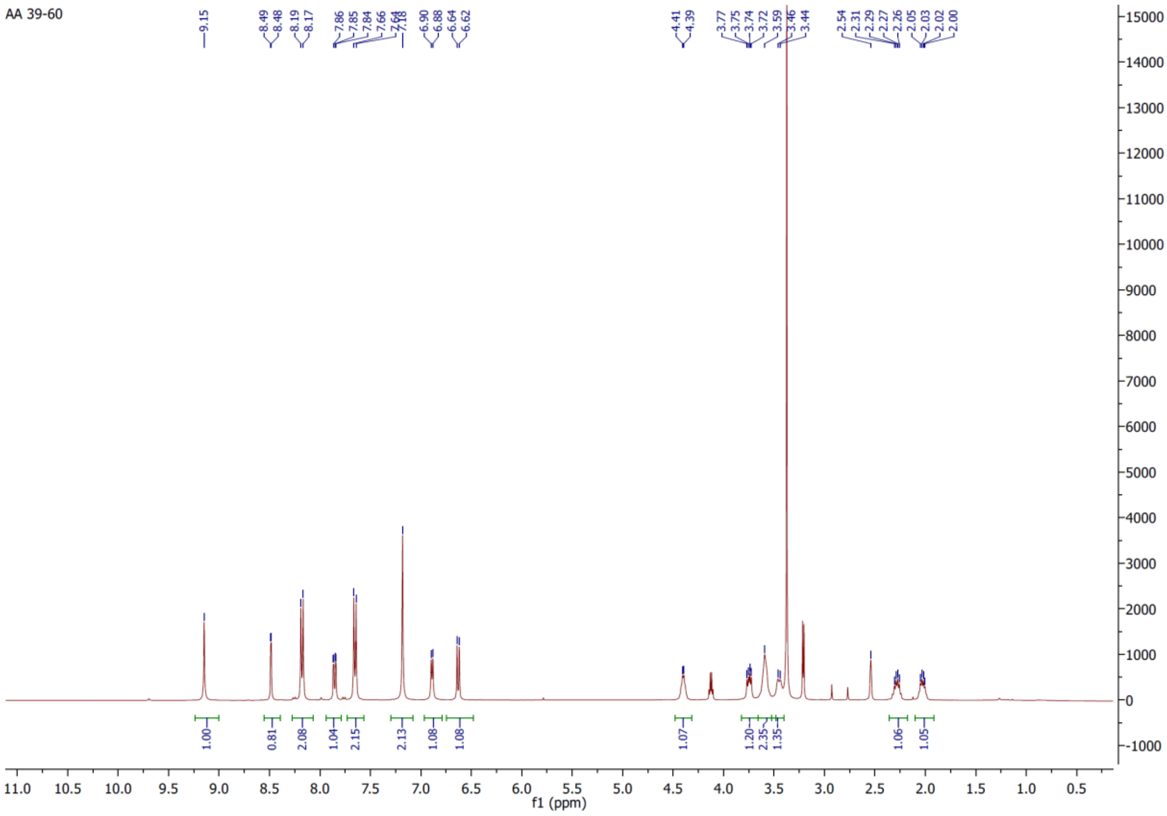

^{1}H NMR spectrum of compound **19b** (400 MHz, DMSO-d_6)

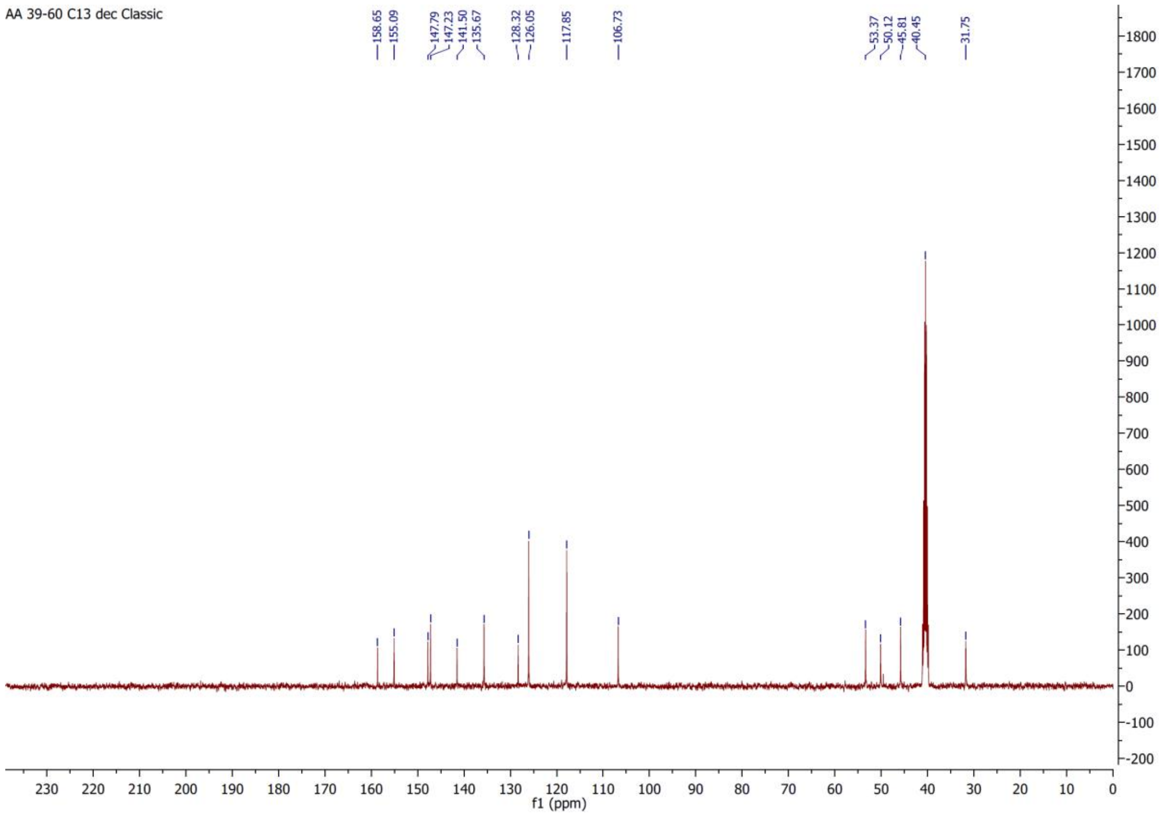

^{13}C NMR spectrum of compound **19b** (100 MHz, DMSO-d_6)

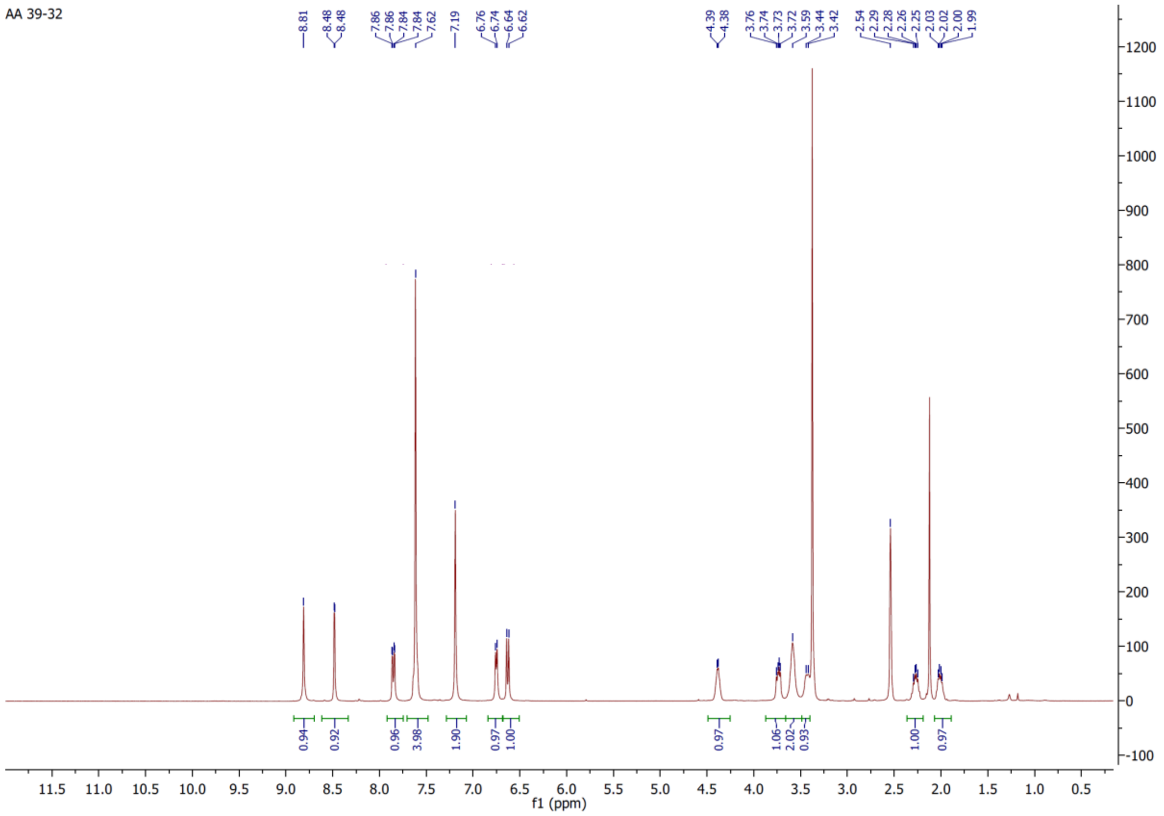

^{1}H NMR spectrum of compound **20a** (400 MHz, DMSO-d_6)

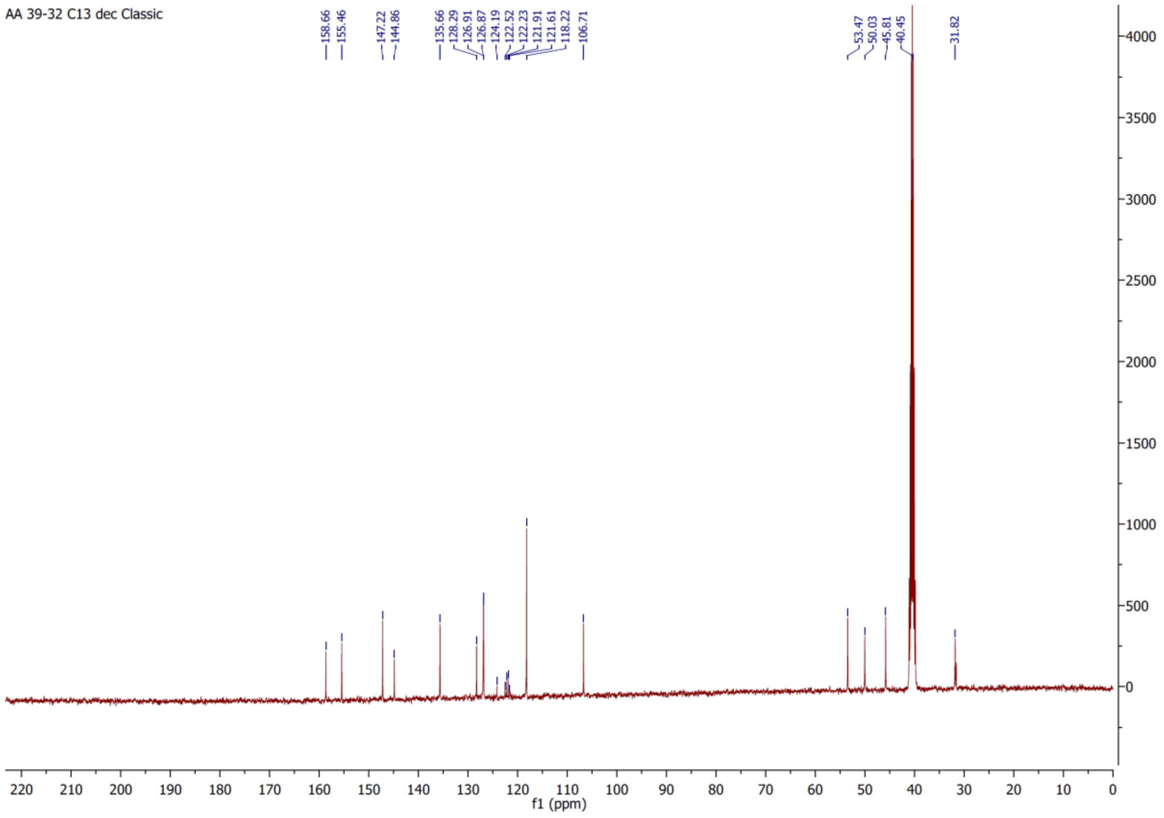

^{13}C NMR spectrum of compound **20a** (100 MHz, DMSO-d_6)

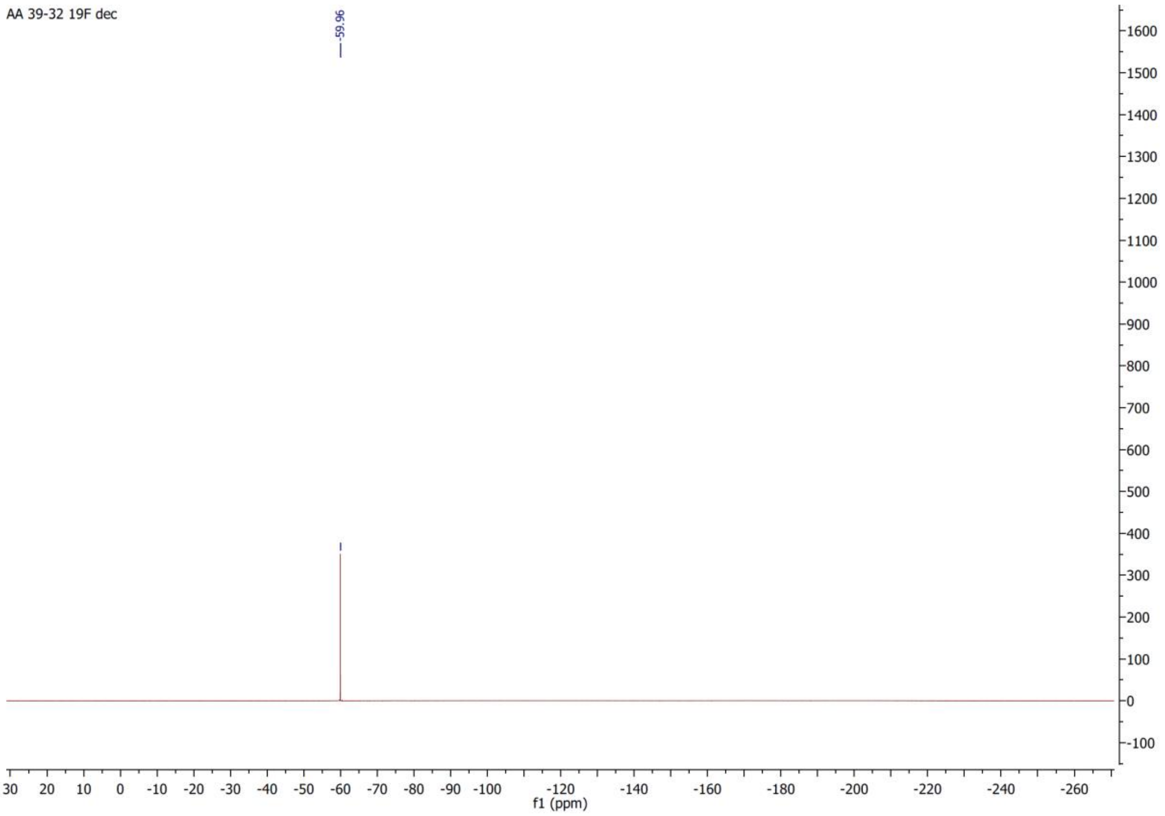

^{19}F NMR spectrum of compound **20a** (376 MHz, DMSO-d_6)

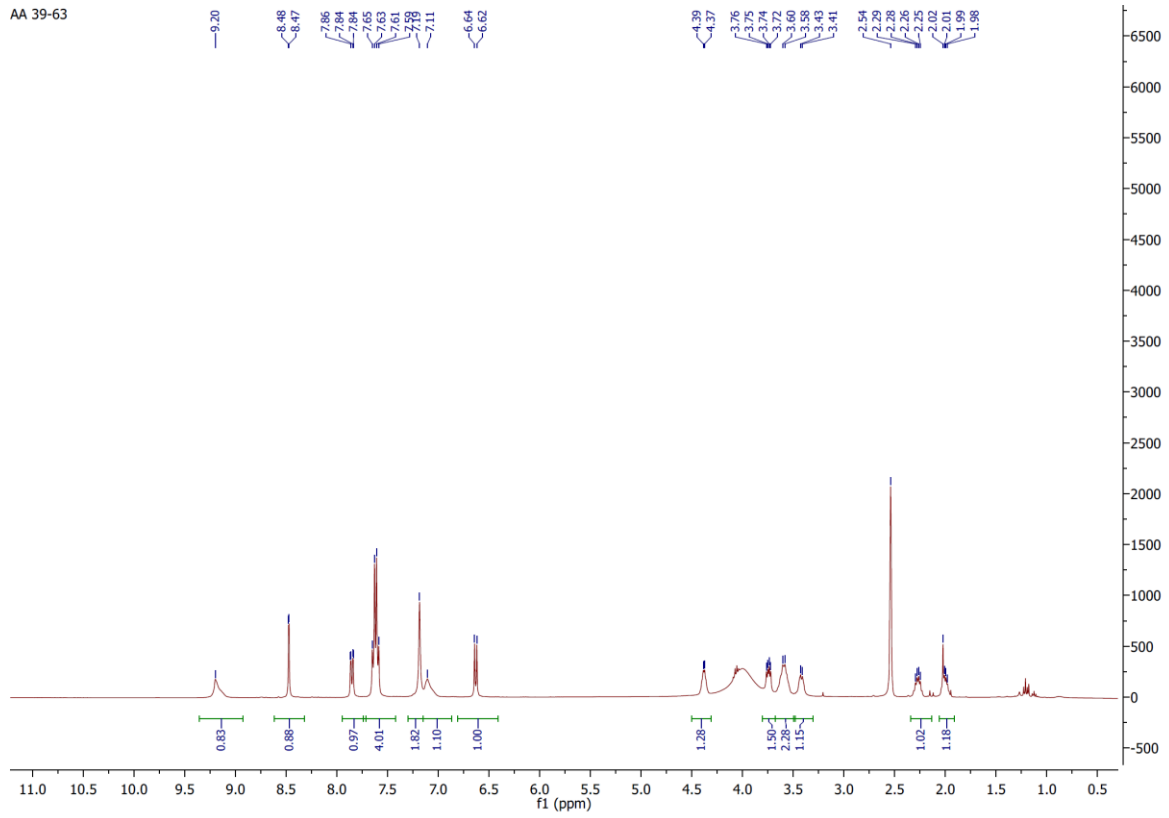

^{1}H NMR spectrum of compound **20b** (400 MHz, DMSO-d_6)

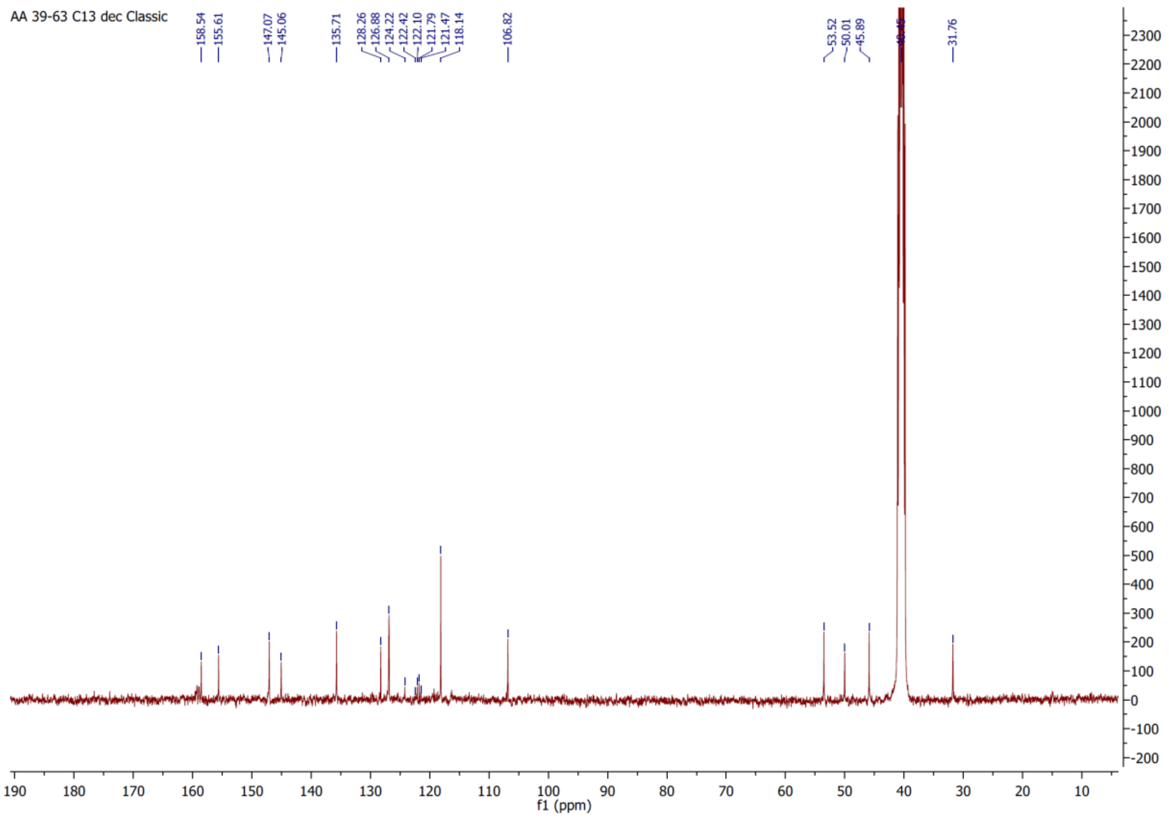

^{13}C NMR spectrum of compound **20b** (100 MHz, DMSO-d_6)

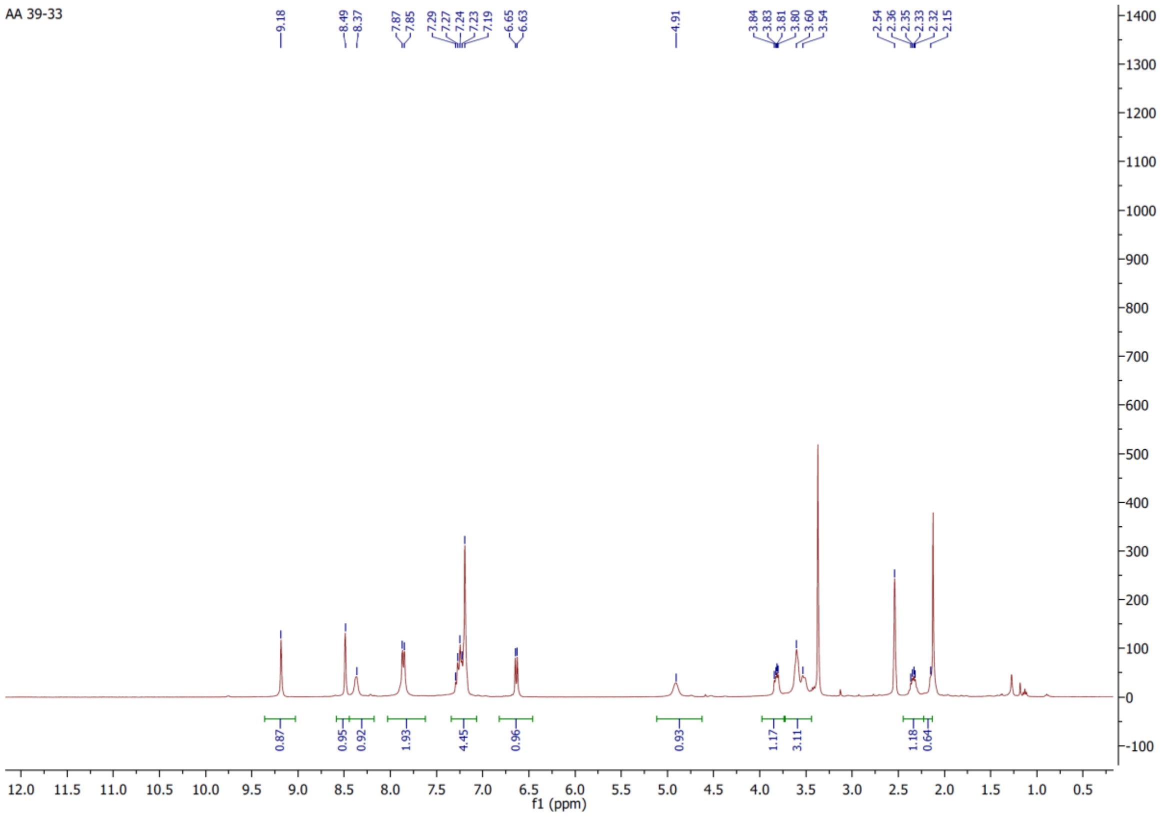

^{1}H NMR spectrum of compound **21a** (400 MHz, DMSO-d_6)

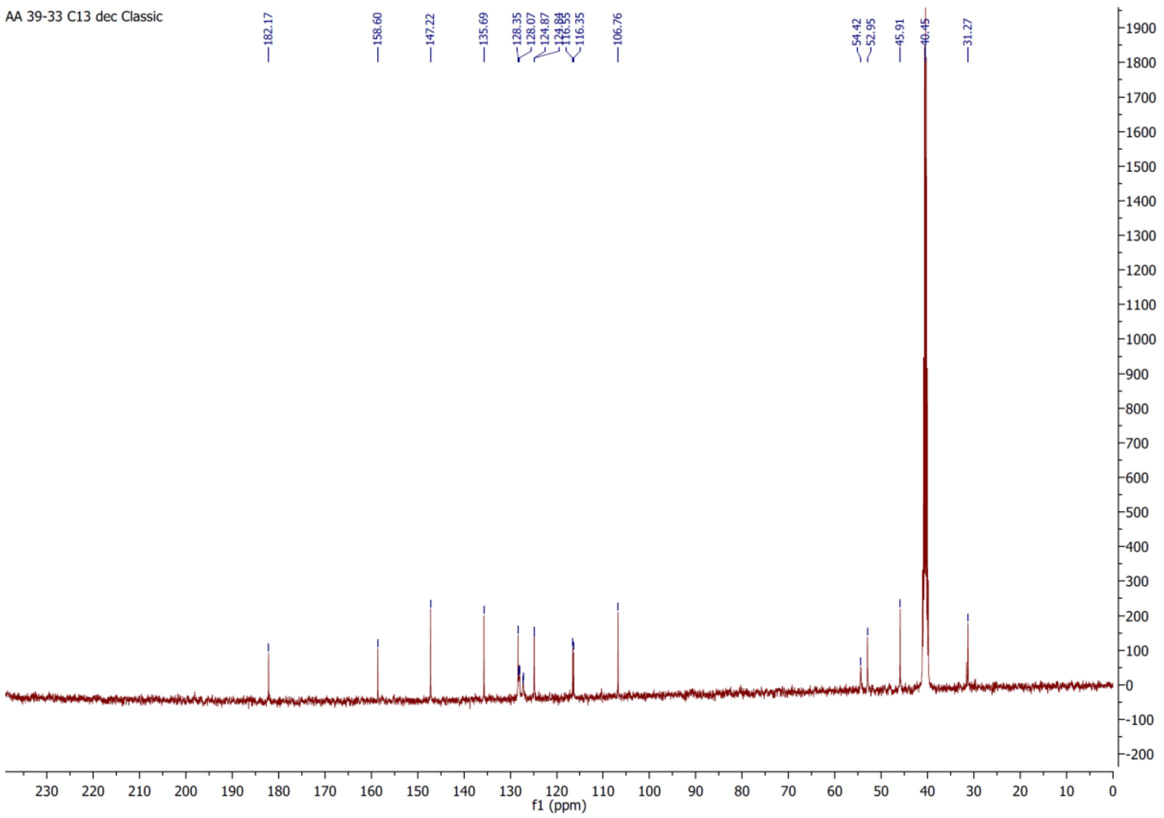

^{13}C NMR spectrum of compound **21a** (100 MHz, DMSO-d_6)

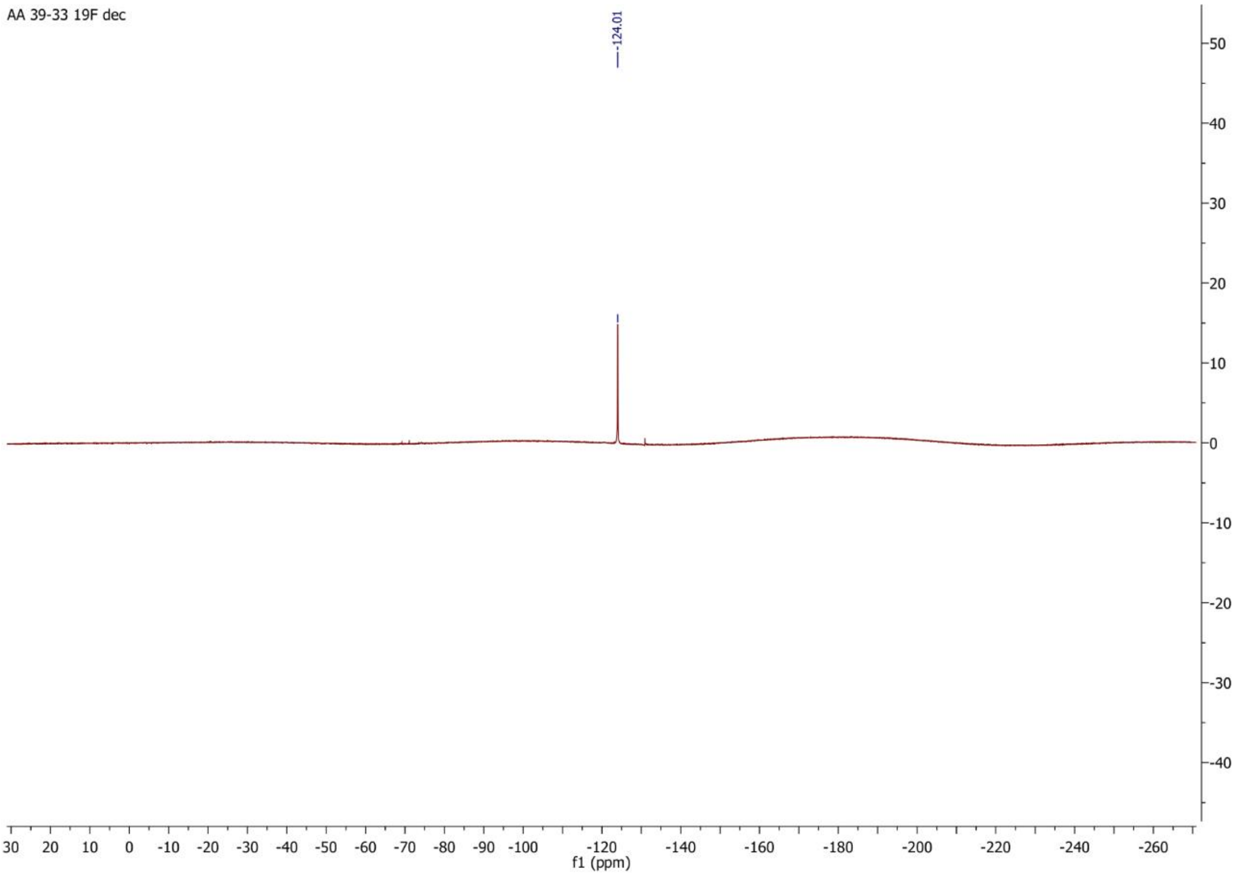

^{19}F NMR spectrum of compound **21a** (376 MHz, DMSO-d_6)

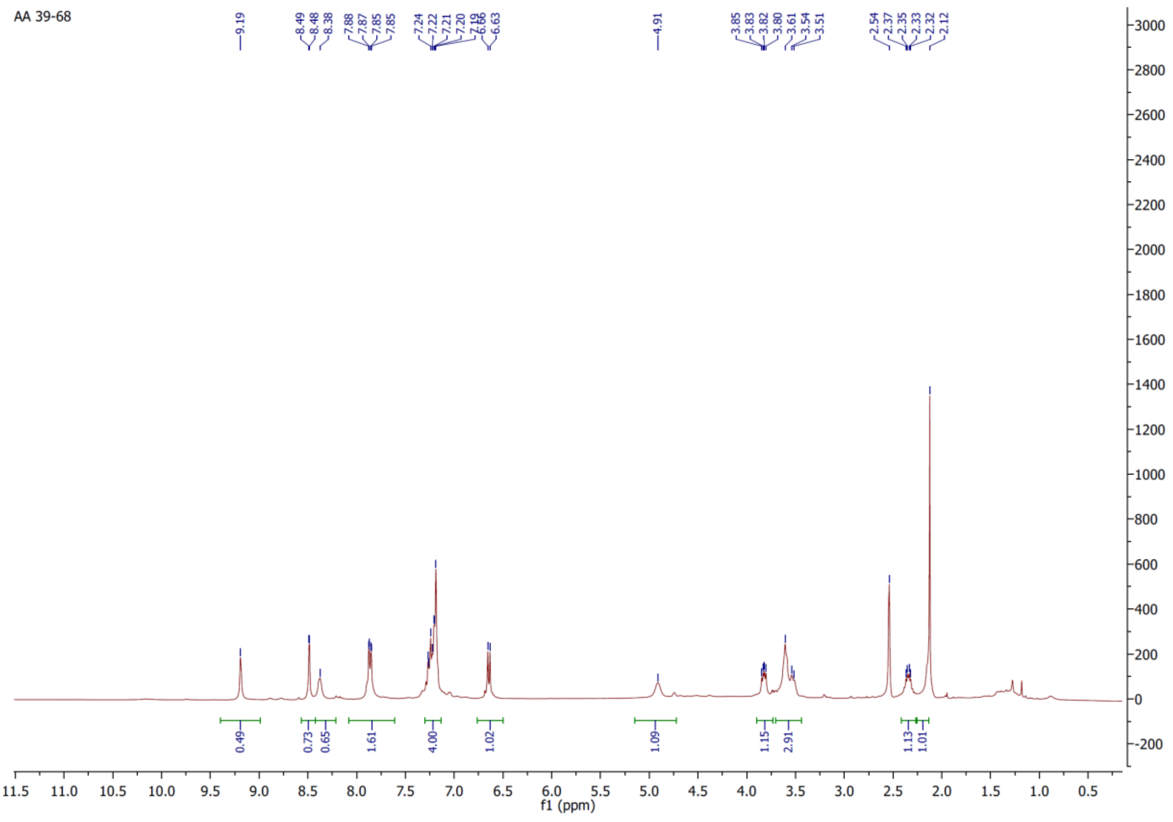

^{1}H NMR spectrum of compound **21b** (400 MHz, DMSO-d_6)

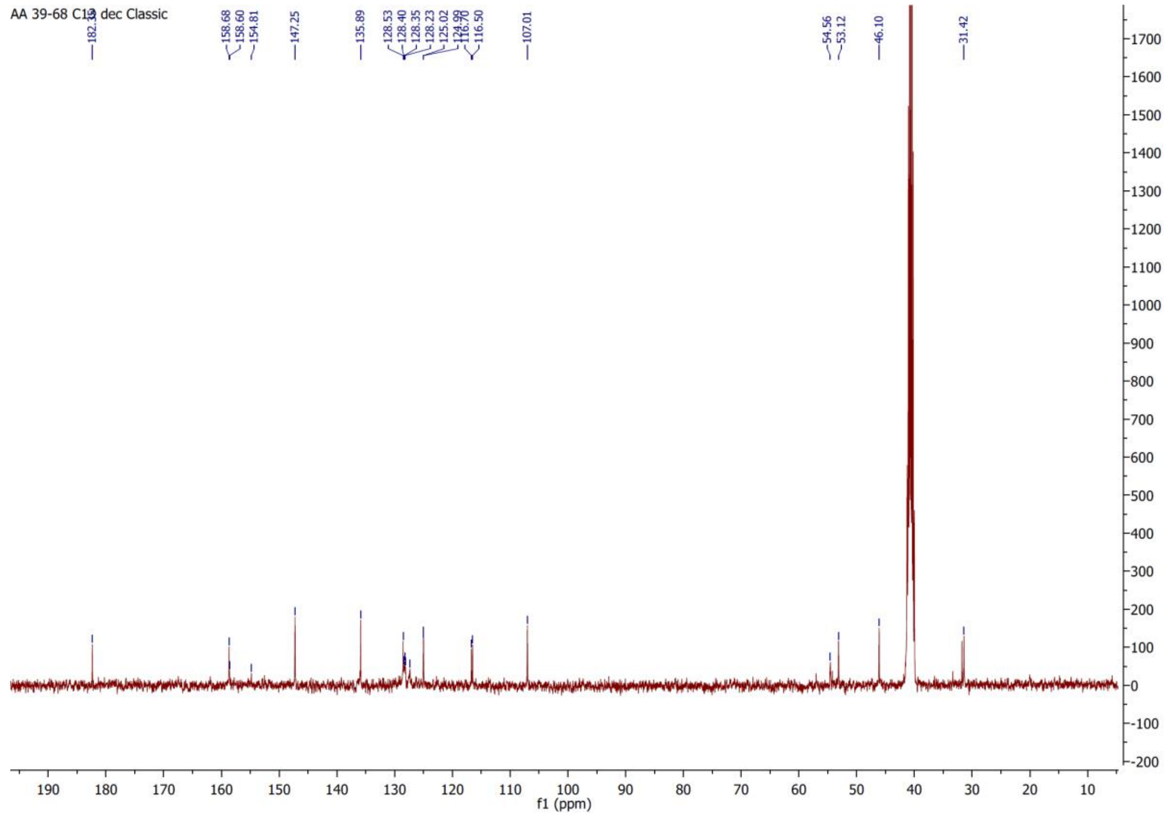

^{13}C NMR spectrum of compound **21b** (100 MHz, DMSO-d_6)

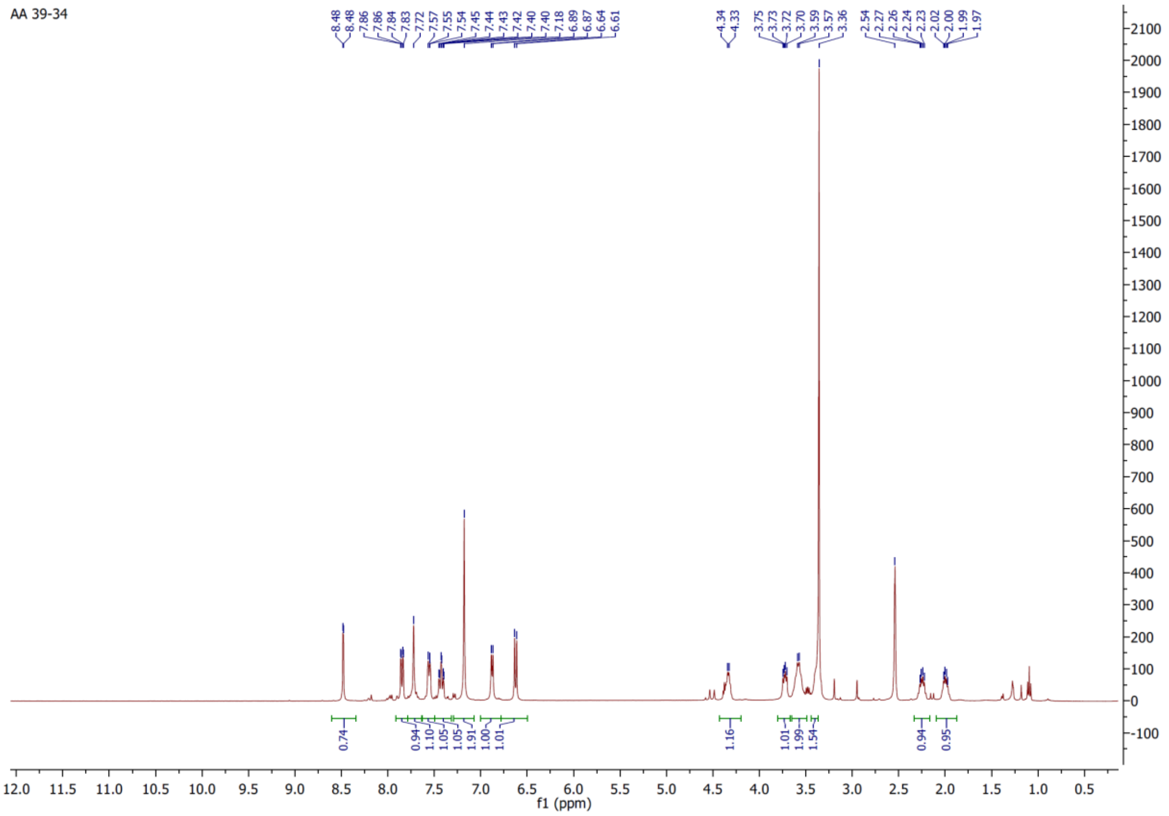

^{1}H NMR spectrum of compound **22a** (400 MHz, DMSO-d_6)

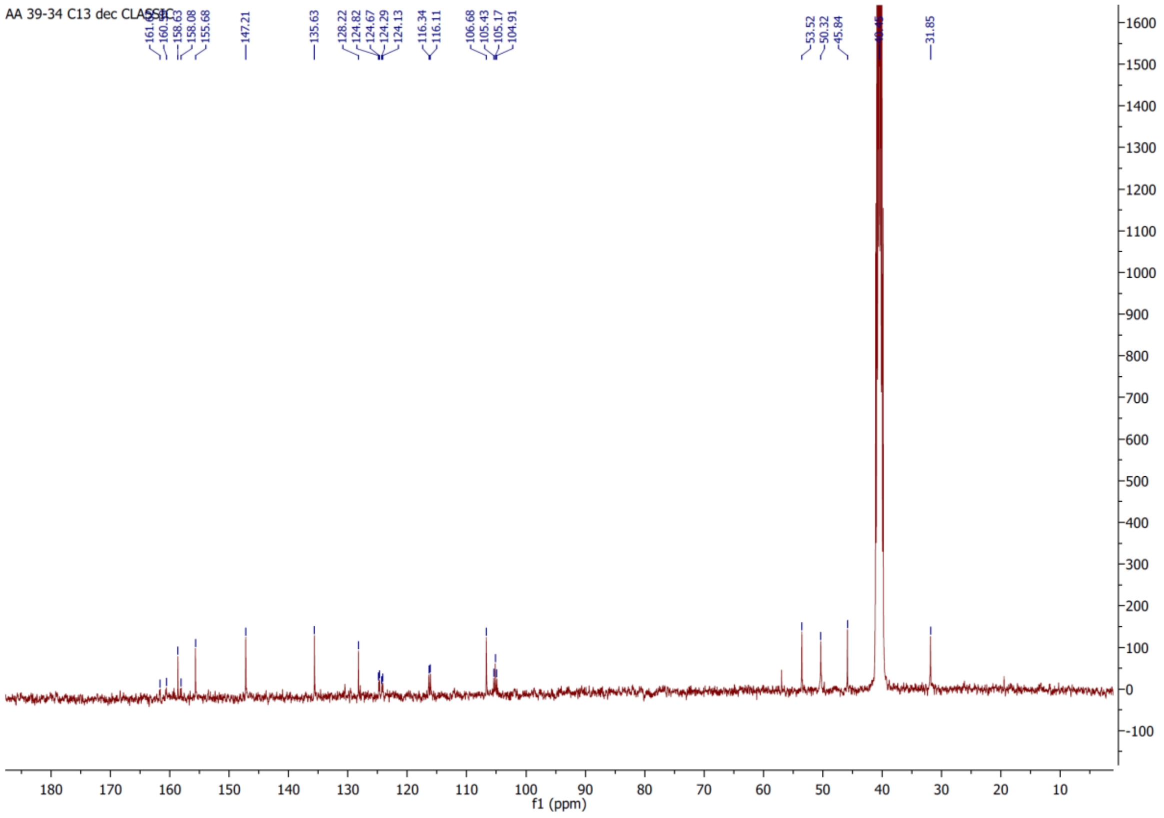

^{13}C NMR spectrum of compound **22a** (100 MHz, DMSO-d_6)

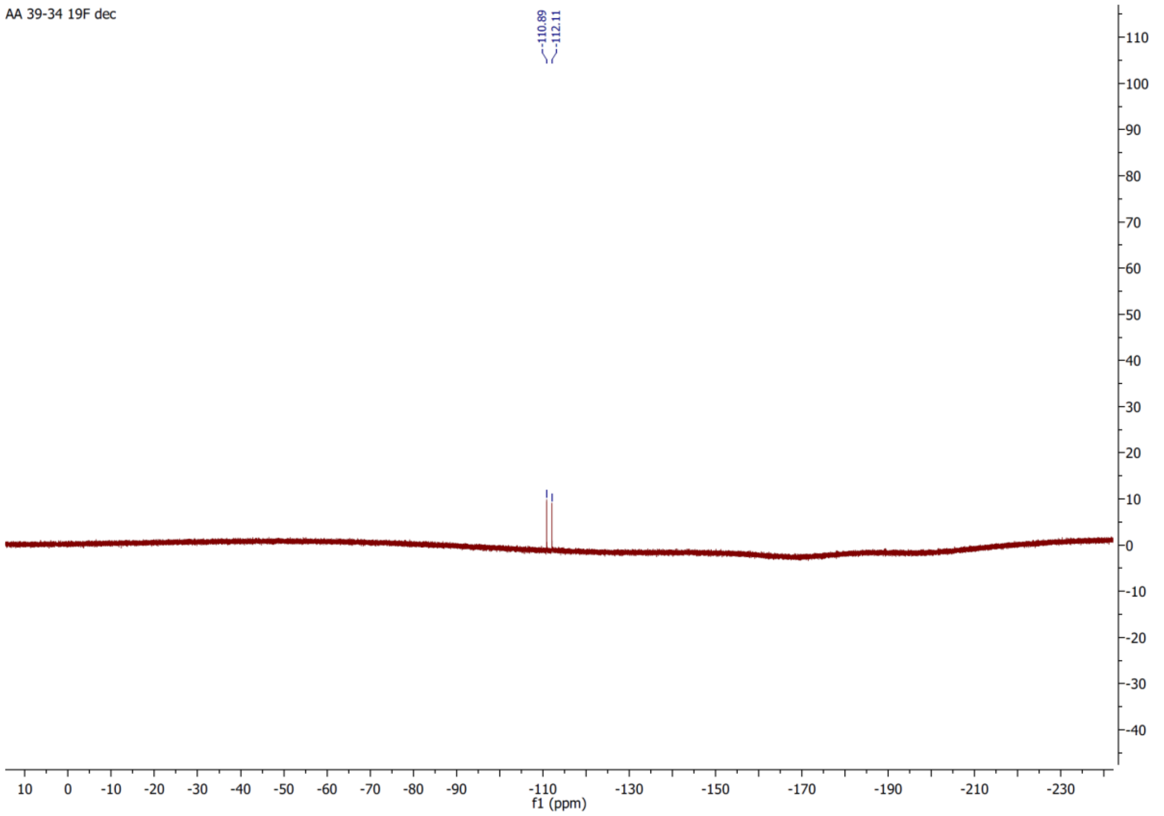

^{19}F NMR spectrum of compound **22a** (376 MHz, DMSO-d_6)

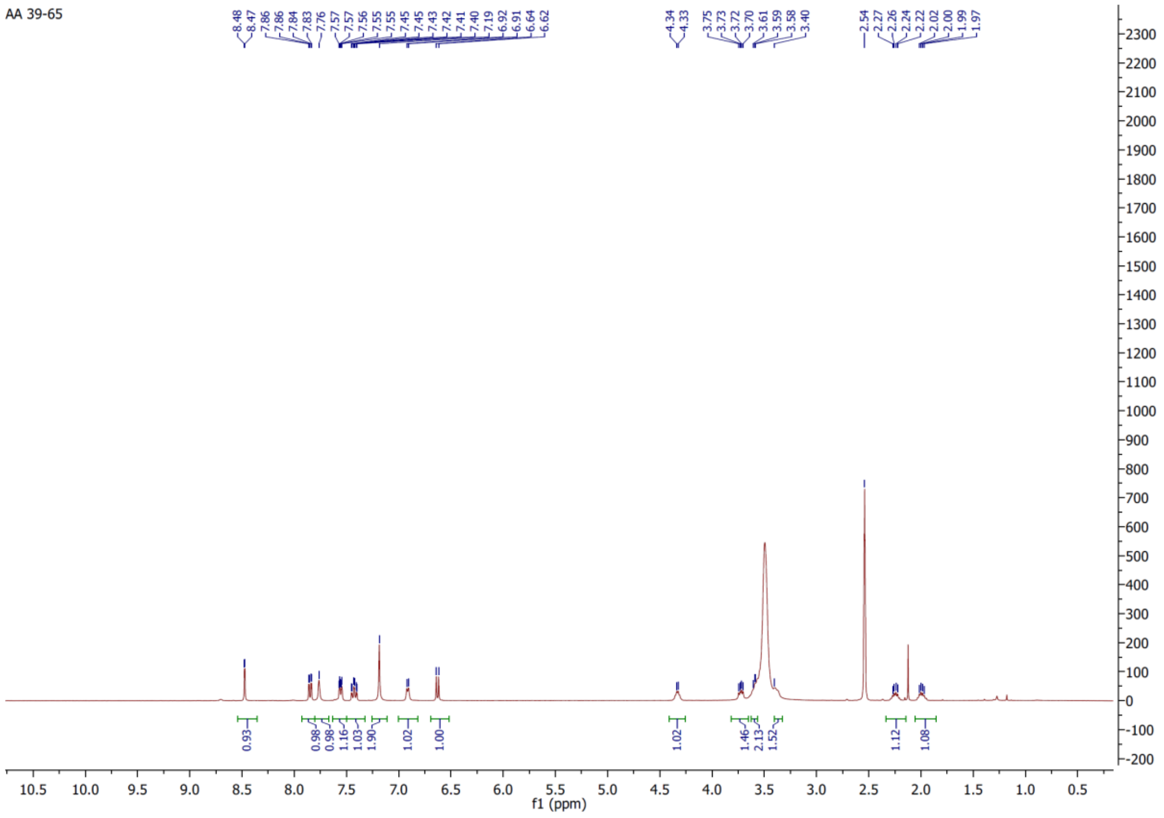

^{1}H NMR spectrum of compound **22b** (400 MHz, DMSO-d_6)

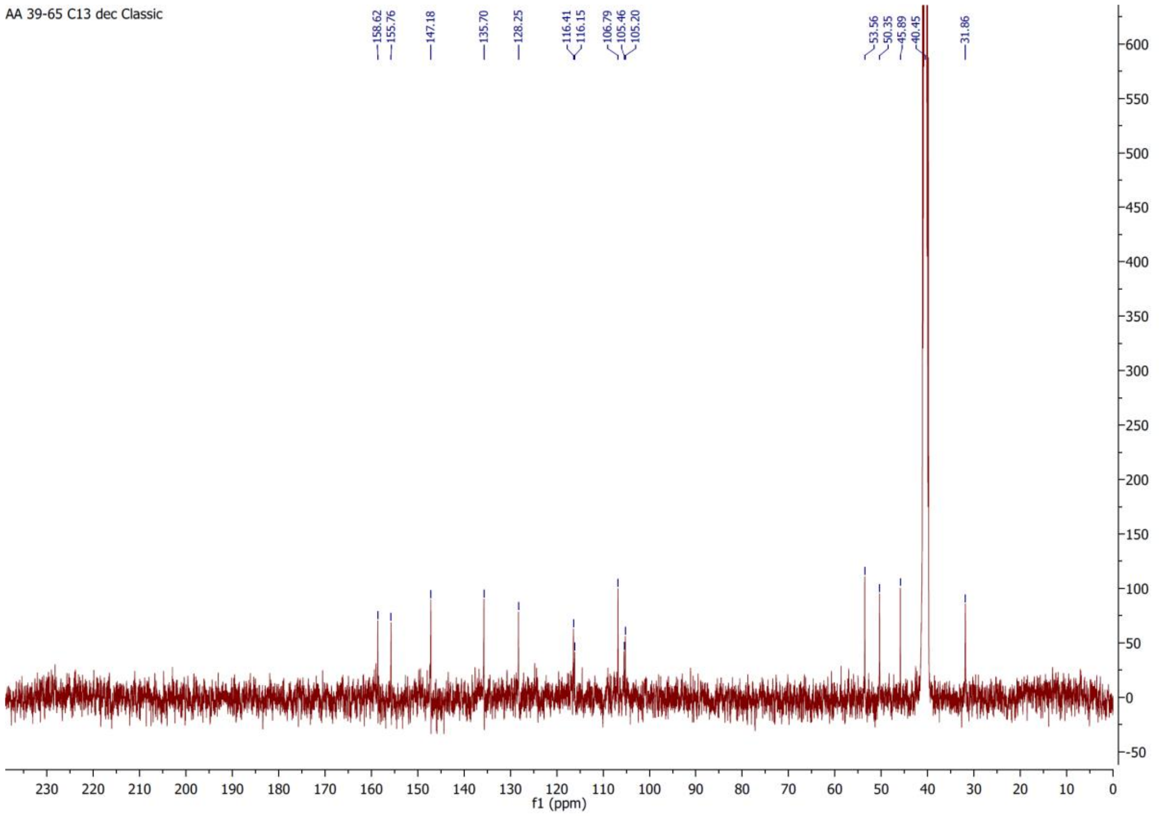

^{13}C NMR spectrum of compound **22b** (100 MHz, DMSO-d_6)

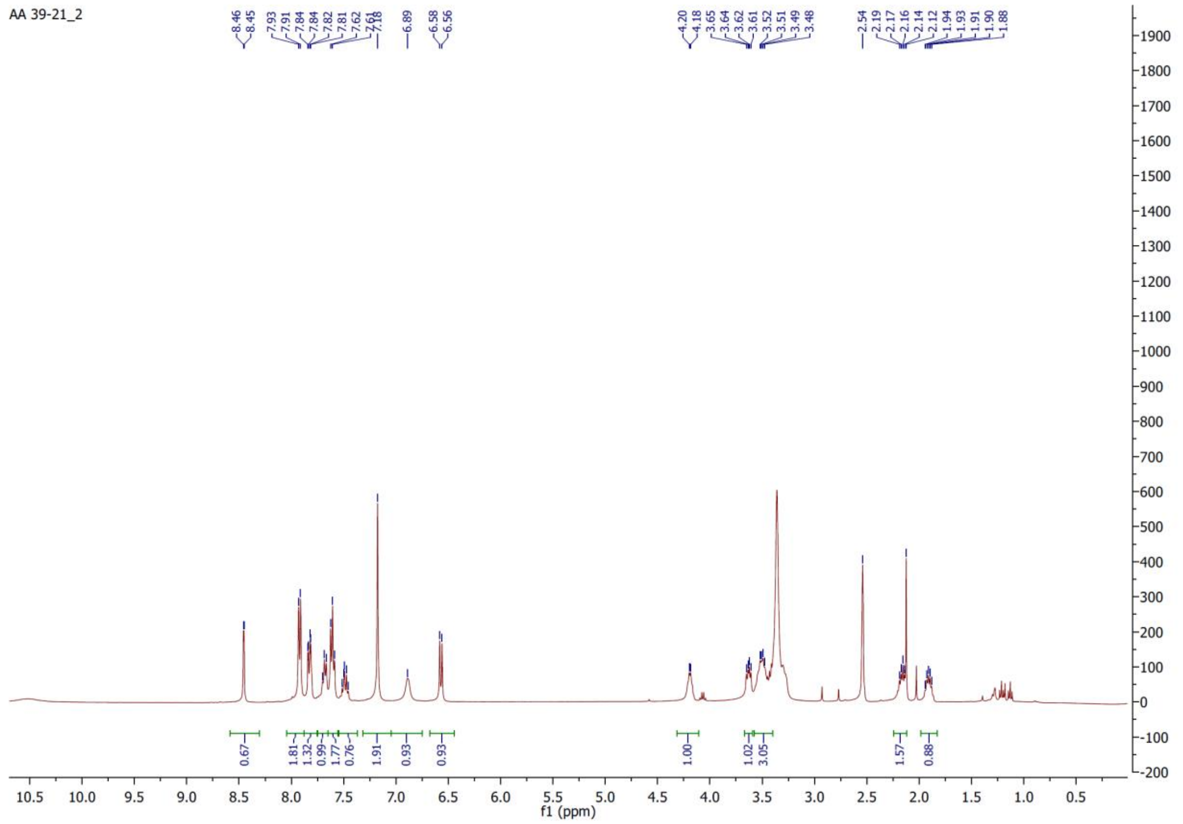

^{1}H NMR spectrum of compound **24a** (400 MHz, DMSO-d_6)

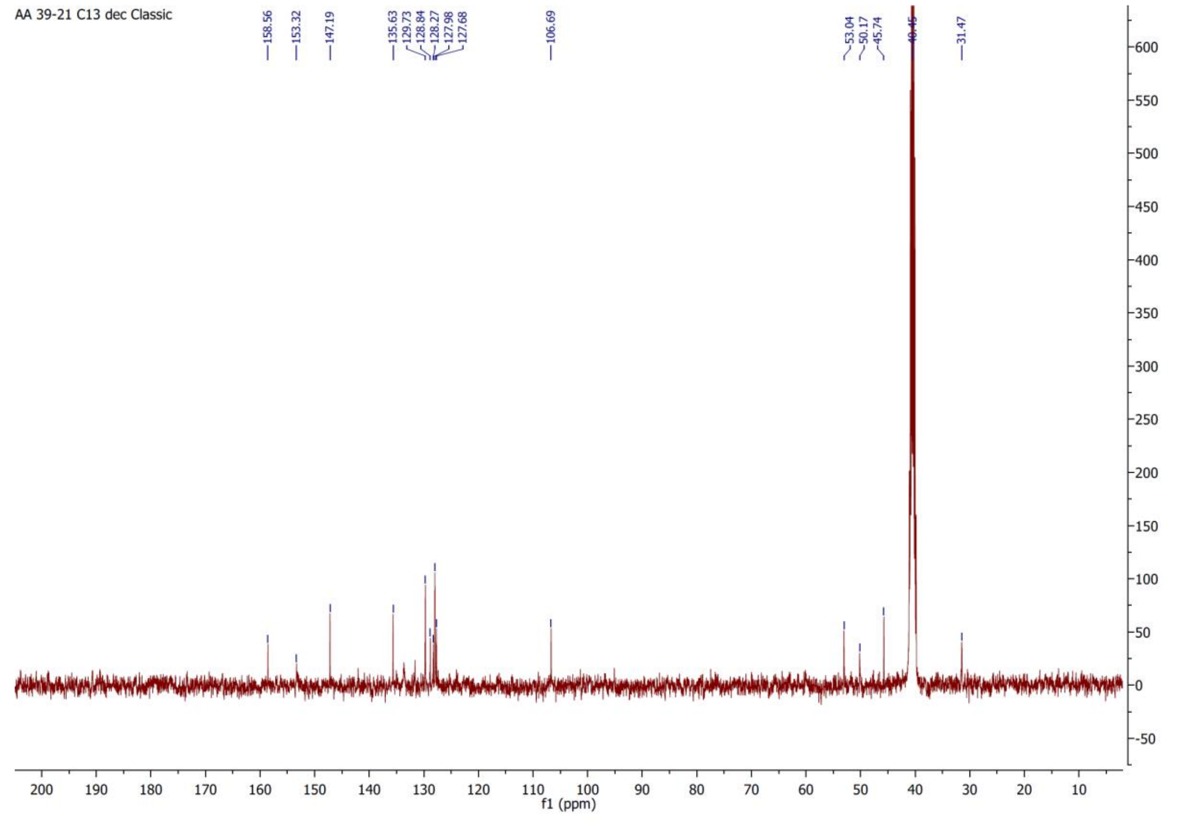

^{13}C NMR spectrum of compound **24a** (100 MHz, DMSO-d_6)

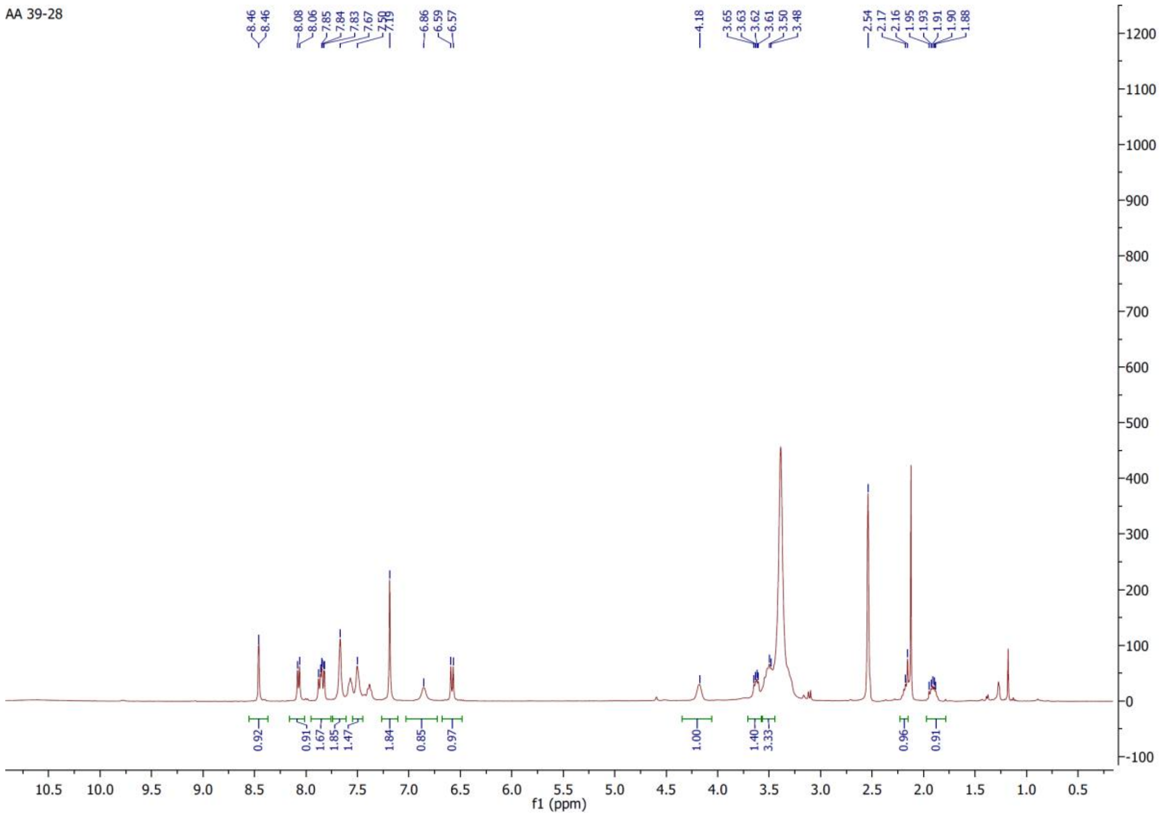

^{1}H NMR spectrum of compound **25a** (400 MHz, DMSO-d_6)

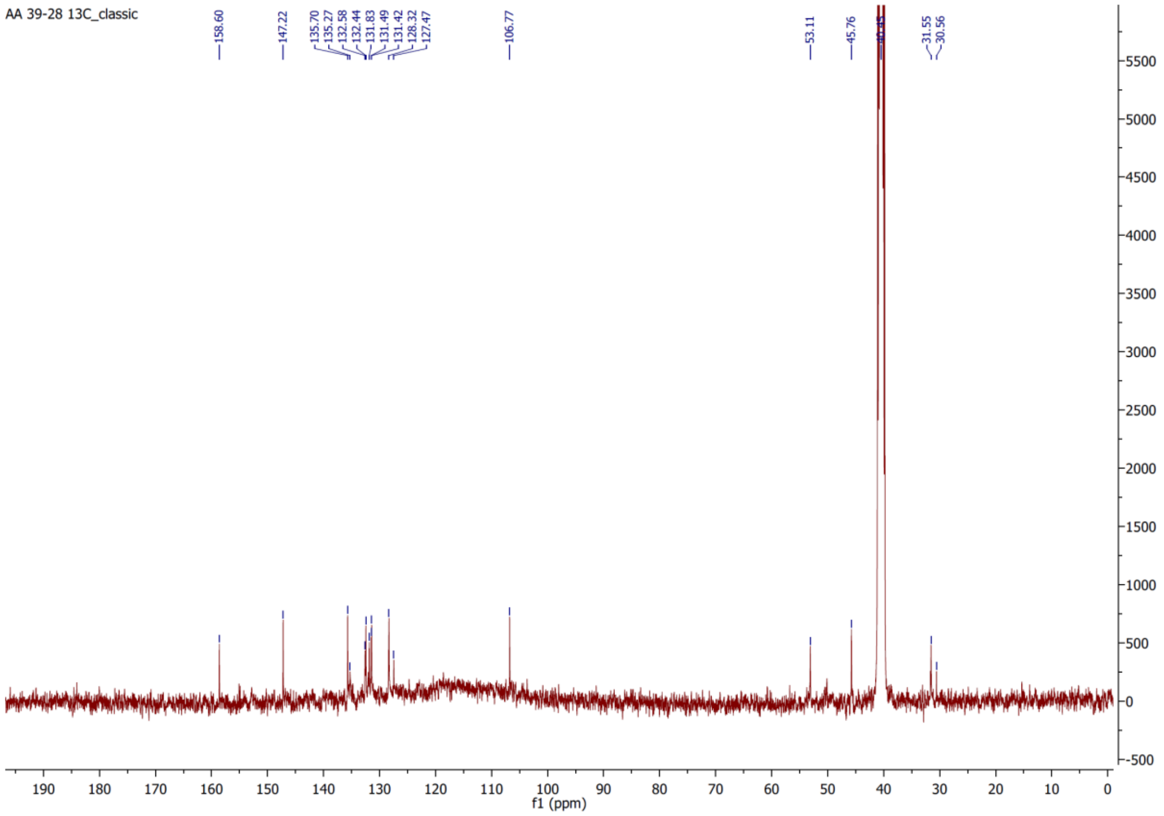

^{13}C NMR spectrum of compound **25a** (100 MHz, DMSO-d_6)

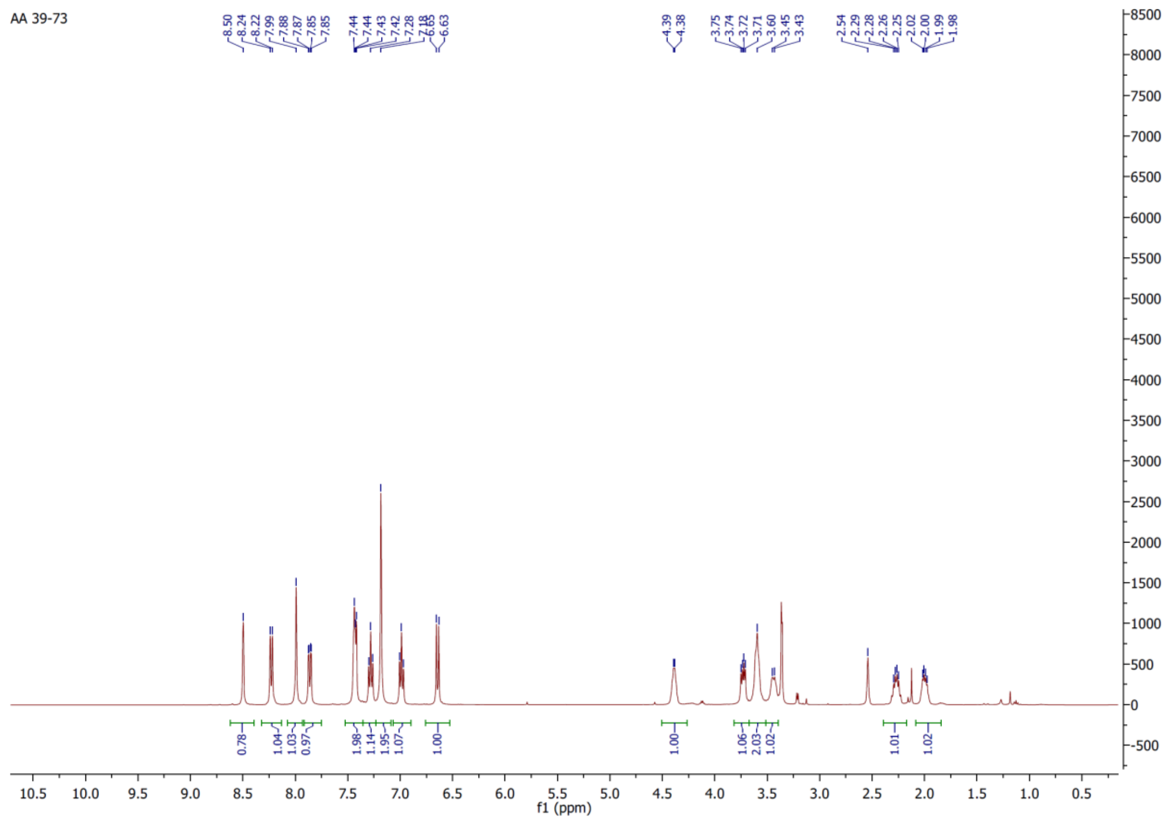

^{1}H NMR spectrum of compound **25b** (400 MHz, DMSO-d_6)

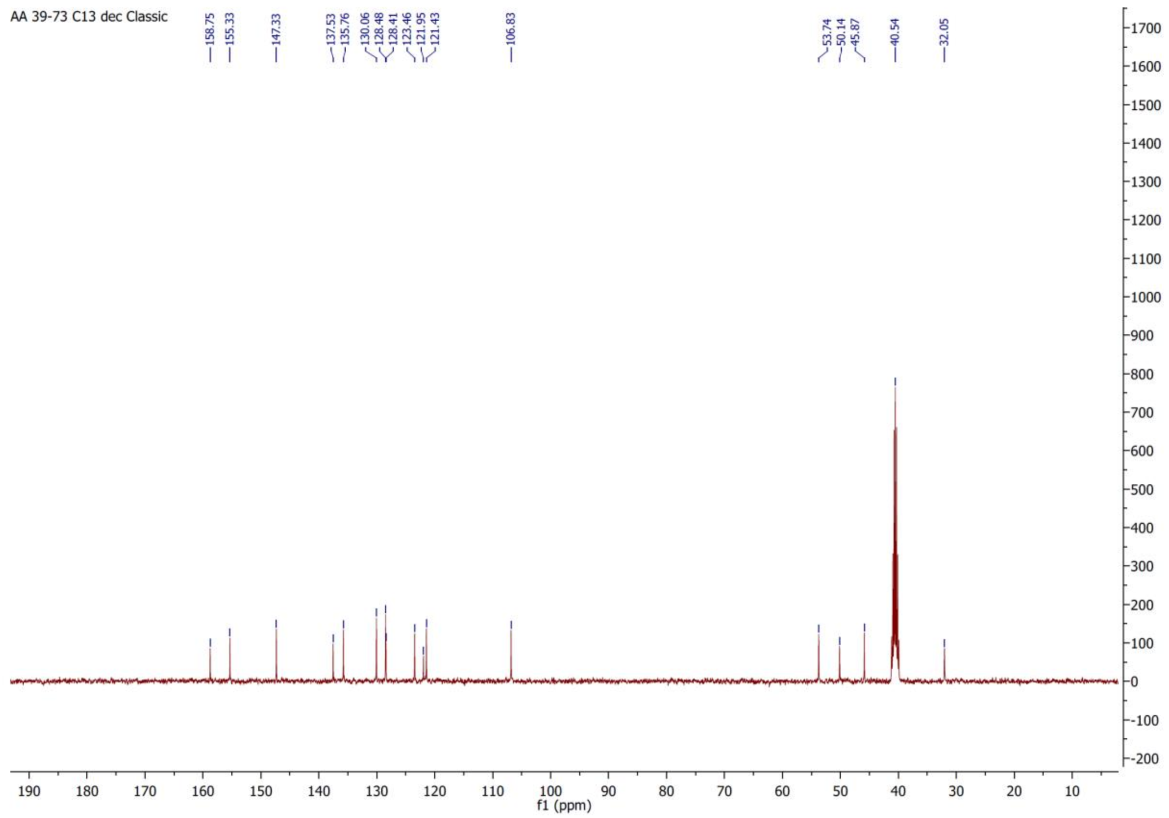

^{13}C NMR spectrum of compound **25b** (100 MHz, DMSO-d_6)

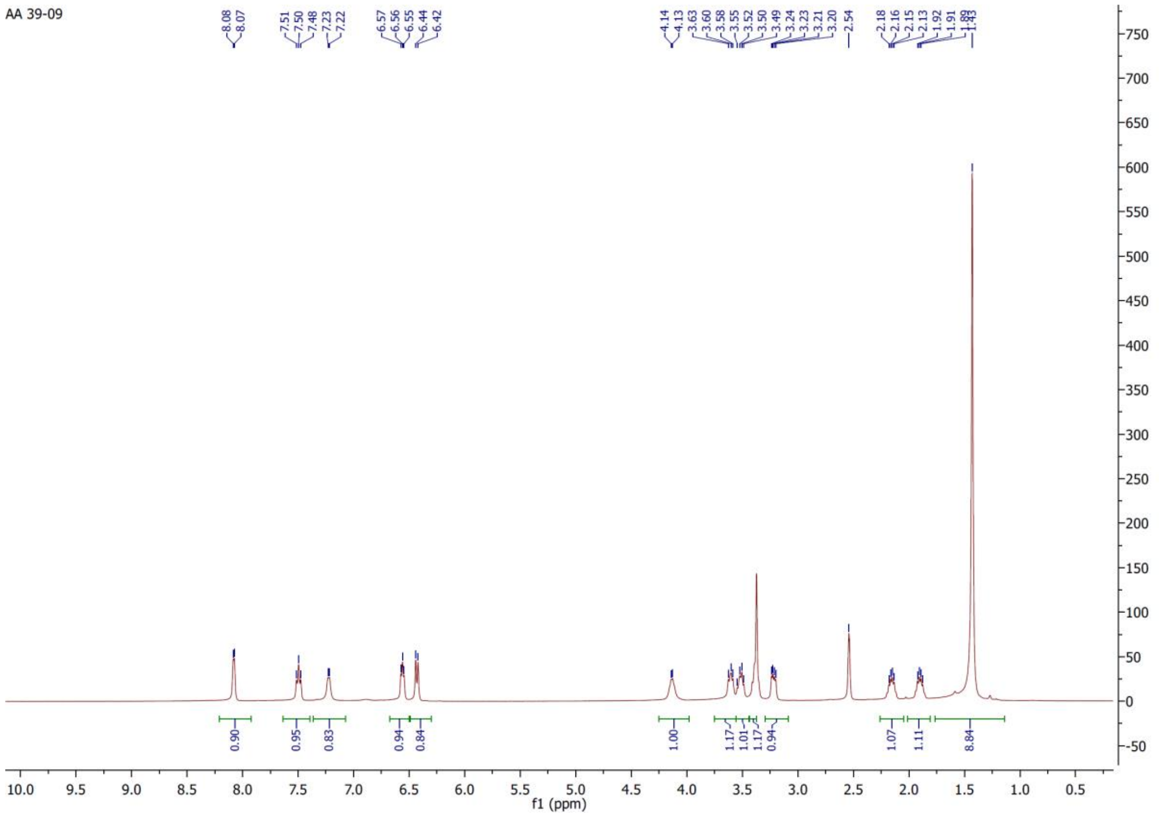

^{1}H NMR spectrum of compound **27a** (400 MHz, DMSO-d_6)

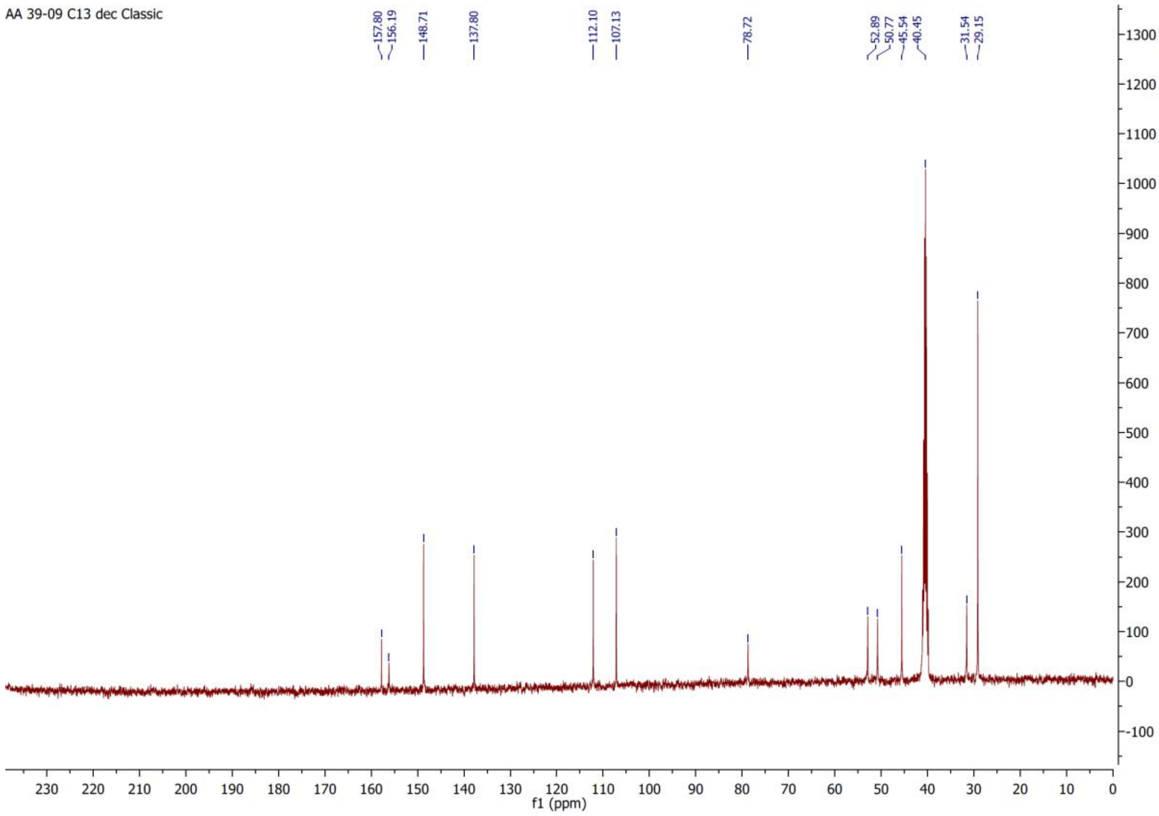

^{13}C NMR spectrum of compound **27a** (100 MHz, DMSO-d_6)

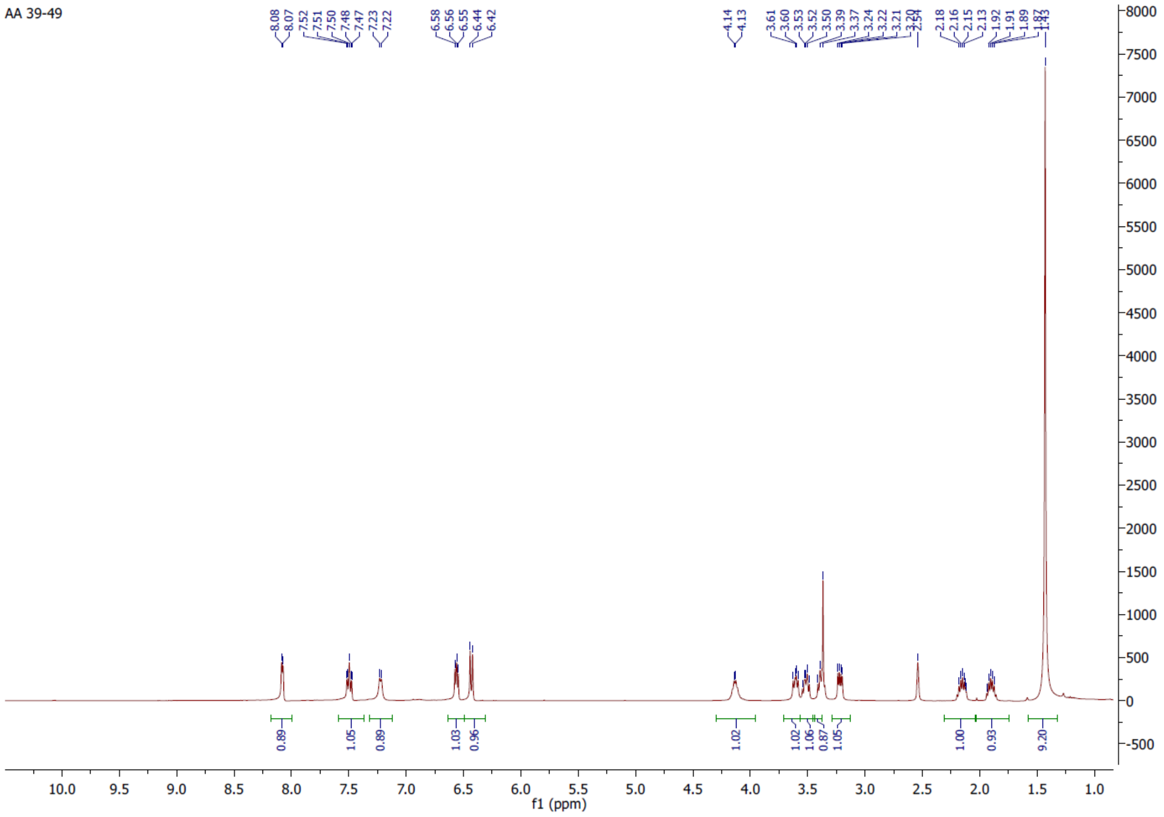

^{1}H NMR spectrum of compound **27b** (400 MHz, DMSO-d_6)

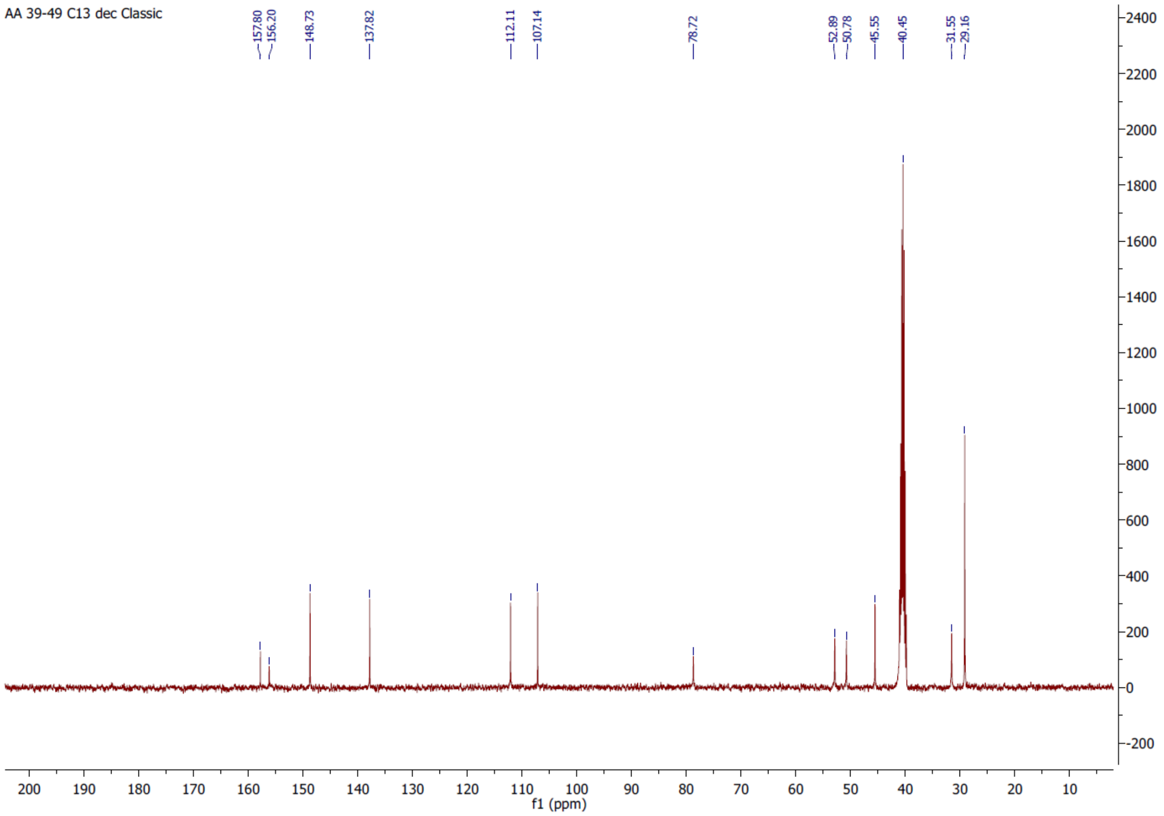

^{13}C NMR spectrum of compound **27b** (100 MHz, DMSO-d_6)

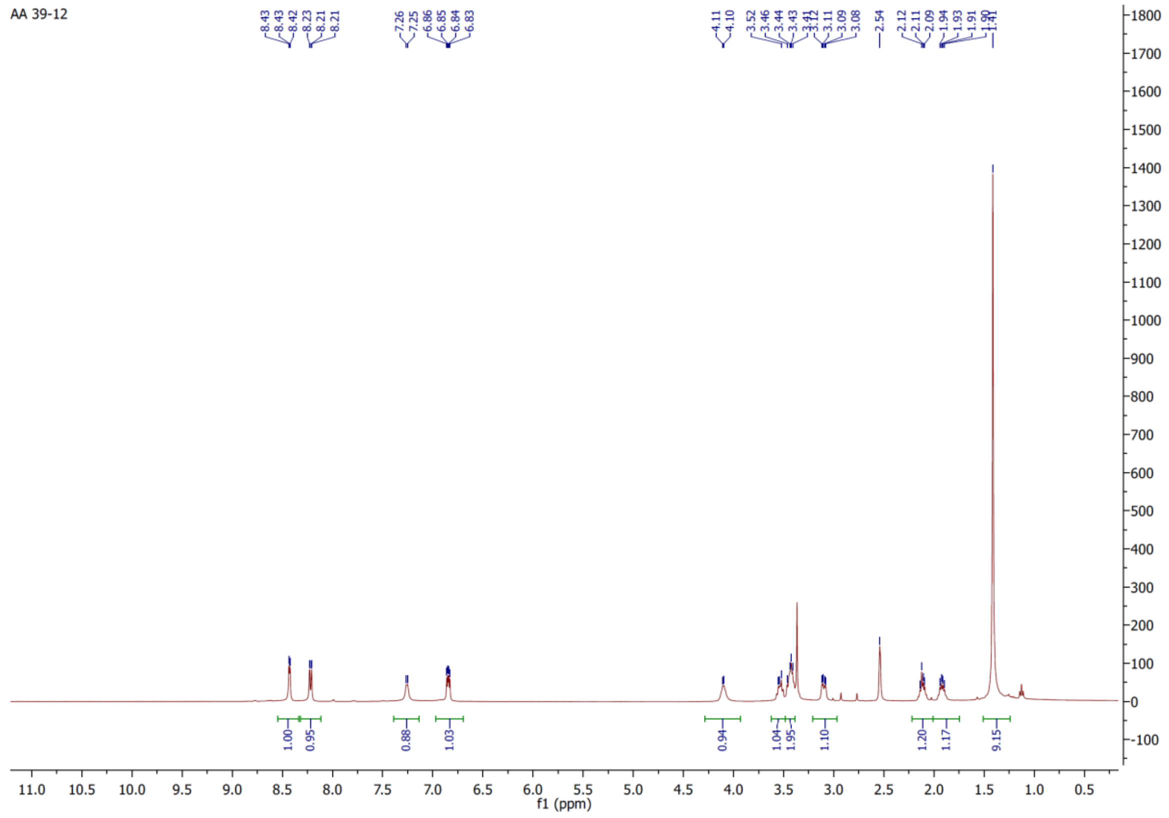

^{1}H NMR spectrum of compound **28a** (400 MHz, DMSO-d_6)

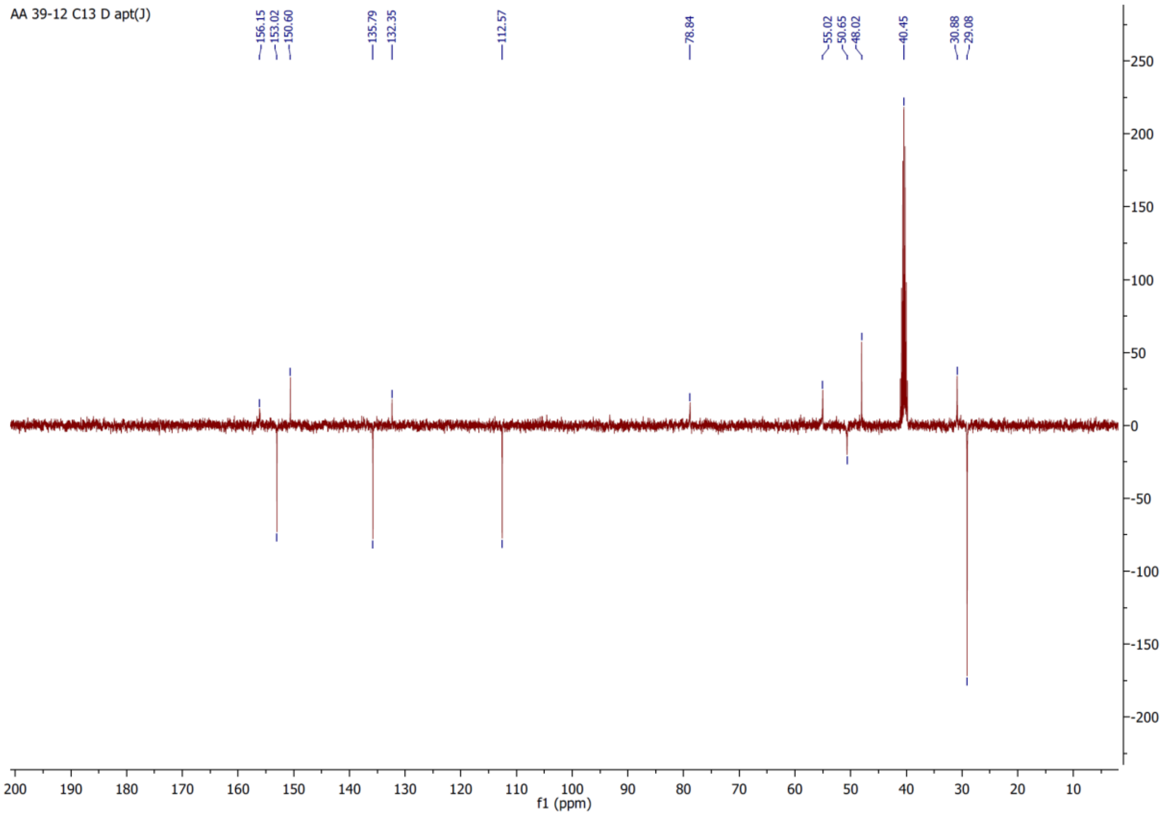

^{13}C NMR spectrum of compound **28a** (100 MHz, DMSO-d_6)

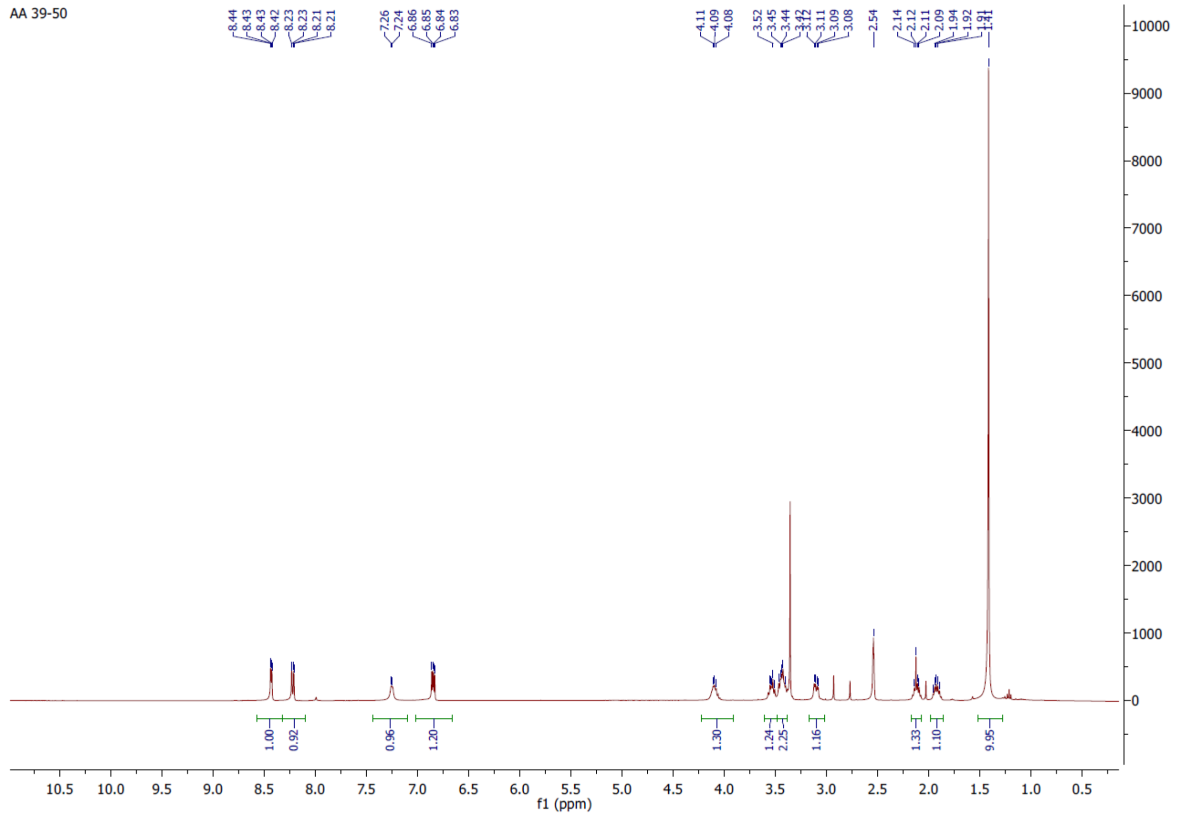

^{1}H NMR spectrum of compound **28b** (400 MHz, DMSO-d_6)

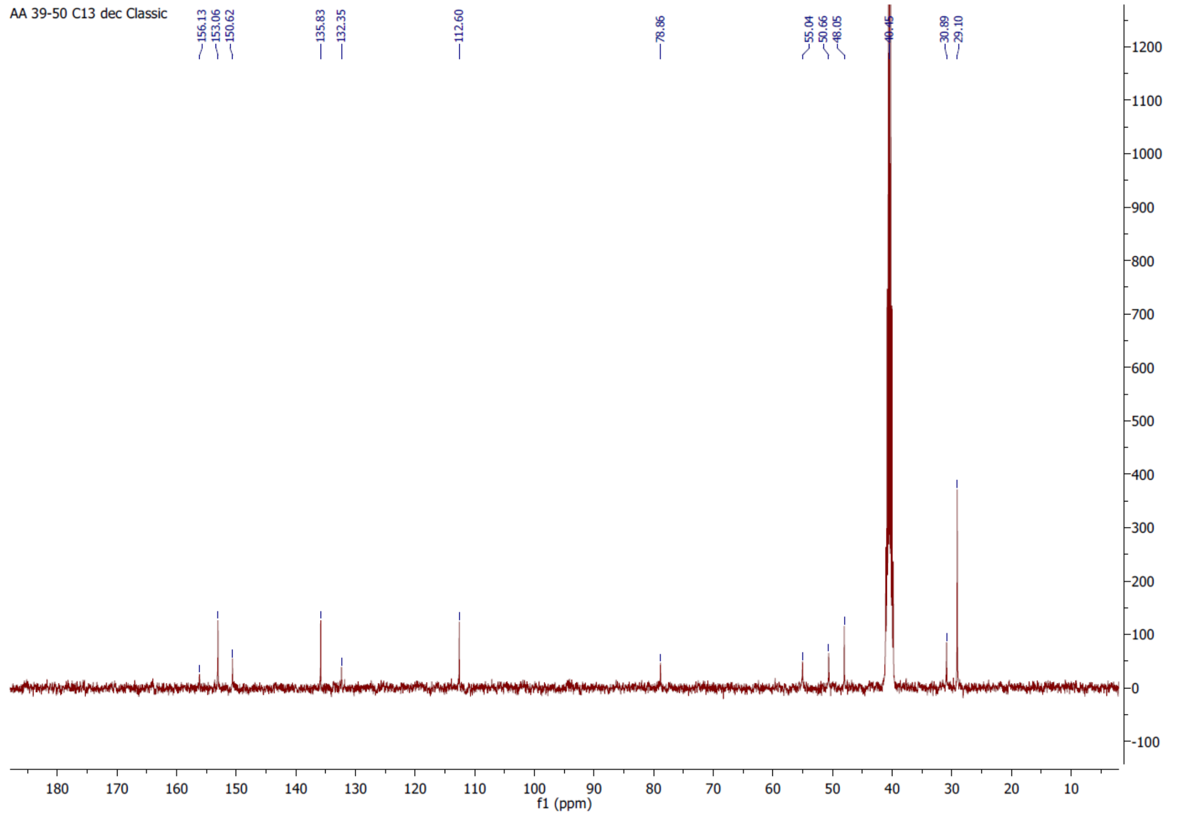

^{13}C NMR spectrum of compound **28b** (100 MHz, DMSO-d_6)

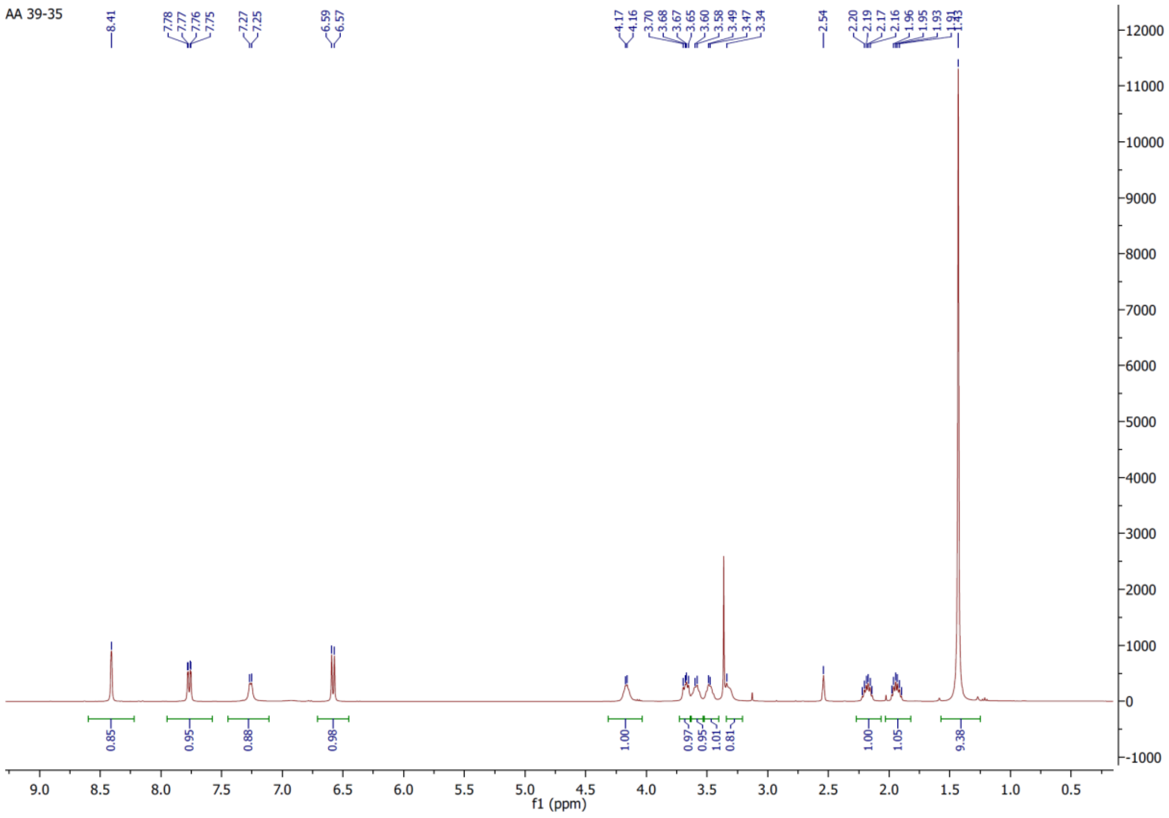

^{1}H NMR spectrum of compound **29a** (400 MHz, DMSO-d_6)

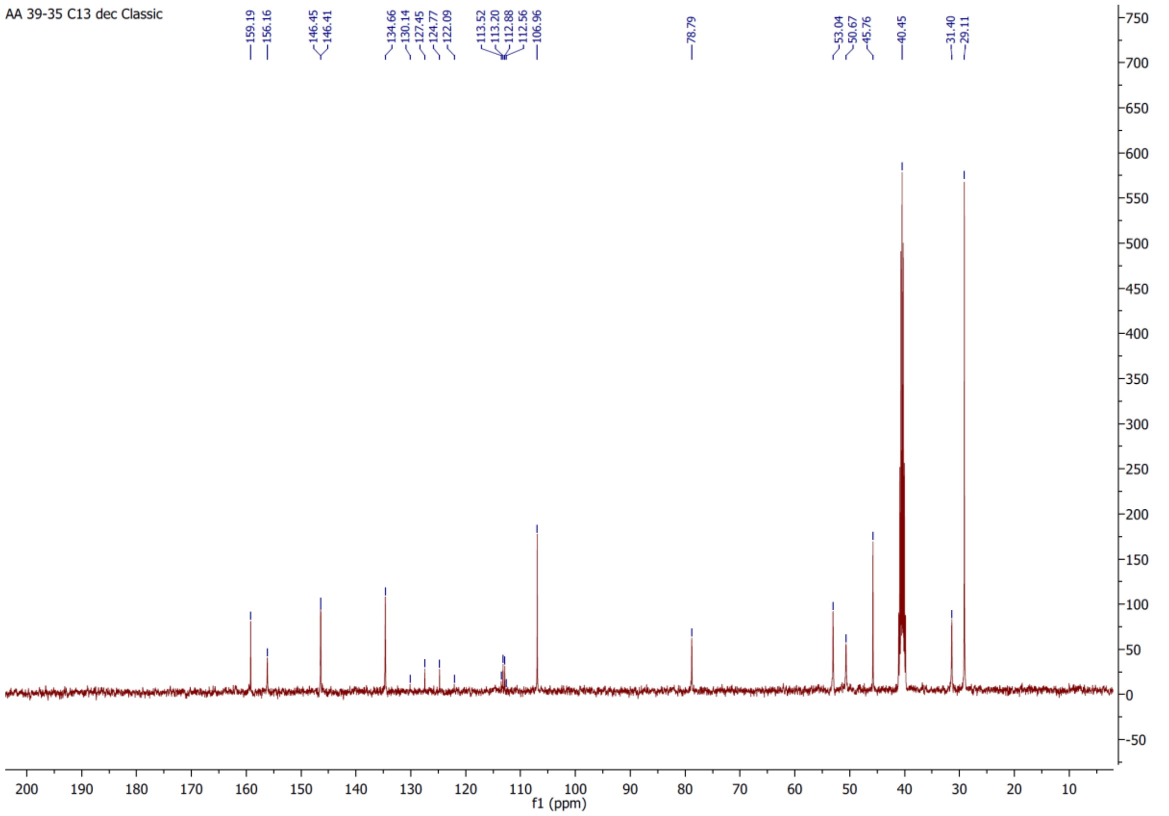

^{13}C NMR spectrum of compound **29a** (100 MHz, DMSO-d_6)

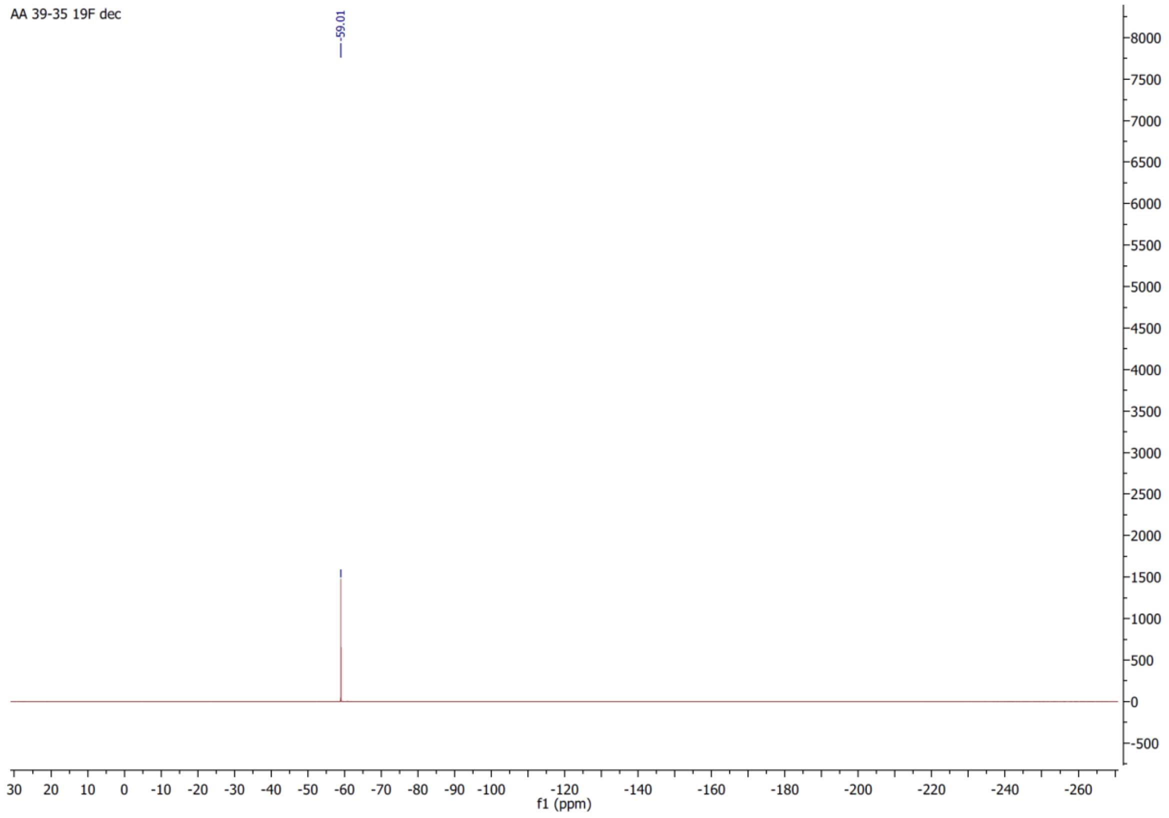

^{19}F NMR spectrum of compound **29a** (376 MHz, DMSO-d_6)

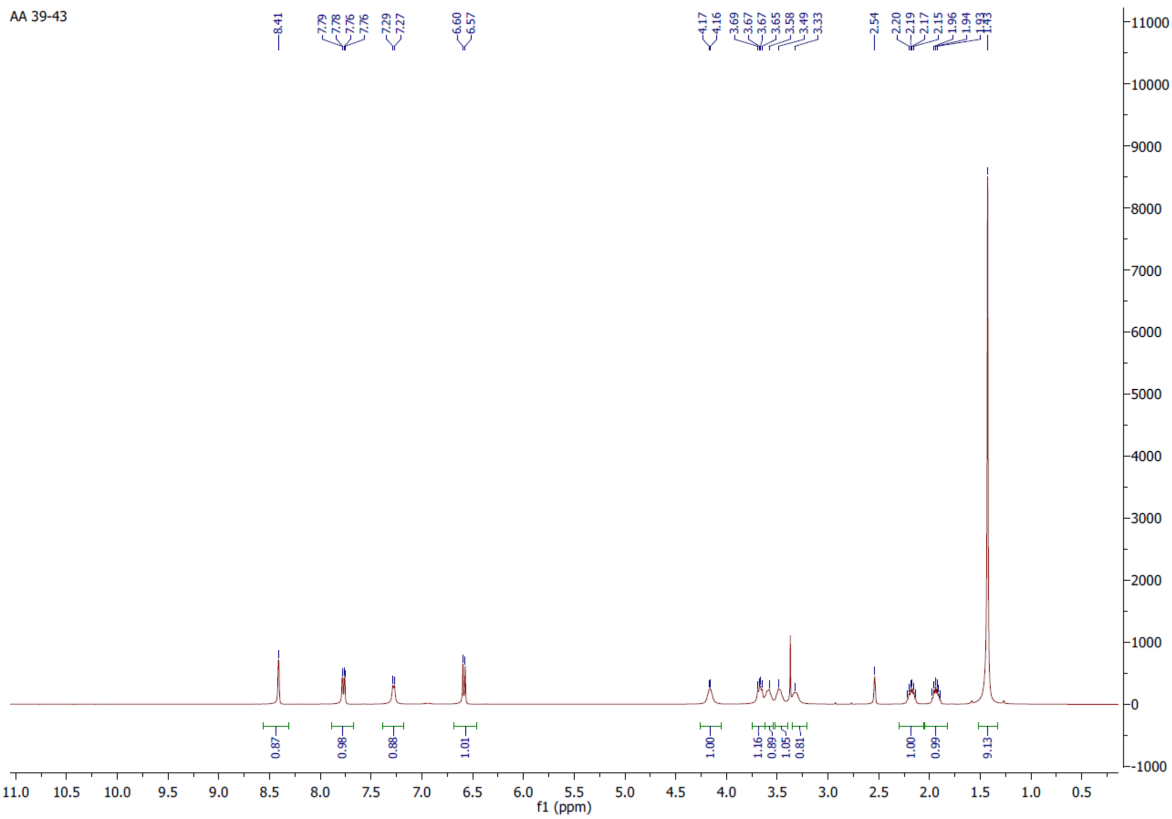

^{1}H NMR spectrum of compound **29b** (400 MHz, DMSO-d_6)

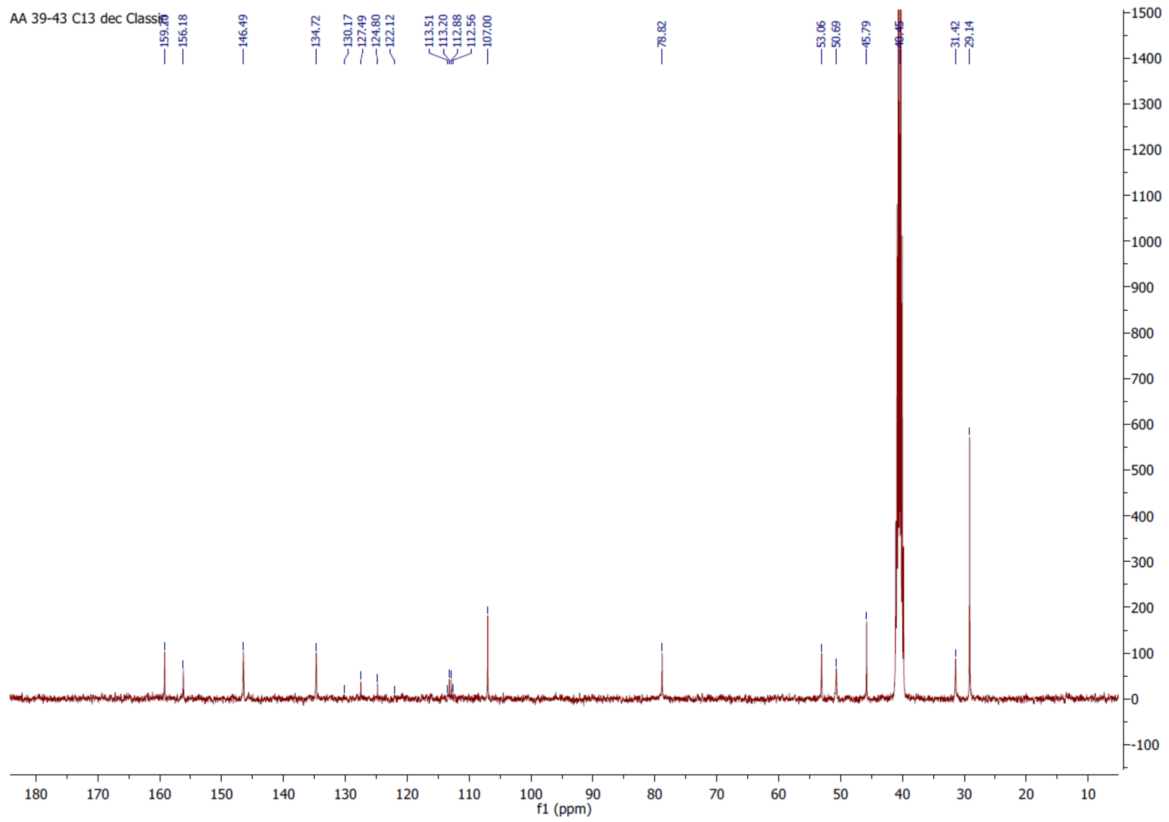

^{13}C NMR spectrum of compound **29b** (100 MHz, DMSO-d_6)

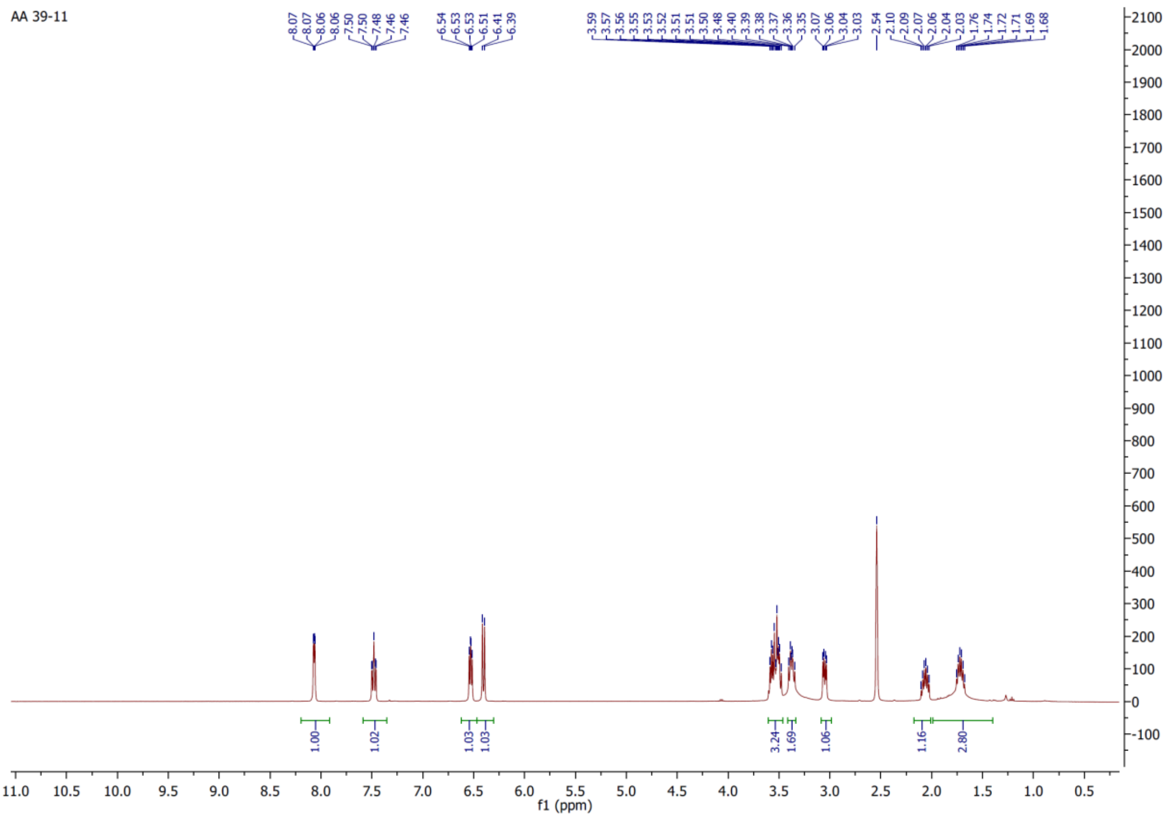

^{1}H NMR spectrum of compound **30a** (400 MHz, DMSO-d_6)

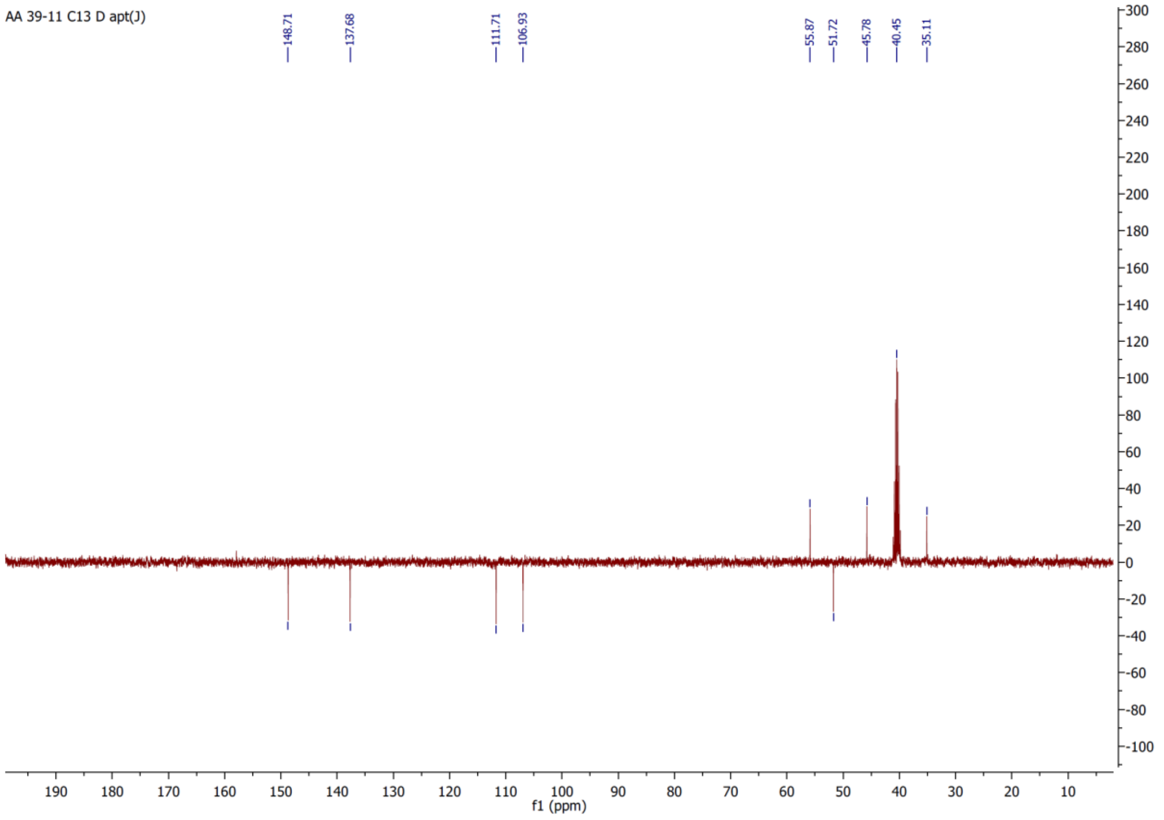

^{13}C NMR spectrum of compound **30a** (100 MHz, DMSO-d_6)

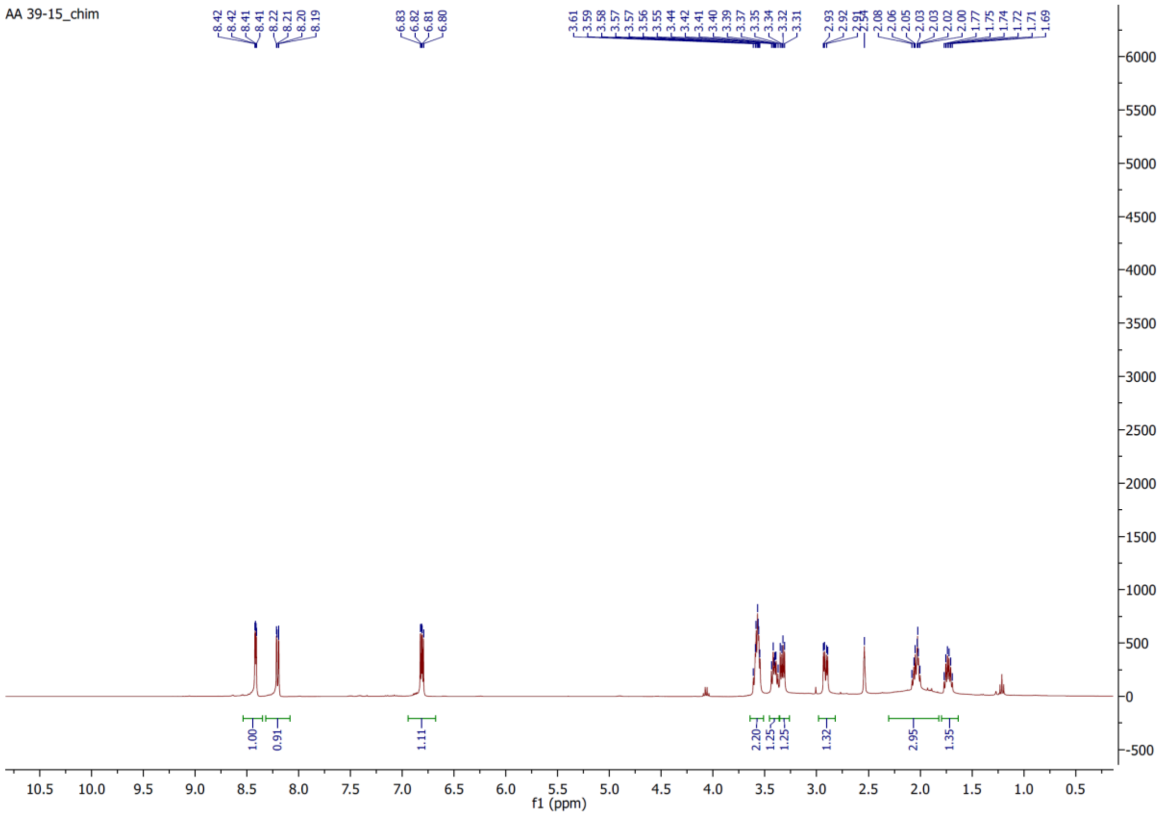

^{1}H NMR spectrum of compound **31a** (400 MHz, DMSO-d_6)

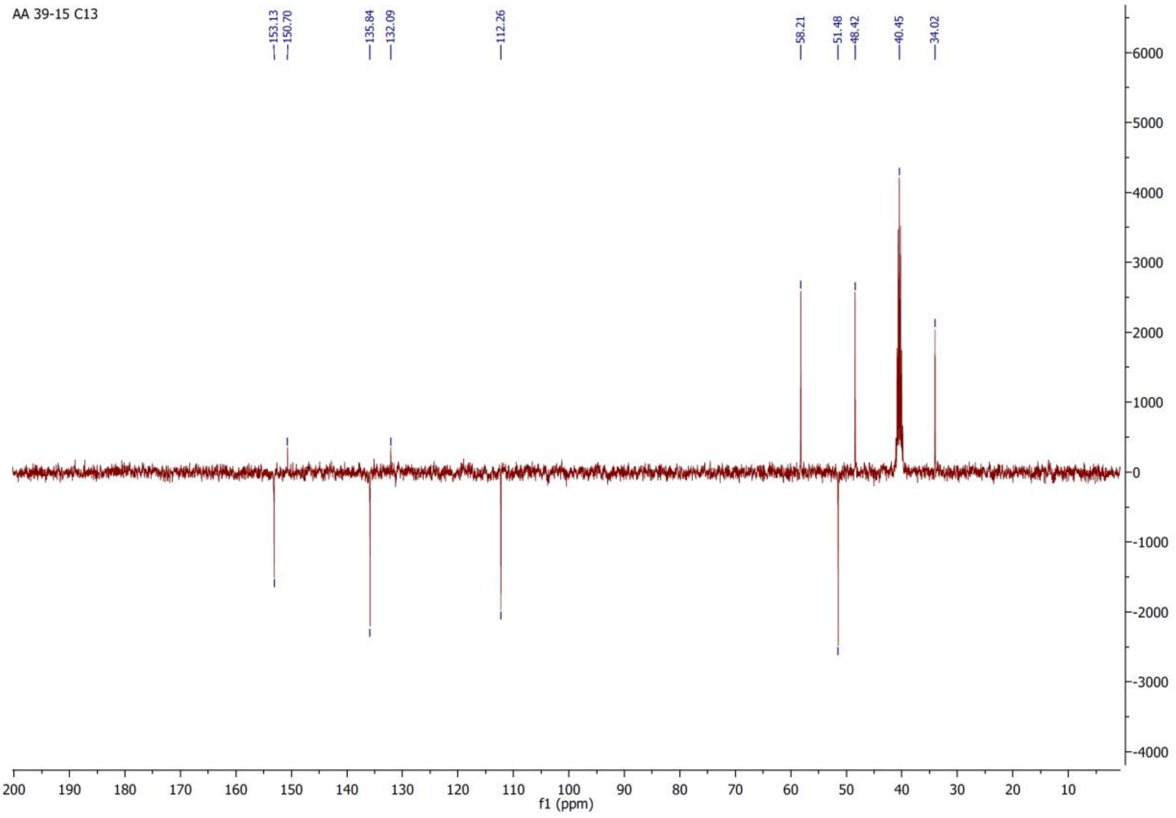

^{13}C NMR spectrum of compound **31a** (100 MHz, DMSO-d_6)

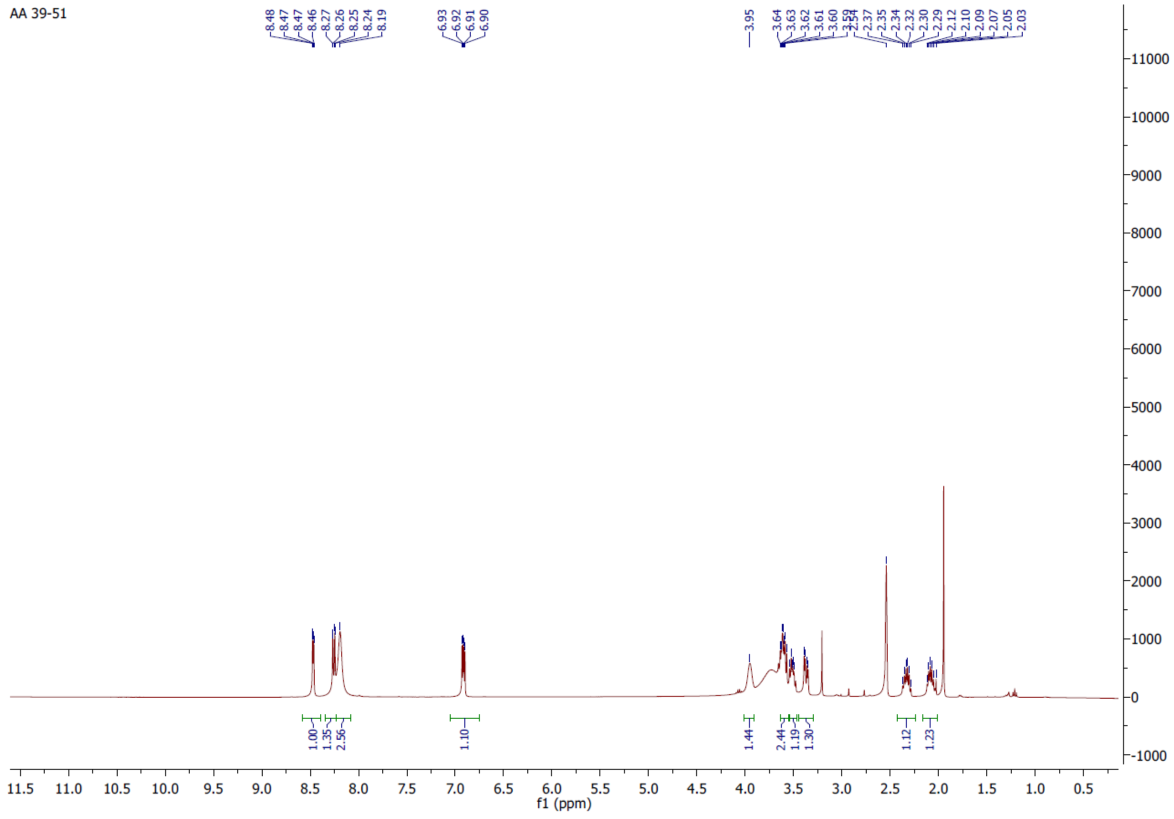

^{1}H NMR spectrum of compound **31b** (400 MHz, DMSO-d_6)

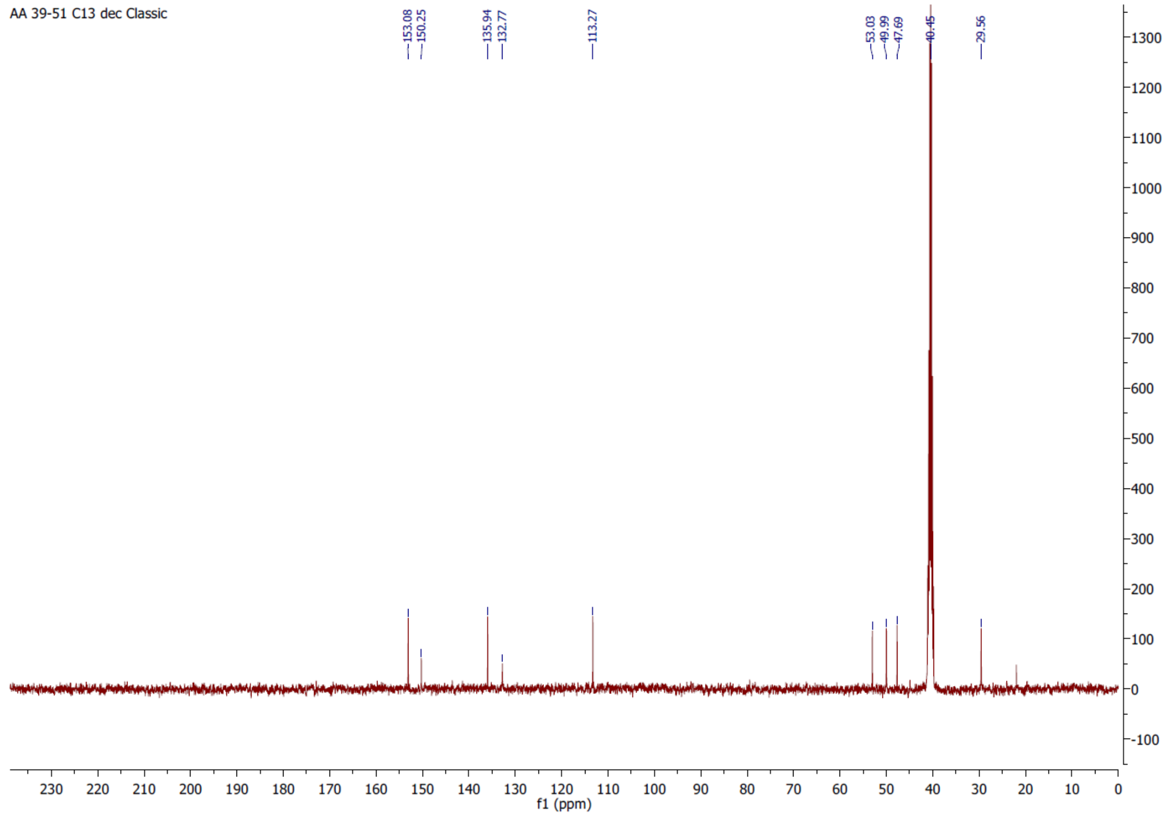

^{13}C NMR spectrum of compound **31b** (100 MHz, DMSO-d_6)

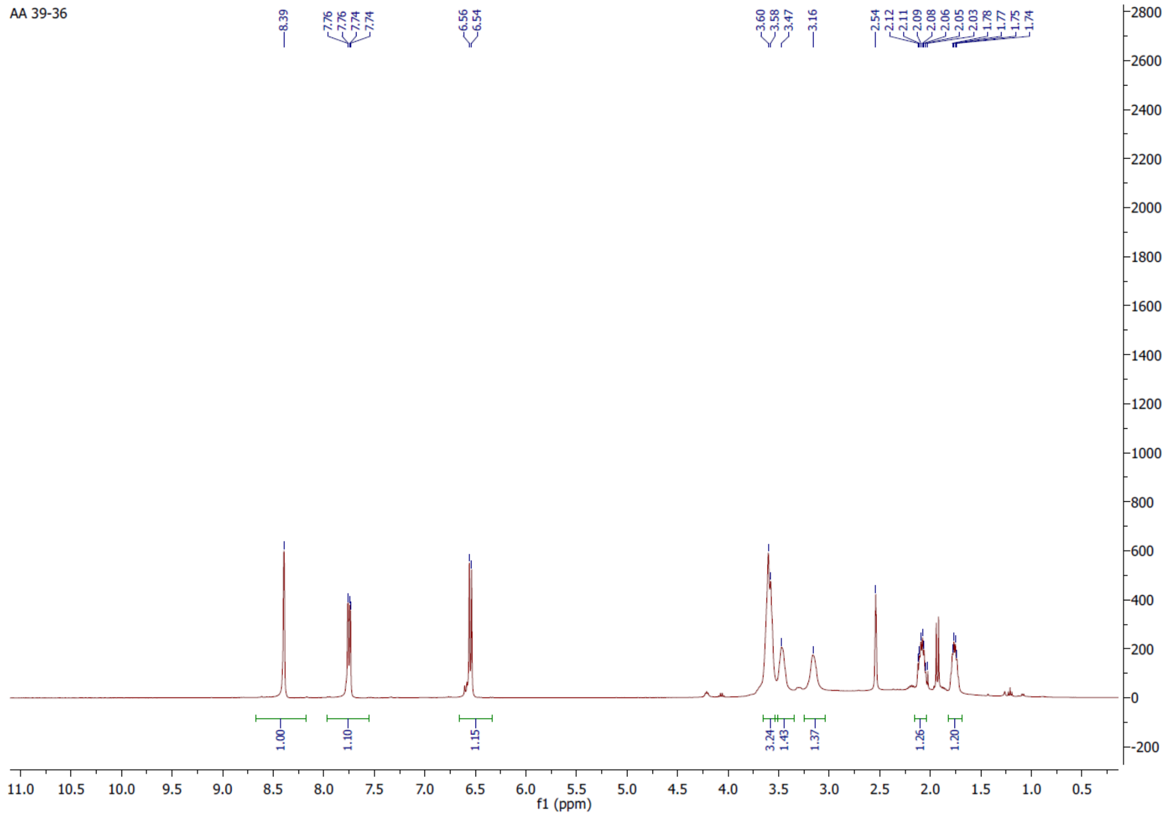

^{1}H NMR spectrum of compound **32a** (400 MHz, DMSO-d_6)

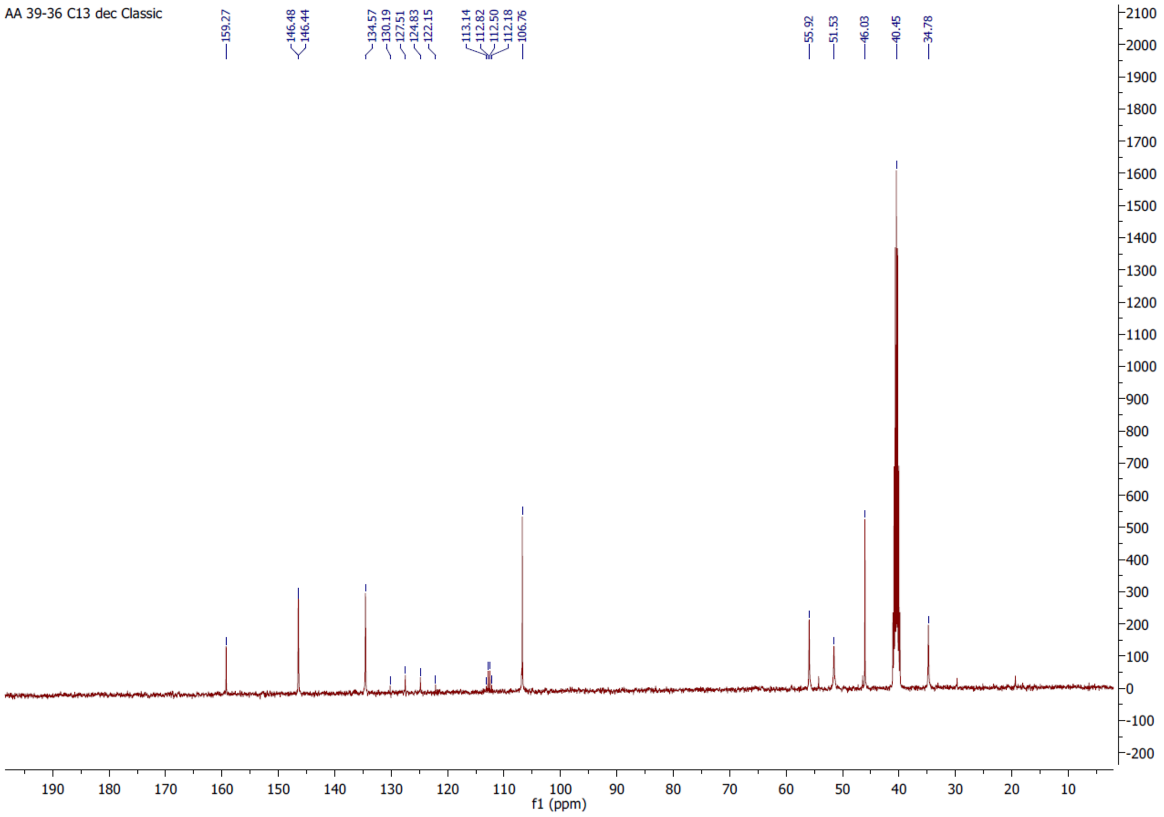

^{13}C NMR spectrum of compound **32a** (100 MHz, DMSO-d_6)

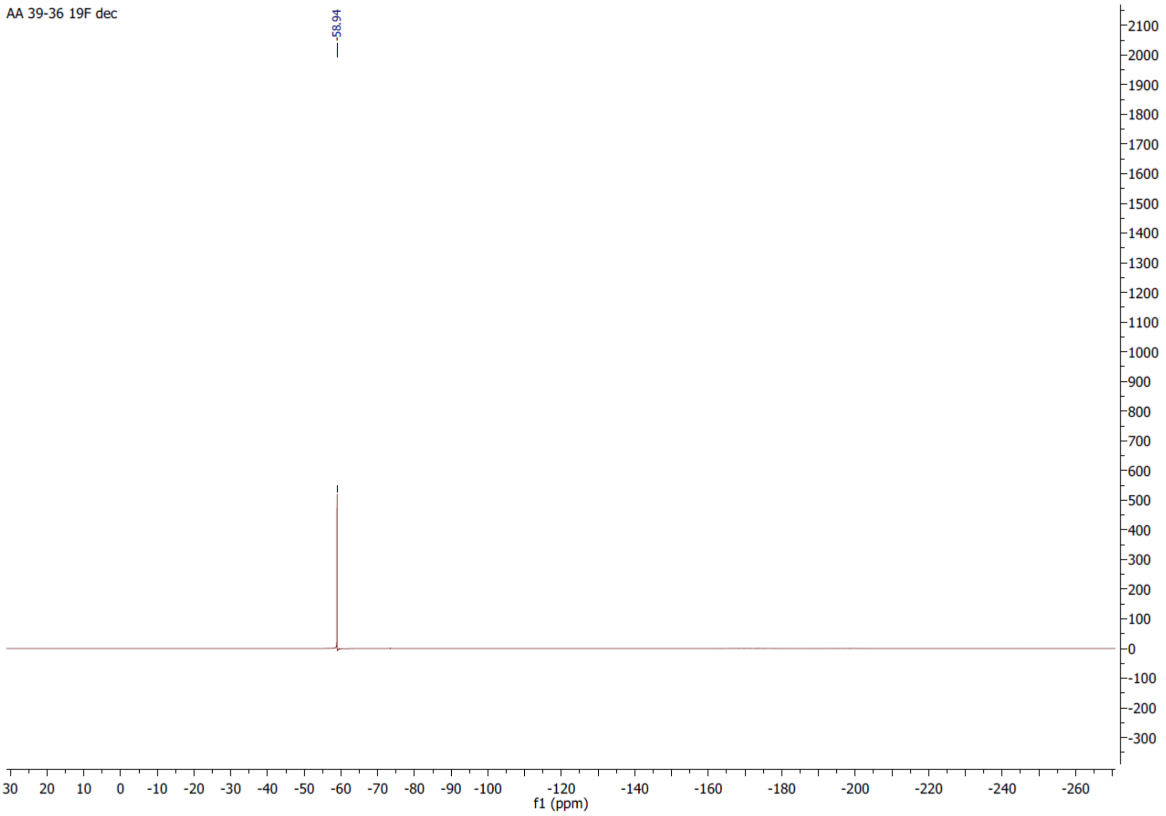

^{19}F NMR spectrum of compound **32a** (376 MHz, DMSO-d_6)

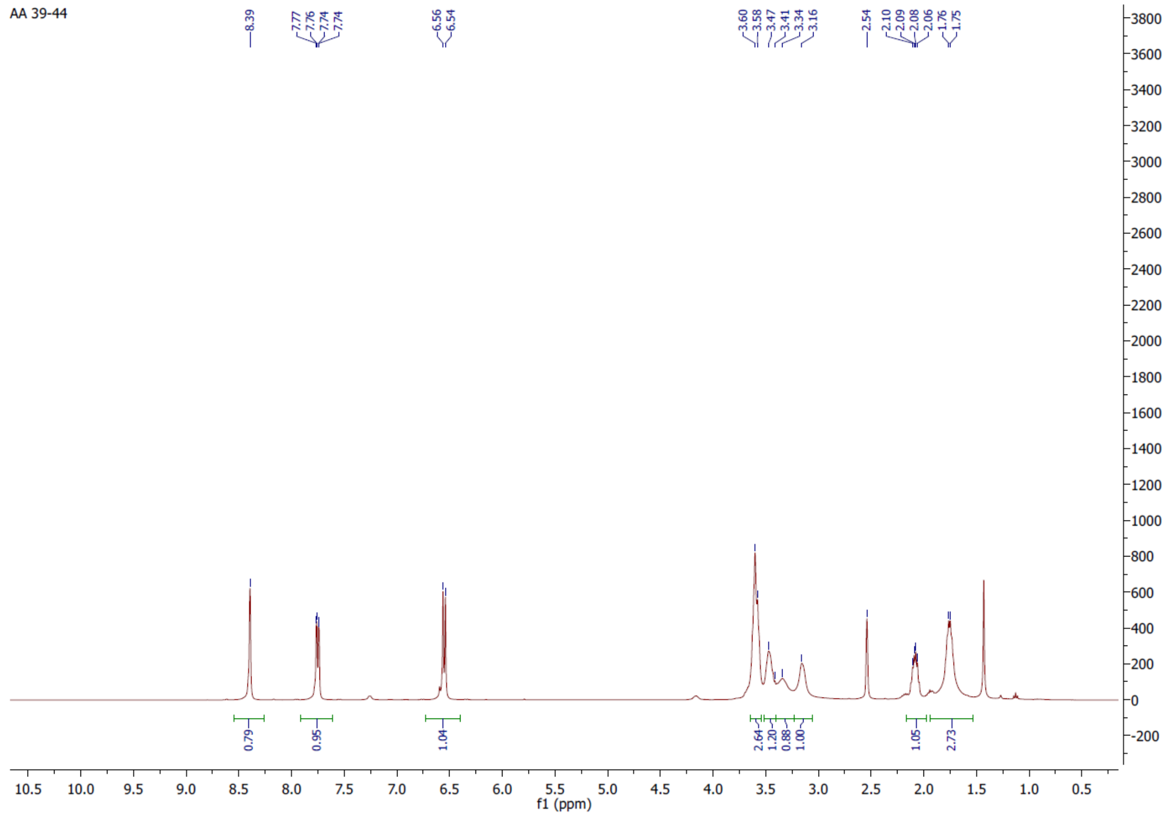

^{1}H NMR spectrum of compound **32b** (400 MHz, DMSO-d_6)

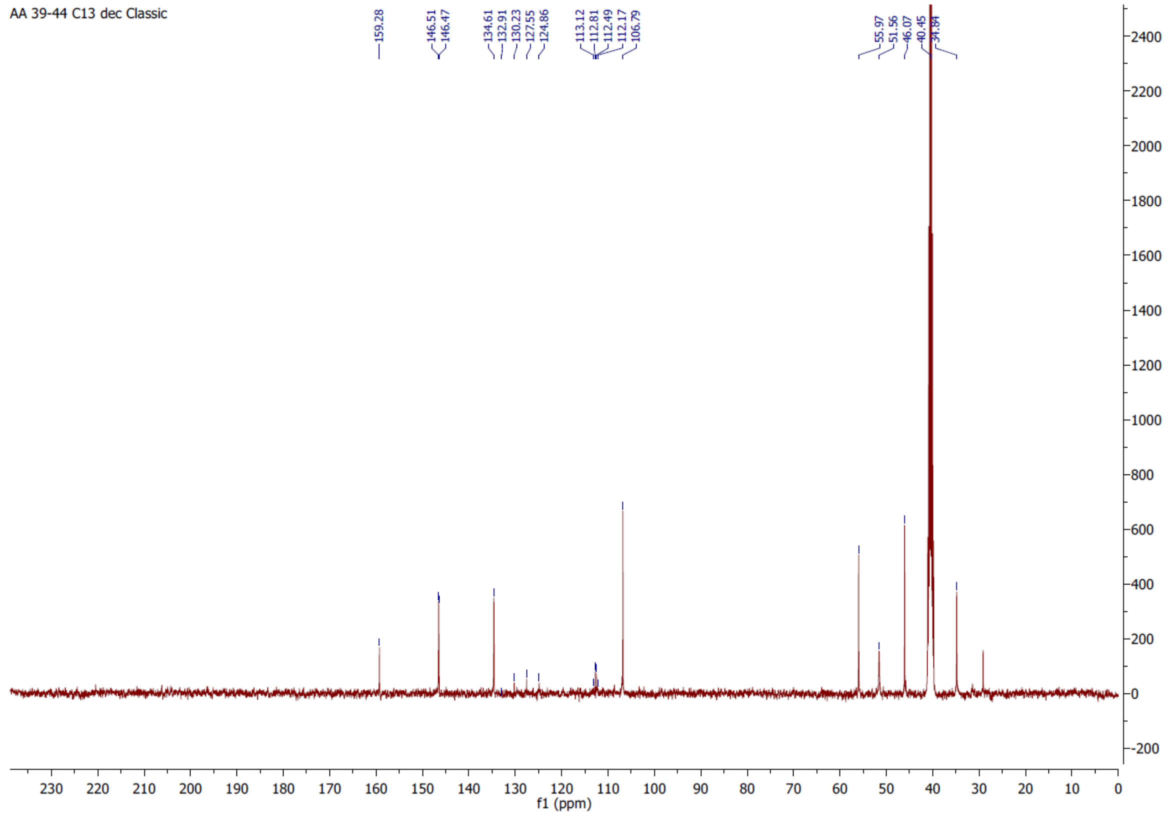

^{13}C NMR spectrum of compound **32b** (100 MHz, DMSO-d_6)

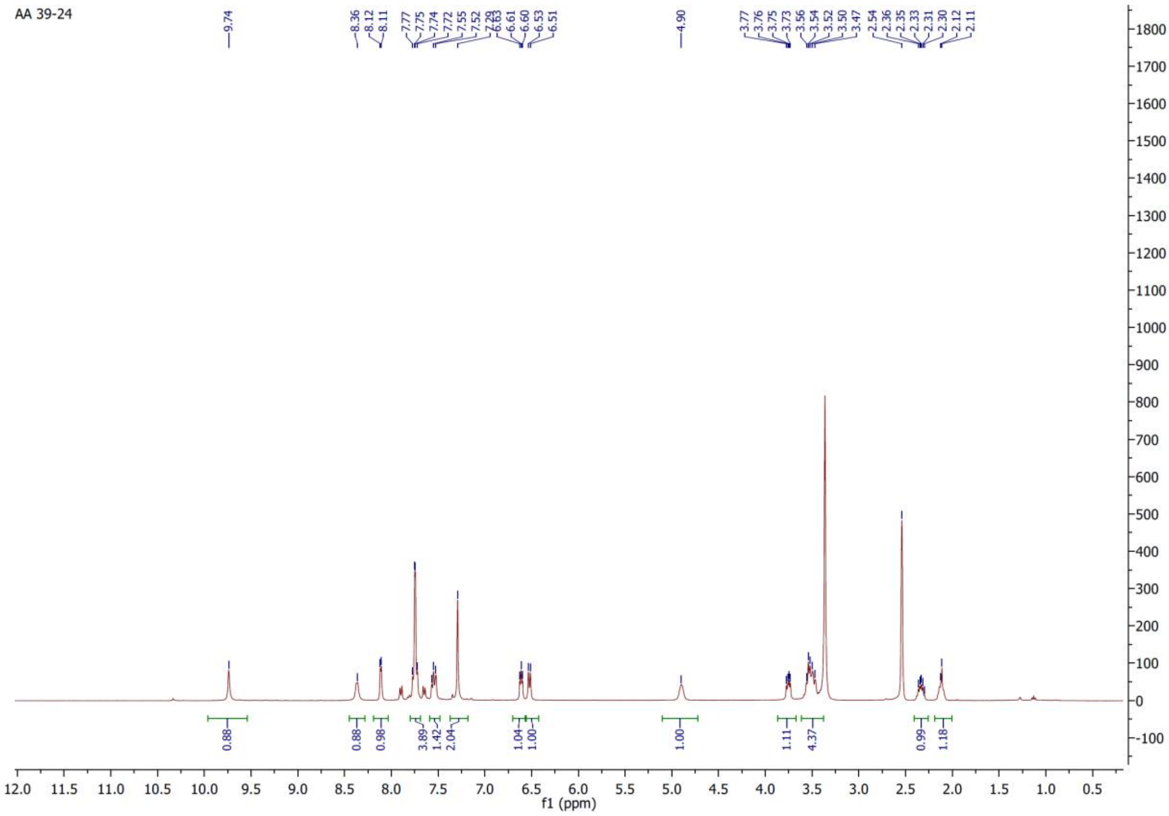

^{1}H NMR spectrum of compound **35a** (400 MHz, DMSO-d_6)

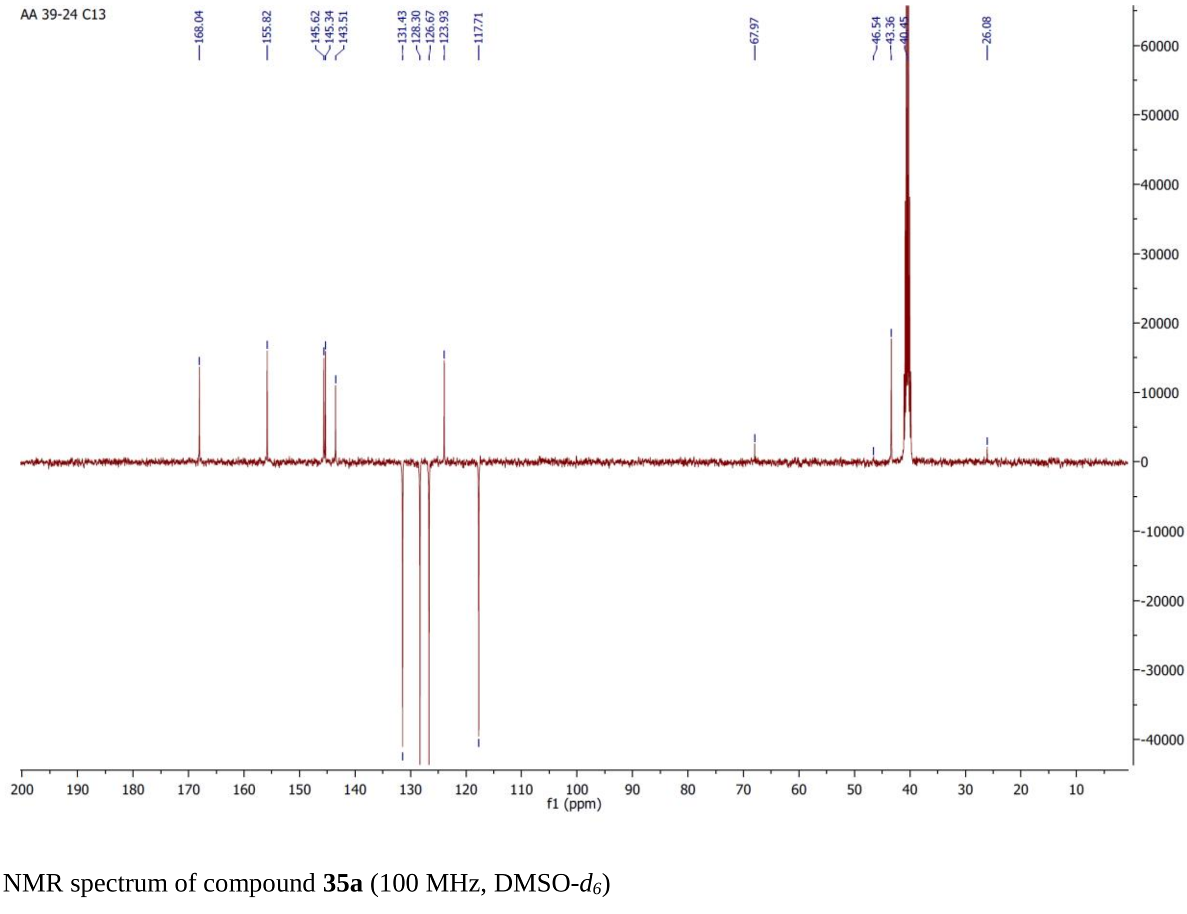

^{13}C NMR spectrum of compound **35a** (100 MHz, DMSO-d_6)

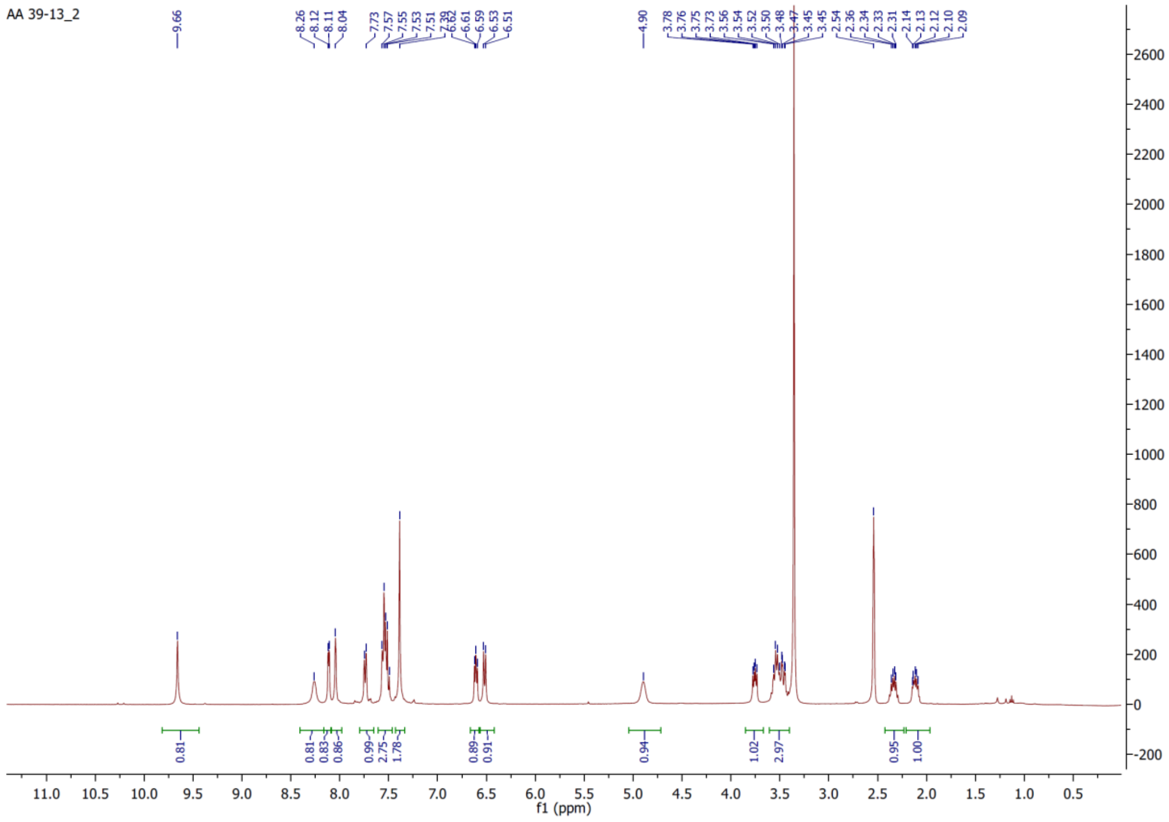

^{1}H NMR spectrum of compound **36a** (400 MHz, DMSO-d_6)

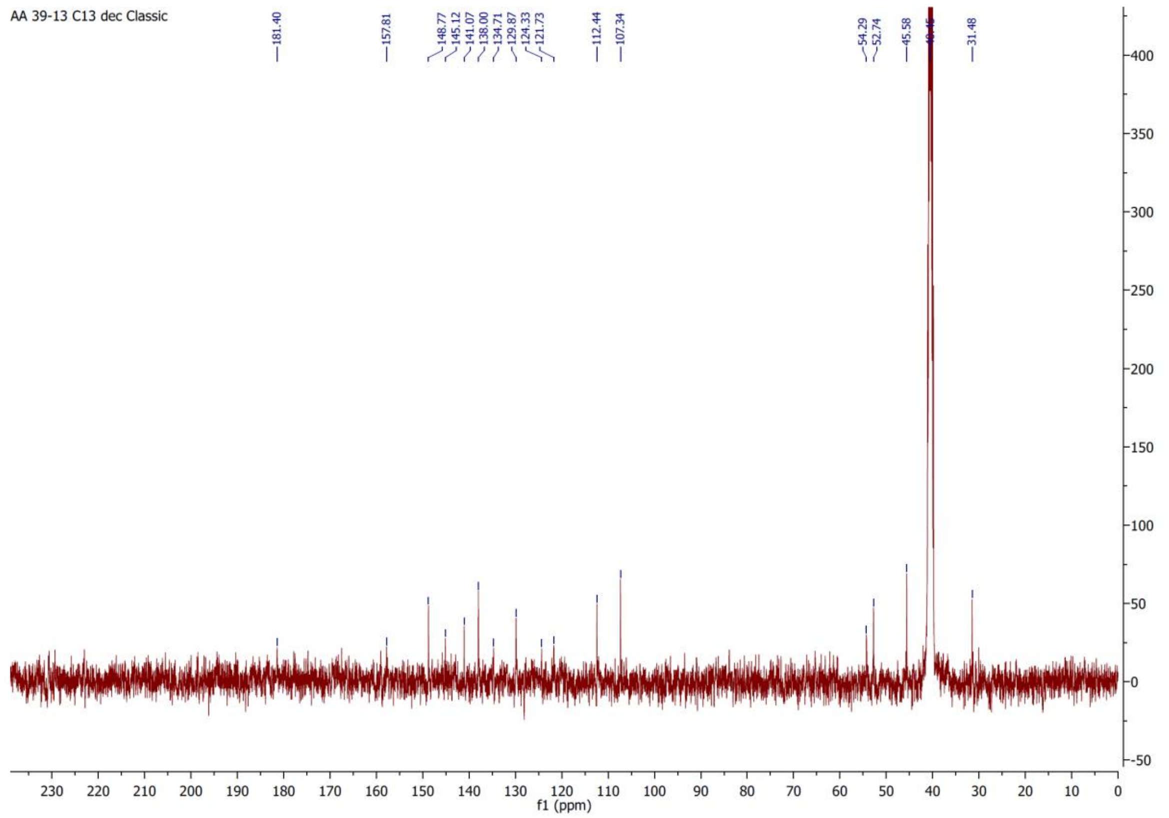

^{13}C NMR spectrum of compound **36a** (100 MHz, DMSO-d_6)

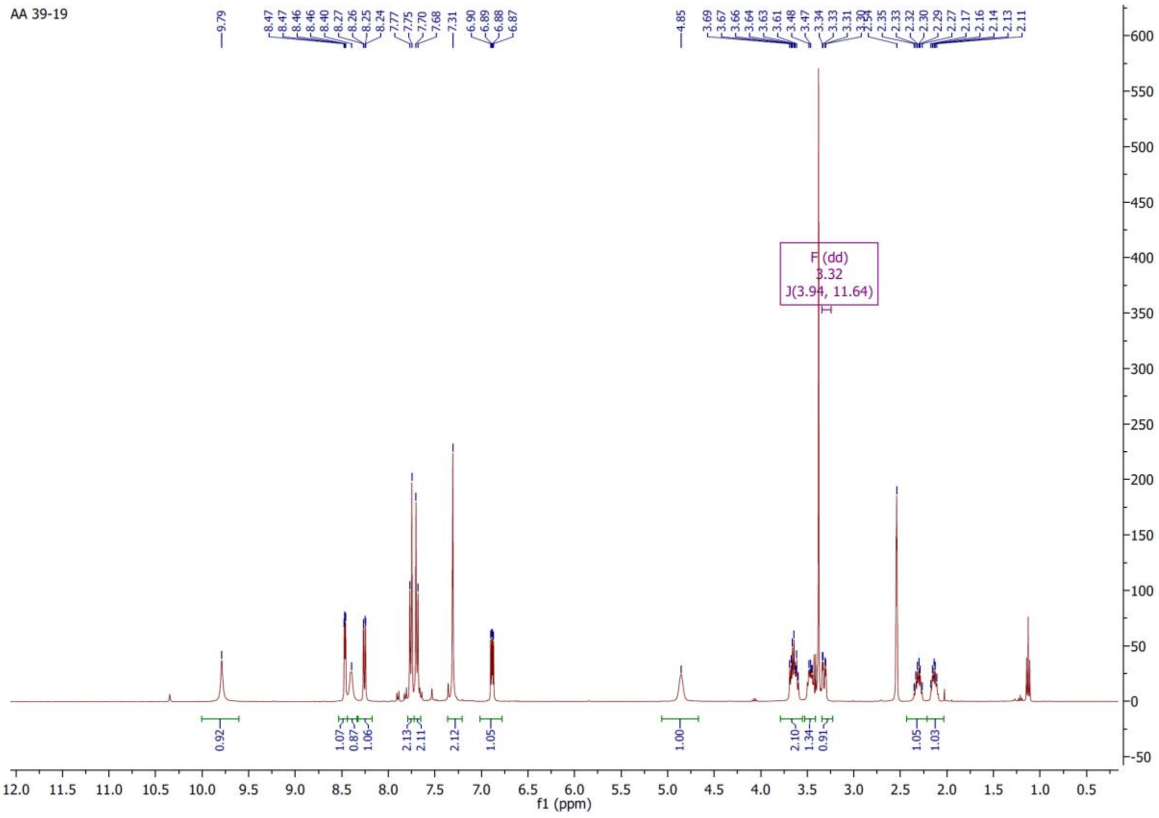

^1H NMR spectrum of compound **37a** (400 MHz, DMSO-d_6)

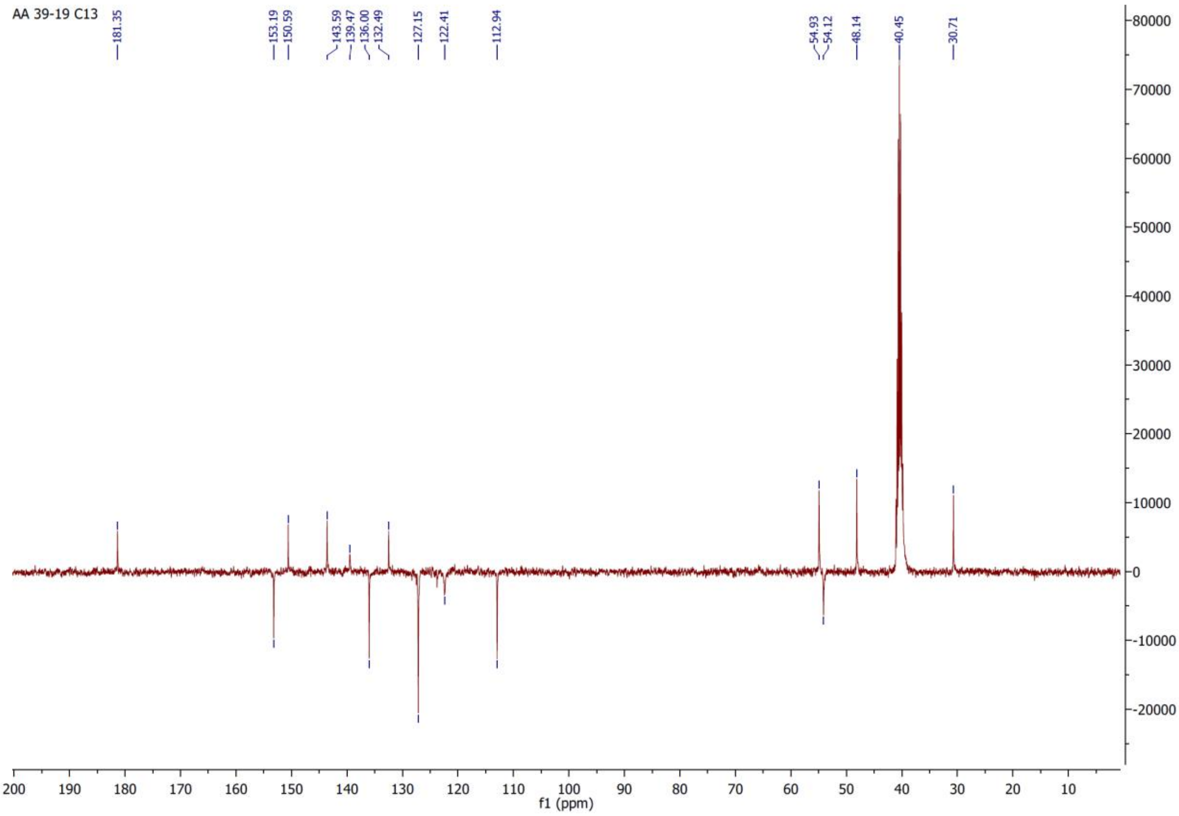

^{13}C NMR spectrum of compound **37a** (100 MHz, DMSO-d_6)

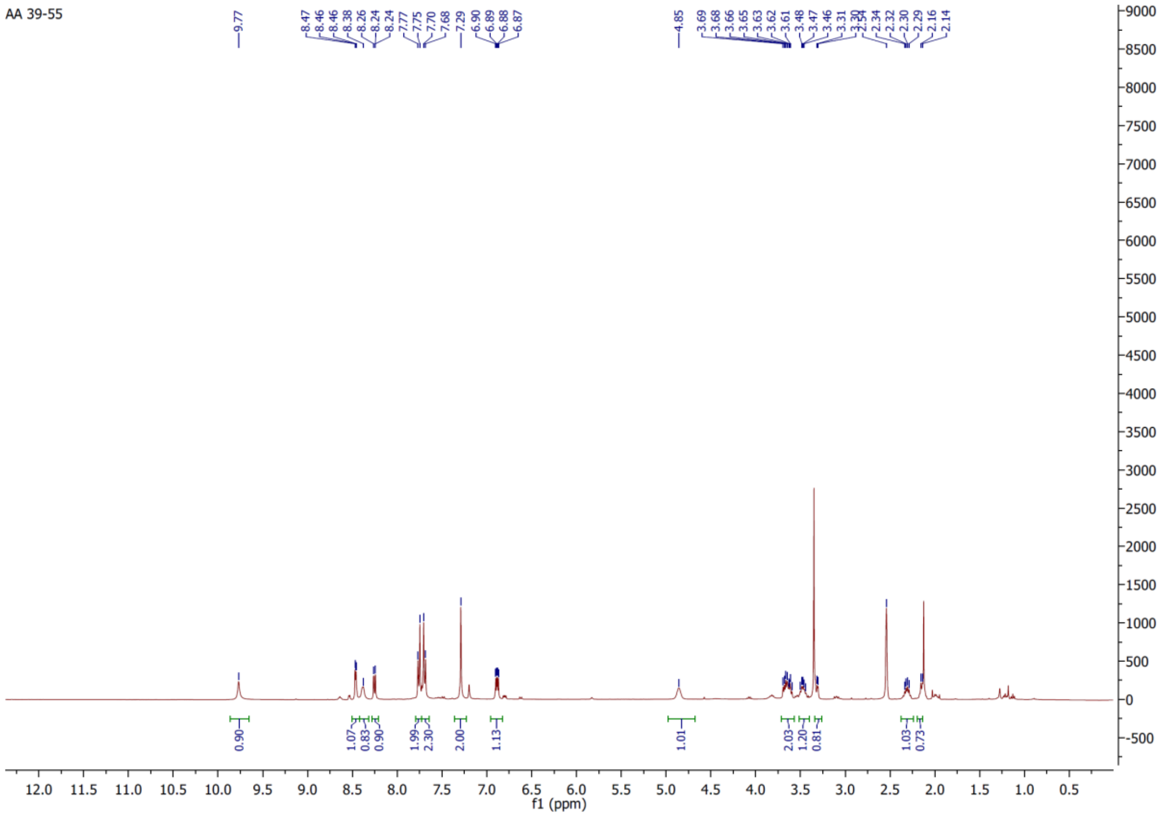

^{1}H NMR spectrum of compound **37b** (400 MHz, DMSO-d_6)

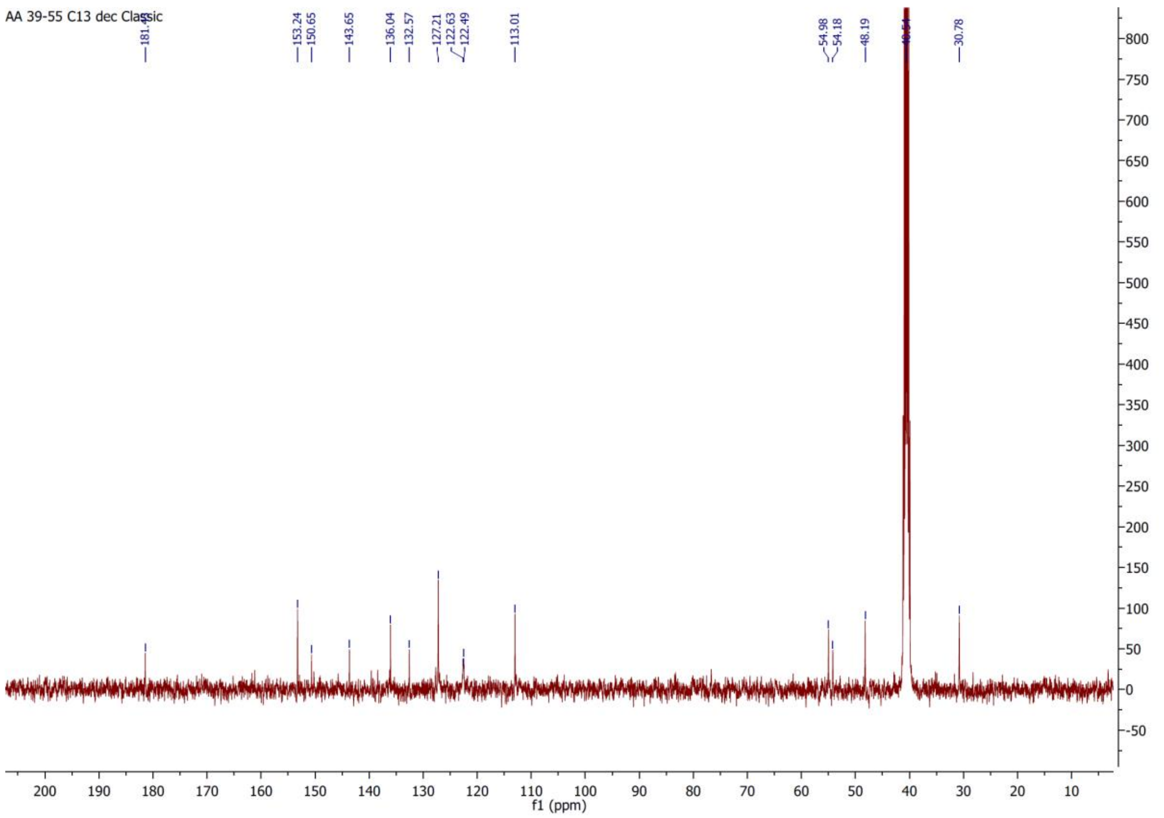

^{13}C NMR spectrum of compound **37b** (100 MHz, DMSO-d_6)

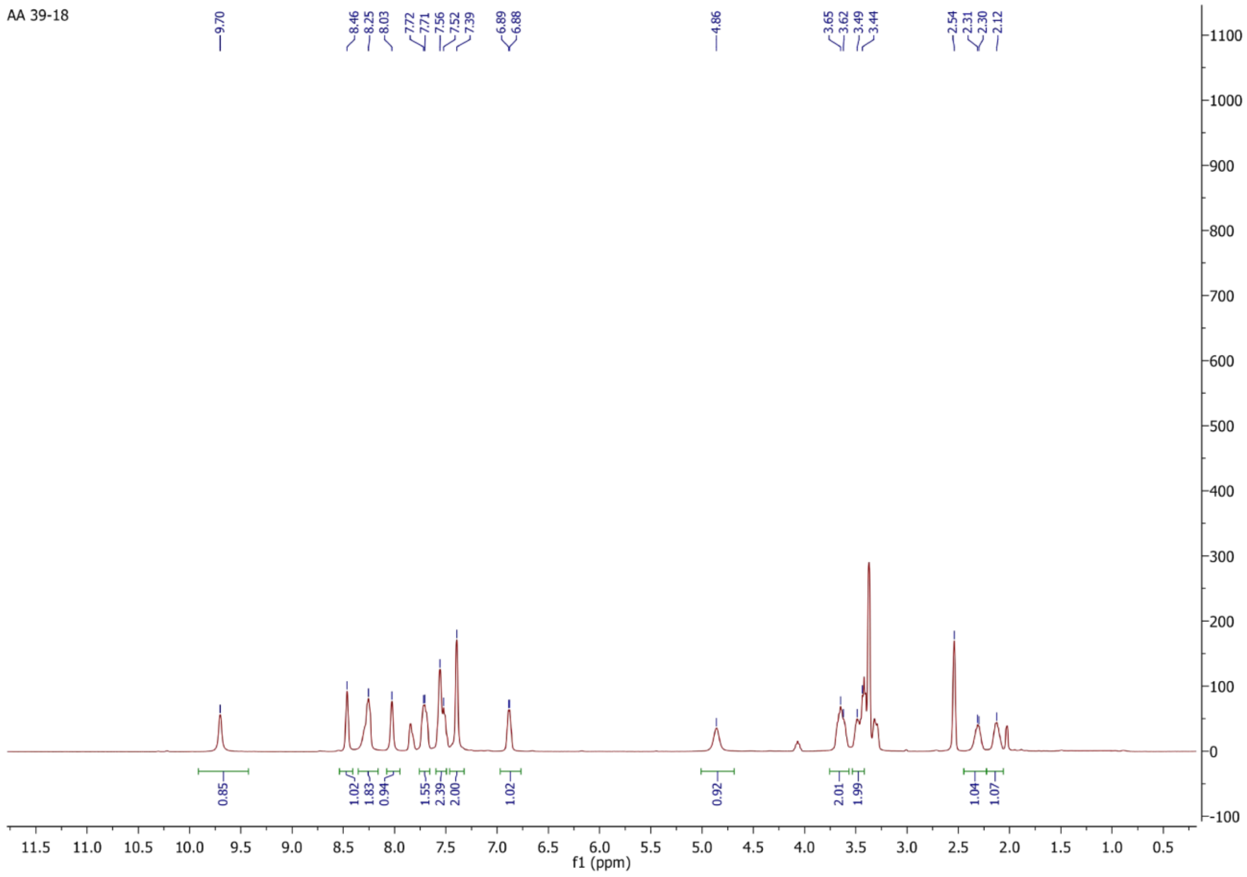

^{1}H NMR spectrum of compound **38a** (400 MHz, DMSO-d_6)

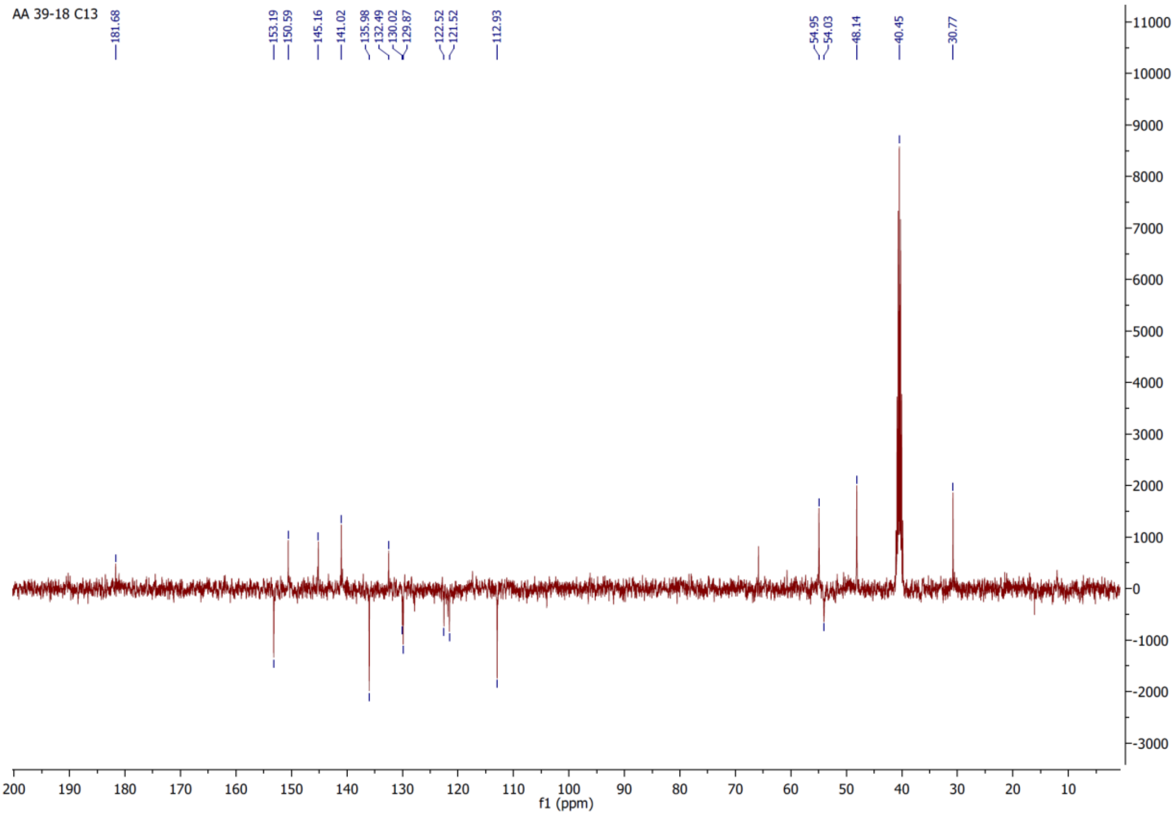

^{13}C NMR spectrum of compound **38a** (100 MHz, DMSO-d_6)

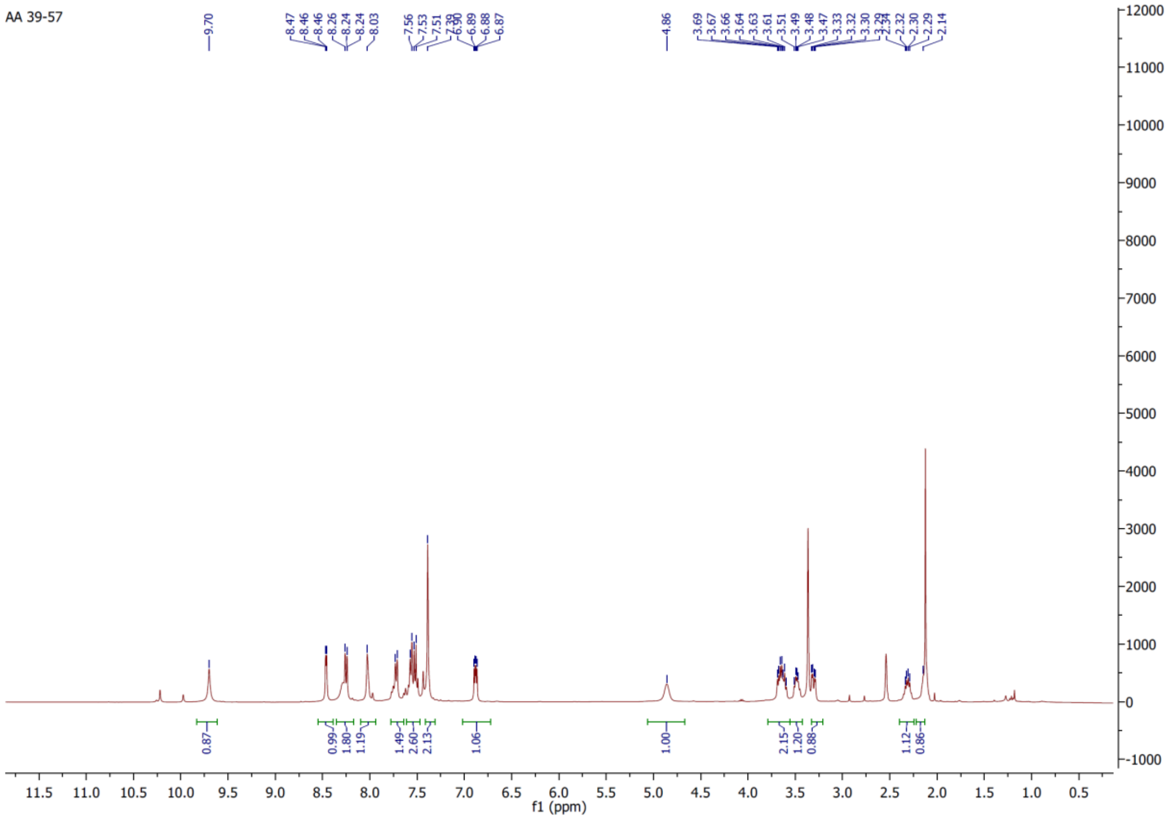

^{1}H NMR spectrum of compound **38b** (400 MHz, DMSO-d_6)

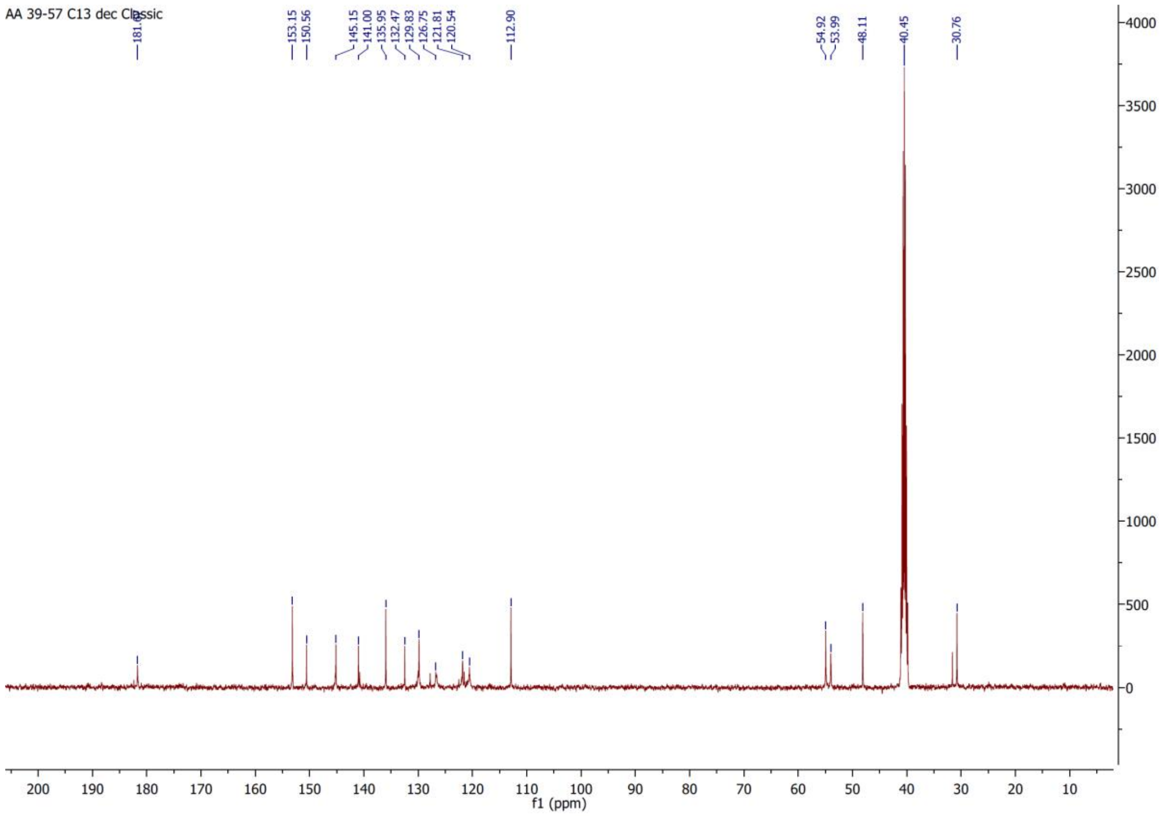

^{13}C NMR spectrum of compound **38b** (100 MHz, DMSO-d_6)

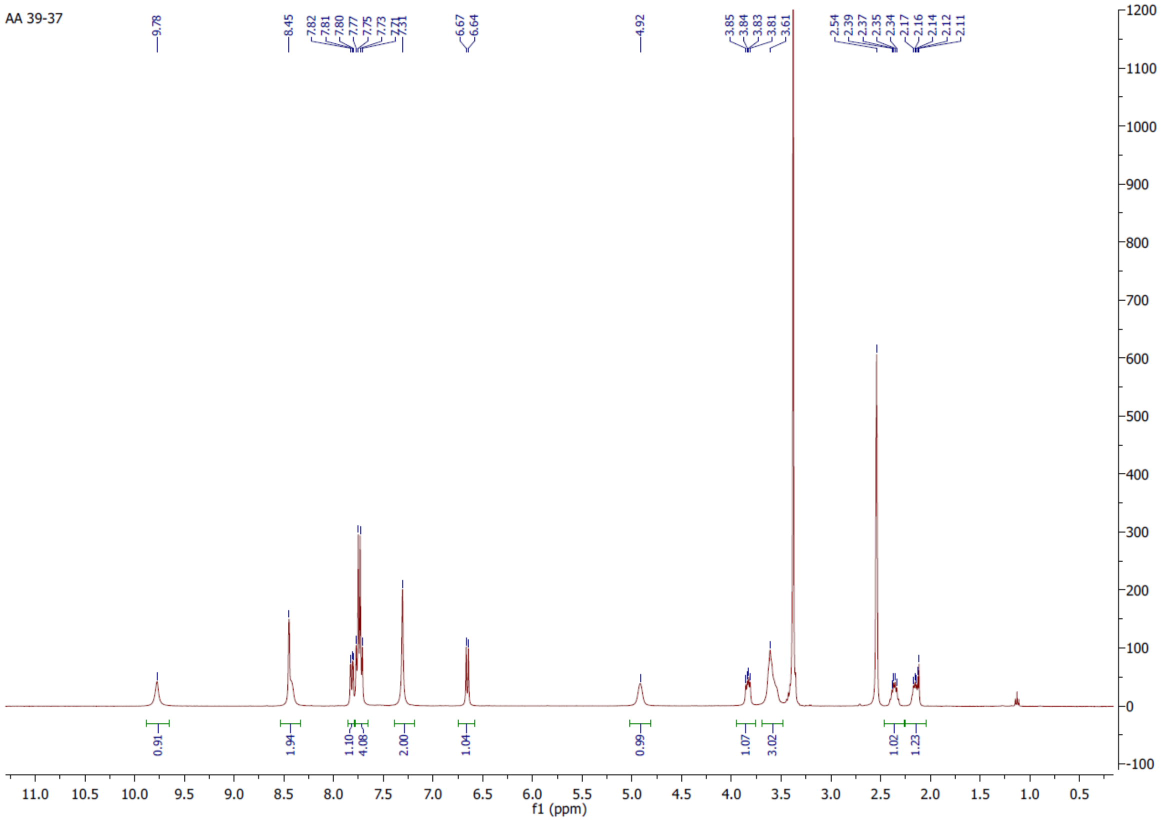

^{1}H NMR spectrum of compound **39a** (400 MHz, DMSO-d_6)

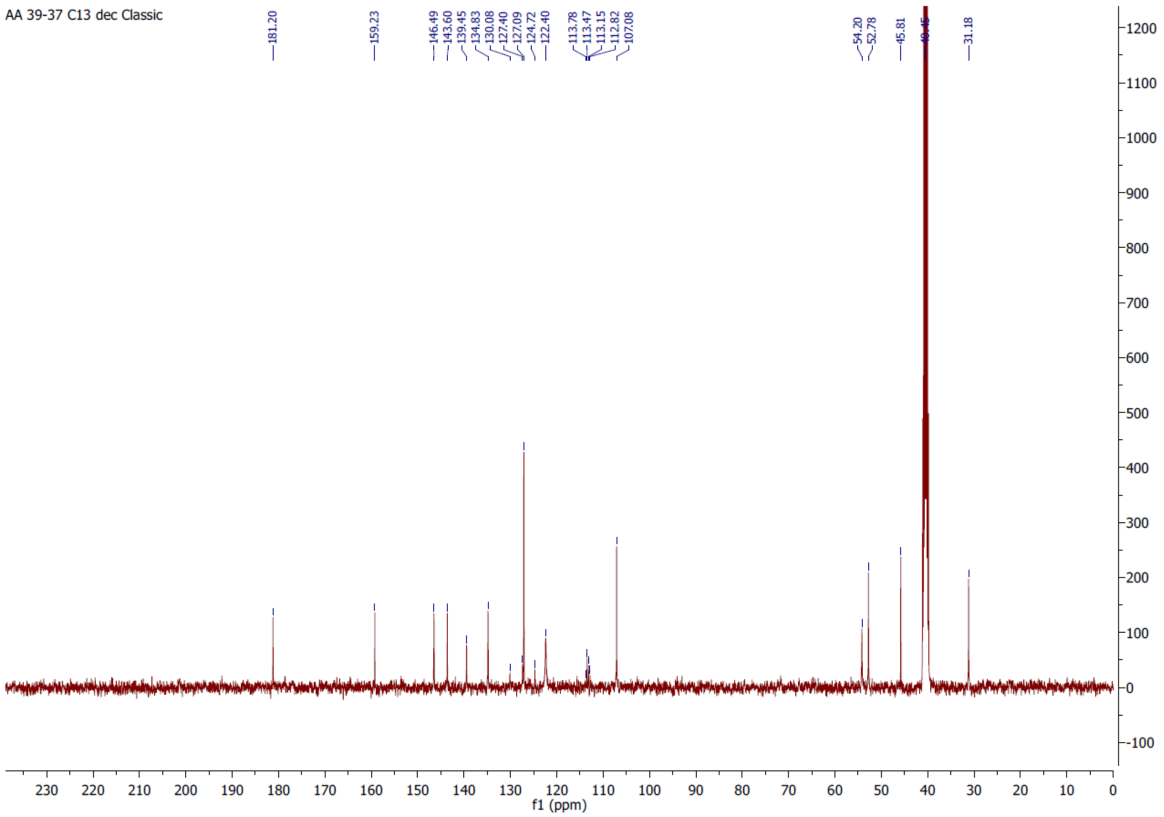

^{13}C NMR spectrum of compound **39a** (100 MHz, DMSO-d_6)

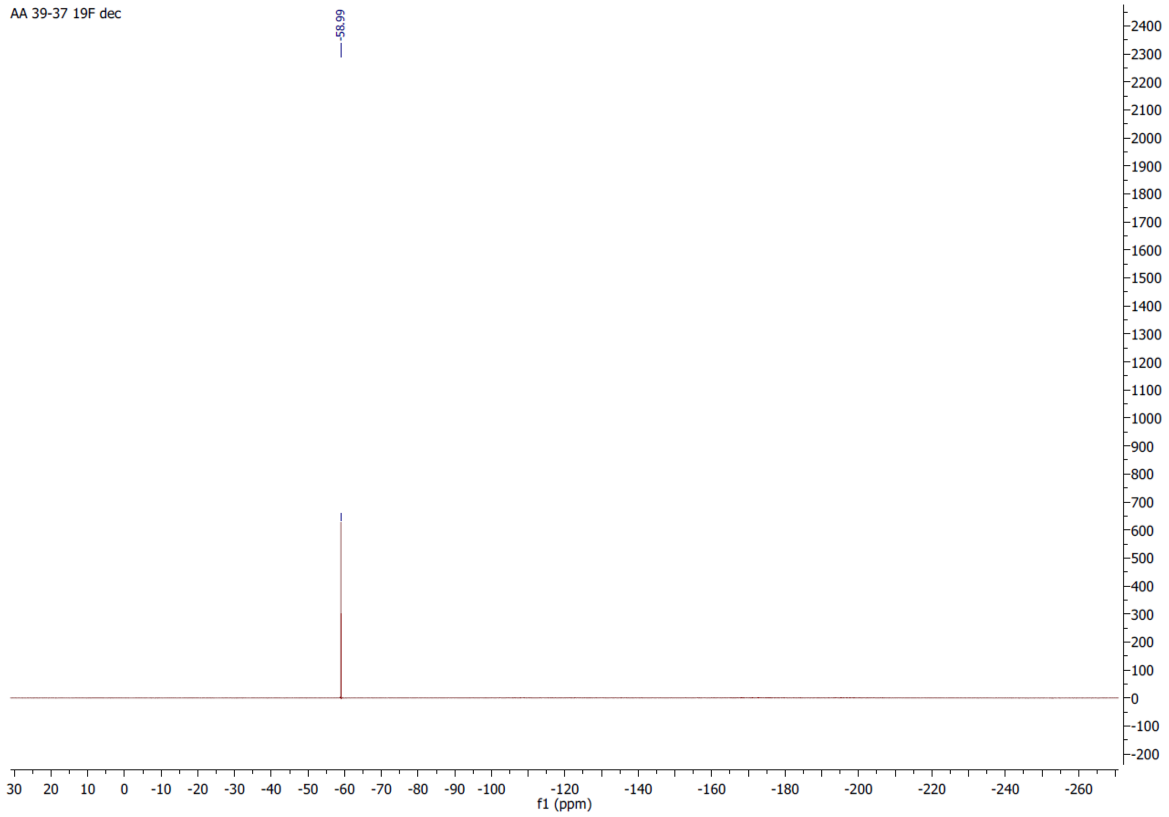

^{19}F NMR spectrum of compound **39a** (100 MHz, DMSO-d_6)

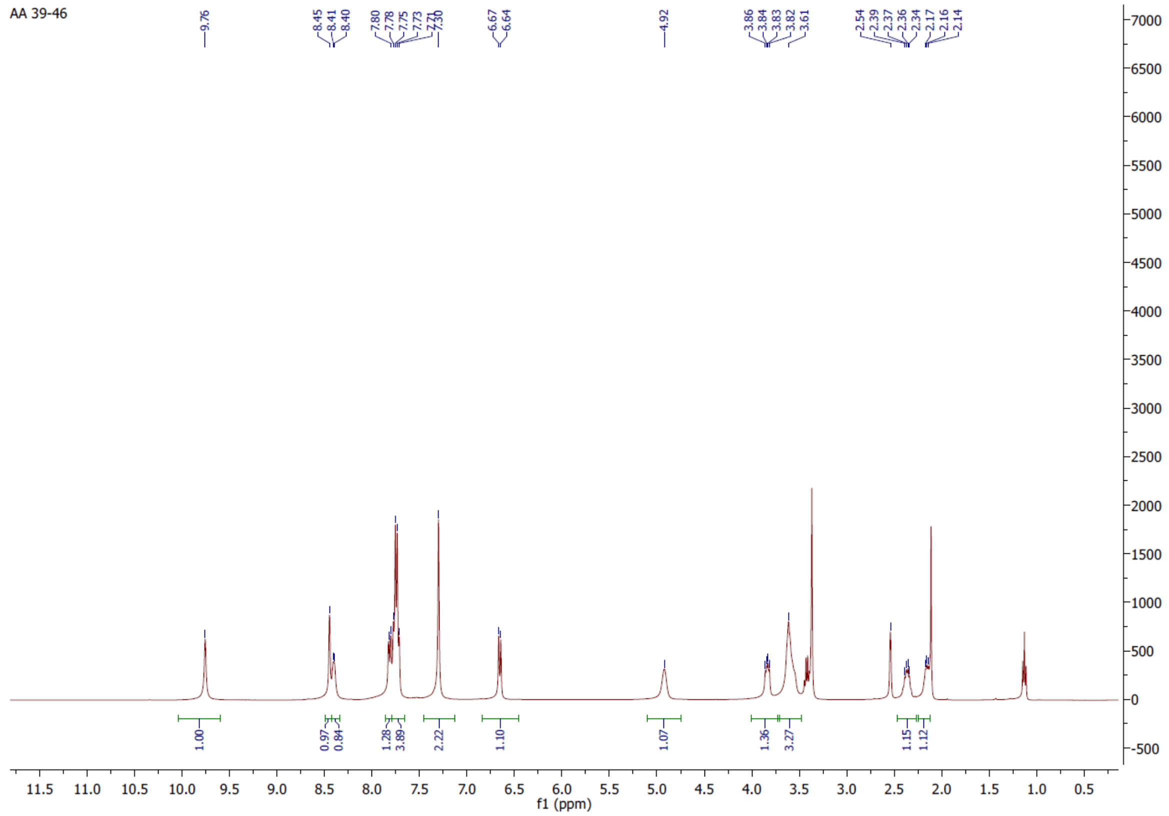

^{1}H NMR spectrum of compound **39b** (400 MHz, DMSO-d_6)

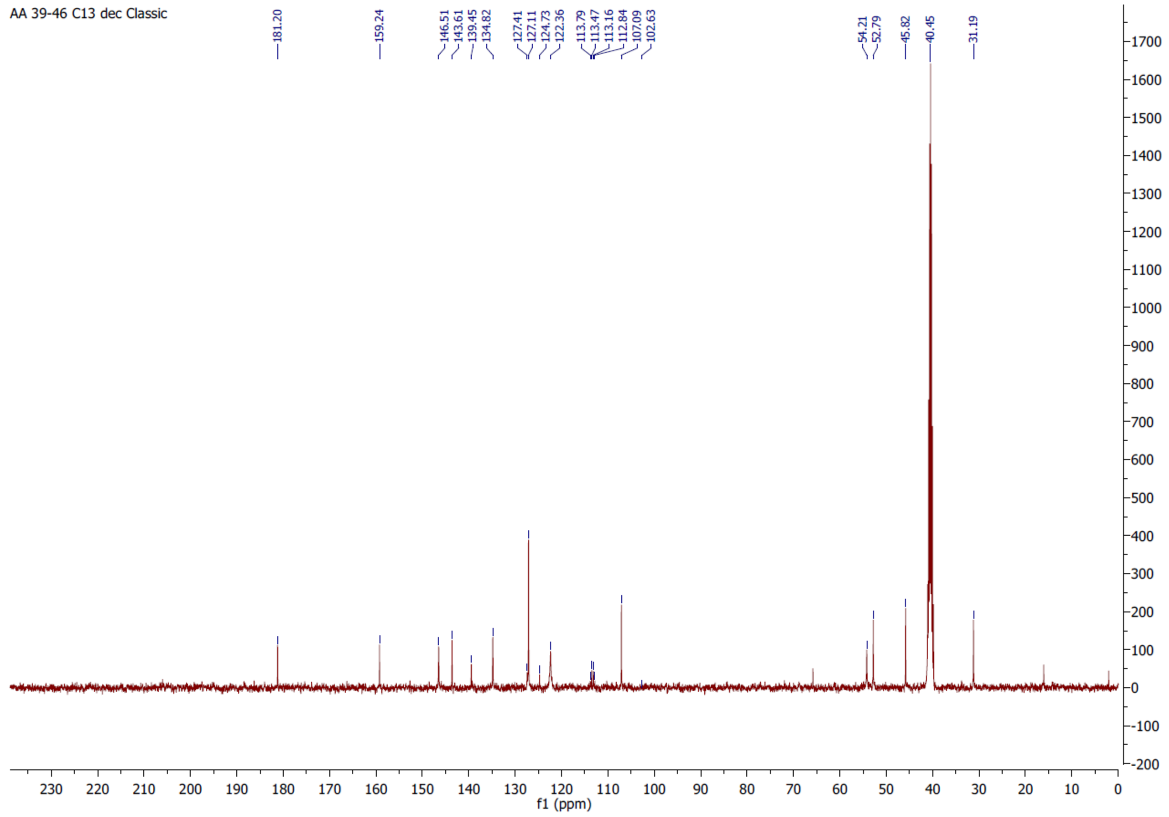

^{13}C NMR spectrum of compound **39b** (100 MHz, DMSO-d_6)

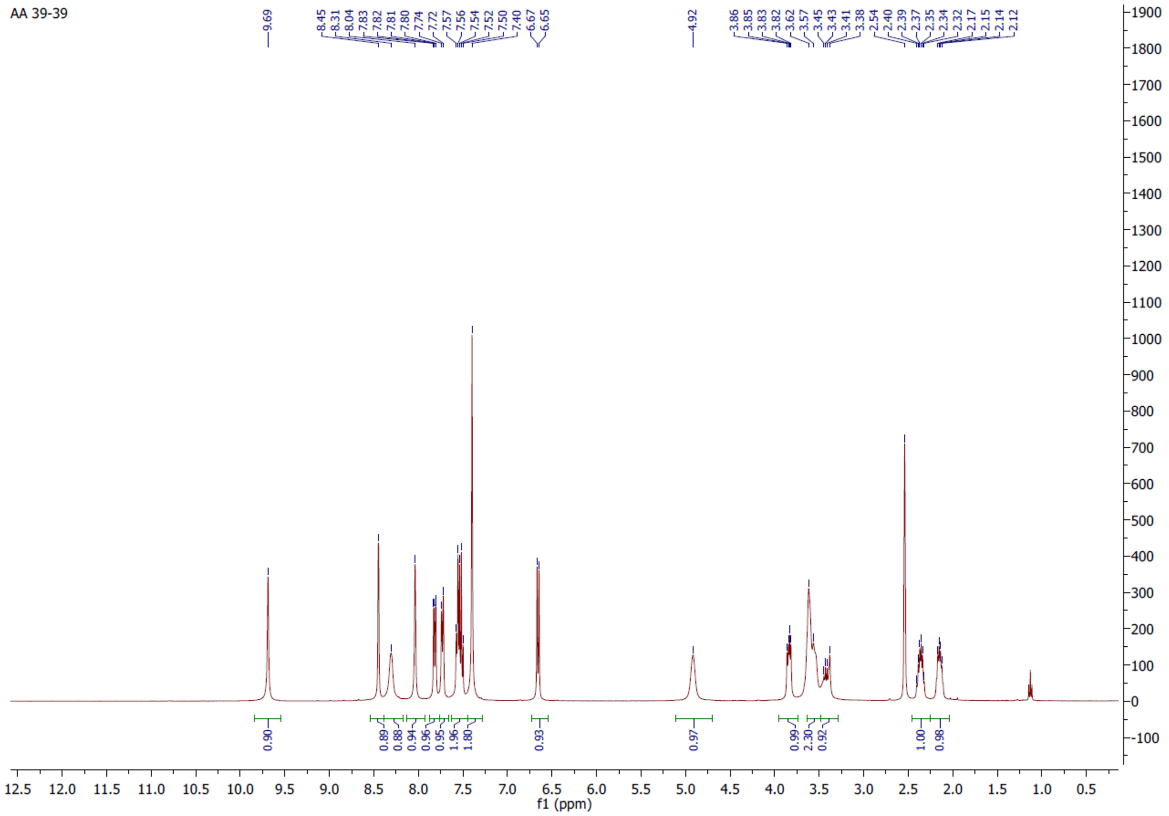

^{1}H NMR spectrum of compound **40a** (400 MHz, DMSO-d_6)

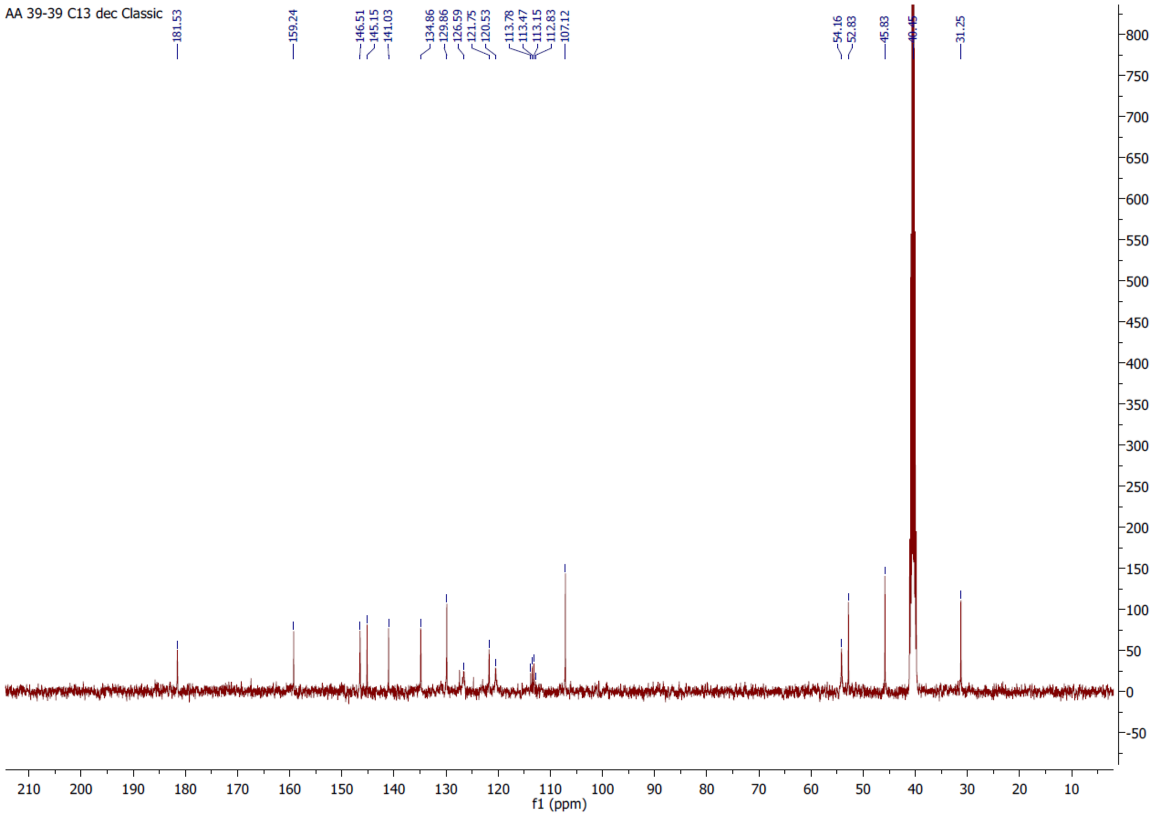

^{13}C NMR spectrum of compound **40a** (100 MHz, DMSO-d_6)

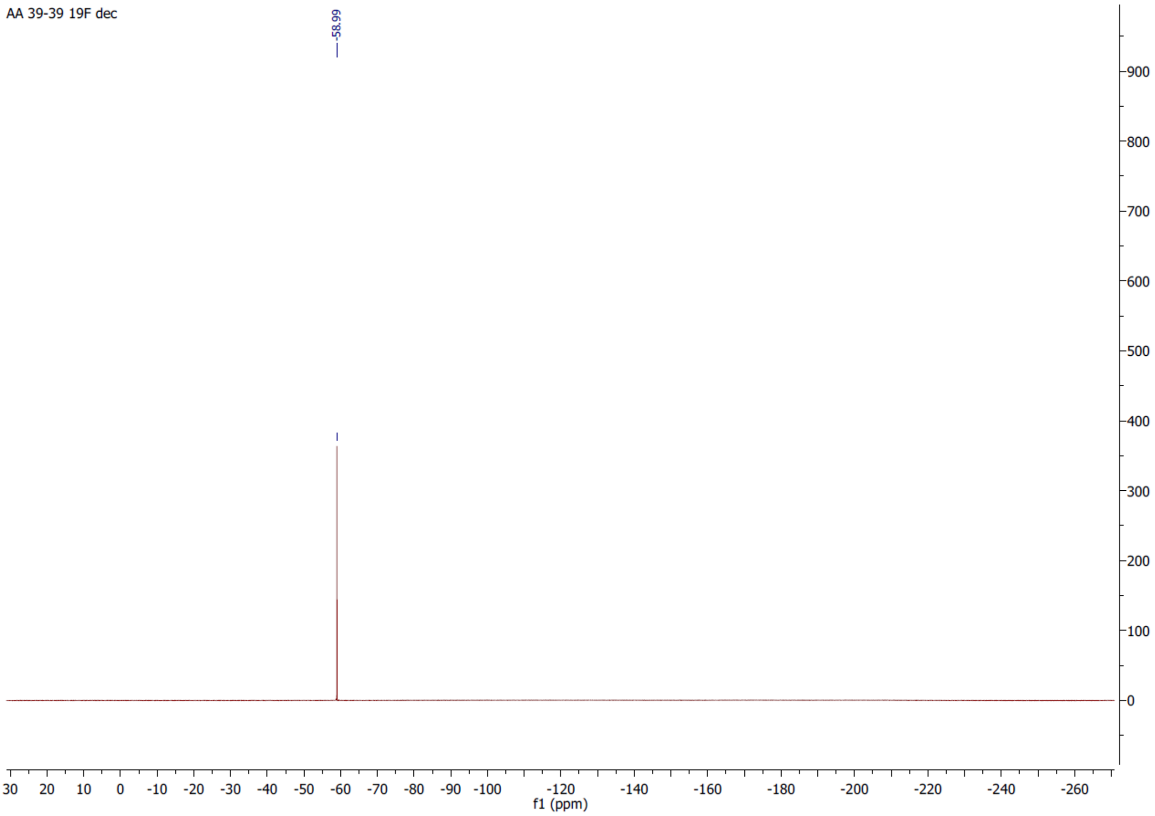

^{19}F NMR spectrum of compound **40a** (376 MHz, DMSO-d_6)

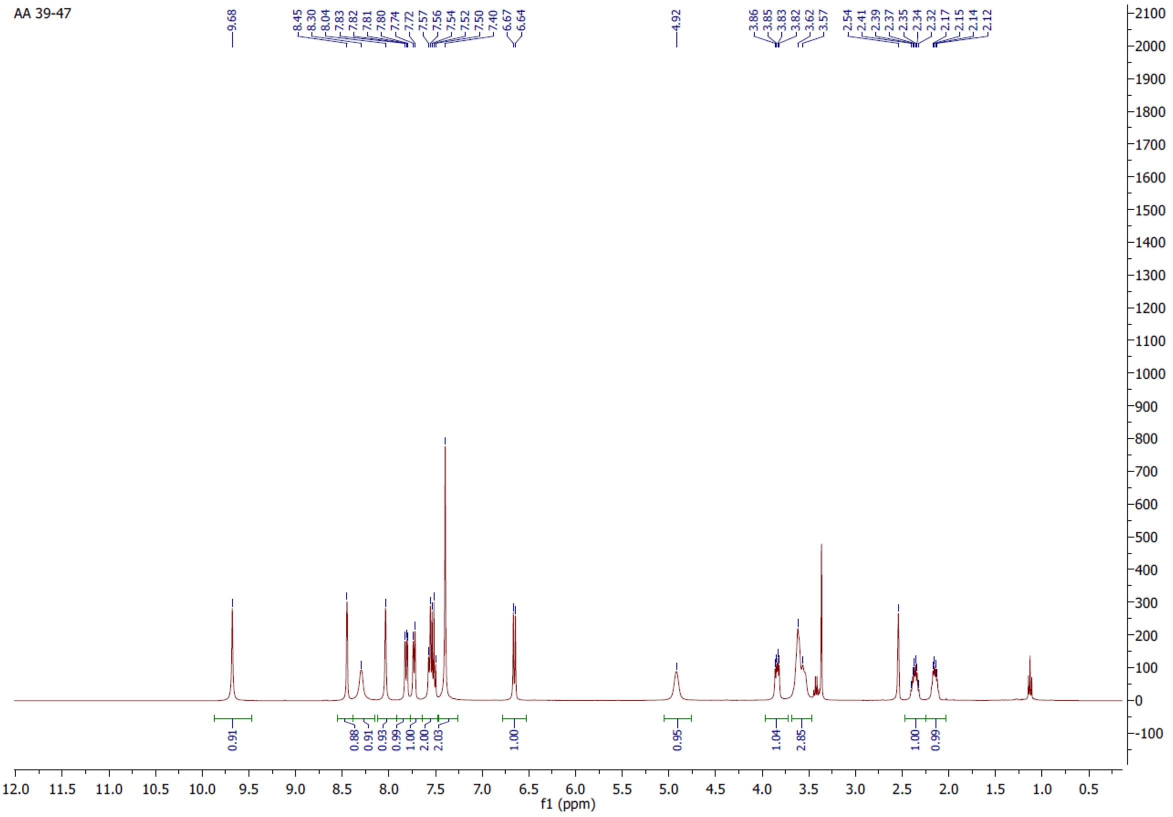

^{1}H NMR spectrum of compound **40b** (400 MHz, DMSO-d_6)

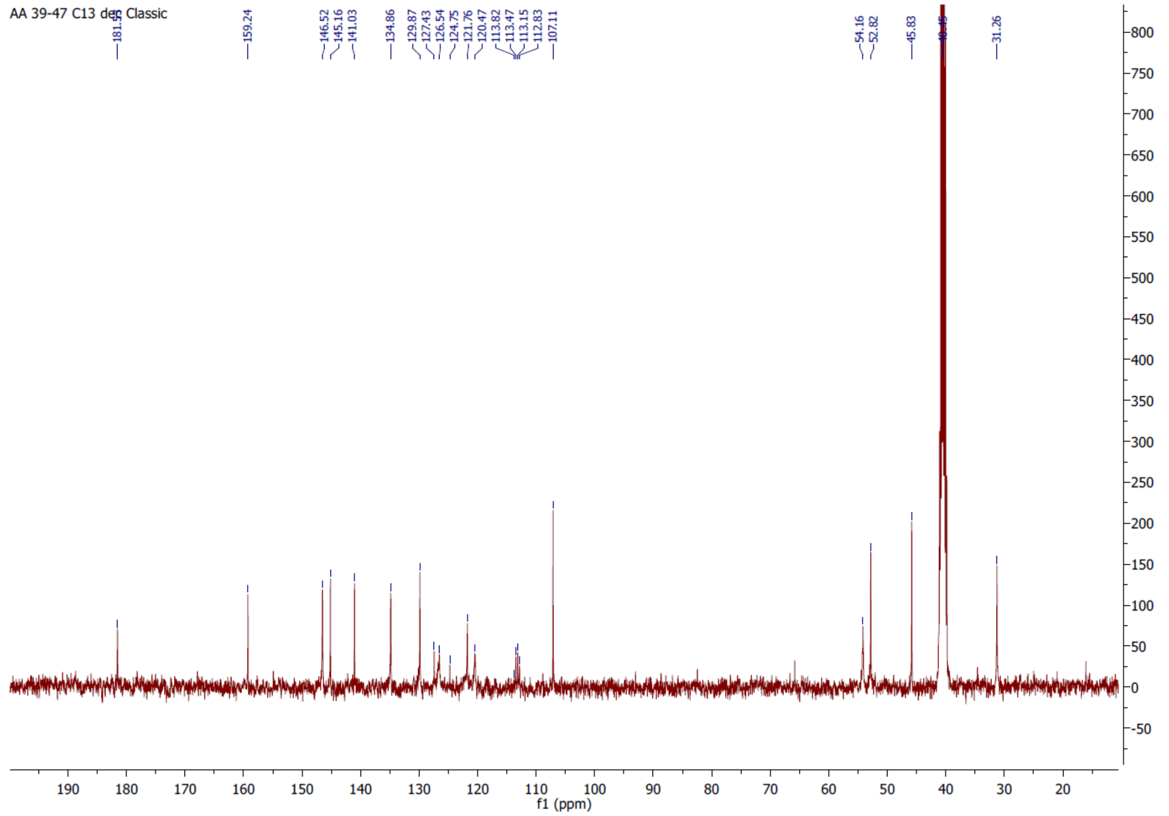

^{13}C NMR spectrum of compound **40b** (100 MHz, DMSO-d_6)

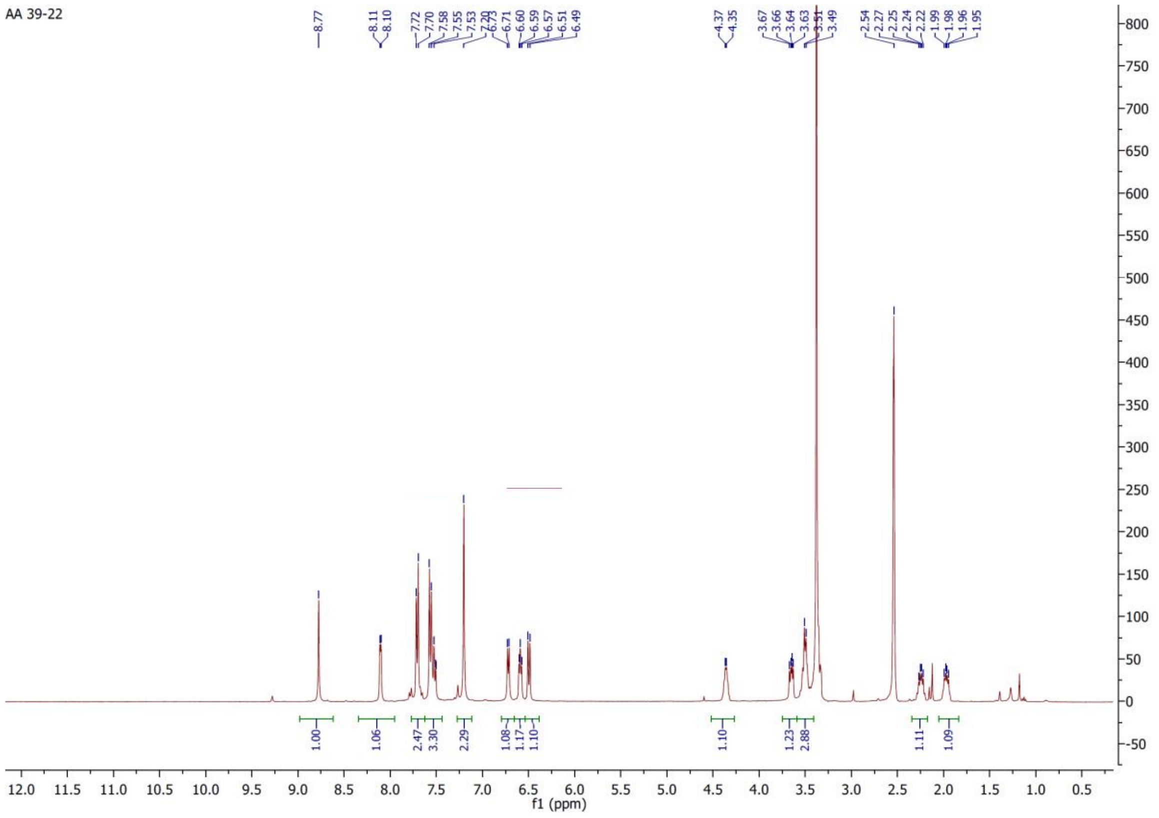

^{1}H NMR spectrum of compound **41a** (400 MHz, DMSO-d_6)

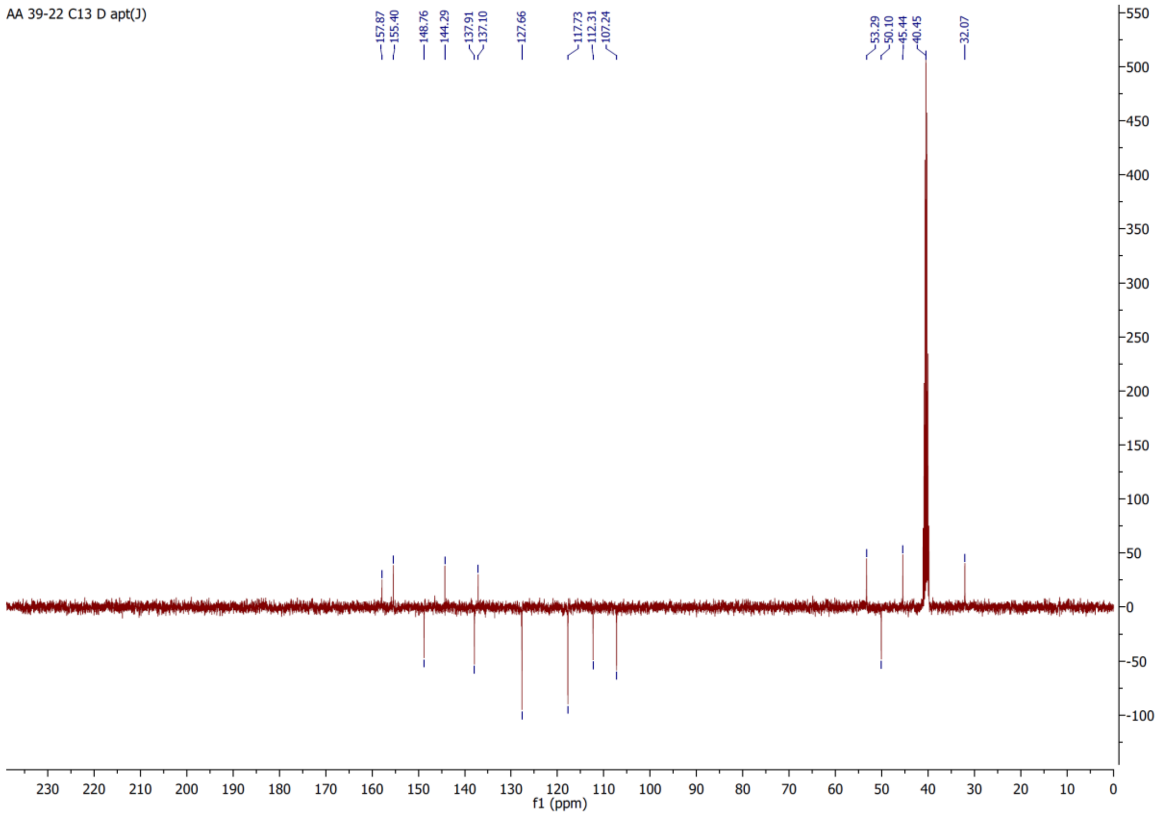

^{13}C NMR spectrum of compound **41a** (100 MHz, DMSO-d_6)

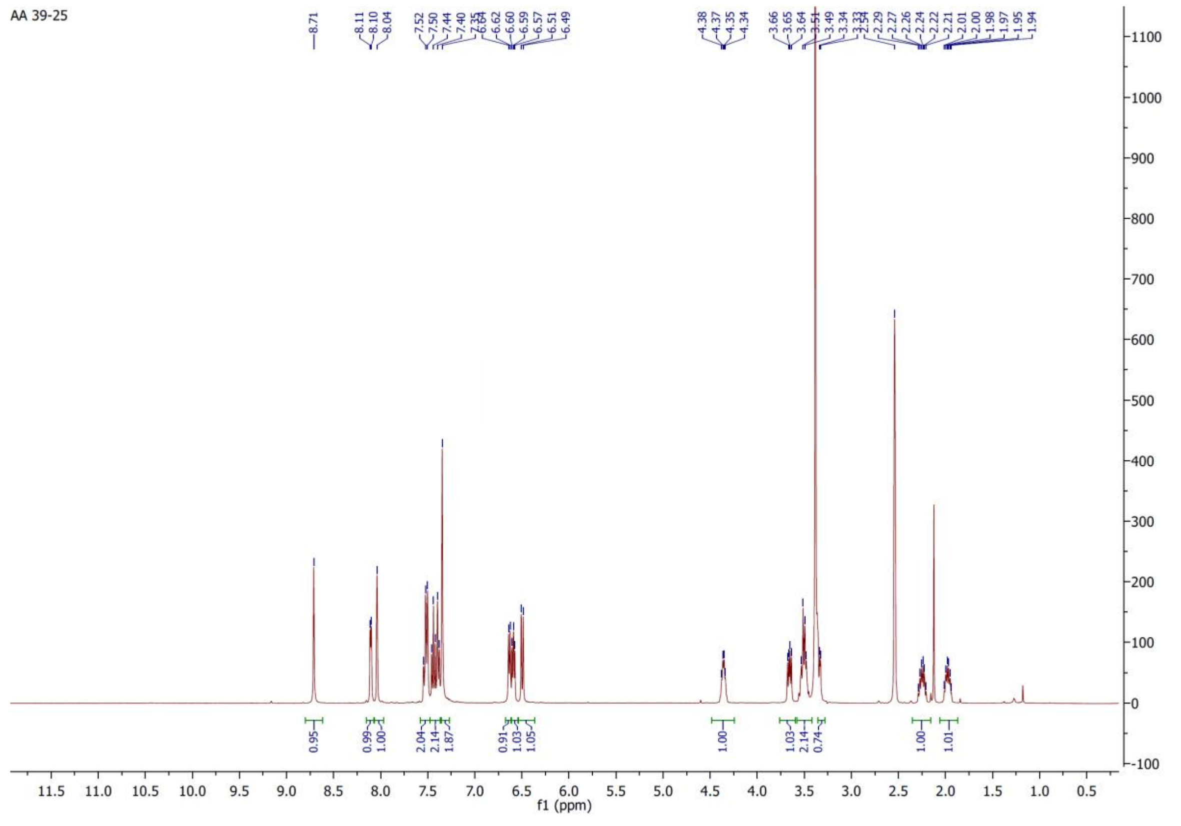

^{1}H NMR spectrum of compound **42a** (400 MHz, DMSO-d_6)

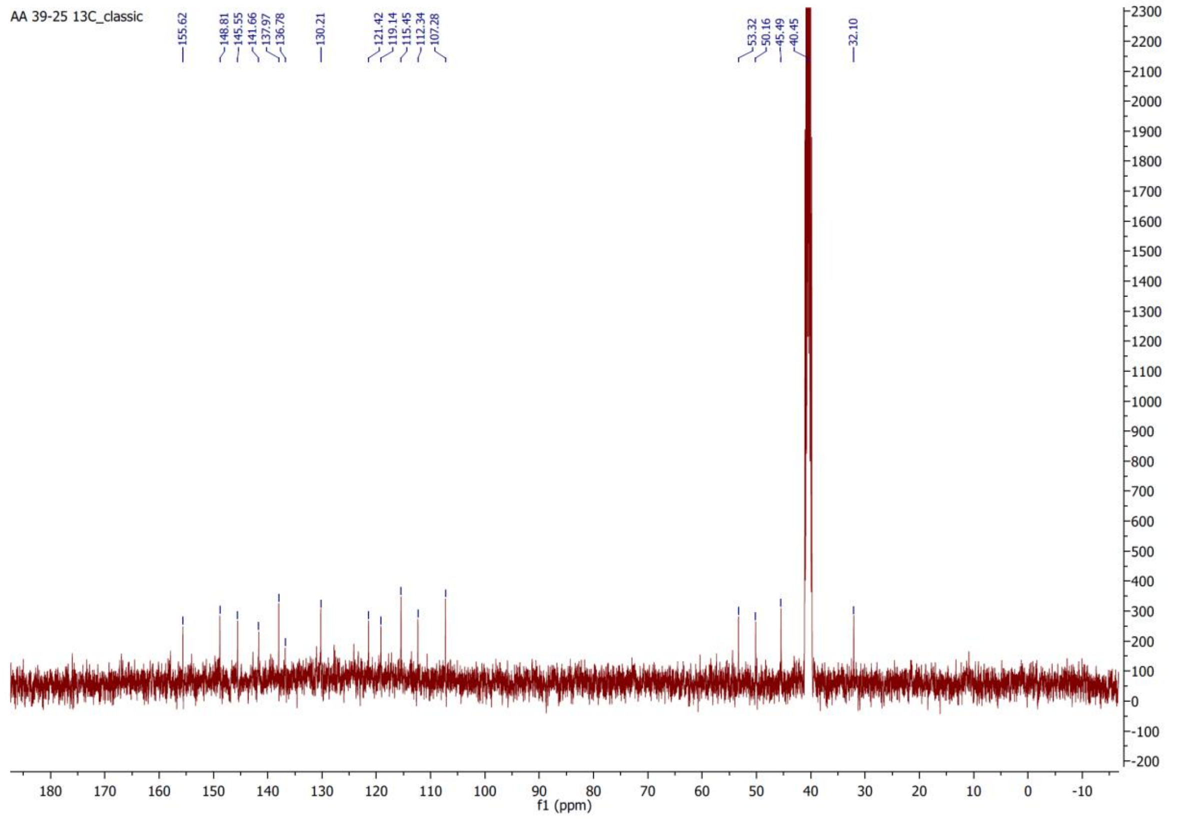

^{13}C NMR spectrum of compound **42a** (100 MHz, DMSO-d_6)

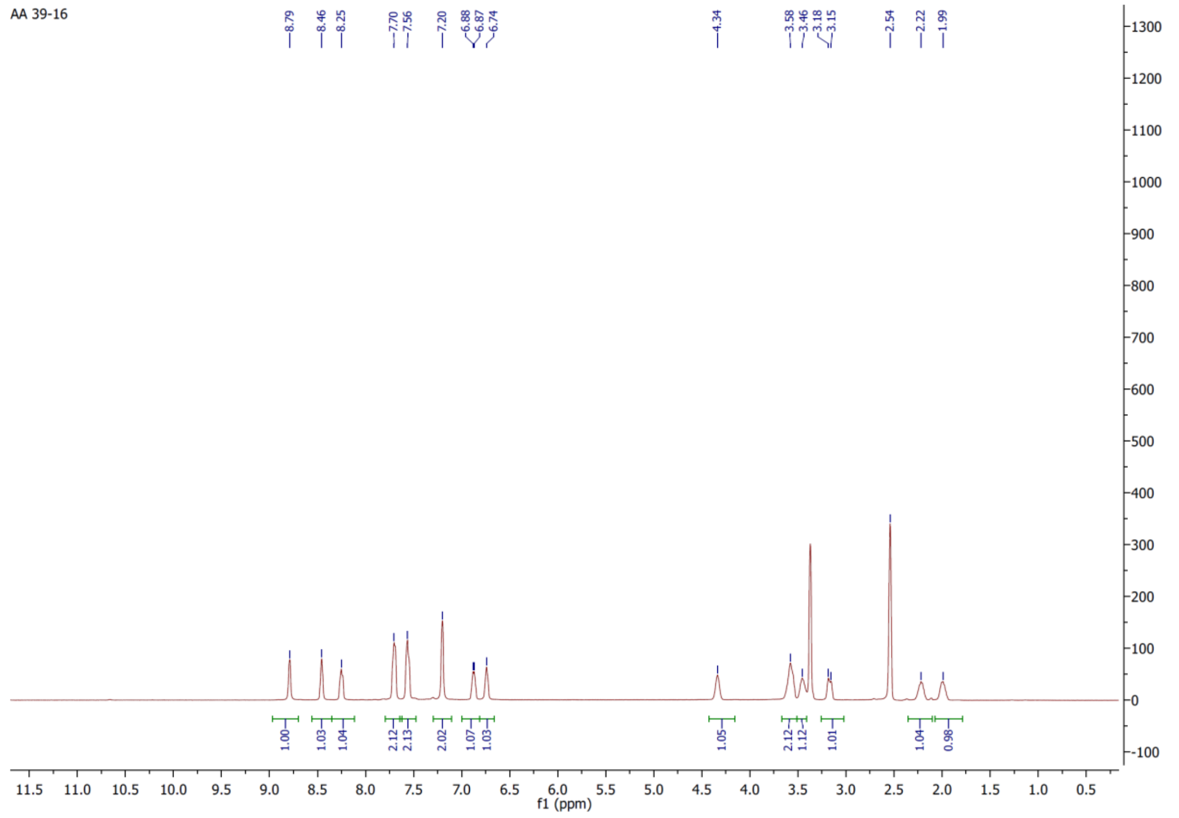

^{1}H NMR spectrum of compound **43a** (400 MHz, DMSO-d_6)

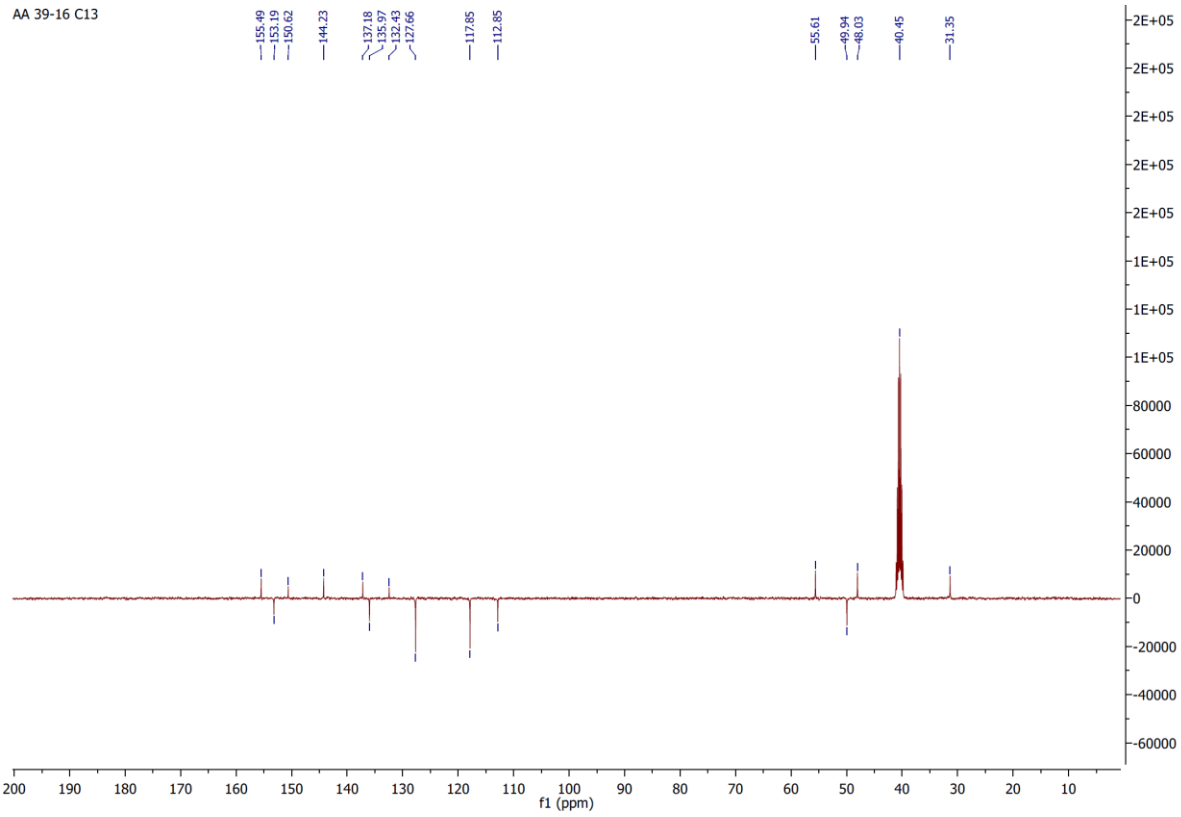

^{13}C NMR spectrum of compound **43a** (100 MHz, DMSO-d_6)

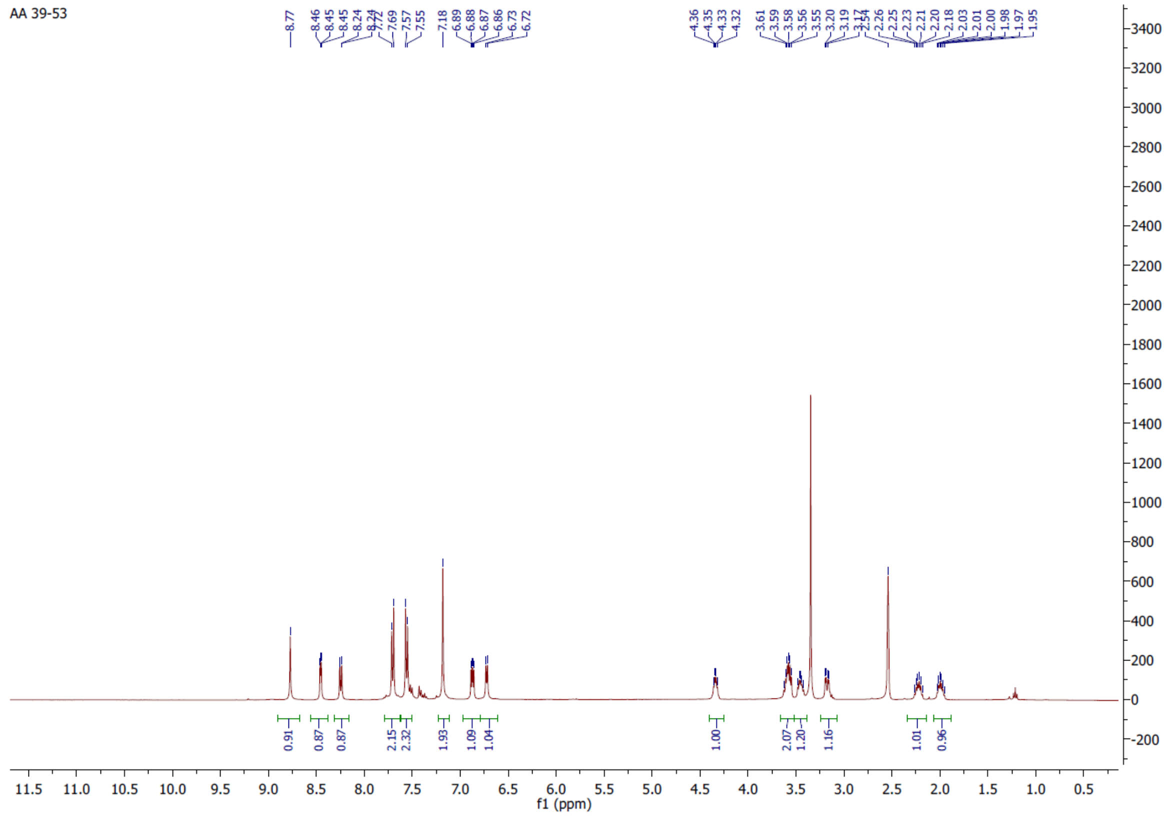

^{1}H NMR spectrum of compound **43b** (400 MHz, DMSO-d_6)

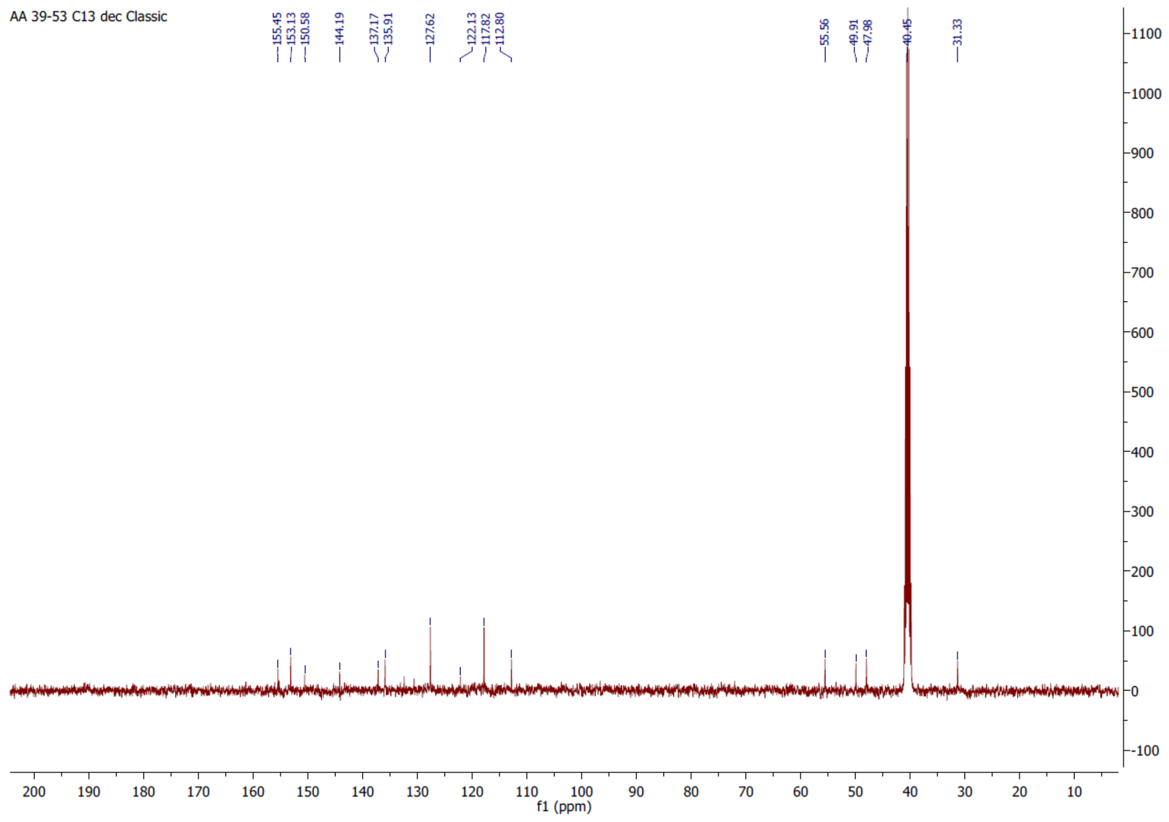

^{13}C NMR spectrum of compound **43b** (100 MHz, DMSO-d_6)

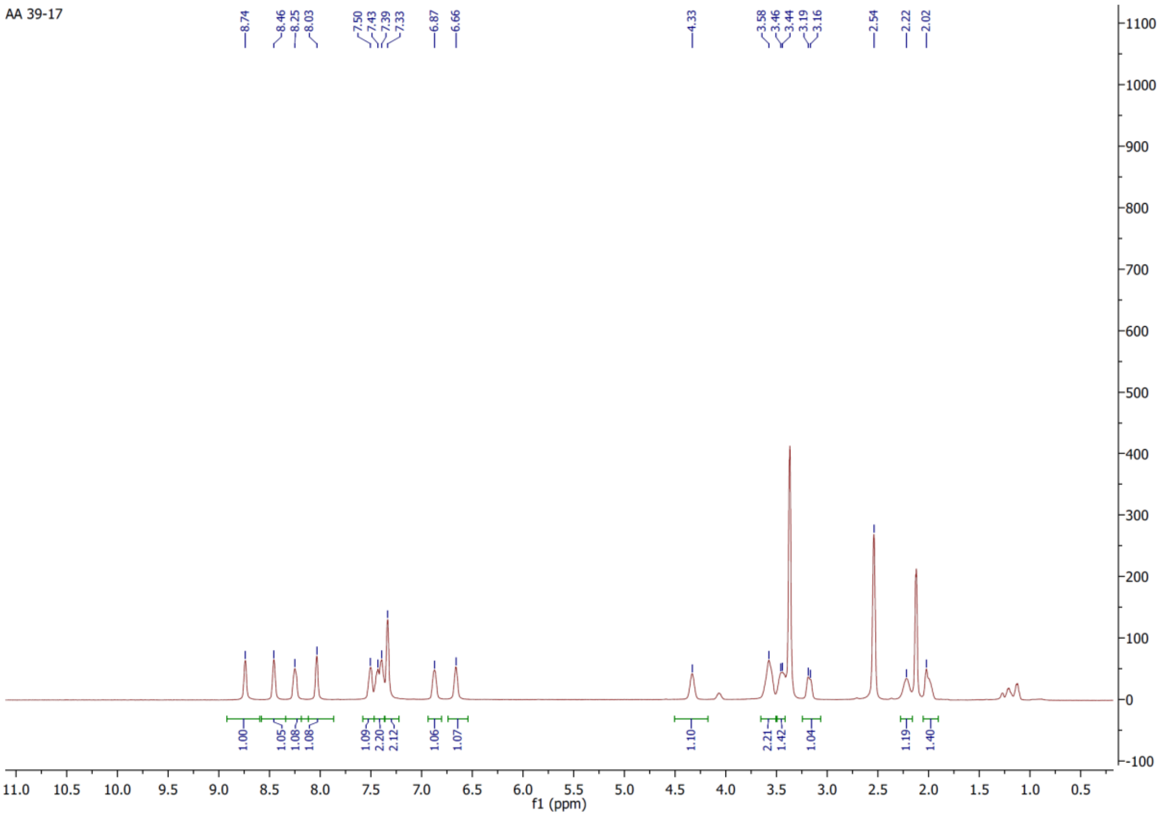

^{1}H NMR spectrum of compound **44a** (400 MHz, DMSO-d_6)

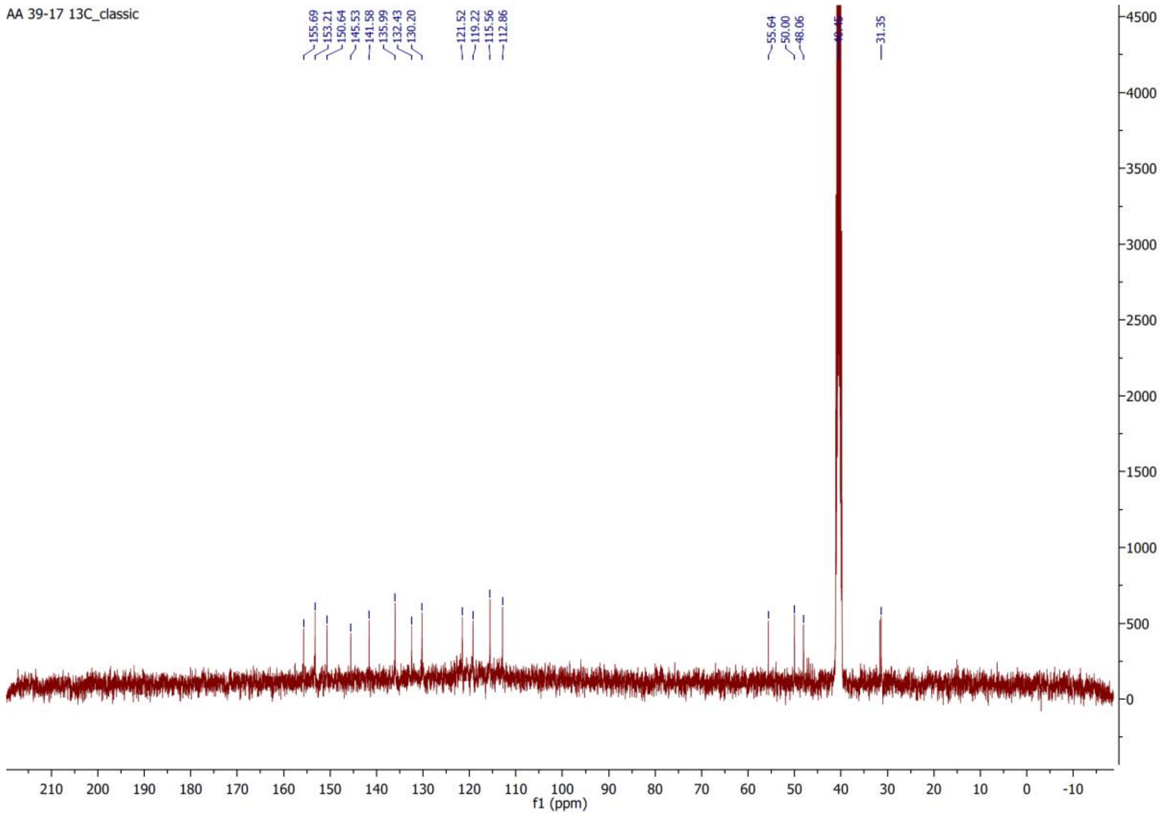

^{13}C NMR spectrum of compound **44a** (100 MHz, DMSO-d_6)

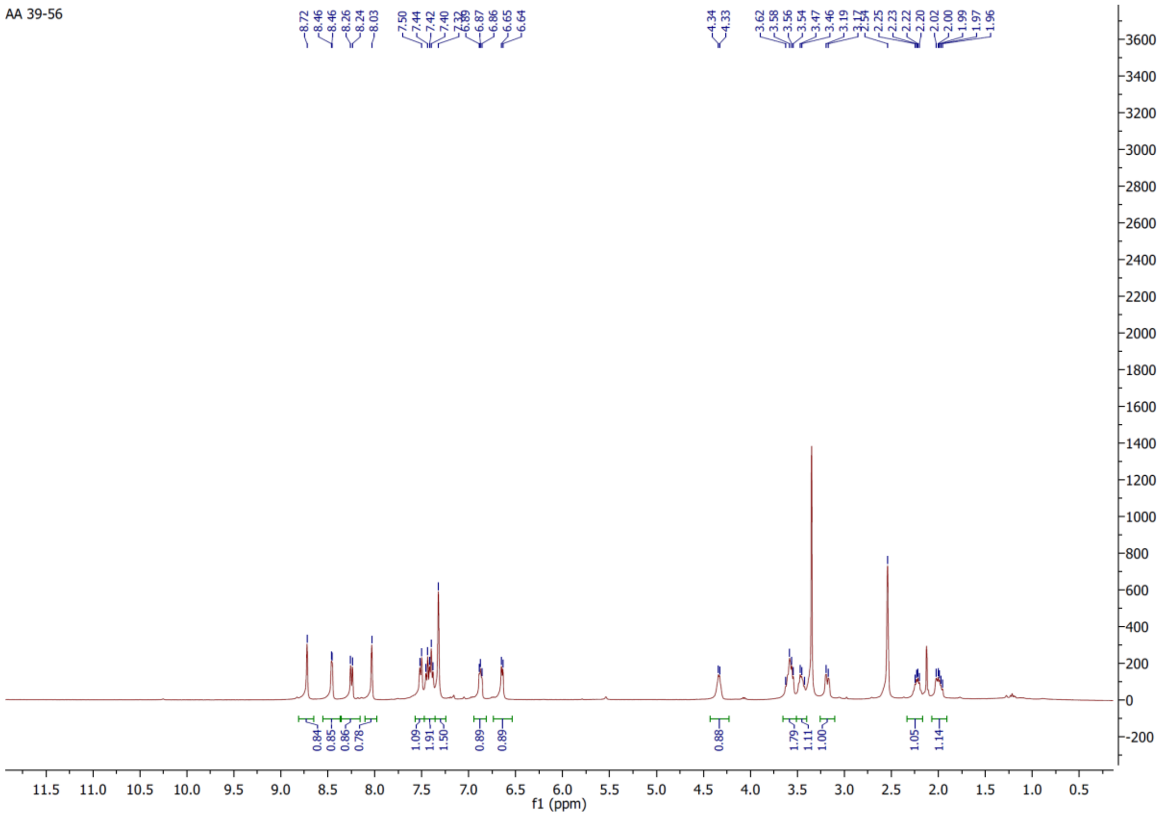

^{1}H NMR spectrum of compound **44b** (400 MHz, DMSO-d_6)

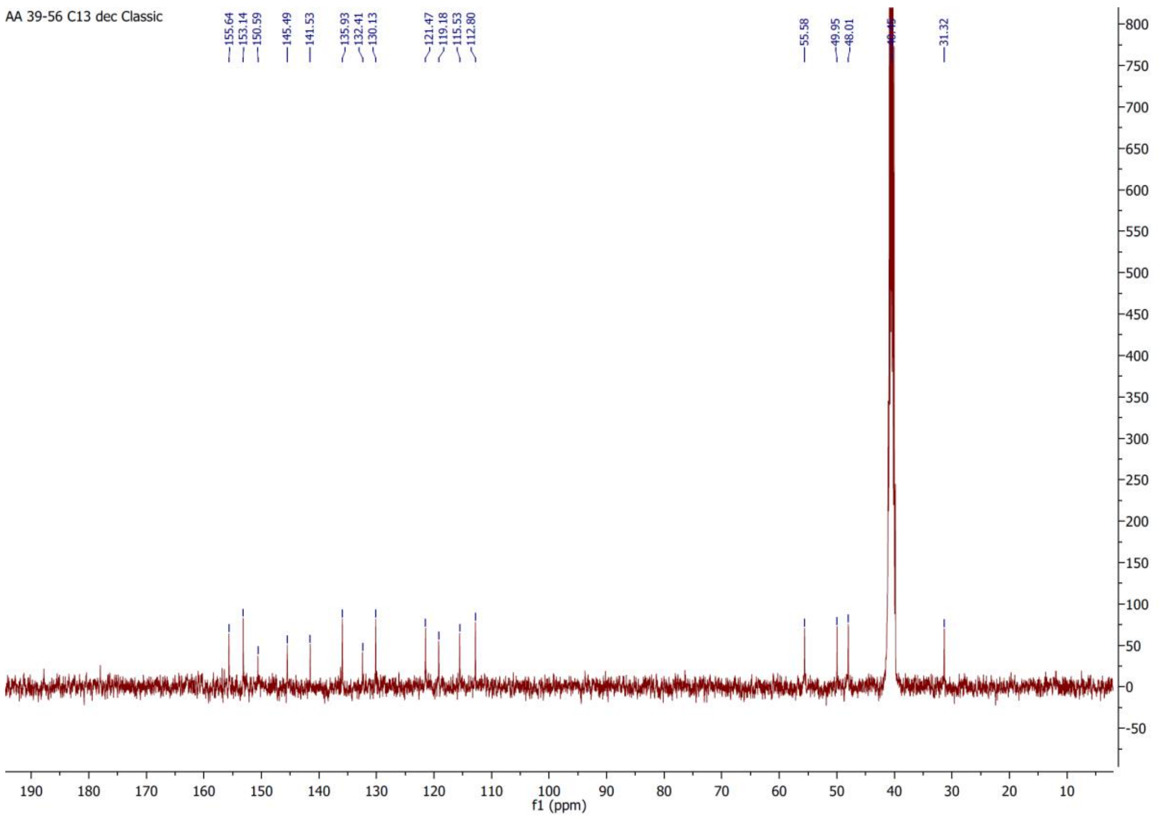

^{13}C NMR spectrum of compound **44b** (100 MHz, DMSO-d_6)

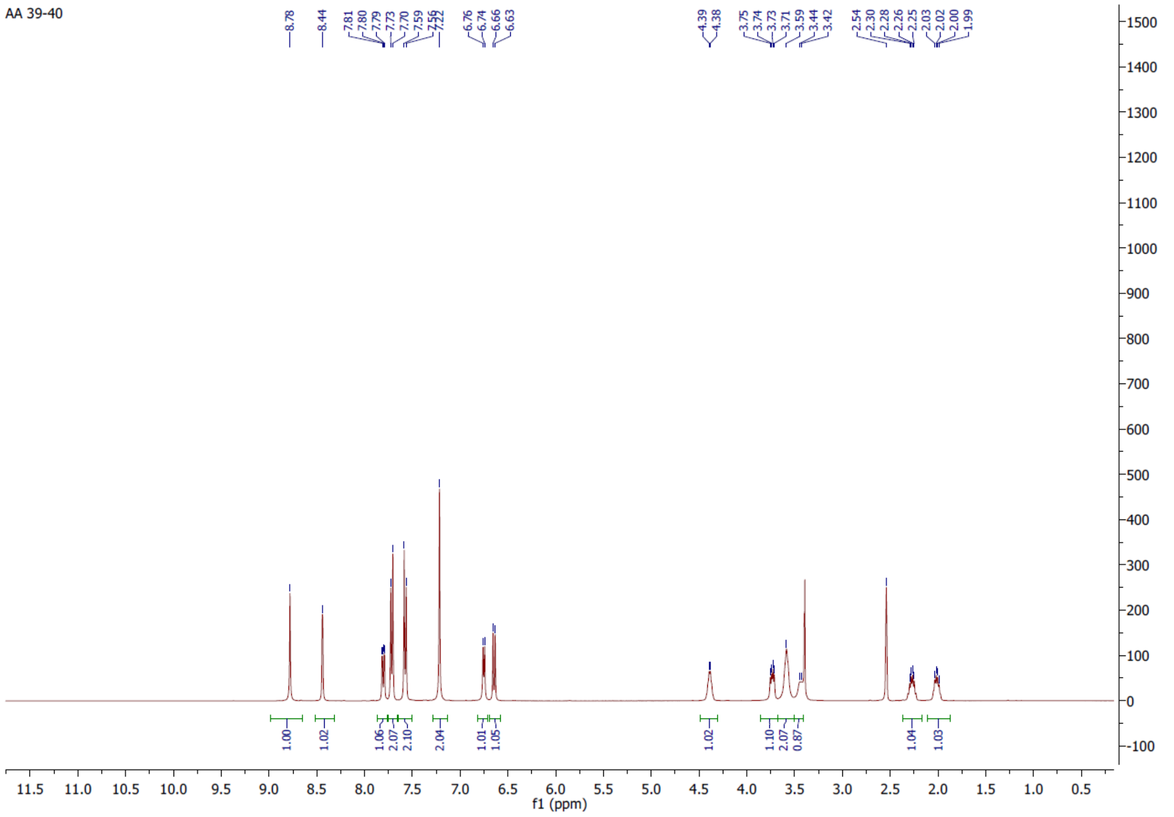

^{1}H NMR spectrum of compound **45a** (400 MHz, DMSO-d_6)

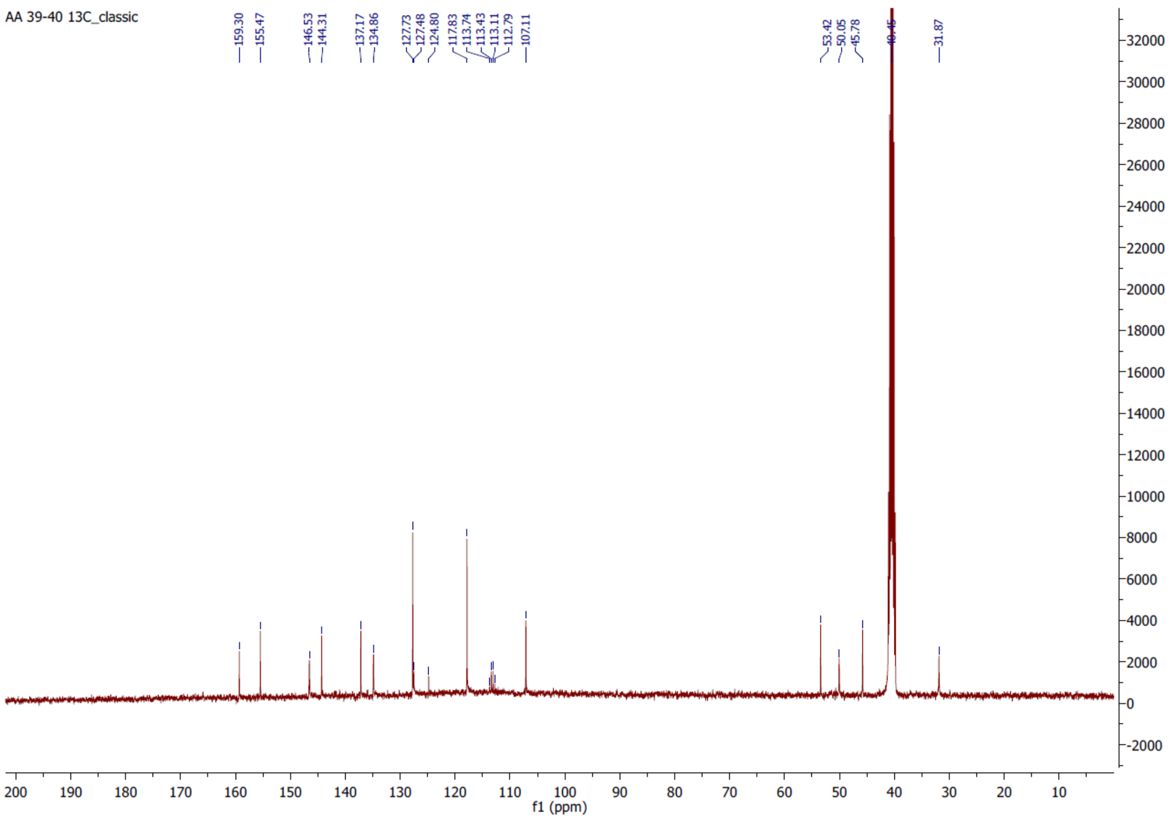

^{13}C NMR spectrum of compound **45a** (100 MHz, DMSO-d_6

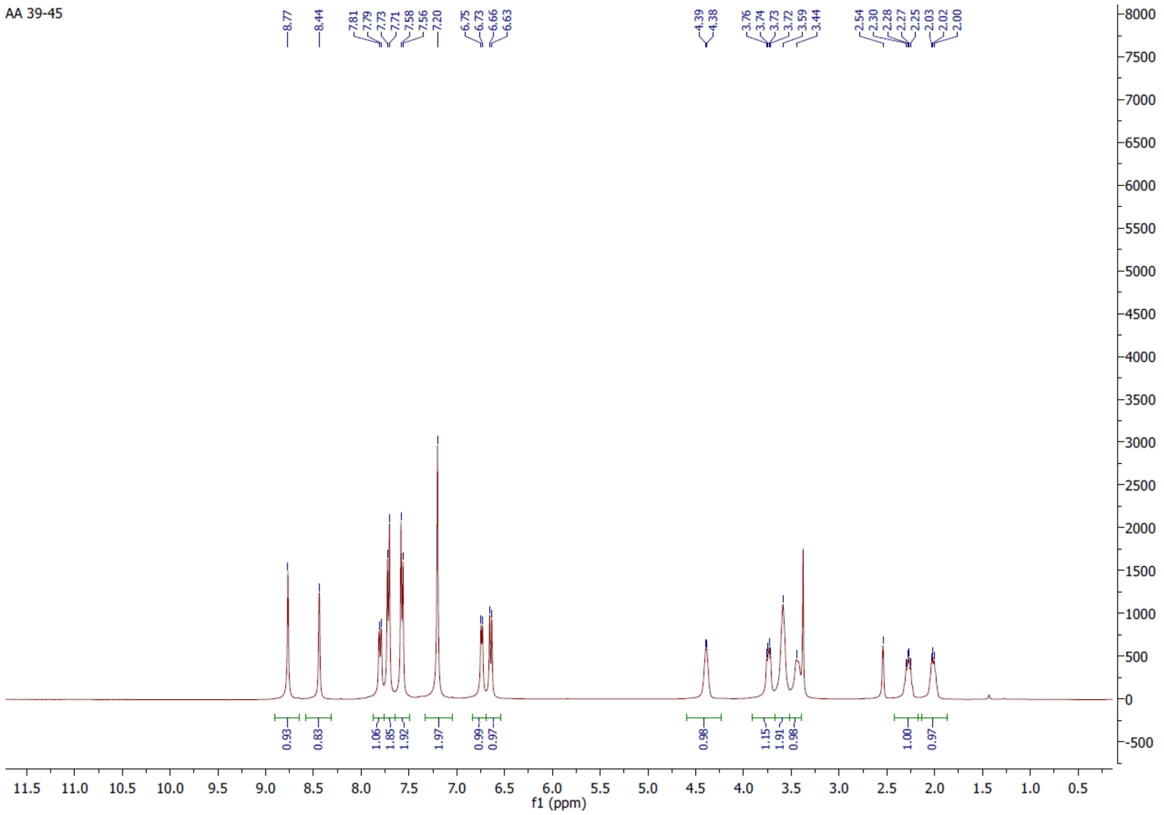

^{1}H NMR spectrum of compound **45a** (400 MHz, DMSO-d_6)

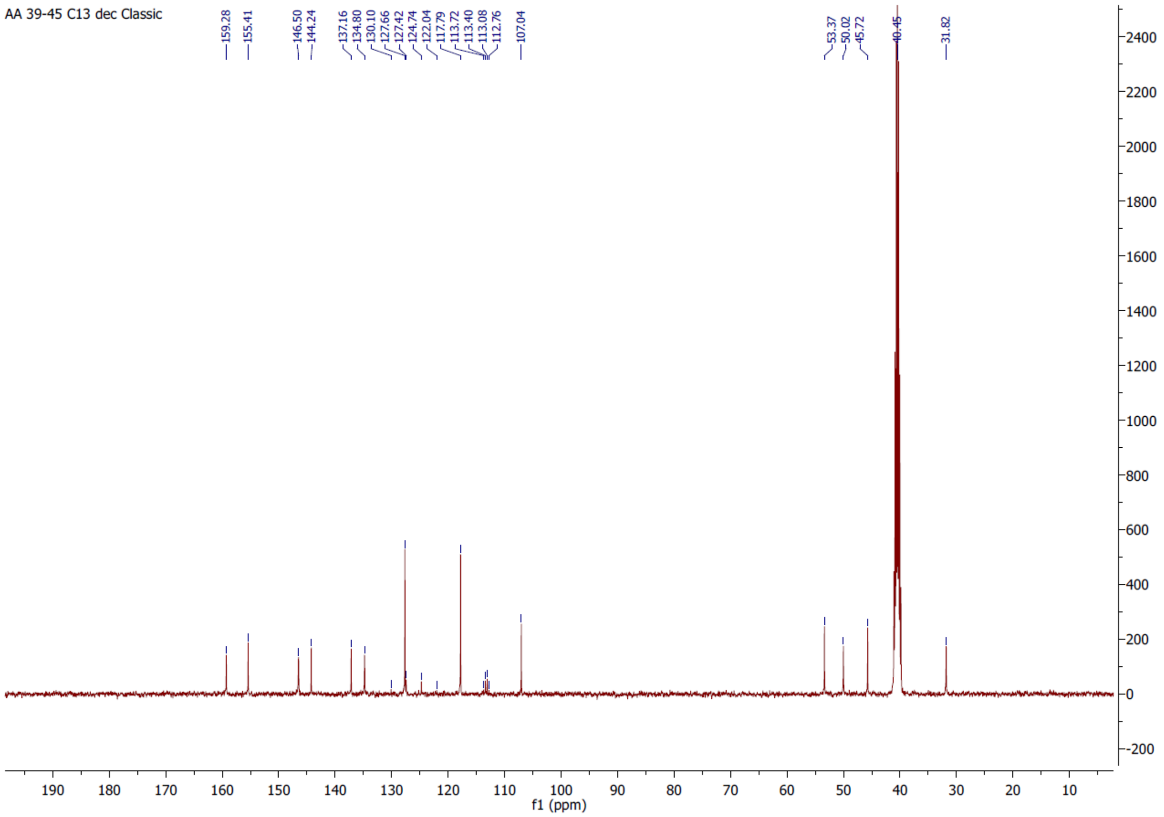

^{13}C NMR spectrum of compound **45a** (100 MHz, DMSO-d_6

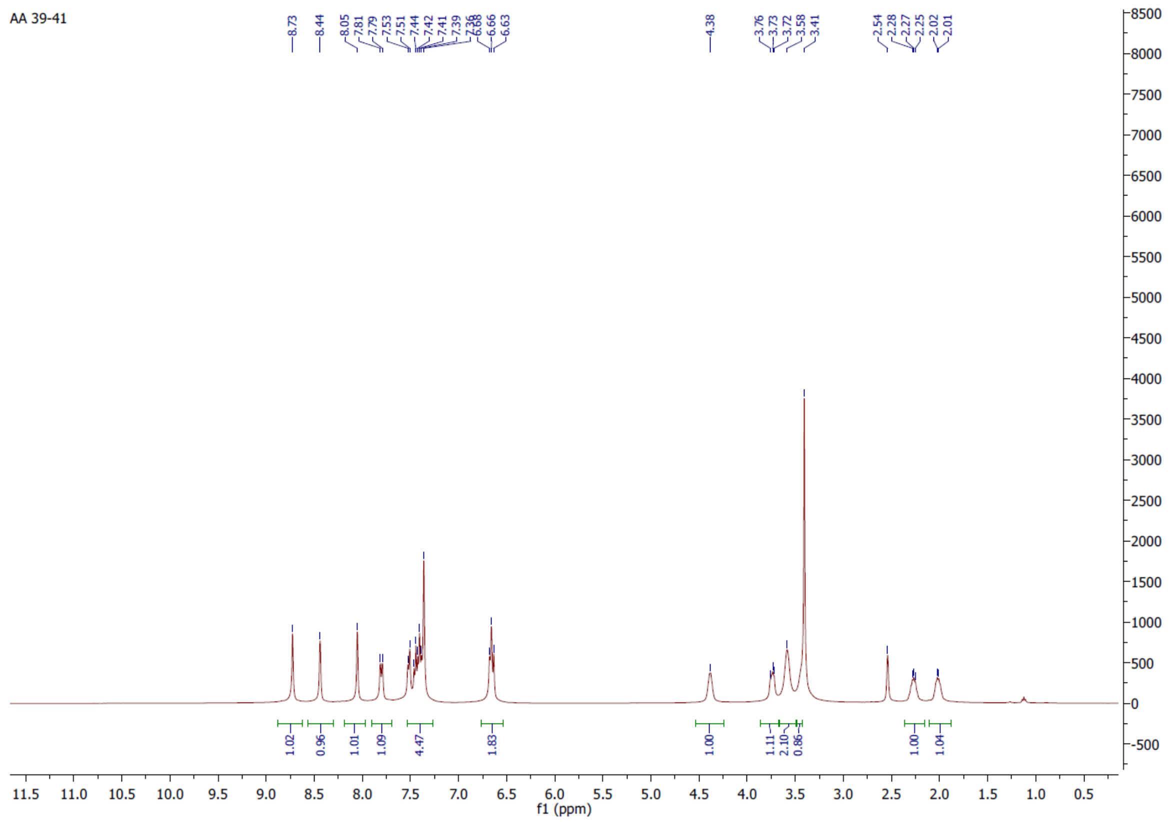

^{1}H NMR spectrum of compound **46a** (400 MHz, DMSO-d_6)

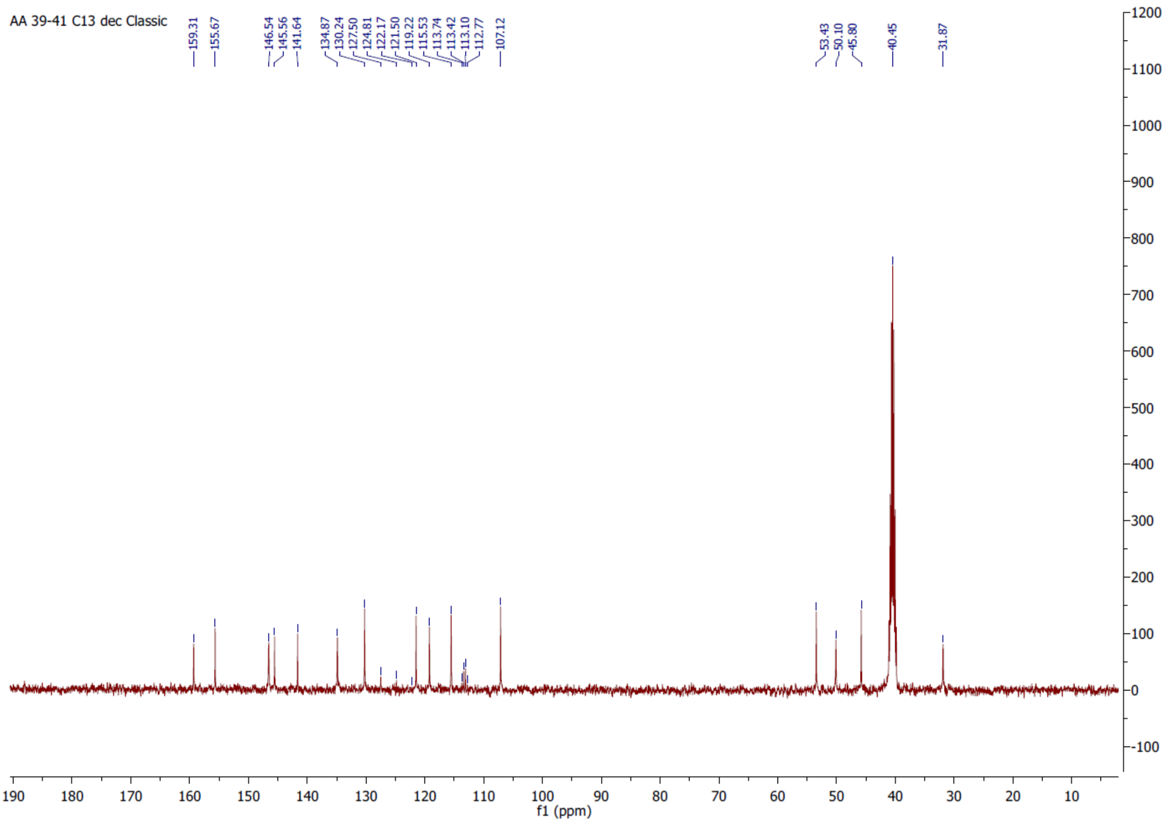

^{13}C NMR spectrum of compound **46a** (100 MHz, DMSO-d_6

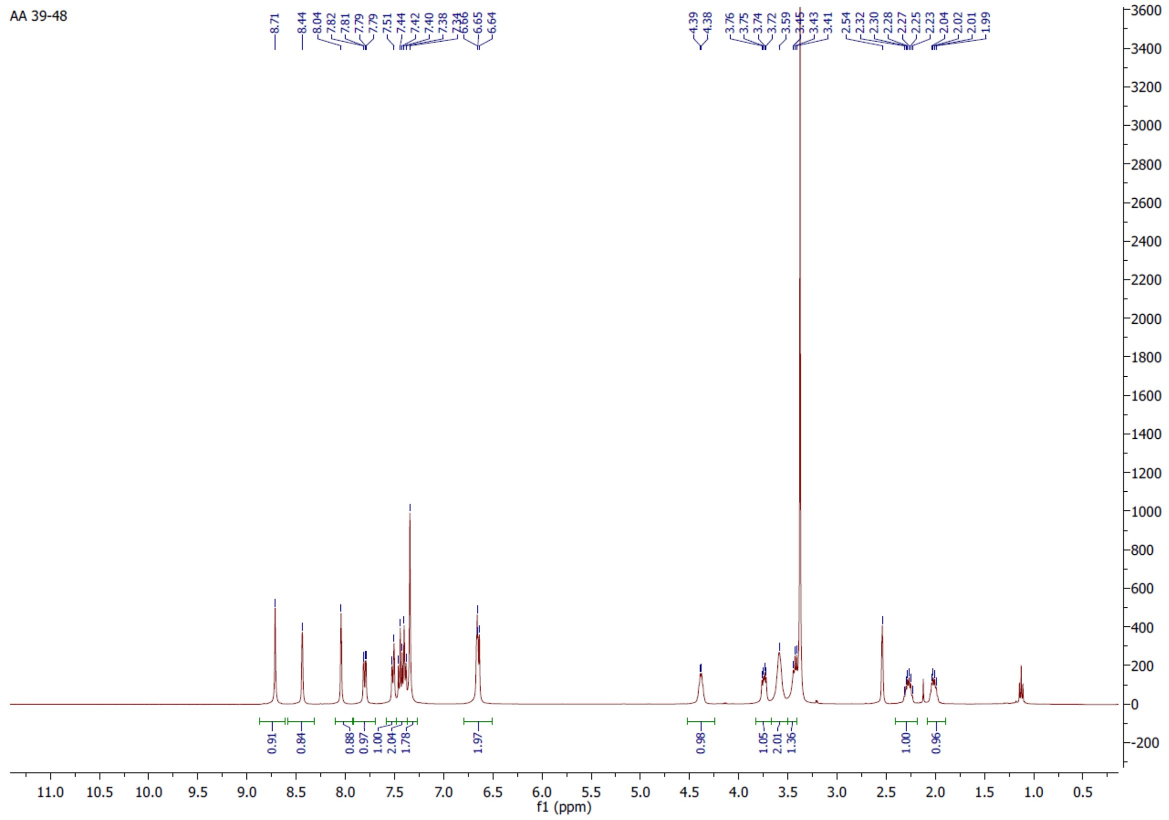

^{1}H NMR spectrum of compound **46b** (400 MHz, DMSO-d_6)

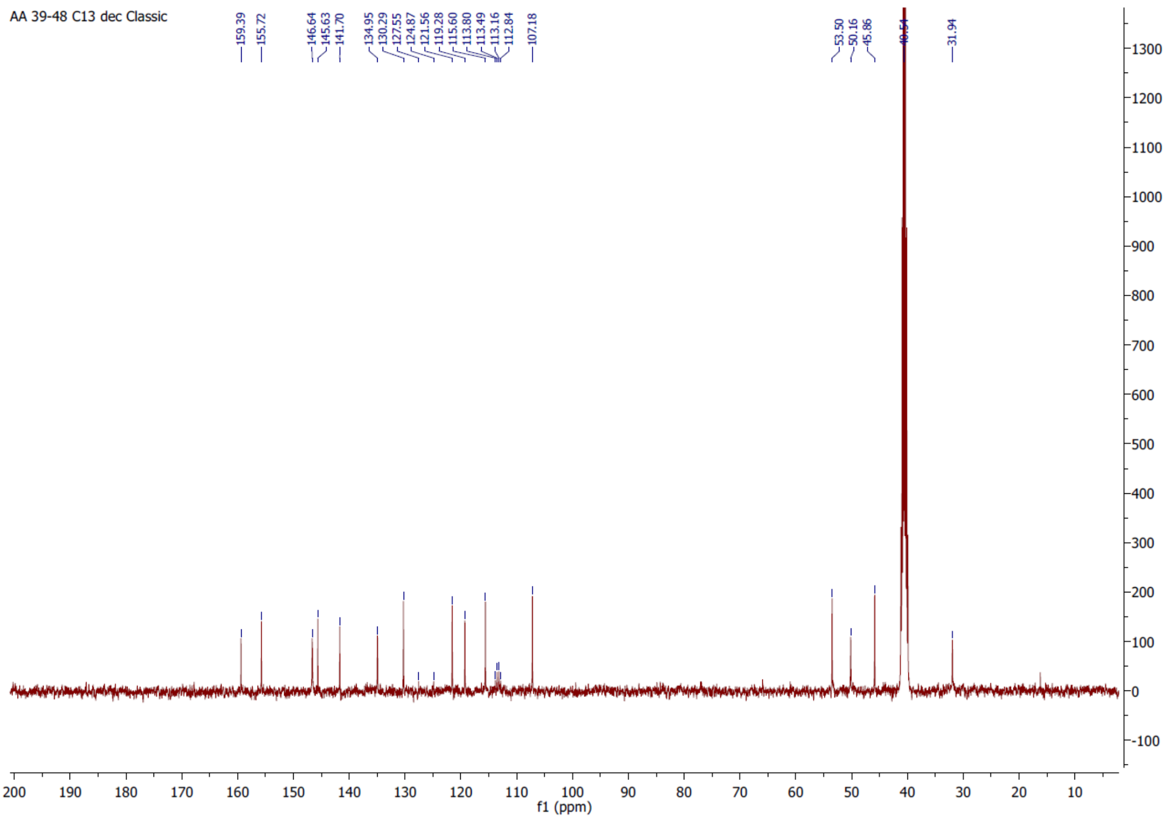

^{13}C NMR spectrum of compound **46b** (100 MHz, DMSO-d_6

Summary of Data Collection and Atomic Model Refinement Statistics for hCAII

	**hCAII + 37a**	**hCAII + 37b**
PDB ID	8BJX	8BOE
Wavelength (Å)	1.00	1.00
Space Group	P21	P21
Unit cell (a, b, c, α, β, γ) (Å,°)	42.32, 41.17, 71.67<br>90.00, 104.33, 90.00	42.58, 41.83, 72.78<br>90.00, 104.74, 90.00
Limiting resolution (Å)	48.00-1.28 (1.28-1.32)	41.83-1.55 (1.55-1.58)
Unique reflections	51575 (1619)	35726 (1716)
Rmerge (%)	13.8 (52.5)	6.5 (9.9)
Rmeas (%)	15.2 (61.7)	7.2 (11.0)
Redundancy	5.1 (3.38)	5.5 (5.3)
Completeness overall (%)	84.1 (36.1)	98.9 (97.0)
<I/σ(I)>	7.61 (2.1)	15.7 (9.5)
CC (1/2)	98.4 (83.1)	99.6 (98.5)
**Refinement statistics**		
Resolution range (Å)	48.00-1.28	41.83-1.55
Rfactor (%)	15.62	15.35
Rfree(%)	20.33	17.23
r.m.s.d. bonds(Å)	0.0208	0.0130
r.m.s.d. angles (°)	2.0588	1.9807
**Ramachandran statistics** (%)		
Most favored	97.3	97.3
additionally allowed	2.7	2.7
outlier regions	0.0	0.0
**Average B factor (Å^2)**		
All atoms	20.275	12.446
inhibitors	40.342	24.680
solvent	30.302	21.371

Figure S1:

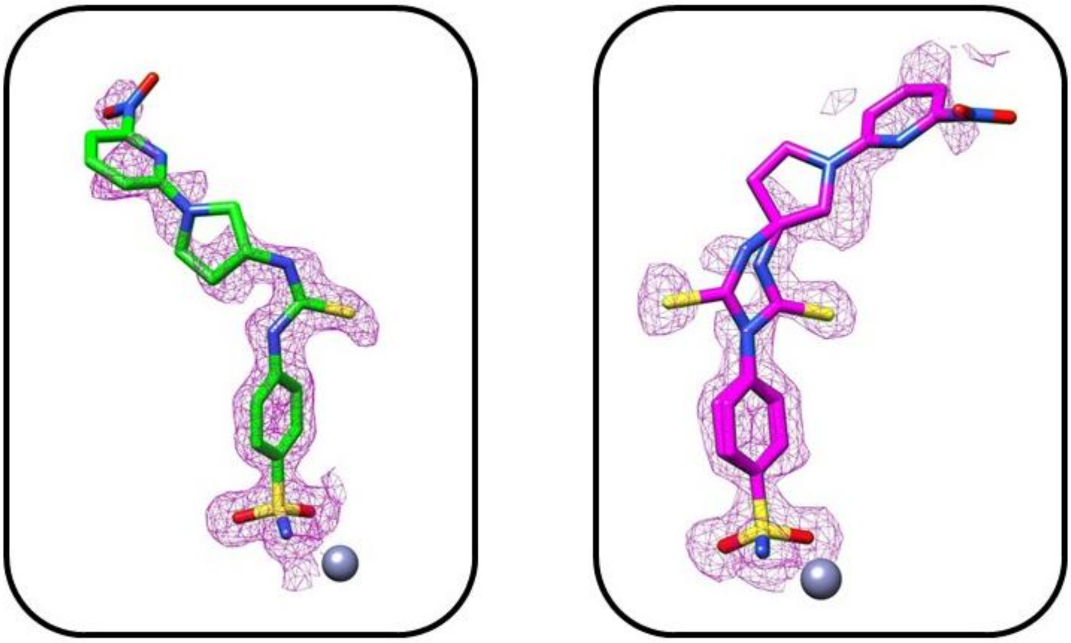

37a **37b**

Electron density of inhibitors **37a** and **37b** bound to zinc (grey) in hCA II active site. $2F_o$-F_c maps and contoured to the 1.0 σ level.
